# Direct visible-light-induced synthesis of P-stereogenic phosphine oxides under air conditions†

**DOI:** 10.1039/d2sc00036a

**Published:** 2022-04-25

**Authors:** Ying Zhang, Jia Yuan, Guanglong Huang, Hong Yu, Jinpeng Liu, Jian Chen, Sixuan Meng, Jian-Ji Zhong, Li Dang, Guang-Ao Yu, Chi-Ming Che

**Affiliations:** Key Laboratory of Pesticide & Chemical Biology, Ministry of Education, and Chemical Biology Center, College of Chemistry, Central China Normal University Wuhan 430079 P. R. China yuguang@mail.ccnu.edu.cn; Department of Chemistry, State Key Laboratory of Synthetic Chemistry, The University of Hong Kong Pokfulam Road Hong Kong P. R. China cmche@hku.hk; Department of Chemistry and Key Laboratory for Preparation and Application of Ordered Structural Materials of Guangdong Province, Shantou University, and Chemistry and Chemical Engineering Guangdong Laboratory Guangdong 515063 P. R. China

## Abstract

Over the past two decades, visible-light-induced transformations have been regarded as being among the most environmentally benign and powerful strategies for constructing complex molecules and diverse synthetic building blocks in organic synthesis. However, the development of efficient photochemical processes for assembling enantiomerically pure molecules remains a significant challenge. Herein, we describe a simple and efficient visible-light-induced C–P bond forming reaction for the synthesis of P-chiral heteroaryl phosphine oxides in moderate to high yields with excellent ee values (97–99% ee). Even in the absence of transition metal or photoredox catalysts, a variety of P-chiral heteroaryl phosphine oxides, including chiral diphosphine oxide 41, have been directly obtained under air conditions. Density functional theory (DFT) calculations have shown that the reaction involves intersystem crossing and single electron transfer to give a diradical intermediate under visible light irradiation.

## Abstract 2

We describe a simple and efficient visible-light-induced C–P bond forming reaction for the synthesis of P-chiral heteroaryl phosphine oxides in moderate to high yields with excellent ee values (97–99% ee).

## Graphical abstract

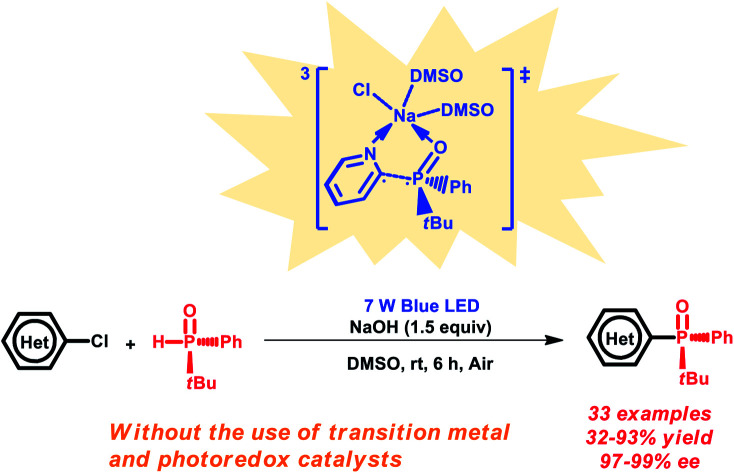

## Introduction

**Scheme 1:**
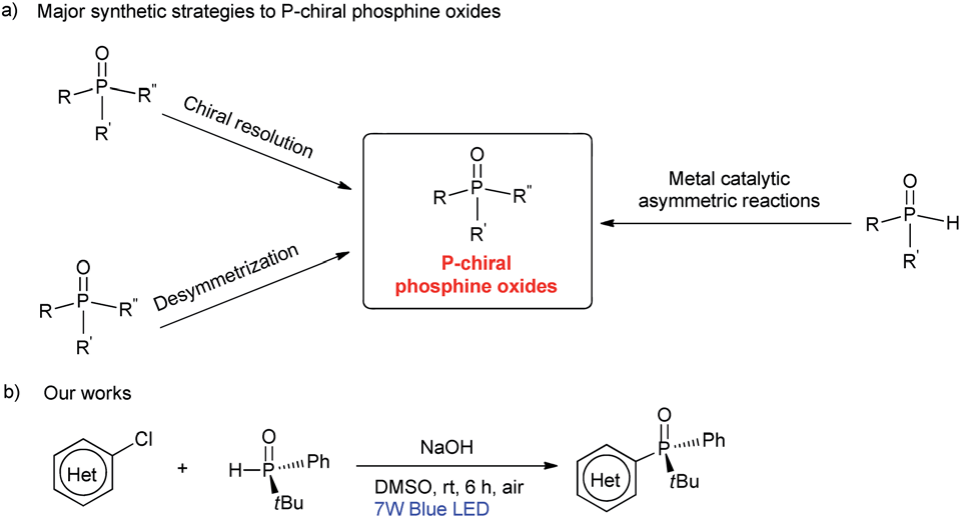
Synthesis of P-chiral phosphine oxides.

P-stereogenic phosphorus compounds are valuable ligands for enantioselective metal-catalysed reactions, that are usually used in the synthesis of pharmaceuticals and agrochemicals.^1^ In general, the synthesis of P-stereogenic phosphorus compounds frequently relies on auxiliary-based or resolution processes.^2^ These methods usually require the use of a stoichiometric amount of a chiral reagent under harsh reaction conditions, and often suffer from limited substrate scope. As more efficient alternatives, several metal-catalysed asymmetric reactions of secondary phosphines or phosphine oxides have been developed for the construction of P-chiral compounds in recent years (Scheme 1).^3–5^ However, such metal-catalysed reactions still suffer from drawbacks, such as high loadings of expensive metal catalysts and chiral ligands, and reactions need to be carried out under an atmosphere of argon or nitrogen. With these limitations in mind, it is still important to develop distinct and robust strategies that provide convenient access to a wide range of P-chiral compounds.

Visible-light-driven organic reactions have emerged as a powerful strategy for constructing C–E bonds (E = C, N, P, *etc.*) under mild and ecologically benign conditions.^6^ However, the high energy intermediates generated in photo-induced transformations often have very short lifetimes and can be difficult to stereo-control with exogenous catalysts. The application of visible-light-driven transformations to access chiral compounds is a real challenge. Over the past decade, many light-induced asymmetric transformations have been developed that operate in the presence of dual catalysis systems or a single bifunctional catalyst.^7^ Despite these significant achievements, one might concede that efficient methods for the visible-light-induced synthesis of P-chiral compounds have yet to be described. Herein, we disclose our results on the visible-light-promoted C–P cross-coupling of heteroaryl chlorides with (*R*)-*tert*-butyl(phenyl)phosphine oxide through a diradical pathway. This reaction can be performed in the absence of a transition metal or photo-redox catalyst under air conditions. In this way, we have succeeded in preparing a series of P-chiral heteroaryl phosphine oxides with high ee values (ee ≥ 97%).

Our group has long sought to develop efficient strategies for producing phosphine compounds.^4*h*,8^ In 2018, we found that unsymmetric *tert*-butyl(phenyl)phosphine oxide (*rac*-1) reacted with 2-chloropyridine to afford *rac*-2 in 54% yield in the presence of *t*BuOK under visible-light irradiation (Table 1, entry 1).^8*b*^ Subsequently, we screened the reaction conditions by employing other solvents. When conducted in DMSO, toluene, DMF, and THF, the reaction gave *rac*-2 in yields of 59%, 28%, 27%, and 40%, respectively (Table 1, entries 2–5). No product was observed using CH_2_Cl_2_ as solvent (Table 1, entry 6). Next, screening of bases revealed that NaOH was optimal in DMSO.

**Table 1:** Optimization of the reaction conditions^a^^,^^b^

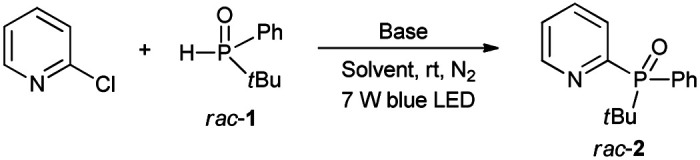				
Entry	Base	Solvent	Time (h)	Yield^b^ (%)
1	*t*BuOK	CH_3_CN	18	54
2	*t*BuOK	DMSO	18	59
3	*t*BuOK	Toluene	18	28
4	*t*BuOK	DMF	18	27
5	*t*BuOK	THF	18	40
6	*t*BuOK	DCM	18	<1
7	*t*BuONa	DMSO	18	80
8	*t*BuOLi	DMSO	18	<1
9	NaOH	DMSO	18	87
10	Na_2_CO_3_	DMSO	18	<1
11	NaOH	DMSO	6	87 (84^c^^,^^d^)
12	NaOH	DMSO	18	<1^e^

a Reaction conditions: 2-chloropyridine (0.24 mmol), *tert*-butyl(phenyl)phosphine oxide (0.20 mmol) and base (0.3 mmol) in solvent (1 mL) at room temperature under N_2_ and blue LED irradiation (7 W).

b Based on ^31^P NMR.

c Isolated yield.

d Under air.

e Reaction performed in the absence of light.

No reaction occurred when using *t*BuOLi or Na_2_CO_3_ as bases (Table 1, entries 7–10). The reaction time could be reduced to 6 h under air, with *rac*-2 being isolated in 84% yield (Table 1, entry 11). Additionally, the expected product *rac*-2 was not obtained in the absence of light (Table 1, entry 12). Based on these results, we surmised that chiral heteroaryl phosphine oxides could probably be constructed by this method. We proceeded to examine the applicability of the standard conditions for the reaction of (*R*)-*tert*-butyl(phenyl)phosphine oxide (3) with 2-chloropyridine. The desired chiral product 4 was obtained in 84% yield with 98% ee. The use of other solvents, such as DMF, toluene, and THF, resulted in 8–44% yields with 98% ee, revealing that solvent had no influence on the ee value (Table 2, entries 1–5). The reaction also gave product 4 in 52% yield with 96% ee at 120 °C in the absence of light (Table 2, entry 6). Reactions with 2-bromopyridine and 2-iodopyridine afforded 4 in yields of 75% and 27%, respectively, both with 98% ee (Table 3).

**Table 2:** The reaction of 2-chloropyridine with 3 in different solvents^a^^,^^b^

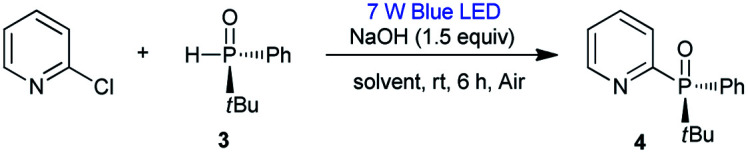			
Entry	Solvent	Yield^b^ (%)	ee (%)
1	DMSO	87 (84^c^)	98
2	Toluene	8	98
3	DMF	44	98
4	THF	29	98
5	CH_3_CN	<1	—
6^d^	DMSO	52	96

a Reaction conditions: 2-chloropyridine (0.24 mmol), 3 (0.20 mmol) and NaOH (0.3 mmol) in solvent (1 mL) at room temperature under blue LED irradiation (7 W).

b Based on ^31^P NMR.

c Isolated yield.

d Reaction performed at 120 °C in the absence of light.

**Table 3:** Visible-light-induced phosphinylation of pyridinyl halides with compound 3^a^^,^^b^

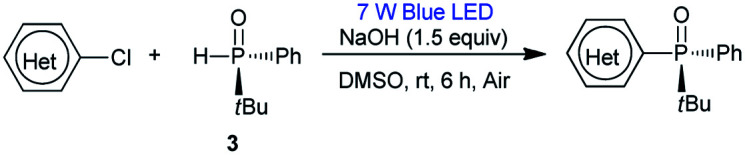
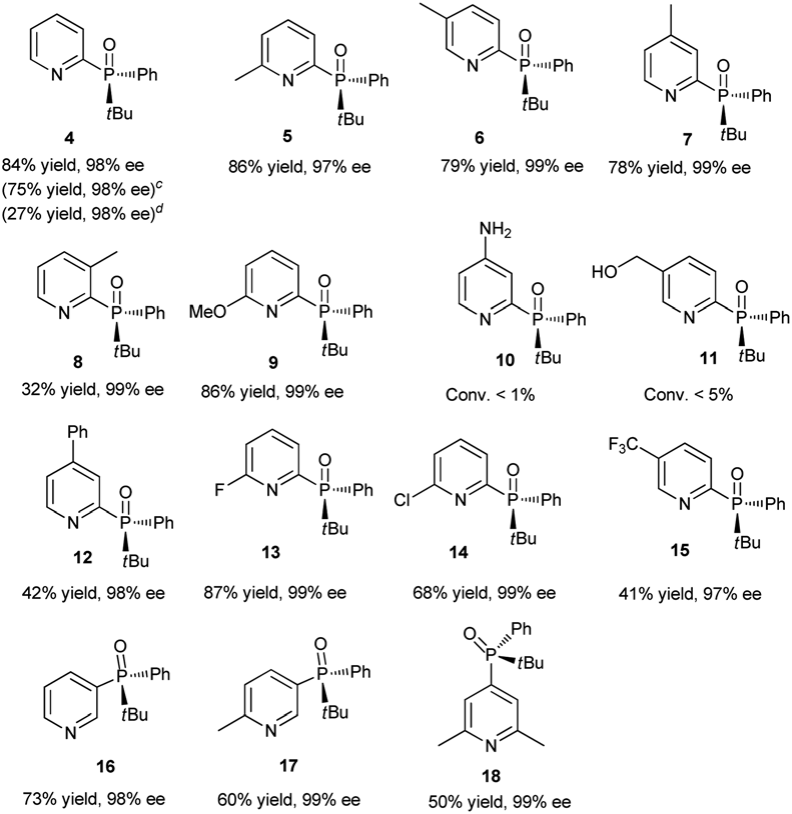

a Reaction conditions: pyridinyl chlorides (0.6 mmol), 3 (0.50 mmol) and NaOH (0.75 mmol) in DMSO (2 mL) under blue LED irradiation (7 W) for 6 h at room temperature.

b Isolated yield.

c 2-Bromopyridine as a substrate.

d 2-Iodopyridine as a substrate.

## Results and discussion

Having determined the optimal conditions, the substrate scope of the reaction was explored. As shown in Table 3, 2-chloropyridines bearing methyl, methoxy, phenyl, fluoro, chloro, and trifluoromethyl groups were phosphinylated to give the desired chiral products 5–9 and 12–15 in moderate to good yields (32–86%) with excellent ee values (97–99%). 2-Chloro-6-methylpyridine, 2-chloro-5-methylpyridine, and 2-chloro-3-methylpyridine gave 5, 6, and 8 in yields of 86%, 79%, and 32%, respectively, revealing that steric effects hinder the transformation. The expected products 10 and 11 were scarcely observed following the reactions of substrates bearing amino and hydroxymethyl groups. The partial deprotonation of –NH_2_ and –OH groups probably deactivate the substrates in the presence of NaOH. On the other hand, 3-chloropyridine, 5-chloro-2-methylpyridine, and 4-chloro-2,6-dimethylpyridine were well tolerated, and the expected products 16–18 were obtained in yields of 50–73% with ee values of 98–99%.

Next, the visible-light-induced C–P bond forming reaction was extended to other heteroaryl chlorides. Chloroquinolines, 1-chloroisoquinoline, 2-chloropyrazines, and 2-chloropyrimidines were also successfully phosphinylated to afford the expected products 19–30 in yields of 50–88% with 97–99% ee. Remarkably, heteroaryl chlorides based on bipyridyl, terpyridyl, and 1,10-phenanthrolinyl cores also proved to be compatible with the standard conditions, giving the expected products 31–35 in 55–90% yields with 97–99% ee (Table 4). Meanwhile, diphosphinylated product was not found in the reaction of 2,6-dichloropyrazine with 1 equivalent of 3, but diphosphinylated product 41 was obtained in 17% yield with 99% ee in the reaction of 2,9-dichloro-1,10-phenanthroline with 1 equivalent of 3. However, the expected products 36 and 37 were not found in the reactions of 2-bromothiophene and 2-bromofuran. When methyl(phenyl)phosphine oxide was used, 38 was scarcely observed under the optimized conditions. 2-Chloroquinoxaline and 2-chloropyrimidine reacted readily with (*R*)-cyclohexyl(phenyl)phosphine oxide gave products 39 and 40 in 60% and 50% yields with ee values of 98% and 97%, respectively. The reaction of 2,9-dichloro-1,10-phenanthroline with 2 equivalents of diphenylphosphine oxide 3 gave the chiral diphosphinylated product 41 selectively in 82% yield with 99% ee, and no *meso* product was formed. Among the various products, the absolute configuration of compound 23 was determined by X-ray crystallographic analysis.

**Table 4:** Visible-light-induced phosphinylation of heteroaryl chlorides with chiral secondary phosphine oxides^a^^,^^b^

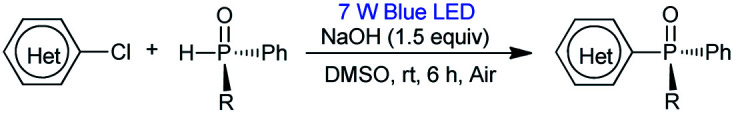
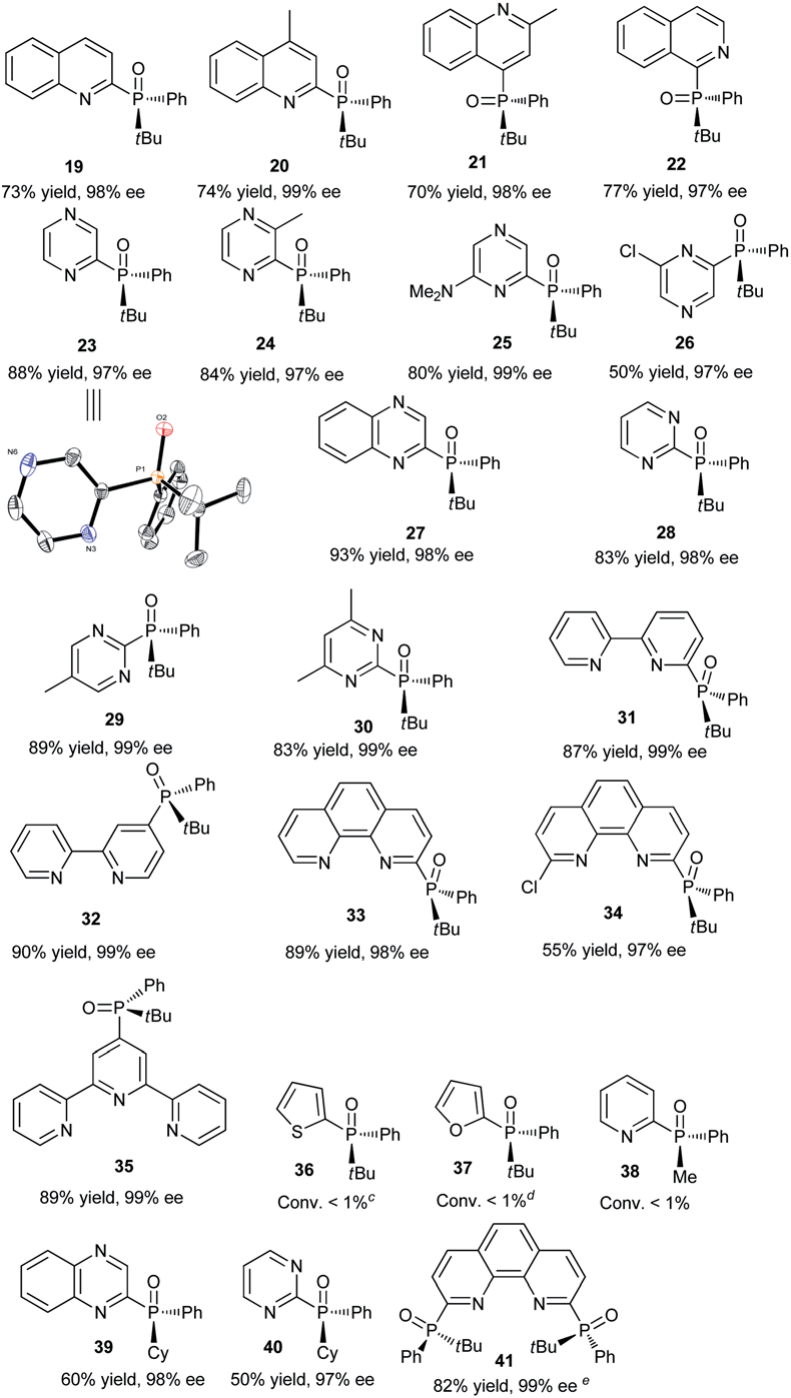

a Reaction conditions: heteroaryl chlorides (0.6 mmol), chiral secondary phosphine oxides (0.50 mmol) and NaOH (0.75 mmol) in DMSO (2 mL) under blue LED irradiation (7 W) for 6 h at room temperature.

b Isolated yield.

c The substrate is 2-bromothiophene.

d The substrate is 2-bromofuran.

e 2,9-dichloro-1,10-phenanthroline (0.5 mmol), 3 (1.0 mmol) and NaOH (1.5 mmol) in DMSO (2 mL) under blue LED irradiation (7 W) for 6 h at room temperature.

**Scheme 2:**
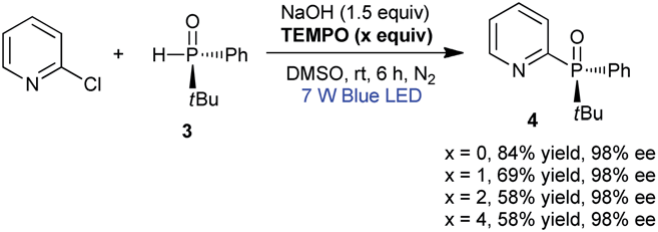
Phosphinylation of 2-chloropyridine in the presence of TEMPO.

**Scheme 3:**
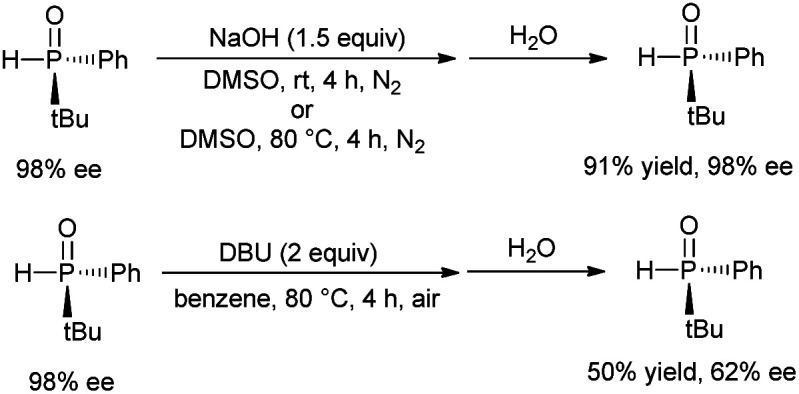
Control experiments.

**Fig. 1:**
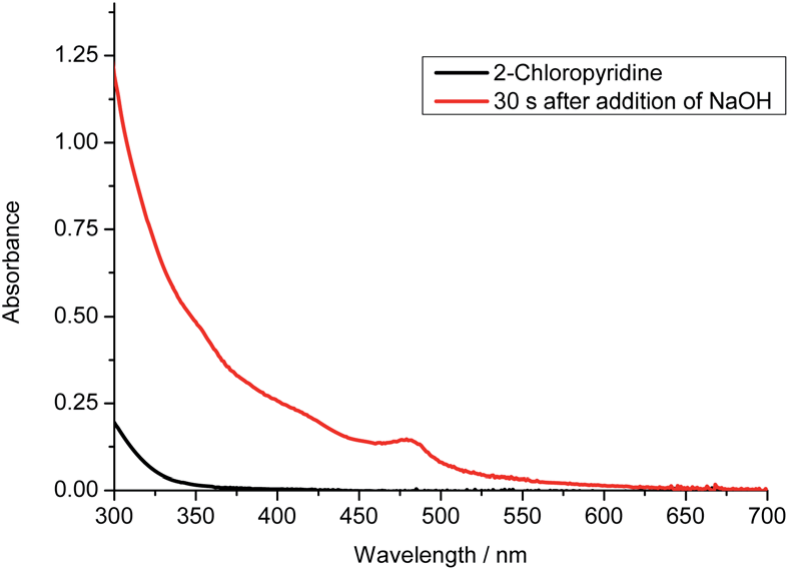
UV-vis absorption of the addition of NaOH (1 equiv.) into a DMSO solution of 2-chloropyridine (0.05 M).

For the photo-induced phosphinylation of aryl/heteroaryl halides, general mechanistic studies have suggested the generation of a reactive phosphorus-centered monoradical and its subsequent coupling with a carbon-centered radical species to construct the C–P bond.^6^ The ee value of 3 is maintained in the presence of NaOH at room temperature or 80 °C, but the ee value is decreased to 62% in the presence of DBU at 80 °C for 4 h (Scheme 3).^9^ By DFT calculations, Minnaard and co-workers suggested that phosphorus-centered monoradical species is easily racemized.^10^ In order to probe the mechanism, the reaction of 3 with 2-chloropyridine in the presence of 2,2,6,6-tetramethylpiperdine-1-oxyl (TEMPO) was examined. Even in the presence of 4 equivalents of TEMPO, approximately 58% of the desired product 4 with 98% ee was obtained (Scheme 2). On the other hand, following the addition of NaOH (1 equiv.) to a 0.05 M solution of 2-chloropyridine in DMSO, we observed an absorption band with a peak at 480 nm and a relatively weak tail spanning the range from 500 to 650 nm (Fig. 1). It is possible that a complex was formed between the pyridine substrate and NaOH in DMSO.^8*b*^

**Fig. 2:**
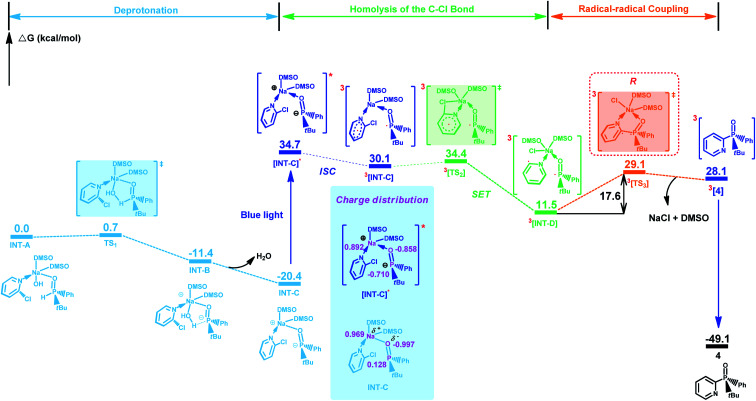
Gibbs free energy profiles for the reaction of 2-chloropyridine, 3 and NaOH in DMSO. The energies are obtained by SMD (solvent = dimethylsulfoxide) solvent model corrected free energy based on optimized geometry from ωB97XD/6-31+G(d) calculations.

Based on these results, we further performed density functional theory calculations (for computational details, see the ESI†) on the reaction of 3 with 2-chloropyridine under the standard conditions. The most favorable pathway for generating the product while maintaining the phosphine center is shown in Fig. 2. Initially, binding interaction between 2-chloropyridine, 3 and NaOH in DMSO would afford intermediate INT-A, which easily undergoes a deprotonation reaction to give intermediate INT-C. Subsequently, INT-C can be converted to ^3^[INT-C] (Δ*G*^≠^ = 50.5 kcal mol^−1^) *via* intersystem crossing (ISC) under blue light irradiation. ^3^[INT-C] undergoes a single electron transfer to give a diradical intermediate ^3^[INT-D]. The energy barrier for the generation of ^3^[TS_3_] from ^3^[INT-D] is 17.6 kcal mol^−1^. Then, product 4 is formed *via* an intramolecular radical–radical coupling reaction, which is compatible with the experimental conditions described in this work.^11^

## Conclusions

In summary, we have developed a highly enantioretentive visible-light-induced C–P coupling of (*R*)-*tert*-butyl(phenyl)phosphine oxide with heteroaryl chlorides, which represents a powerful strategy for the synthesis of P-stereogenic heteroaryl phosphine oxides under air conditions without the use of an external photosensitizer. Various heteroaryl chlorides, including challenging polypyridyl and 1,10-phenanthrolinyl chlorides, were also successfully phosphinylated to give the chiral products in moderate to high yields with excellent ee values. Mechanistic studies and DFT calculations have revealed that the key intermediate ^3^[INT-C] was generated under blue LED irradiation, and subsequently undergoes single electron transfer and intramolecular radical–radical coupling to afford product with retention of the chiral phosphine center. These findings will probably lead to expedient and green approaches for the synthesis of valuable chiral molecules.

## Data availability

All experimental, computational and crystallographic data associated with this study can be found in the article or in the ESI.†

## Author contributions

C.-M. Che, G.-A. Yu and J. Yuan conceived and designed the experiments. Y. Zhang and J. Liu performed the experiments. G. Huang, J.-J. Zhong and L. Dang performed the DFT calculations. H. Yu, J. Chen and S. Meng analyzed the experiments. C.-M. Che, G.-A. Yu, and J. Yuan revised writing of the manuscript. All authors have given approval to the final version of the manuscript.

## Conflicts of interest

There are no conflicts to declare.

## Supplementary Material

SC-013-D2SC00036A-s001

SC-013-D2SC00036A-s002
