# Supplementary material for: Direct visible-light-induced synthesis of P-stereogenic phosphine oxides under air conditions
Source: Chem Sci. 2022 Apr 25;13(22):6519–24. doi: 10.1039/d2sc00036a (PMC9172294; doi:10.1039/d2sc00036a)
Supplement: SC-013-D2SC00036A-s001 [file SC-013-D2SC00036A-s001.pdf]

Electronic Supplementary Material (ESI) for Chemical Science.
This journal is © The Royal Society of Chemistry 2022

Supporting Information

Direct visible-light-induced synthesis of P-stereogenic phosphine oxides under air conditions

Ying Zhang,‡[a] Jia Yuan,‡[b] Guanglong Huang,[c] Hong Yu,[a] Jinpeng Liu,[a] Jian Chen,[a] Sixuan Meng,[a] Jian-Ji Zhong,[c] Li Dang,*[c] Guang-Ao Yu,*[a] and Chi-Ming Che*[bc]

Table of Contents

1. General considerations. S2
2. General procedures for visible-light-promoted phosphinylation of heteroaryl halides under air....................S2
3. X-ray Structural Determination. S14
4. Computational Methods....................S15
5. References S33
6. ^{1}H, ^{13}C, ^{19}F and ^{31}P NMR spectra for all products. S34
7. HPLC spectra for all products. S85

1. General considerations

All chemical reagents were purchased from Alfa-Aesar and J&K Scientific Ltd. (*R*)-*tert*-butyl(phenyl)phosphine oxide, (±)-methyl(phenyl)phosphine oxide and (±)-cyclohexyl(phenyl)phosphine oxide were synthesized according to the published procedures. [1] Chira Resolution – Semi-Prep HPLC (chiracel AD-H column, 15% 2-Propanol in Hexane, 5 ml/min) – (*S*)-[cyclohexyl(phenyl)phosphine oxide] t = 8.304 min, (*R*)-[cyclohexyl(phenyl)phosphine oxide] t = 9.542 min. Utilizing a 10 x 250 mm column with stacked injections of variable sizes (50 mg/ml concentration), we have been able to resolve up to 100 mg of racemic monomer in a 4 hour period.

The ^{1}H, ^{13}C, ^{19}F and ^{31}P NMR spectroscopic data were recorded on Bruker Mercury Plus 400 MHz NMR spectrometers. Chemical shifts (δ) for ^{1}H and ^{13}C are referenced to internal solvent resonances and reported relative to $SiMe_4$. Chemical shifts for ^{19}F are reported relative to an external $CFCl_3$ standard. Chemical shifts for ^{31}P are reported relative to an external 85% H_3PO_4 standard. High resolution mass analysis is performed on Varian 7.0T Fourier-transform mass spectrometry with ESI resource. High performance liquid chromatography (HPLC) was performed on DIONEX Ultimate 3000 series chromatographs using a Daicel Chiracel *AD-H* (4.6 mm Ø x 250 mm) or *OJ-H* (4.6 mm Ø x 250 mm) or *AS-H* (4.6 mm Ø x 250 mm) column with *n*-hexane/*i*-PrOH as an eluent. UV-vis absorption spectrum was recorded on a Hewlett-Packard 8453 diode array spectrophotometer.

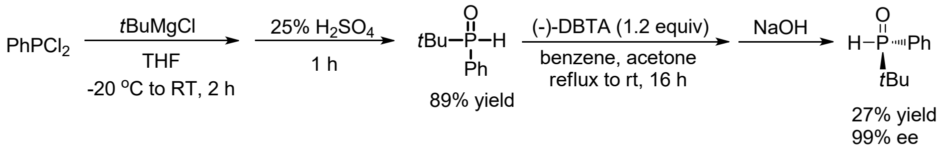

Scheme S1. Synthesis of optically pure (*R*)-*tert*-butyl(phenyl)phosphine oxide [1]

2. General procedures for visible-light-promoted phosphinylation of heteroaryl halides under air

To a round bottom flask, heteroaryl halides (0.6 mmol), (*R*)-*tert*-butyl(phenyl)phosphine oxide (0.5 mmol), NaOH (0.75mmol) and DMSO (2.5 mL) were added. The mixture was stirred at room temperature under 7 W blue LED irradiation. After stirring for 6 h, the reaction mixture was quenched with 10 mL of water and extracted with 10 mL of CH_2Cl_2 three times. The combined organic fractions were dried by $MgSO_4$, filtered and concentrated via rotary evaporation. The crude product was purified by chromatograph on silica gel (dichloromethane/acetone).

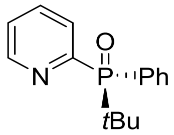

(*R*)-*tert*-butyl(phenyl)(pyridin-2-yl)phosphine oxide (4): Performed according to the general procedure to afford 109 mg (84%) of **4** as white solid. ^{1}H NMR (400 MHz, $CDCl_3$): δ 8.72 (d, *J* = 4.4 Hz, 1 H, Ar), 8.19–8.12 (m, 3 H, Ar), 7.75–7.68 (m, 1 H, Ar), 7.43–7.33 (m, 3 H, Ar), 7.32–7.27 (m, 1 H, Ar), 1.14 (d, *J* = 15.1 Hz, 9 H, $C(CH_3)_3$). ^{13}C NMR (101 MHz, $CDCl_3$): δ 156.7 (d, J_{C-P} = 117.2 Hz, Ar), 149.2 (d, J_{C-P} = 17.6 Hz, Ar), 135.9 (d, J_{C-P} = 8.5 Hz, Ar), 132.7 (d, J_{C-P} = 7.6 Hz, Ar), 131.3 (d, J_{C-P} = 2.5 Hz, Ar), 130.0 (d, J_{C-P} = 89.6 Hz, Ar), 129.2 (s, Ar), 129.0 (s, Ar), 127.8 (s, Ar), 127.7 (s, Ar), 124.9 (d, J_{C-P} = 2.9 Hz, Ar), 33.8 (d, J_{C-P} = 69.6 Hz, $C(CH_3)_3$), 24.6 (s, $C(CH_3)_3$). ^{31}P NMR (162 MHz, $CDCl_3$): δ 32.6 (s). HRMS (ESI): m/z: $[M+H]^+$ calculated for $C_{15}H_{19}NOP$: 260.1199, found: 260.1197. HPLC analysis of the product: Daicel Chiralpak AD-H column; *n*-hexane/*i*-PrOH = 90/10, flow rate = 1 mL/min, UV = 254 nm, t_{R1} = 12.392 min (minor) and t_{R2} = 13.512 min (major), ee = 98%. $[\alpha]_D^{20}$ = +73.0 (c = 1.0 in $CHCl_3$).

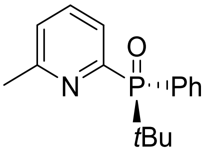

(*R*)-*tert*-butyl(6-methylpyridin-2-yl)(phenyl)phosphine oxide (5): Performed according to the general procedure to afford 117 mg (86%) of **5** as white solid. ^{1}H NMR (400 MHz, $CDCl_3$): δ 8.28–8.21 (m, 2 H, Ar), 8.06–8.01 (m, 1 H, Ar), 7.70–7.64 (m, 1 H, Ar), 7.50–7.41 (m, 3 H, Ar), 7.22 (d, *J* = 7.9 Hz, 1 H, Ar), 2.66 (s, 3 H, C*H*$_3$), 1.22 (d, *J* = 15.1 Hz, 9 H, C(C*H*$_3$)$_3$). ^{13}C NMR (101 MHz, $CDCl_3$): δ 158.1 (d, J_{C-P} = 17.6 Hz, Ar), 155.8 (d, J_{C-P} = 118.7 Hz, Ar), 136.1 (d, J_{C-P} = 9.0 Hz, Ar), 132.8 (d, J_{C-P} = 7.5 Hz, Ar), 131.3 (d, J_{C-P} = 2.7 Hz, Ar), 130.2 (d, J_{C-P} = 89.2 Hz, Ar), 127.7 (d, J_{C-P} = 10.9 Hz, Ar), 126.3 (s, Ar), 126.1 (s, Ar), 124.7 (d, J_{C-P} = 3.1 Hz, Ar), 33.8 (d, J_{C-P} = 69.6 Hz, *C*(CH$_3$)$_3$), 24.7 (s, C(*C*H$_3$)$_3$), 24.6 (s, *C*H$_3$). ^{31}P NMR (162 MHz, $CDCl_3$): δ 32.0 (s). HRMS (ESI): m/z: $[M+H]^+$ calculated for $C_{16}H_{21}NOP$: 274.1355, found: 274.1353. HPLC analysis of the product: Daicel Chiralpak AD-H column; *n*-hexane/*i*-PrOH = 85/15, flow rate = 1 mL/min, UV = 254 nm, t_{R1} = 5.965 min (major) and t_{R2} = 6.979 min (minor), ee = 97%. $[\alpha]_D^{20}$ = +13.0 (c = 1.0 in $CHCl_3$).

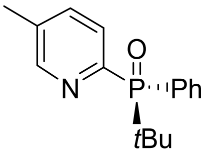

(*R*)-*tert*-butyl(5-methylpyridin-2-yl)(phenyl)phosphine oxide (6): Performed according to the general procedure to afford 108 mg (79%) of **6** as white solid. ^{1}H NMR (400 MHz, $CDCl_3$): δ 8.64 (s, 1 H, Ar), 8.30–8.18 (m, 2 H, Ar), 8.16–8.10 (m, 1 H, Ar), 7.61 (d, *J* = 7.8 Hz, 1 H, Ar), 7.51–7.41 (m, 3 H, Ar), 2.39 (s, 3 H, C*H*$_3$), 1.21 (d, *J* = 15.1 Hz, 9 H, C(C*H*$_3$)$_3$). ^{13}C NMR (101 MHz, $CDCl_3$): δ 153.4 (d, J_{C-P} = 119.8 Hz, Ar), 150.1 (d, J_{C-P} = 18.1 Hz, Ar), 136.3 (d, J_{C-P} = 9.0 Hz, Ar), 135.0 (d, J_{C-P} = 3.1 Hz, Ar), 132.8 (d, J_{C-P} = 7.6 Hz, Ar), 131.3 (d, J_{C-P} = 2.7 Hz, Ar), 130.3 (d, J_{C-P} = 89.6 Hz, Ar), 128.9 (s, Ar), 128.7 (s, Ar), 127.8 (d, J_{C-P} = 10.9 Hz, Ar), 33.8 (d, J_{C-P} = 69.8 Hz, *C*(CH$_3$)$_3$), 24.6 (s, C(*C*H$_3$)$_3$), 18.6 (d, J_{C-P} = 1.4 Hz, *C*H$_3$). ^{31}P NMR (162 MHz, $CDCl_3$): δ 32.9 (s). HRMS (ESI): m/z: $[M+H]^+$ calculated for $C_{16}H_{21}NOP$: 274.1355, found: 274.1352. HPLC analysis of the product: Daicel Chiralpak AD-H column; *n*-hexane/*i*-PrOH = 85/15, flow rate = 1 mL/min, UV = 254 nm, t_{R1} = 11.099 min (major) and t_{R2} = 22.752 min (minor), ee = 99%. $[\alpha]_D^{20}$ = +133.0 (c = 1.0 in $CHCl_3$).

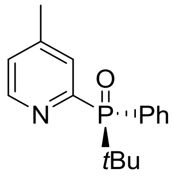

(*R*)-*tert*-butyl(4-methylpyridin-2-yl)(phenyl)phosphine oxide (7): Performed according to the general procedure to afford 107 mg (78%) of **7** as white solid. ^{1}H NMR (400 MHz, $CDCl_3$): δ 8.68–8.62 (m, 1 H, Ar), 8.22 (t, *J* = 8.1 Hz, 2 H, Ar), 8.10 (d, *J* = 2.9 Hz, 1 H, Ar), 7.50–7.41 (m, 3 H, Ar), 7.19 (s, 1 H, Ar), 2.38 (d, *J* = 2.5 Hz, 3 H, C*H*$_3$), 1.27–1.20 (m, 9 H, C(C*H*$_3$)$_3$). ^{13}C NMR (101 MHz, $CDCl_3$): δ 156.2 (d, J_{C-P} = 117.3 Hz, Ar), 149.2 (d, J_{C-P} = 18.5 Hz, Ar), 147.3 (d, J_{C-P} = 8.7 Hz, Ar), 132.7 (d, J_{C-P} = 7.6 Hz, Ar), 131.3 (d, J_{C-P} = 2.4 Hz, Ar), 130.2 (d, J_{C-P} = 89.4 Hz, Ar), 130.0 (d, J_{C-P} = 17.0 Hz, Ar), 127.7 (d, J_{C-P} = 10.9 Hz, Ar), 125.8 (d, J_{C-P} = 2.7 Hz, Ar), 33.8 (d, J_{C-P} = 69.6 Hz, *C*(CH$_3$)$_3$), 24.6 (s, C(*C*H$_3$)$_3$), 21.0 (s, *C*H$_3$). ^{31}P NMR (162 MHz, $CDCl_3$): δ 32.9 (s). HRMS (ESI): m/z: $[M+H]^+$ calculated for $C_{16}H_{21}NOP$: 274.1355, found: 274.1351. HPLC analysis of the product: Daicel Chiralpak AD-H column; *n*-hexane/*i*-PrOH = 98/2, flow rate = 1 mL/min, UV = 254 nm, t_{R1} = 42.386 min (minor) and t_{R2} = 45.219 min (major), ee = 99%. $[\alpha]_D^{20}$ = +25.0 (c = 1.0 in $CHCl_3$).

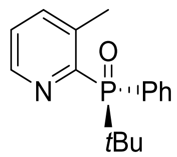

(*R*)-*tert*-butyl(3-methylpyridin-2-yl)(phenyl)phosphine oxide (8): Performed according to the general procedure to afford 43 mg (32%) of **8** as white solid. ^{1}H NMR (400 MHz, $CDCl_3$): δ 8.58 (s, 1 H, Ar), 7.91 (t, *J* = 8.3 Hz, 2 H, Ar), 7.48 (d, *J* = 4.8 Hz, 2 H, Ar), 7.43 (d, *J* = 7.4 Hz, 2 H, Ar), 7.27 (d, *J* = 2.6 Hz, 1 H, Ar), 2.51 (s, 3 H, CH_3), 1.36–1.30 (m, 9 H, $C(CH_3)_3$). ^{13}C NMR (101 MHz, $CDCl_3$): δ 154.3 (d, $J_{C\text{-}P}$ = 120.7 Hz, Ar), 145.5 (d, $J_{C\text{-}P}$ = 18.6 Hz, Ar), 140.9 (d, $J_{C\text{-}P}$ = 18.4 Hz, Ar), 139.3 (d, $J_{C\text{-}P}$ = 8.0 Hz, Ar), 132.7 (d, $J_{C\text{-}P}$ = 8.0 Hz, Ar), 131.3 (d, $J_{C\text{-}P}$ = 86.7 Hz, Ar), 131.2 (d, $J_{C\text{-}P}$ = 2.7 Hz, Ar), 127.8 (d, $J_{C\text{-}P}$ = 10.8 Hz, Ar), 124.8 (d, $J_{C\text{-}P}$ = 3.1 Hz, Ar), 35.0 (d, $J_{C\text{-}P}$ = 71.1 Hz, $C(CH_3)_3$), 25.0 (s, $C(CH_3)_3$), 19.3 (s, CH_3). ^{31}P NMR (162 MHz, $CDCl_3$): δ 38.7 (s). HRMS (ESI): m/z: $[M+H]^+$ calculated for $C_{16}H_{21}NOP$: 274.1355, found: 274.1354. HPLC analysis of the product: Daicel Chiralpak AD-H column; *n*-hexane/*i*-PrOH = 85/15, flow rate = 1 mL/min, UV = 254 nm, t_{R1} = 4.832 min (major) and t_{R2} = 5.279 min (minor), ee = 99%. $[\alpha]_D^{20}$ = +52.0 (c = 1.0 in $CHCl_3$).

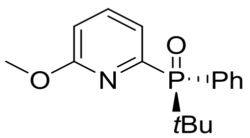

(*R*)-*tert*-butyl(6-methoxypyridin-2-yl)(phenyl)phosphine oxide (9): Performed according to the general procedure to afford 124 mg (86%) of **9** as white solid. ^{1}H NMR (400 MHz, $CDCl_3$): δ 8.25–8.18 (m, 2 H, Ar), 7.84 (t, *J* = 6.5 Hz, 1 H, Ar), 7.72–7.65 (m, 1 H, Ar), 7.52–7.42 (m, 3 H, Ar), 6.91–6.81 (m, 1 H, Ar), 4.05 (s, 3 H, OCH_3), 1.25 (d, *J* = 15.1 Hz, 9 H, $C(CH_3)_3$). ^{13}C NMR (101 MHz, $CDCl_3$): δ 163.2 (d, $J_{C\text{-}P}$ = 18.0 Hz, Ar), 153.0 (d, $J_{C\text{-}P}$ = 117.8 Hz, Ar), 138.5 (d, $J_{C\text{-}P}$ = 10.0 Hz, Ar), 132.5 (d, $J_{C\text{-}P}$ = 7.6 Hz, Ar), 131.4 (d, $J_{C\text{-}P}$ = 2.5 Hz, Ar), 130.1 (d, $J_{C\text{-}P}$ = 89.8 Hz, Ar), 127.8 (d, $J_{C\text{-}P}$ = 10.9 Hz, Ar), 123.1 (d, $J_{C\text{-}P}$ = 16.4 Hz, Ar), 113.4 (d, $J_{C\text{-}P}$ = 2.5 Hz, Ar), 53.9 (s, OCH_3), 33.7 (d, $J_{C\text{-}P}$ = 69.9 Hz, $C(CH_3)_3$), 24.6 (s, $C(CH_3)_3$). ^{31}P NMR (162 MHz, $CDCl_3$): δ 32.9 (s). HRMS (ESI): m/z: $[M+H]^+$ calculated for $C_{16}H_{21}NO_2P$: 290.1304, found: 290.1303. HPLC analysis of the product: Daicel Chiralpak AD-H column; *n*-hexane/*i*-PrOH = 85/15, flow rate = 1 mL/min, UV = 254 nm, t_{R1} = 5.652 min (major) and t_{R2} = 6.192 min (minor), ee = 99%. $[\alpha]_D^{20}$ = +56.0 (c = 1.0 in $CHCl_3$).

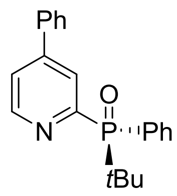

(*R*)-*tert*-butyl(phenyl)(4-phenylpyridin-2-yl)phosphine oxide (12): Performed according to the general procedure to afford 70 mg (42%) of **12** as white solid. ^{1}H NMR (400 MHz, $CDCl_3$): δ 8.80–8.75 (m, 1 H, Ar), 8.46–8.41 (m, 1 H, Ar), 8.19 (t, *J* = 8.6 Hz, 2 H, Ar), 7.64 (d, *J* = 6.7 Hz, 2 H, Ar), 7.54 (d, *J* = 2.1 Hz, 1 H, Ar), 7.44–7.36 (m, 6 H, Ar), 1.22–1.60 (m, 9 H, $C(CH_3)_3$). ^{13}C NMR (101 MHz, $CDCl_3$): δ 156.2 (d, $J_{C\text{-}P}$ = 116.7 Hz, Ar), 148.8 (d, $J_{C\text{-}P}$ = 18.3 Hz, Ar), 147.3 (d, $J_{C\text{-}P}$ = 8.7 Hz, Ar), 136.3 (s, Ar), 131.8 (d, $J_{C\text{-}P}$ = 7.6 Hz, Ar), 130.4 (d, $J_{C\text{-}P}$ = 2.7 Hz, Ar), 129.0 (d, $J_{C\text{-}P}$ = 89.7 Hz, Ar), 128.4 (s, Ar), 128.1 (s, Ar), 126.8 (d, $J_{C\text{-}P}$ = 11.0 Hz, Ar), 126.1 (s, Ar), 125.9 (d, $J_{C\text{-}P}$ = 17.5 Hz, Ar), 121.5 (d, $J_{C\text{-}P}$ = 3.0 Hz, Ar), 32.9 (d, $J_{C\text{-}P}$ = 69.5 Hz, $C(CH_3)_3$), 23.7 (s, $C(CH_3)_3$). ^{31}P NMR (162 MHz, $CDCl_3$): δ 33.2 (s). HRMS (ESI): m/z: $[M+H]^+$ calculated for $C_{21}H_{23}NOP$: 336.1512, found: 336.1508. HPLC analysis of the product: Daicel Chiralpak AD-H column; *n*-hexane/*i*-PrOH = 85/15, flow rate = 1 mL/min, UV = 254 nm, t_{R1} = 9.312 min (minor) and t_{R2} = 19.099 min (major), ee = 98%. $[\alpha]_D^{20}$ = -86.0 (c = 1.0 in $CHCl_3$).

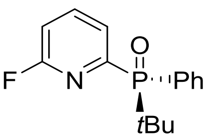

(*R*)-*tert*-butyl(6-fluoropyridin-2-yl)(phenyl)phosphine oxide (13): Performed according to the general procedure to afford 120 mg (87%) of **13** as white solid. ^{1}H NMR (400 MHz, $CDCl_3$): δ 8.23–8.15 (m, 3 H, Ar), 7.82–7.75 (m, 1 H, Ar), 7.54–7.40 (m, 4 H, Ar), 1.23 (d, J = 15.4 Hz, 9 H, C(CH_3)$_3$). ^{13}C NMR (101 MHz, $CDCl_3$): δ 157.4 (d, $J_{C\text{-}F}$ = 113.3 Hz, Ar), 151.1 (d, $J_{C\text{-}P}$ = 19.0 Hz, Ar), 138.7 (d, $J_{C\text{-}P}$ = 8.7 Hz, Ar), 132.7 (d, $J_{C\text{-}P}$ = 7.7 Hz, Ar), 131.7 (d, $J_{C\text{-}P}$ = 2.8 Hz, Ar), 129.2 (d, $J_{C\text{-}P}$ = 90.7 Hz, Ar), 128.1 (s, Ar), 128.0 (s, Ar), 127.8 (d, $J_{C\text{-}P}$ = 15.8 Hz, Ar), 126.0 (d, $J_{C\text{-}P}$ = 2.6 Hz, Ar), 34.0 (d, $J_{C\text{-}P}$ = 69.9 Hz, *C*(CH_3)$_3$), 24.5 (s, C(*C*H_3)$_3$). ^{31}P NMR (162 MHz, $CDCl_3$): δ 32.4 (s). ^{19}F NMR (376 MHz, $CDCl_3$): δ -65.5 (s). HRMS (ESI): m/z: [M+H]$^+$ calculated for $C_{15}H_{18}FNOP$: 278.1105, found: 278.1105. HPLC analysis of the product: Daicel Chiralpak AD-H column; *n*-hexane/*i*-PrOH = 85/15, flow rate = 1 mL/min, UV = 254 nm, t_{R1} = 7.739 min (major) and t_{R2} = 8.505 min (minor), ee = 99%. $[\alpha]_D^{20}$ = +87.0 (c = 1.0 in $CHCl_3$).

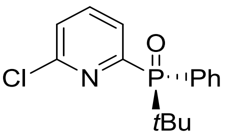

(*R*)-*tert*-butyl(6-chloropyridin-2-yl)(phenyl)phosphine oxide (14): Performed according to the general procedure to afford 100 mg (68%) of **14** as white solid. ^{1}H NMR (400 MHz, $CDCl_3$): δ 8.23–8.15 (m, 3 H, Ar), 7.81–7.75 (m, 1 H, Ar), 7.55–7.45 (m, 3 H, Ar), 7.42 (d, J = 8.1 Hz, 1 H, Ar), 1.23 (d, J = 15.4 Hz, 9 H, C(CH_3)$_3$). ^{13}C NMR (101 MHz, $CDCl_3$): δ 157.5 (d, $J_{C\text{-}P}$ = 113.2 Hz, Ar), 151.1 (d, $J_{C\text{-}P}$ = 18.7 Hz, Ar), 138.7 (d, $J_{C\text{-}P}$ = 8.7 Hz, Ar), 132.7 (d, $J_{C\text{-}P}$ = 7.7 Hz, Ar), 131.7 (d, $J_{C\text{-}P}$ = 2.7 Hz, Ar), 129.3 (d, $J_{C\text{-}P}$ = 90.6 Hz, Ar), 128.1 (s, Ar), 128.0 (s, Ar), 127.9 (s, Ar), 127.8 (s, Ar), 126.0 (d, $J_{C\text{-}P}$ = 2.5 Hz, Ar), 34.0 (d, $J_{C\text{-}P}$ = 69.9 Hz, *C*(CH_3)$_3$), 24.6 (s, C(*C*H_3)$_3$) ^{31}P NMR (162 MHz, $CDCl_3$): δ 32.3 (s). HRMS (ESI): m/z: [M+H]$^+$ calculated for $C_{15}H_{18}ClNOP$: 294.0809, found: 294.0808. HPLC analysis of the product: Daicel Chiralpak AD-H column; *n*-hexane/*i*-PrOH = 85/15, flow rate = 1 mL/min, UV = 254 nm, t_{R1} = 5.925 min (major) and t_{R2} = 6.412 min (minor), ee = 99%. $[\alpha]_D^{20}$ = +283.0 (c = 1.0 in $CHCl_3$).

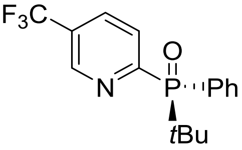

(*R*)-*tert*-butyl(phenyl)(5-(trifluoromethyl)pyridin-2-yl)phosphine oxide (15): Performed according to the general procedure to afford 67 mg (41%) of **15** as yellow solid ^{1}H NMR (400 MHz, $CDCl_3$): δ 9.02 (s, 1 H, Ar), 8.37–8.32 (m, 1 H, Ar), 8.14–8.09 (m, 2 H, Ar), 8.00 (d, J = 8.1 Hz, 1 H, Ar), 7.47–7.38 (m, 3 H, Ar), 1.17 (d, J = 15.4 Hz, 9 H, C(CH_3)$_3$). ^{13}C NMR (101 MHz, $CDCl_3$): δ 161.91 (d, $J_{C\text{-}P}$ = 1.3 Hz, Ar), 160.80 (d, $J_{C\text{-}P}$ = 1.3 Hz, Ar), 146.08 (m, Ar), 145.91 (m, Ar), 133.11 (m, Ar), 132.74 (d, $J_{C\text{-}P}$ = 7.8 Hz, Ar), 131.81 (d, $J_{C\text{-}P}$ = 2.8 Hz, Ar), 128.94 (m, Ar), 128.04 (d, $J_{C\text{-}P}$ = 11.1 Hz, Ar), 127.73 (d, $J_{C\text{-}P}$ = 2.9 Hz, Ar), 127.39 (d, $J_{C\text{-}P}$ = 2.9 Hz, Ar), 123.2 (q, $J_{C\text{-}F}$ = 272.7Hz, *C*F_3), 34.08 (d, $J_{C\text{-}P}$ = 69.7 Hz, *C*(CH_3)$_3$), 24.5 (s, C(*C*H_3)$_3$). ^{19}F NMR (376 MHz, $CDCl_3$): δ -62.7 (s). ^{31}P NMR (162 MHz, $CDCl_3$): δ 33.0 (s). HRMS (ESI): m/z: [M+H]$^+$ calculated for $C_{16}H_{18}F_3NOP$: 328.1073, found: 328.1071. HPLC analysis of the product: Daicel Chiralpak AD-H column; *n*-hexane/*i*-PrOH = 90/10, flow rate = 1 mL/min, UV = 254 nm, t_{R1} = 7.925 min (major) and t_{R2} = 22.659 min (minor), ee = 97%. $[\alpha]_D^{20}$ = +36.0 (c = 1.0 in $CHCl_3$).

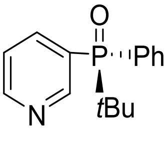

(*R*)-*tert*-butyl(phenyl)(pyridin-3-yl)phosphine oxide (16): Performed according to the general procedure to afford 95 mg (73%) of **16** as white solid. ^{1}H NMR (400 MHz, $CDCl_3$): δ 9.06 (s, 1 H, Ar), 8.68 (s, 1 H, Ar), 8.28–8.21 (m, 1 H, Ar), 7.90–7.83 (m, 2 H, Ar), 7.50–7.42 (m, 3 H, Ar), 7.40–7.35 (m, 1 H, Ar), 1.19 (d, J = 15.3 Hz, 9 H, $C(CH_3)_3$). ^{13}C NMR (101 MHz, $CDCl_3$): δ 152.1 (d, $J_{C\text{-}P}$ = 1.6 Hz, Ar), 152.0 (d, $J_{C\text{-}P}$ = 9.9 Hz, Ar), 140.4 (d, $J_{C\text{-}P}$ = 5.9 Hz, Ar), 132.1 (s), 132.0 (s), 131.9 (s), 130.6 (s, Ar), 129.7 (s, Ar), 128.6 (s, Ar), 128.5 (s, Ar), 123.5 (d, $J_{C\text{-}P}$ = 6.3 Hz, Ar), 34.1 (d, $J_{C\text{-}P}$ = 71.4 Hz, $C(CH_3)_3$), 25.0 (s, $C(CH_3)_3$). ^{31}P NMR (162 MHz, $CDCl_3$): δ 37.3 (s). HRMS (ESI): m/z: $[M+H]^+$ calculated for $C_{15}H_{19}NOP$: 260.1199, found: 260.1198. HPLC analysis of the product: Daicel Chiralpak OJ-H column; *n*-hexane/*i*-PrOH = 95/05, flow rate = 1 mL/min, UV = 254 nm, t_{R1} = 11.859 min (minor) and t_{R2} = 13.179 min (major), ee = 98%. $[\alpha]_D^{20}$ = -21.0 (c = 1.0 in $CHCl_3$).

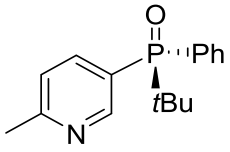

(*R*)-*tert*-butyl(6-methylpyridin-3-yl)(phenyl)phosphine oxide (17): Performed according to the general procedure to afford 82 mg (60%) of **17** as white solid. ^{1}H NMR (400 MHz, $CDCl_3$): δ 9.05–8.97 (m, 1 H, Ar), 8.24–8.14 (m, 1 H, Ar), 7.96–7.87 (m, 2 H, Ar), 7.56–7.47 (m, 3 H, Ar), 7.29 (s, 1 H, Ar), 2.62 (s, 3 H, CH_3), 1.25 (d, J = 15.3 Hz, 9 H, $C(CH_3)_3$). ^{13}C NMR (101 MHz, $CDCl_3$): δ 161.6 (d, $J_{C\text{-}P}$ = 1.9 Hz, Ar), 151.6 (d, $J_{C\text{-}P}$ = 10.1 Hz, Ar), 140.6 (d, $J_{C\text{-}P}$ = 6.4 Hz, Ar), 132.0 (d, $J_{C\text{-}P}$ = 8.3 Hz, Ar), 131.8 (d, $J_{C\text{-}P}$ = 2.7 Hz, Ar), 130.4 (d, $J_{C\text{-}P}$ = 91.8 Hz, Ar), 128.4 (d, $J_{C\text{-}P}$ = 11.1 Hz, Ar), 124.3 (d, $J_{C\text{-}P}$ = 88.4 Hz, Ar), 123.2 (d, $J_{C\text{-}P}$ = 8.3 Hz, Ar), 34.1 (d, $J_{C\text{-}P}$ = 71.7 Hz, $C(CH_3)_3$), 24.9 (s, $C(CH_3)_3$), 24.6 (d, $J_{C\text{-}P}$ = 1.5 Hz, CH_3). ^{31}P NMR (162 MHz, $CDCl_3$): δ 37.7 (s). HRMS (ESI): m/z: $[M+H]^+$ calculated for $C_{16}H_{21}NOP$: 274.1355, found: 274.1354. HPLC analysis of the product: Daicel Chiralpak AD-H column; *n*-hexane/*i*-PrOH = 85/15, flow rate = 1 mL/min, UV = 254 nm, t_{R1} = 17.125 min (minor) and t_{R2} = 17.919 min (major), ee = 99%. $[\alpha]_D^{20}$ = +20.0 (c = 1.0 in $CHCl_3$).

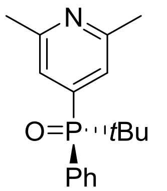

(*R*)-*tert*-butyl(2,6-dimethylpyridin-4-yl)(phenyl)phosphine oxide (18): Performed according to the general procedure to afford 72 mg (50%) of **18** as white solid. ^{1}H NMR (400 MHz, $CDCl_3$): δ 7.99–7.90 (m, 2 H, Ar), 7.57–7.45 (m, 5 H, Ar), 2.59 (s, 6 H, CH_3), 1.26 (d, J = 15.2 Hz, 9 H, $C(CH_3)_3$). ^{13}C NMR (101 MHz, $CDCl_3$): δ 157.9 (d, $J_{C\text{-}P}$ = 9.2 Hz, Ar), 141.0 (d, $J_{C\text{-}P}$ = 82.8 Hz, Ar), 131.9 (t, $J_{C\text{-}P}$ = 6.3 Hz, Ar), 129.9 (d, $J_{C\text{-}P}$ = 91.3 Hz, Ar), 128.4 (d, $J_{C\text{-}P}$ = 11.0 Hz, Ar), 122.4 (d, $J_{C\text{-}P}$ = 6.6 Hz, Ar), 33.9 (d, $J_{C\text{-}P}$ = 70.3 Hz, $C(CH_3)_3$), 25.0 (s, $C(CH_3)_3$), 24.6 (s, CH_3). ^{31}P NMR (162 MHz, $CDCl_3$): δ 37.3 (s). HRMS (ESI): m/z: $[M+H]^+$ calculated for $C_{17}H_{23}NOP$: 288.1512, found: 288.1510. HPLC analysis of the product: Daicel Chiralpak AD-H column; *n*-hexane/*i*-PrOH = 85/15, flow rate = 1 mL/min, UV = 254 nm, t_{R1} = 5.639 min (major) and t_{R2} = 7.379 min (minor), ee = 99%. $[\alpha]_D^{20}$ = +13.0 (c = 1.0 in $CHCl_3$).

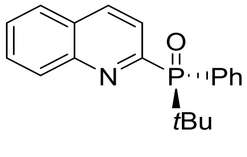

(*R*)-*tert*-butyl(phenyl)(quinolin-2-yl)phosphine oxide (19): Performed according to the general procedure to afford 113 mg (73%) of **19** as yellow solid. ^{1}H NMR (400 MHz, $CDCl_3$): δ 8.36–8.21 (m, 5 H, Ar), 7.86–7.74 (m, 2 H, Ar), 7.60 (t, J = 7.5 Hz, 1 H, Ar), 7.50–7.40 (m, 3 H, Ar), 1.30 (d, J = 15.2 Hz, 9 H, $C(CH_3)_3$). ^{13}C NMR (101 MHz, $CDCl_3$): δ 157.5 (d, $J_{C\text{-}P}$ = 116.4 Hz, Ar), 147.7 (d, $J_{C\text{-}P}$ = 19.9 Hz, Ar), 135.8 (d, $J_{C\text{-}P}$ = 8.3 Hz, Ar), 132.9 (d, $J_{C\text{-}P}$ = 7.6 Hz, Ar),

131.5 (d, J_{C-P} = 2.4 Hz, Ar), 130.2 (s, Ar), 130.0 (s, Ar), 129.9 (d, J_{C-P} = 88.9 Hz, Ar), 128.0 (s, Ar), 127.9 (s, Ar), 127.8 (s, Ar), 124.26 (s, Ar), 124.1 (s, Ar), 34.4 (d, J_{C-P} = 69.1 Hz, $C(CH_3)_3$), 24.7 (s, $C(CH_3)_3$). ^{31}P NMR (162 MHz, $CDCl_3$): δ 33.0 (s). HRMS (ESI): m/z: $[M+H]^+$ calculated for $C_{19}H_{21}NOP$: 310.1355, found: 310.1354. HPLC analysis of the product: Daicel Chiralpak AD-H column; *n*-hexane/*i*-PrOH = 80/20, flow rate = 1 mL/min, UV = 254 nm, t_{R1} = 6.879 min (major) and t_{R2} = 22.645 min (minor), ee = 98%. $[\alpha]_D^{20}$ = +208.0 (c = 1.0 in $CHCl_3$).

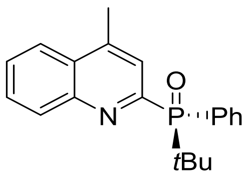

(*R*)-*tert*-butyl(4-methylquinolin-2-yl)(phenyl)phosphine oxide (20): Performed according to the general procedure to afford 119 mg (74%) of **20** as white solid. ^{1}H NMR (400 MHz, $CDCl_3$): δ 8.24–8.16 (m, 3 H, Ar), 8.06 (d, *J* = 4.0 Hz, 1 H, Ar), 7.97 (d, *J* = 8.4 Hz, 1 H, Ar), 7.71 (t, *J* = 7.4 Hz, 1 H, Ar), 7.57 (t, *J* = 7.6 Hz, 1 H, Ar), 7.42–7.33 (m, 3 H, Ar), 2.66 (s, 3 H, CH_3), 1.23 (d, *J* = 15.1 Hz, 9 H, $C(CH_3)_3$). ^{13}C NMR (101 MHz, $CDCl_3$): δ 157.2 (d, J_{C-P} = 115.7 Hz, Ar), 147.5 (d, J_{C-P} = 20.2 Hz, Ar), 144.4 (d, J_{C-P} = 8.4 Hz, Ar), 132.9 (d, J_{C-P} = 7.6 Hz, Ar), 131.4 (d, J_{C-P} = 2.4 Hz, Ar), 130.9 (s, Ar), 130.2 (d, J_{C-P} = 88.3 Hz, Ar), 129.5 (s, Ar), 128.1 (d, J_{C-P} = 2.4 Hz, Ar), 127.9 (s, Ar), 127.7 (d, J_{C-P} = 2.9 Hz, Ar), 124.8 (d, J_{C-P} = 18.4 Hz, Ar), 124.0 (s, Ar), 34.3 (d, J_{C-P} = 69.0 Hz, $C(CH_3)_3$), 24.8 (s, $C(CH_3)_3$), 18.7 (s, CH_3). ^{31}P NMR (162 MHz, $CDCl_3$): δ 33.2 (s). HRMS (ESI): m/z: $[M+H]^+$ calculated for $C_{20}H_{23}NOP$: 324.1512, found: 324.1510. HPLC analysis of the product: Daicel Chiralpak AD-H column; *n*-hexane/*i*-PrOH = 85/15, flow rate = 1 mL/min, UV = 254 nm, t_{R1} = 5.285 min (major) and t_{R2} = 9.705 min (minor), ee = 99%. $[\alpha]_D^{20}$ = +100.0 (c = 1.0 in $CHCl_3$).

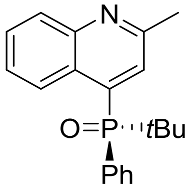

(*R*)-*tert*-butyl(2-methylquinolin-4-yl)(phenyl)phosphine oxide (21): Performed according to the general procedure to afford 113 mg (70%) of **21** as yellow solid. ^{1}H NMR (400 MHz, $CDCl_3$): δ 8.81 (d, *J* = 8.5 Hz, 1 H, Ar), 8.03 (d, *J* = 8.4 Hz, 1 H, Ar), 7.92–7.85 (m, 2 H, Ar), 7.66 (t, *J* = 12.1 Hz, 2 H, Ar), 7.55 (d, *J* = 6.6 Hz, 1 H, Ar), 7.51–7.42 (m, 3 H, Ar), 2.77 (s, 3 H, CH_3), 1.40 (d, *J* = 15.1 Hz, 9 H, $C(CH_3)_3$). ^{13}C NMR (101 MHz, $CDCl_3$): δ 156.8 (d, J_{C-P} = 10.5 Hz, Ar), 148.6 (d, J_{C-P} = 7.0 Hz, Ar), 137.2 (d, J_{C-P} = 80.2 Hz, Ar), 132.1 (d, J_{C-P} = 8.4 Hz, Ar), 131.9 (d, J_{C-P} = 2.4 Hz, Ar), 131.0 (s, Ar), 129.7 (s, Ar), 129.3 (s, Ar), 128.5 (d, J_{C-P} = 11.1 Hz, Ar), 127.8 (d, J_{C-P} = 3.4 Hz, Ar), 126.8 (d, J_{C-P} = 5.3 Hz, Ar), 126.5 (s, Ar), 126.3 (d, J_{C-P} = 8.3 Hz, Ar), 34.8 (d, J_{C-P} = 69.6 Hz, $C(CH_3)_3$), 26.0 (s, $C(CH_3)_3$), 25.5 (s, CH_3). ^{31}P NMR (162 MHz, $CDCl_3$): δ 42.9 (s). HRMS (ESI): m/z: $[M+H]^+$ calculated for $C_{20}H_{23}NOP$: 324.1512, found: 324.1510. HPLC analysis of the product: Daicel Chiralpak AD-H column; *n*-hexane/*i*-PrOH = 85/15, flow rate = 1 mL/min, UV = 254 nm, t_{R1} = 8.792 min (minor) and t_{R2} = 10.739min (major), ee = 98%. $[\alpha]_D^{20}$ = -84.0 (c = 1.0 in $CHCl_3$).

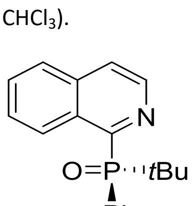

(*R*)-*tert*-butyl(isoquinolin-1-yl)(phenyl)phosphine oxide (22): Performed according to the general procedure to afford 119 mg (77%) of **22** as yellow solid. ^{1}H NMR (400 MHz, $CDCl_3$): δ 9.44 (d, *J* = 8.6 Hz, 1 H, Ar), 8.70 (d, *J* = 5.4 Hz, 1 H, Ar), 8.14–8.04 (m, 2 H, Ar), 7.81 (d, *J* = 8.1 Hz, 1 H, Ar), 7.74 (d, *J* = 5.2 Hz, 1 H, Ar), 7.65 (t, *J* = 7.5 Hz, 1 H,

Ar), 7.59–7.53 (m, 1 H, Ar), 7.50–7.40 (m, 3 H, Ar), 1.36 (d, *J* = 15.0 Hz, 9 H, C(C*H*$_3$)$_3$). ^{13}C NMR (101 MHz, $CDCl_3$): δ 156.3 (d, J_{C-P} = 117.0 Hz, Ar), 140.8 (s, Ar), 140.6 (s, Ar), 136.1 (d, J_{C-P} = 6.7 Hz, Ar), 133.0 (d, J_{C-P} = 7.8 Hz, Ar), 132.1 (d, J_{C-P} = 19.2 Hz, Ar), 131.3 (d, J_{C-P} = 2.4 Hz, Ar), 131.2 (d, J_{C-P} = 87.6 Hz, Ar), 130.3 (s, Ar), 128.0 (s, Ar), 127.8 (s, Ar), 127.7 (s, Ar), 127.5 (s, Ar), 127.1 (s, Ar), 122.9 (d, J_{C-P} = 2.9 Hz, Ar), 35.4 (d, J_{C-P} = 70.5 Hz, *C*(CH$_3$)$_3$), 25.1 (s, C(*C*H$_3$)$_3$). ^{31}P NMR (162 MHz, $CDCl_3$): δ 38.7 (s). HRMS (ESI): m/z: $[M+H]^+$ calculated for $C_{19}H_{21}NOP$: 310.1355, found: 310.1353. HPLC analysis of the product: Daicel Chiralpak AD-H column; *n*-hexane/*i*-PrOH = 95/05, flow rate = 1 mL/min, UV = 254 nm, t_{R1} = 26.065 min (minor) and t_{R2} = 28.952 min (major), ee = 97%. $[\alpha]_D^{20}$ = -28.0 (c = 1.0 in $CHCl_3$).

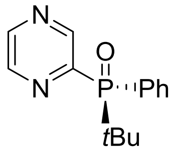

(*R*)-*tert*-butyl(phenyl)(pyrazin-2-yl)phosphine oxide (23): Performed according to the general procedure to afford 114 mg (88%) of **23** as yellow solid. ^{1}H NMR (400 MHz, $CDCl_3$): δ 9.37 (s, 1 H, Ar), 8.78 (s, 1 H, Ar), 8.70 (t, *J* = 2.7 Hz, 1 H, Ar), 8.19–8.12 (m, 2 H, Ar), 7.55–7.45 (m, 3 H, Ar), 1.24 (d, *J* = 15.5 Hz, 9 H, C(C*H*$_3$)$_3$). ^{13}C NMR (101 MHz, $CDCl_3$): δ 149.6 (d, J_{C-P} = 17.4 Hz, Ar), 146.0 (s, Ar), 144.0 (d, J_{C-P} = 13.3 Hz, Ar), 132.6 (d, J_{C-P} = 7.7 Hz, Ar), 131.9 (s, Ar), 128.1 (d, J_{C-P} = 11.1 Hz, Ar), 34.1 (d, J_{C-P} = 69.8 Hz, *C*(CH$_3$)$_3$), 24.4 (s, C(*C*H$_3$)$_3$). ^{31}P NMR (162 MHz, $CDCl_3$): δ 32.5 (s). HRMS (ESI): m/z: $[M+H]^+$ calculated for $C_{14}H_{18}N_2OP$: 261.1151, found: 261.1148. HPLC analysis of the product: Daicel Chiralpak AD-H column; *n*-hexane/*i*-PrOH = 85/15, flow rate = 1 mL/min, UV = 254 nm, t_{R1} = 9.185 min (minor) and t_{R2} = 16.112 min (major), ee = 97%. $[\alpha]_D^{20}$ = +173.0 (c = 1.0 in $CHCl_3$).

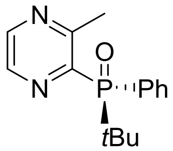

(*R*)-*tert*-butyl(3-methylpyrazin-2-yl)(phenyl)phosphine oxide (24): Performed according to the general procedure to afford 115 mg (84%) of **24** as yellow solid. ^{1}H NMR (400 MHz, $CDCl_3$): δ 8.57–8.51 (m, 2 H, Ar), 7.85 (t, *J* = 8.6 Hz, 2 H, Ar), 7.50 (d, *J* = 6.9 Hz, 1 H, Ar), 7.43 (t, *J* = 7.3 Hz, 2 H, Ar), 2.72 (s, 3 H, C*H*$_3$), 1.34–1.27 (m, 9 H, C(C*H*$_3$)$_3$). ^{13}C NMR (101 MHz, $CDCl_3$): δ 159.9 (d, J_{C-P} = 17.6 Hz, Ar), 150.8 (d, J_{C-P} = 112.9 Hz, Ar), 144.6 (d, J_{C-P} = 3.0 Hz, Ar), 140.2 (d, J_{C-P} = 14.1 Hz, Ar), 132.5 (d, J_{C-P} = 8.1 Hz, Ar), 131.7 (d, J_{C-P} = 2.7 Hz, Ar), 130.2 (d, J_{C-P} = 88.8 Hz, Ar), 128.0 (d, J_{C-P} = 11.0 Hz, Ar), 35.0 (d, J_{C-P} = 71.1 Hz, *C*(CH$_3$)$_3$), 24.8 (s, C(*C*H$_3$)$_3$), 22.8 (s, *C*H$_3$). ^{31}P NMR (162 MHz, $CDCl_3$): δ 33.1 (s). HRMS (ESI): m/z: $[M+H]^+$ calculated for $C_{15}H_{20}N_2OP$: 275.1308, found: 275.1304. HPLC analysis of the product: Daicel Chiralpak AD-H column; *n*-hexane/*i*-PrOH = 90/10, flow rate = 1 mL/min, UV = 254 nm, t_{R1} = 7.519min (major) and t_{R2} = 8.092 min (minor), ee = 97%. $[\alpha]_D^{20}$ = +21.0 (c = 1.0 in $CHCl_3$).

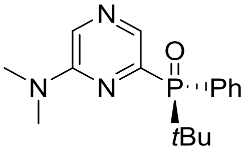

(*R*)-*tert*-butyl(6-(dimethylamino)pyrazin-2-yl)(phenyl)phosphine oxide (25): Performed according to the general procedure to afford 121 mg (80%) of **25** as yellow solid. ^{1}H NMR (400 MHz, $CDCl_3$): δ 8.54 (s, 1 H, Ar), 8.19–8.13 (m, 3 H, Ar), 7.53–7.43 (m, 3 H, Ar), 3.22 (s, 6 H, N(C*H*$_3$)$_2$), 1.25 (d, *J* = 15.1 Hz, 9 H, C(C*H*$_3$)$_3$). ^{13}C NMR (101 MHz, $CDCl_3$): δ 153.2 (d, J_{C-P} = 15.0 Hz, Ar), 147.6 (d, J_{C-P} = 114.2 Hz, Ar), 136.2 (d, J_{C-P} = 18.4 Hz, Ar), 132.4 (d, J_{C-P} = 7.6 Hz, Ar), 131.9 (d, J_{C-P} = 2.6 Hz, Ar), 131.4 (s, Ar), 129.82 (d, J_{C-P} = 89.8 Hz, Ar), 127.7 (d, J_{C-P} = 10.9 Hz, Ar), 37.8 (s, N(*C*H$_3$)$_2$), 33.8 (d, J_{C-P} = 69.6 Hz, *C*(CH$_3$)$_3$), 24.5 (s, C(*C*H$_3$)$_3$). ^{31}P NMR (162 MHz, $CDCl_3$): δ 32.5 (s). HRMS (ESI): m/z: $[M+H]^+$ calculated for $C_{16}H_{23}N_3OP$: 304.1573, found: 304.1571. HPLC analysis of the product: Daicel Chiralpak AD-H column;

n-hexane/i-PrOH = 85/15, flow rate = 1 mL/min, UV = 254 nm, t_{R1} = 11.279 min (major) and t_{R2} = 13.519 min (minor), ee = 99%. $[\alpha]_D^{20}$ = +229.0 (c = 1.0 in $CHCl_3$).

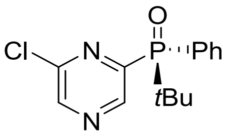

(*R*)-*tert*-butyl(6-chloropyrazin-2-yl)(phenyl)phosphine oxide (26): Performed according to the general procedure to afford 73 mg (50%) of **26** as white solid. ^{1}H NMR (400 MHz, $CDCl_3$): δ 9.26 (s, 1 H, Ar), 8.71 (d, J = 2.5 Hz, 1 H, Ar), 8.15–8.09 (m, 2 H, Ar), 7.56–7.48 (m, 3 H, Ar), 1.25 (d, J = 15.7 Hz, 9 H, C(CH_3)$_3$). ^{13}C NMR (101 MHz, $CDCl_3$): δ 152.0 (d, J_{C-P} = 105.0 Hz, Ar), 148.8 (d, J_{C-P} = 13.5 Hz, Ar), 147.2 (d, J_{C-P} = 16.1 Hz, Ar), 146.3 (d, J_{C-P} = 2.6 Hz, Ar), 132.5 (d, J_{C-P} = 7.9 Hz, Ar), 132.1 (d, J_{C-P} = 2.8 Hz, Ar), 128.4 (d, J_{C-P} = 91.7 Hz, Ar), 128.3 (d, J_{C-P} = 11.3 Hz, Ar), 34.3 (d, J_{C-P} = 70.0 Hz, C(CH_3)$_3$), 24.4 (s, C(CH_3)$_3$). ^{31}P NMR (162 MHz, $CDCl_3$): δ 32.2 (s). HRMS (ESI): m/z: $[M+H]^+$ calculated for $C_{14}H_{17}ClN_2OP$: 295.0762, found: 295.0760. HPLC analysis of the product: Daicel Chiralpak AD-H column; n-hexane/i-PrOH = 98/02, flow rate = 1 mL/min, UV = 254 nm, t_{R1} = 38.425 min (minor) and t_{R2} = 42.452 min (major), ee = 97%. $[\alpha]_D^{20}$ = +193.0 (c = 1.0 in $CHCl_3$).

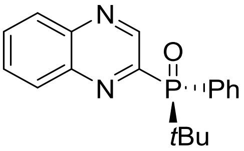

(*R*)-*tert*-butyl(phenyl)(quinoxalin-2-yl)phosphine oxide (27): Performed according to the general procedure to afford 144 mg (93%) of **27** as yellow solid. ^{1}H NMR (400 MHz, $CDCl_3$): δ 9.60 (s, 1 H, Ar), 8.29–8.21 (m, 3 H, Ar), 8.10–8.15 (m, 1 H, Ar), 7.90–7.84 (m, 2 H, Ar), 7.55–7.46 (m, 3 H, Ar), 1.32 (d, J = 15.5 Hz, 9 H, C(CH_3)$_3$). ^{13}C NMR (101 MHz, $CDCl_3$): δ 152.6 (d, J_{C-P} = 109.1 Hz, Ar), 147.3 (d, J_{C-P} = 18.8 Hz, Ar), 142.5 (d, J_{C-P} = 2.3 Hz, Ar), 141.7 (d, J_{C-P} = 15.6 Hz, Ar), 132.7 (d, J_{C-P} = 7.8 Hz, Ar), 131.9 (d, J_{C-P} = 2.8 Hz, Ar), 131.8 (s, Ar), 130.68 (s, Ar), 130.1 (d, J_{C-P} = 0.8 Hz, Ar), 129.7 (d, J_{C-P} = 1.6 Hz, Ar), 129.6 (s, Ar), 128.7 (s, Ar), 128.2 (s, Ar), 128.0 (s, Ar), 34.6 (d, J_{C-P} = 69.3 Hz, C(CH_3)$_3$), 24.5 (s, C(CH_3)$_3$). ^{31}P NMR (162 MHz, $CDCl_3$): δ 33.2 (s). HRMS (ESI): m/z: $[M+H]^+$ calculated for $C_{18}H_{20}N_2OP$: 311.1308, found: 311.1307. HPLC analysis of the product: Daicel Chiralpak AD-H column; n-hexane/i-PrOH = 85/15, flow rate = 1 mL/min, UV = 254 nm, t_{R1} = 13.772 min (major) and t_{R2} = 21.719 min (minor), ee = 98%. $[\alpha]_D^{20}$ = +153.0 (c = 1.0 in $CHCl_3$).

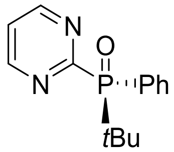

(*R*)-*tert*-butyl(phenyl)(pyrimidin-2-yl)phosphine oxide (28): Performed according to the general procedure to afford 107 mg (83%) of **28** as white solid. ^{1}H NMR (400 MHz, $CDCl_3$): δ 8.94 (d, J = 4.7 Hz, 2 H, Ar), 8.10 (t, J = 8.8 Hz, 2 H, Ar), 7.52 (d, J = 7.3 Hz, 1 H, Ar), 7.48–7.39 (m, 3 H, Ar), 1.30 (d, J = 15.3 Hz, 9 H, C(CH_3)$_3$). ^{13}C NMR (101 MHz, $CDCl_3$): δ 156.6 (d, J_{C-P} = 12.6 Hz, Ar), 132.9 (d, J_{C-P} = 8.0 Hz, Ar), 131.73 (s, Ar), 127.9 (d, J_{C-P} = 11.2 Hz, Ar), 121.9 (s, Ar), 34.1 (d, J_{C-P} = 69.5 Hz, C(CH_3)$_3$), 24.8 (s, C(CH_3)$_3$). ^{31}P NMR (162 MHz, $CDCl_3$): δ 35.0 (s). HRMS (ESI): m/z: $[M+H]^+$ calculated for $C_{14}H_{18}N_2OP$: 261.1151, found: 261.1149. HPLC analysis of the product: Daicel Chiralpak AD-H column; n-hexane/i-PrOH = 85/15, flow rate = 1 mL/min, UV = 254 nm, t_{R1} = 11.839 min (minor) and t_{R2} = 12.679 min (major), ee = 98%. $[\alpha]_D^{20}$ = +173.0 (c = 1.0 in $CHCl_3$).

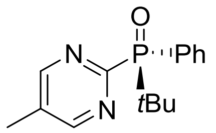

(*R*)-*tert*-butyl(5-methylpyrimidin-2-yl)(phenyl)phosphine oxide (29): Performed according to the general procedure to afford 122 mg (89%) of **29** as white solid. ^{1}H NMR (400 MHz, $CDCl_3$): δ 8.75 (s, 2 H, Ar), 8.09 (t, *J* = 8.6 Hz, 2 H, Ar), 7.53–7.41 (m, 3 H, Ar), 2.38 (s, 3 H, C*H*$_3$), 1.29 (d, *J* = 15.2 Hz, 9 H, C(C*H*$_3$)$_3$). ^{13}C NMR (101 MHz, $CDCl_3$): δ 156.8 (d, $J_{C\text{-}P}$ = 13.1 Hz, Ar), 132.9 (d, $J_{C\text{-}P}$ = 8.1 Hz, Ar), 131.8 (d, $J_{C\text{-}P}$ = 2.4 Hz, Ar), 131.6 (d, $J_{C\text{-}P}$ = 2.2 Hz, Ar), 127.8 (d, $J_{C\text{-}P}$ = 11.1 Hz, Ar), 34.0 (d, $J_{C\text{-}P}$ = 69.9 Hz, *C*(CH$_3$)$_3$), 24.8 (s, C(*C*H$_3$)$_3$), 15.9 (s, *C*H$_3$). ^{31}P NMR (162 MHz, $CDCl_3$): δ 34.8 (s). HRMS (ESI): m/z: $[M+H]^+$ calculated for $C_{15}H_{20}N_2OP$: 275.1308, found: 275.1304. HPLC analysis of the product: Daicel Chiralpak AD-H column; *n*-hexane/*i*-PrOH = 85/15, flow rate = 1 mL/min, UV = 254 nm, t_{R1} = 12.525 min (major) and t_{R2} = 22.972 min (minor), ee = 99%. $[\alpha]_D^{20}$ = +141.0 (c = 1.0 in $CHCl_3$).

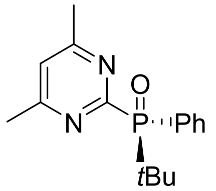

(*R*)-*tert*-butyl(4,6-dimethylpyrimidin-2-yl)(phenyl)phosphine oxide (30): Performed according to the general procedure to afford 119 mg (83%) of **30** as white solid. ^{1}H NMR (400 MHz, $CDCl_3$): δ 8.12–8.05 (m, 2 H, Ar), 7.52–7.47 (m, 1 H, Ar), 7.46–7.40 (m, 2 H, Ar), 7.09 (d, *J* = 2.6 Hz, 1 H, Ar), 2.56 (s, 6 H, C*H*$_3$), 1.30 (d, *J* = 15.1 Hz, 9 H, C(C*H*$_3$)$_3$). ^{13}C NMR (101 MHz, $CDCl_3$): δ 166.5 (d, $J_{C\text{-}P}$ = 13.4 Hz, Ar), 166.3 (d, $J_{C\text{-}P}$ = 144.0 Hz, Ar), 133.0 (d, $J_{C\text{-}P}$ = 8.0 Hz, Ar), 131.5 (d, $J_{C\text{-}P}$ = 2.7 Hz, Ar), 129.4 (d, $J_{C\text{-}P}$ = 90.0 Hz, Ar), 127.7 (d, $J_{C\text{-}P}$ = 11.1 Hz, Ar), 121.0 (d, $J_{C\text{-}P}$ = 2.5 Hz, Ar), 34.0 (d, $J_{C\text{-}P}$ = 69.5 Hz, *C*(CH$_3$)$_3$), 24.9 (s, C(*C*H$_3$)$_3$), 24.0 (s, *C*H$_3$). ^{31}P NMR (162 MHz, $CDCl_3$): δ 34.3 (s). HRMS (ESI): m/z: $[M+H]^+$ calculated for $C_{16}H_{22}N_2OP$: 289.1464, found: 289.1462. HPLC analysis of the product: Daicel Chiralpak AS-H column; *n*-hexane/*i*-PrOH = 90/10, flow rate = 1 mL/min, UV = 254 nm, t_{R1} = 7.879 min (major) and t_{R2} = 11.565 min (minor), ee = 99%. $[\alpha]_D^{20}$ = +89.0 (c = 1.0 in $CHCl_3$).

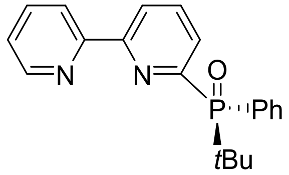

(*R*)-[2,2'-bipyridin]-6-yl(*tert*-butyl)(phenyl)phosphine oxide (31): Performed according to the general procedure to afford 147 mg (87%) of **31** as white solid. ^{1}H NMR (400 MHz, $CDCl_3$): δ 8.73 (d, *J* = 4.3 Hz, 1 H, Ar), 8.58 (d, *J* = 8.1 Hz, 1 H, Ar), 8.45 (d, *J* = 7.9 Hz, 1 H, Ar), 8.30–8.21 (m, 3 H, Ar), 7.99–7.90 (m, 2 H, Ar), 7.52–7.43 (m, 3 H, Ar), 7.41–7.36 (m, 1 H, Ar), 1.31 (d, *J* = 15.1 Hz, 9 H, C(C*H*$_3$)$_3$). ^{13}C NMR (101 MHz, $CDCl_3$): δ 156.2 (d, $J_{C\text{-}P}$ = 62.5 Hz, Ar), 155.6 (s, Ar), 155.6 (d, $J_{C\text{-}P}$ = 37.8 Hz, Ar), 149.4 (s, Ar), 137.2 (t, $J_{C\text{-}P}$ = 4.3 Hz, Ar), 132.7 (d, $J_{C\text{-}P}$ = 7.6 Hz, Ar), 131.5 (d, $J_{C\text{-}P}$ = 2.6 Hz, Ar), 130.1 (d, $J_{C\text{-}P}$ = 89.6 Hz, Ar), 129.3 (s, Ar), 129.2 (s, Ar), 127.9 (d, $J_{C\text{-}P}$ = 10.9 Hz, Ar), 124.2 (s, Ar), 122.6 (d, $J_{C\text{-}P}$ = 2.9 Hz, Ar), 120.9 (s, Ar), 33.9 (d, $J_{C\text{-}P}$ = 69.6 Hz, *C*(CH$_3$)$_3$), 24.8 (s, C(*C*H$_3$)$_3$). ^{31}P NMR (162 MHz, $CDCl_3$): δ 33.0 (s). HRMS (ESI): m/z: $[M+H]^+$ calculated for $C_{20}H_{22}N_2OP$: 337.1464, found: 337.1462. HPLC analysis of the product: Daicel Chiralpak AD-H column; *n*-hexane/*i*-PrOH = 80/20, flow rate = 1 mL/min, UV = 254 nm, t_{R1} = 6.365 min (major) and t_{R2} = 6.739 min (minor), ee = 99%. $[\alpha]_D^{20}$ = +117.0 (c = 1.0 in $CHCl_3$).

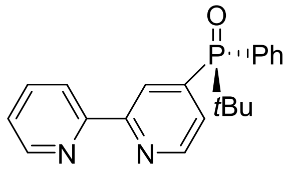

(*R*)-[2,2'-bipyridin]-4-yl(*tert*-butyl)(phenyl)phosphine oxide (32): Performed according to the general procedure to afford 151 mg (90%) of **32** as white solid. ^{1}H NMR (400 MHz, $CDCl_3$): δ 8.97 (d, *J* = 10.1 Hz, 1 H, Ar), 8.83 (t, *J* = 4.1 Hz, 1 H, Ar), 8.70 (d, *J* = 4.1 Hz, 1 H, Ar), 8.42 (d, *J* = 8.0 Hz, 1 H, Ar), 8.05–7.98 (m, 2 H, Ar), 7.96–7.90 (m, 1 H, Ar), 7.86–7.79 (m, 1 H, Ar), 7.58–7.49 (m, 3 H, Ar), 7.35–7.30 (m, 1 H, Ar), 1.31 (d, *J* = 15.3 Hz, 9 H, $C(CH_3)_3$). ^{13}C NMR (101 MHz, $CDCl_3$): δ 155.9 (d, $J_{C\text{-}P}$ = 8.9 Hz, Ar), 155.3 (d, $J_{C\text{-}P}$ = 1.4 Hz, Ar), 149.4 (s, Ar), 149.32 (s, Ar), 141.8 (d, $J_{C\text{-}P}$ = 82.8 Hz, Ar), 137.0 (s, Ar), 132.2 (d, $J_{C\text{-}P}$ = 8.2 Hz, Ar), 132.0 (d, $J_{C\text{-}P}$ = 2.7 Hz, Ar), 129.8 (d, $J_{C\text{-}P}$ = 91.5 Hz, Ar), 128.6 (d, $J_{C\text{-}P}$ = 11.1 Hz, Ar), 126.5 (d, $J_{C\text{-}P}$ = 6.0 Hz, Ar), 124.1 (s, Ar), 122.9 (d, $J_{C\text{-}P}$ = 8.0 Hz, Ar), 121.2 (s, Ar), 34.1 (d, $J_{C\text{-}P}$ = 70.5 Hz, $C(CH_3)_3$), 25.1 (s, $C(CH_3)_3$). ^{31}P NMR (162 MHz, $CDCl_3$): δ 37.3 (s). HRMS (ESI): m/z: $[M+H]^+$ calculated for $C_{20}H_{22}N_2OP$: 337.1464, found: 337.1461. HPLC analysis of the product: Daicel Chiralpak AD-H column; *n*-hexane/*i*-PrOH = 85/15, flow rate = 1 mL/min, UV = 254 nm, t_{R1} = 9.899 min (minor) and t_{R2} = 11.185 min (major), ee = 99%. $[\alpha]_D^{20}$ = +114.0 (c = 1.0 in $CHCl_3$).

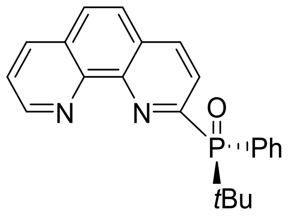

(*R*)-*tert*-butyl(1,10-phenanthrolin-2-yl)(phenyl)phosphine oxide (33): Performed according to the general procedure to afford 160 mg (89%) of **33** as white solid. ^{1}H NMR (400 MHz, $CDCl_3$): δ 9.28–9.20 (m, 1 H, Ar), 8.63–8.56 (m, 2 H, Ar), 8.54–8.49 (m, 1 H, Ar), 8.36–8.30 (m, 1 H, Ar), 8.25–8.20 (m, 1 H, Ar), 7.84–7.75 (m, 2 H, Ar), 7.68–7.62 (m, 1 H, Ar), 7.48 (d, *J* = 2.2 Hz, 3 H, Ar), 1.37 (d, *J* = 15.2 Hz, 9 H, $C(CH_3)_3$). ^{13}C NMR (101 MHz, $CDCl_3$): δ 157.1 (d, $J_{C\text{-}P}$ = 118.2 Hz, Ar), 150.9 (s, Ar), 146.7 (s, Ar), 146.0 (d, $J_{C\text{-}P}$ = 18.5 Hz, Ar), 135.8 (s, Ar), 135.6 (d, $J_{C\text{-}P}$ = 8.3 Hz, Ar) 133.4 (d, $J_{C\text{-}P}$ = 7.6 Hz, Ar), 131.3 (d, $J_{C\text{-}P}$ = 2.7 Hz, Ar), 130.0 (d, $J_{C\text{-}P}$ = 89.4 Hz, Ar), 129.0 (s, Ar), 128.8 (d, $J_{C\text{-}P}$ = 2.8 Hz, Ar), 128.3 (s, Ar), 127.9 (d, $J_{C\text{-}P}$ = 10.9 Hz, Ar), 126.5 (d, $J_{C\text{-}P}$ = 18.6 Hz, Ar), 126.3 (s, Ar), 123.3 (s, Ar), 34.1 (d, $J_{C\text{-}P}$ = 69.1 Hz, $C(CH_3)_3$), 24.8 (s, $C(CH_3)_3$). ^{31}P NMR (162 MHz, $CDCl_3$): δ 33.6 (s). HRMS (ESI): m/z: $[M+H]^+$ calculated for $C_{22}H_{22}N_2OP$: 361.1464, found: 361.1463. HPLC analysis of the product: Daicel Chiralpak AD-H column; *n*-hexane/*i*-PrOH = 85/15, flow rate = 1 mL/min, UV = 254 nm, t_{R1} = 13.272 min (minor) and t_{R2} = 14.252 min (major), ee = 98%. $[\alpha]_D^{20}$ = +216.0 (c = 1.0 in $CHCl_3$).

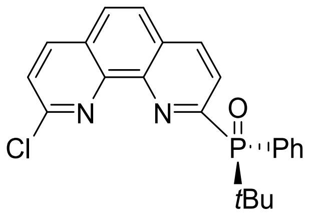

(*R*)-*tert*-butyl(9-chloro-1,10-phenanthrolin-2-yl)(phenyl)phosphine oxide (34): Performed according to the general procedure to afford 108 mg (55%) of **34** as white solid. ^{1}H NMR (400 MHz, $CDCl_3$): δ 8.68–8.58 (m, 2 H, Ar), 8.54–8.48 (m, 1 H, Ar), 8.39–8.32 (m, 1 H, Ar), 8.19 (d, *J* = 8.4 Hz, 1 H, Ar), 7.82 (s, 2 H, Ar), 7.66 (d, *J* = 8.4 Hz, 1 H, Ar), 7.55–7.49 (m, 3 H, Ar), 1.34 (d, *J* = 15.2 Hz, 9 H, $C(CH_3)_3$). ^{13}C NMR (101 MHz, $CDCl_3$): δ 157.6 (d, $J_{C\text{-}P}$ = 117.4 Hz, Ar), 151.7 (s, Ar), 146.4 (s, Ar), 144.7 (d, $J_{C\text{-}P}$ = 18.6 Hz, Ar), 138.5 (s, Ar), 135.6 (d, $J_{C\text{-}P}$ = 8.2 Hz, Ar), 133.7 (d, $J_{C\text{-}P}$ =

7.5 Hz, Ar), 131.5 (d, $J_{C\text{-}P}$ = 2.7 Hz, Ar), 129.7 (d, $J_{C\text{-}P}$ = 90.0 Hz, Ar), 129.0 (d, $J_{C\text{-}P}$ = 2.8 Hz, Ar), 128.0 (s, Ar), 127.6 (s, Ar), 127.6 (s, Ar), 127.3 (s, Ar), 126.9 (s, Ar), 126.7 (s, Ar), 124.5 (s, Ar), 34.1 (d, $J_{C\text{-}P}$ = 69.0 Hz, *C*(CH_3)$_3$), 24.7 (s, C(*C*H_3)$_3$). ^{31}P NMR (162 MHz, $CDCl_3$): δ 33.8 (s). HRMS (ESI): m/z: [M+H]+ calculated for C22H21ClN2OP: 395.1075, found: 395.1077. HPLC analysis of the product: Daicel Chiralpak AD-H column; *n*-hexane/*i*-PrOH = 85/15, flow rate = 1 mL/min, UV = 254 nm, t_{R1} = 13.172 min (minor) and t_{R2} = 25.485 min (major), ee = 97%. $[\alpha]_D^{20}$ = +206.0 (c = 1.0 in $CHCl_3$).

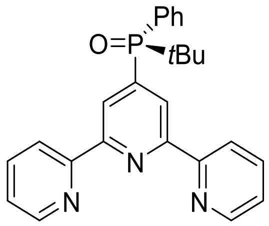

(*R*)-[2,2':6',2''-terpyridin]-4'-yl(*tert*-butyl)(phenyl)phosphine oxide (35): Performed according to the general procedure to afford 183 mg (89%) of **35** as white solid. ^{1}H NMR (400 MHz, $CDCl_3$): δ 9.06 (d, *J* = 10.3 Hz, 2 H, Ar), 8.74–8.67 (m, 2 H, Ar), 8.60 (d, *J* = 7.9 Hz, 2 H, Ar), 8.10–8.03 (m, 2 H, Ar), 7.88–7.81 (m, 2 H, Ar), 7.57–7.49 (m, 3 H, Ar), 7.36–7.31 (m, 2 H, Ar), 1.36 (d, *J* = 15.3 Hz, 9 H, C(C*H*$_3$)$_3$). ^{13}C NMR (101 MHz, $CDCl_3$): δ 155.6 (d, $J_{C\text{-}P}$ = 9.3 Hz, Ar), 155.4 (s, Ar), 149.4 (s, Ar), 142.9 (d, $J_{C\text{-}P}$ = 83.3 Hz, Ar), 136.8 (s, Ar), 132.3 (d, $J_{C\text{-}P}$ = 8.2 Hz, Ar), 131.9 (d, $J_{C\text{-}P}$ = 2.7 Hz, Ar), 130.0 (d, $J_{C\text{-}P}$ = 91.2 Hz, Ar), 128.5 (d, $J_{C\text{-}P}$ = 11.1 Hz, Ar), 124.1 (s, Ar), 123.4 (d, $J_{C\text{-}P}$ = 7.8 Hz, Ar), 121.3 (s, Ar), 34.2 (d, $J_{C\text{-}P}$ = 70.4 Hz, *C*(CH_3)$_3$), 25.2 (s, C(*C*H_3)$_3$). ^{31}P NMR (162 MHz, $CDCl_3$): δ 37.2 (s). HRMS (ESI): m/z: $[M+H]^+$ calculated for $C_{25}H_{25}N_3OP$: 414.1730, found: 414.1727. HPLC analysis of the product: Daicel Chiralpak AD-H column; *n*-hexane/*i*-PrOH = 85/15, flow rate = 1 mL/min, UV = 254 nm, t_{R1} = 9.919 min (minor) and t_{R2} = 11.079 min (major), ee = 99%. $[\alpha]_D^{20}$ = +84.0 (c = 1.0 in $CHCl_3$).

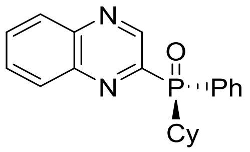

(*R*)-cyclohexyl(phenyl)(quinoxalin-2-yl)phosphine oxide (39): Performed according to the general procedure to afford 106 mg (60%) of **39** as white solid. ^{1}H NMR (400 MHz, $CDCl_3$): δ 9.50 (s, 1 H, Ar), 8.27 – 8.20 (m, 1 H, Ar), 8.19 – 8.12 (m, 1 H, Ar), 8.11 – 8.01 (m, 2 H, Ar), 7.90 – 7.83 (m, 2 H, Ar), 7.50 (t, *J* = 7.4 Hz, 3 H, Ar), 2.77 – 2.65 (m, 1 H, Cy), 1.83 – 1.64 (m, 7 H, Cy), 1.28 (d, *J* = 8.1 Hz, 3 H, Cy). ^{13}C NMR (101 MHz, $CDCl_3$): δ 151.5 (d, $J_{C\text{-}P}$ = 112.9 Hz, Ar), 145.5 (d, $J_{C\text{-}P}$ = 19.5 Hz, Ar), 141.6 (d, $J_{C\text{-}P}$ = 2.2 Hz, Ar), 141.2 (d, $J_{C\text{-}P}$ = 16.2 Hz, Ar), 130.9 (d, $J_{C\text{-}P}$ = 2.7 Hz, Ar), 130.6 (s, Ar), 130.5 (s, Ar), 130.4 (s, Ar), 129.7 (s, Ar), 129.6 (s, Ar), 129.0 (s, Ar), 128.8 (s, Ar), 128.7 (d, $J_{C\text{-}P}$ = 1.6 Hz, Ar), 127.5 (s, Ar), 127.4 (s, Ar), 36.2 (d, $J_{C\text{-}P}$ = 73.1 Hz, Cy), 25.2 (d, $J_{C\text{-}P}$ = 24.8 Hz, Cy), 25.1 (d, $J_{C\text{-}P}$ = 2.9 Hz, Cy), 24.7 (s, Cy), 23.6 (d, $J_{C\text{-}P}$ = 3.4 Hz, Cy), 22.9 (d, $J_{C\text{-}P}$ = 2.3 Hz, Cy). ^{31}P NMR (162 MHz, $CDCl_3$): δ 31.8 (s). HRMS (ESI): m/z: $[M+H]^+$ calculated for $C_{20}H_{22}N_2OP$: 337.1464, found: 337.1462. HPLC analysis of the product: Daicel Chiralpak AD-H column; *n*-hexane/*i*-PrOH = 85/15, flow rate = 1 mL/min, UV = 254 nm, t_{R1} = 12.539 min (major) and t_{R2} = 13.965 min (minor), ee = 98%. $[\alpha]_D^{20}$ = +73.0 (c = 1.0 in $CHCl_3$).

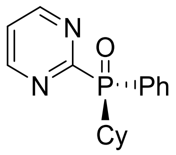

(*R*)-cyclohexyl(phenyl)(pyrimidin-2-yl)phosphine oxide (40): Performed according to the general procedure to afford 76 mg (50%) of **40** as white solid. ^{1}H NMR (400 MHz, $CDCl_3$): δ 8.90 (s, 2 H, Ar), 8.11 – 7.90 (m, 2 H, Ar), 7.54

– 7.43 (m, 3 H, Ar), 7.37 (s, 1 H, Ar), 2.64 (d, J = 14.5 Hz, 1 H, Cy), 1.70 (t, J = 39.3 Hz, 7 H, Cy), 1.27 (s, 3 H, Cy). ^{13}C NMR (101 MHz, $CDCl_3$): δ 168.8 (s, Ar), 167.3 (s, Ar), 157.0 (d, $J_{C\text{-}P}$ = 12.8 Hz, Ar), 131.8 (d, $J_{C\text{-}P}$ = 2.7 Hz, Ar), 131.7 (s, Ar), 131.6 (s, Ar), 130.4 (s, Ar), 129.4 (s, Ar), 128.5 (s, Ar), 128.4 (s, Ar), 121.8 (s, Ar), 36.9 (d, $J_{C\text{-}P}$ = 73.5 Hz, Cy), 26.3 (d, $J_{C\text{-}P}$ = 23.8 Hz, Cy), 26.3 (d, $J_{C\text{-}P}$ = 4.1 Hz, Cy), 25.8 (d, $J_{C\text{-}P}$ = 1.0 Hz, Cy), 25.8 (d, $J_{C\text{-}P}$ = 1.0 Hz, Cy), 24.7 (d, $J_{C\text{-}P}$ = 3.4 Hz, Cy), 24.1 (d, $J_{C\text{-}P}$ = 2.3 Hz, Cy). ^{31}P NMR (162 MHz, $CDCl_3$): δ 30.8 (s). HRMS (ESI): m/z: $[M+H]^+$ calculated for $C_{16}H_{20}N_2OP$: 287.1308, found: 287.1306. HPLC analysis of the product: Daicel Chiralpak AD-H column; *n*-hexane/*i*-PrOH = 85/15, flow rate = 1 mL/min, UV = 254 nm, t_{R1} = 13.992 min (major) and t_{R2} = 17.232 min (minor), ee = 97%. $[\alpha]_D^{20}$ = -4.0 (c = 1.0 in $CHCl_3$).

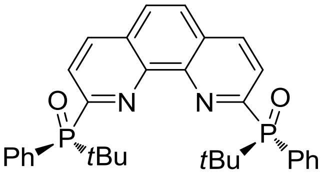

(*1R,1'R*)-(1,10-phenanthroline-2,9-diyl)bis(*tert*-butyl(phenyl)phosphine oxide (41): Performed according to the general procedure to afford 221 mg (82%) of **41** as white solid. ^{1}H NMR (400 MHz, $CDCl_3$): δ 8.79–8.70 (m, 2 H, Ar), 8.45 (t, J = 8.9 Hz, 6 H, Ar), 7.94 (s, 2 H, Ar), 7.48 (d, J = 7.0 Hz, 2 H, Ar), 7.42 (d, J = 5.4 Hz, 4 H, Ar), 1.46 (d, J = 15.3 Hz, 18 H, C(CH_3)$_3$). ^{13}C NMR (101 MHz, $CDCl_3$): δ 158.0 (d, $J_{C\text{-}P}$ = 115.1 Hz, Ar), 146.3 (d, $J_{C\text{-}P}$ = 18.3 Hz, Ar), 136.3 (d, $J_{C\text{-}P}$ = 8.3 Hz, Ar), 132.8 (d, $J_{C\text{-}P}$ = 7.8 Hz, Ar), 131.6 (d, $J_{C\text{-}P}$ = 2.6 Hz, Ar), 130.2 (s, Ar), 129.7 (d, $J_{C\text{-}P}$ = 2.5 Hz, Ar), 129.3 (s, Ar), 128.4 (s, Ar), 128.0 (s, Ar), 127.9 (s, Ar), 127.8 (s, Ar), 127.6 (s, Ar), 34.1 (d, $J_{C\text{-}P}$ = 69.2 Hz, *C*(CH_3)$_3$), 24.9 (s, C(*C*H_3)$_3$). ^{31}P NMR (162 MHz, $CDCl_3$): δ 33.5 (s). HRMS (ESI): m/z: $[M+H]^+$ calculated for $C_{32}H_{35}N_2O_2P_2$: 541.2168, found: 541.2171. HPLC analysis of the product: Daicel Chiralpak AD-H column; *n*-hexane/*i*-PrOH = 85/15, flow rate = 1 mL/min, UV = 254 nm, t_{R1} = 15.545 min (major) and t_{R2} = 18.132 min (minor), ee = 99%. $[\alpha]_D^{20}$ = +130.0 (c = 1.0 in $CHCl_3$).

3. X-ray structural determination

The X-ray date was collected on a Rigaku Saturn CCDC diffractometer using graphite-monochromated Mo K*α* radiation (λ = 0.71073 Å). The structure was solved by direct methods (SHELXS-97)[2] and refined by full-matrix least squares on F^2. All non-hydrogen atoms were refined anisotropically and hydrogen atoms by a riding model (SHELXL-97).[3] The crystal data and structural refinements details are listed in Table S1. CCDC 2121510 (**23**) contains the supplementary crystallographic data for this paper. This data can be obtained free of charge from The Cambridge Crystallographic Data Centre via www.ccdc.cam.ac.uk/data_request/cif.

Table S1. Crystal Data and Summary of X-ray Data Collection for compound **23**

	**23**
formula	$C_{14}H_{17}N_2OP$
fw	260.26
T (K)	296
space group	P 21 21 21
crystal system	orthorhombic
a (Å)	7.2488(9)
b (Å)	10.7508(14)
c (Å)	17.638(2)
α (deg.)	90
β (deg.)	90
γ (deg.)	90
V (Å3)	1374.5(3)
Z	4
*d*calcd. (mg/cm3)	1.258
F(000)	552.0
GOF	1.094
R1 ($I > 2\sigma(I)$)	0.0339
*w*R2 (all data)	0.0887

4. Computational Methods

All the calculations were carried out via density functional theory (DFT) calculation using Gaussian 16 [4] with the ωB97XD [5] functional. Geometric structures of all species in this work were optimized in gas phase. In addition, free energy corrections were considered at a concentration of 1 M and a temperature of 298.15 K. Frequency calculation were performed to determine all the stationary points (no imaginary frequency) and transition state structures (only one imaginary frequency). The 6-31+G(d) [6] basis set was used for all atoms. In addition, the intrinsic reaction coordinate (IRC) calculation [7] were applied to confirm the connection of each transition state to its corresponding appropriate intermediates, reactants, or products. Base on the gas phase optimized geometries, solvent effects were computed by using the SMD [8] model at the same level of theory while 6-311++G(d,p) basis set for all atoms. Dimethylsulfoxide (ε = 46.826) was used as the solvent, and Bondi atomic radii [9] was used for the SMD calculation. All 3D molecular structures were drawn by using the CYLview (Version) program [10]. The Mulliken charge distribution were calculated by Gaussian 16 with the level of ωB97XD/6-31+G(d). The spin-orbit crossing (SOC) values were calculated by ORCA package.[11]

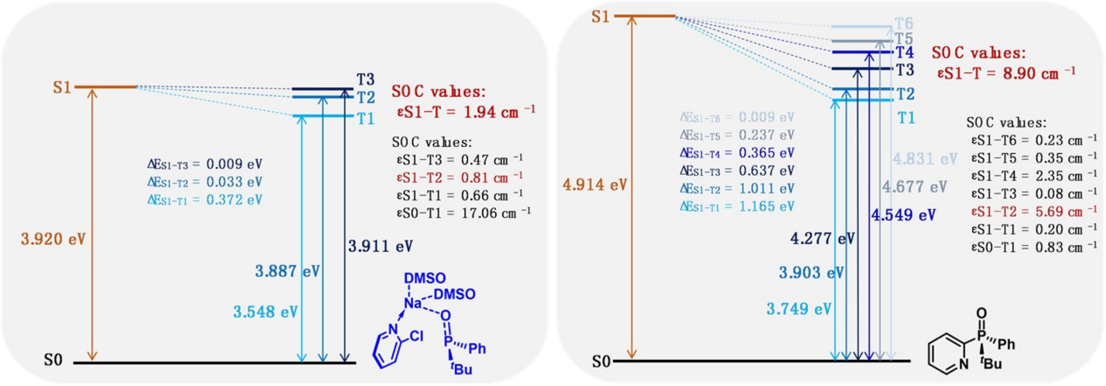

Figure S2. The spin-orbit coupling (SOC) values of **^{3}[INT-C]** and **^{3}[4]**. The data were obtained by ORCA package.

Table S2. The gas phase relative free energies (ΔG_{gas}), gas phase relative electronic energies (ΔE_{gas}) and solvation corrected relative electronic energies (ΔE_{sol}) calculated for species involved in Figure 1 and S4.

Species	ΔGgas	ΔEgas	ΔEsol
**INT-A**	0	0	0
**TS$_1$**	-0.1	0.9	1.7
**INT-B**	-10.8	-12.7	-13.2
**INT-C**	-10.2	2.5	-7.8
**^{3}[INT-C]**	41.2	56.2	45.1
**^{3}[TS$_2$]**	47.7	62.1	48.8
**^{3}[INT-D]**	27.1	42.2	26.5
**^{3}[TS$_3$]**	47.5	60.6	42.2

**[INT-C]***	43.7	30.8	21.8
**^{3}[4]**	69.0	113.1	72.2
**4**	-9.5	30.0	-9.6

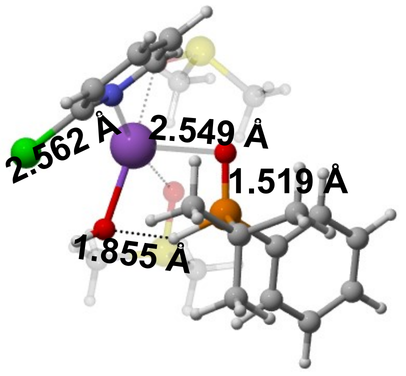

INT-A

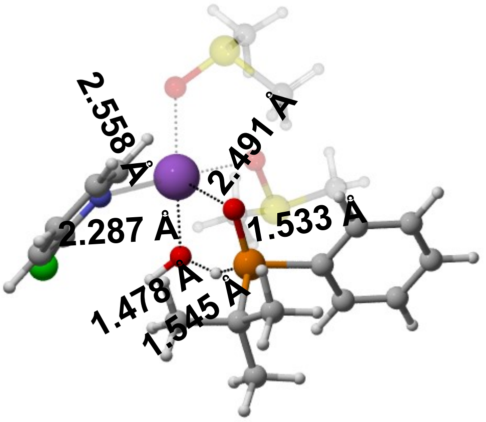

TS_1

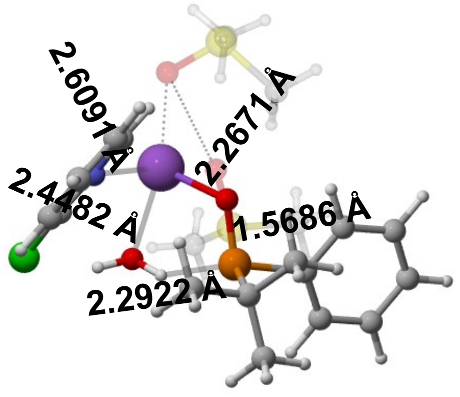

INT-B

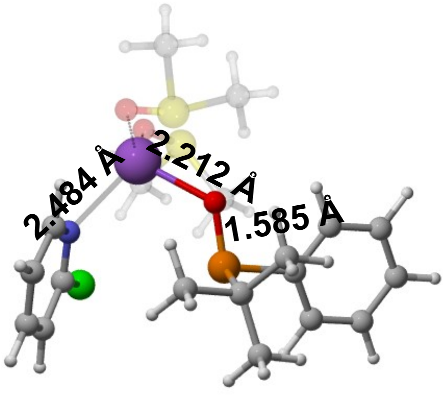

INT-C

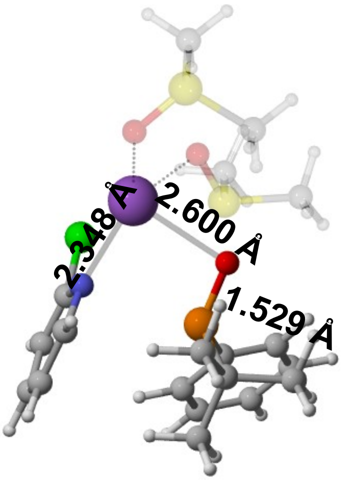

^{3}[INT-C]

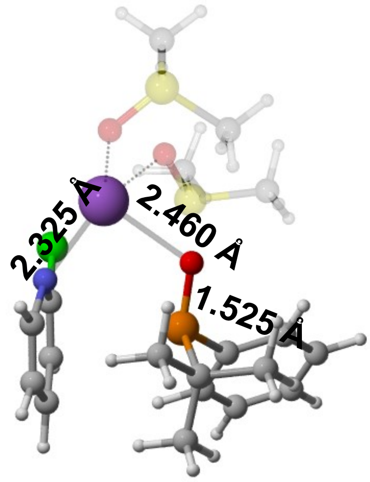

[INT-C]*

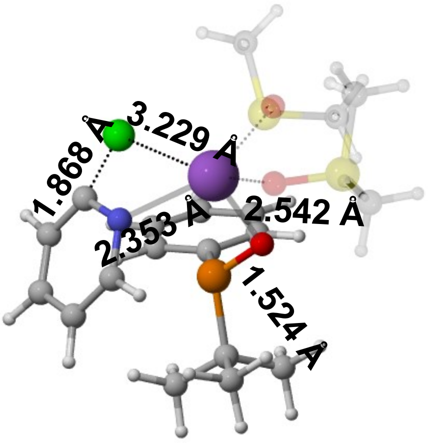

^{3}[TS$_2$]

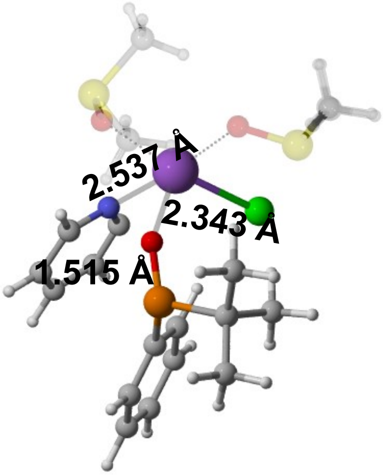

^{3}[INT-D]

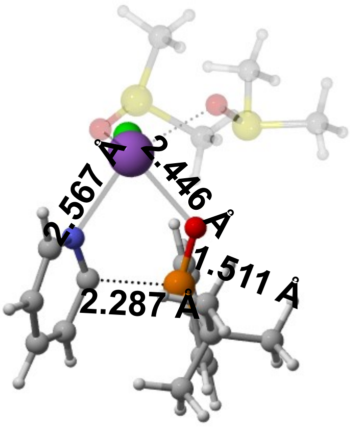

^{3}[TS$_3$]

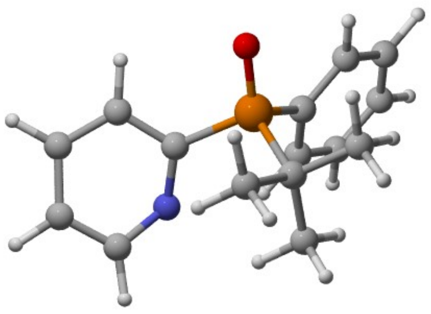

^{3}[4]

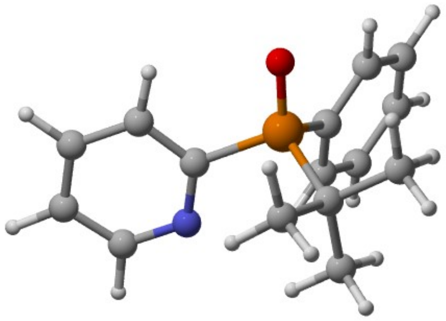

4

Table S3. Absolute Calculation Energies and imaginary frequencies.

Species	E($_{gas-ωB97XD}$) [1]	G($_{corr-ωB97XD}$) [2]	H($_{corr-ωB97XD}$) [3]	E($_{solv-ωB97XD}$) [4]	IF [5]
**INT-A**	-2858.779859	0.415255	0.528451	-2859.204566	-
**TS$_1$**	-2858.778420	0.413667	0.525253	-2859.201785	-625.80
**INT-B**	-2858.800083	0.418200	0.530840	-2859.225637	-
**INT-C**	-2782.379064	0.392087	0.502670	-2782.776262	-
**^{3}[INT-C]**	-2782.293526	0.388416	0.500064	-2782.692023	-
**^{3}[TS$_2$]**	-2782.284071	0.389322	0.499403	-2782.686064	-251.86
**^{3}[INT-D]**	-2782.315831	0.388290	0.502282	-2782.721631	-
**^{3}[TS$_3$]**	-2782.286418	0.391374	0.500490	-2782.696614	-451.09
**[INT-C]***	-2782.295863	0.394726	0.502652	-2782.673437	-
**^{3}[4]**	-1053.430809	0.249699	0.315773	-1053.638198	-
**4**	-1053.563191	0.256958	0.320239	-1053.768480	-

[1]The electronic energy calculated by ωB97XD in gas phase. [2]The thermal correction to Gibbs free energy calculated by ωB97XD in gas phase. [3]The thermal correction to enthalpy calculated by ωB97XD in gas phase. [4]The electronic energy calculated by ωB97XD in dimethylsulfoxide solvent. [5]The ωB97XD calculated imaginary frequencies for the transition states.

Cartesian coordinates for the complexes calculated in this study: [gas phase results optimized at the level of ωB97XD/6-31+G(d)]

INT-A

P	-0.92873500	0.35301500	1.15641700
H	0.40977300	0.03456900	1.57249700
O	-1.21230400	-0.06608900	-0.27565300
C	-1.13974300	2.15054200	1.37820900
C	-1.94012900	2.85865100	0.47802500
C	-0.47588700	2.84204000	2.39658800
C	-2.08458400	4.23925500	0.59919100

H	-2.43508500	2.31912700	-0.32524000
C	-0.62051000	4.22237100	2.52032400
H	0.17296500	2.30303900	3.08283700
C	-1.42539500	4.92212300	1.62110300
H	-2.70867700	4.78334700	-0.10459700
H	-0.10043900	4.75277700	3.31347500
H	-1.53570800	5.99910000	1.71521800
C	-2.04241600	-0.49199100	2.36530900
C	-3.50376900	-0.14105200	2.06184000
H	-4.16835300	-0.70732600	2.72755200
H	-3.70284300	0.92613300	2.21673000
H	-3.76168100	-0.39353700	1.02736500
C	-1.67081300	-0.08821500	3.79777600
H	-2.26563400	-0.67469900	4.50977900
H	-0.61217800	-0.28120700	4.01137300
H	-1.87586200	0.97118800	3.98887800
C	-1.80375500	-1.99811400	2.16151800
H	-0.75363700	-2.26710900	2.32966300
H	-2.41973400	-2.56943300	2.86838800
H	-2.07368000	-2.30335100	1.14565100
Na	1.16172200	-0.76863400	-0.88515500
S	0.29204500	0.69387100	-3.92919100
C	1.78909700	1.71195800	-3.96054900
H	1.55451100	2.68491500	-4.40401600
H	2.15036700	1.82102800	-2.93204000
H	2.52158400	1.18958000	-4.58048000
C	-0.66435500	1.72254900	-2.78863400
H	-0.07488500	1.90785500	-1.88577400
H	-0.93129300	2.65308000	-3.30107800
H	-1.55910900	1.15678100	-2.52146000
O	0.66783000	-0.57296600	-3.17424300
S	2.39075600	2.07270200	0.57665000
C	2.76631400	3.82538700	0.29614700
H	3.32791700	4.22316300	1.14748700
H	3.33656000	3.93944000	-0.63066100
H	1.80855000	4.34619300	0.21409400
C	4.07383600	1.43464500	0.58019700
H	4.56369100	1.69301900	-0.36365100
H	4.61735600	1.84930400	1.43518200
H	3.92325800	0.35509900	0.69982100
O	1.77745800	1.62588000	-0.75171400
O	2.06404200	-0.68385500	1.13772000
H	2.29724600	-1.43173700	1.69833700
C	0.01060600	-4.08567200	-0.15551500

C	-1.05826000	-4.95871000	0.04114400
C	-2.22353600	-4.70482100	-0.67207100
C	-2.26998000	-3.61094400	-1.53362400
C	-1.14030200	-2.80943900	-1.64666600
N	-0.01033100	-3.04545600	-0.96559600
H	-3.08551500	-5.35491400	-0.55194200
H	-0.97180100	-5.79464000	0.72543900
H	-3.16282700	-3.37737700	-2.10358000
H	-1.11173600	-1.93921700	-2.29436500
Cl	1.49680300	-4.36845600	0.71960800

TS_1

P	0.50869700	-1.17968200	-2.29110300
H	1.95092900	-1.63178200	-1.97017400
O	0.18939600	-1.50729400	-3.75436800
C	0.33073500	0.62490900	-2.01605900
C	-0.36949200	1.39154400	-2.95118800
C	0.95252900	1.26779500	-0.94001700
C	-0.45957200	2.77574000	-2.80840400
H	-0.83074600	0.88980600	-3.79794100
C	0.86104300	2.64991900	-0.79136200
H	1.53387200	0.68610400	-0.22792800
C	0.15402400	3.40684300	-1.72685600
H	-1.00856600	3.36229700	-3.54058400
H	1.34729300	3.13851400	0.04904900
H	0.08542300	4.48563000	-1.61407000
C	-0.71984700	-1.98550700	-1.15056600
C	-2.14203500	-1.54693700	-1.51746200
H	-2.87365900	-2.09356700	-0.90683200
H	-2.29007600	-0.47498900	-1.34048900
H	-2.35378500	-1.75304600	-2.57254300
C	-0.40442500	-1.63835200	0.30928800
H	-1.06144400	-2.21215100	0.97621700
H	0.63225800	-1.88641400	0.57018800
H	-0.56776900	-0.57523500	0.51858500
C	-0.56203300	-3.49816300	-1.37558500
H	0.45469400	-3.83725400	-1.14019200
H	-1.25962300	-4.04782300	-0.72936600
H	-0.77686100	-3.76357300	-2.41568300
Na	2.48245400	-2.20049300	-4.43871300
S	1.56366800	-0.85671600	-7.48840800
C	3.05546000	0.15699700	-7.64729300
H	2.79012500	1.12127000	-8.09237000
H	3.49151600	0.28690200	-6.65101500

H	3.74203700	-0.37854900	-8.30717300
C	0.68604100	0.20031500	-6.31213100
H	1.34692300	0.42921500	-5.47091200
H	0.35757100	1.10531800	-6.83424900
H	-0.16814500	-0.37075300	-5.94174000
O	1.99068100	-2.10347400	-6.72520000
S	3.69025700	0.62557200	-3.05066800
C	3.97763300	2.39632000	-3.29934200
H	4.40338600	2.82979500	-2.38909800
H	4.64409400	2.54914200	-4.15345100
H	3.00198900	2.84973100	-3.49335000
C	5.39631900	0.10128300	-2.81254500
H	5.98543700	0.36233400	-3.69670800
H	5.80070400	0.57496700	-1.91258100
H	5.32048600	-0.98047100	-2.67095200
O	3.29456500	0.11740700	-4.43851100
O	3.31043900	-2.10508100	-2.30908900
H	3.50819200	-2.91068300	-1.81691600
C	1.42401700	-5.53754400	-3.59599400
C	0.38048000	-6.43917100	-3.39593700
C	-0.78461800	-6.22962000	-4.12357500
C	-0.85458700	-5.14934200	-5.00035600
C	0.25128900	-4.31573500	-5.11411700
N	1.38193900	-4.50952300	-4.41988500
H	-1.62854800	-6.90255800	-4.00191800
H	0.48479000	-7.26200200	-2.69846700
H	-1.74850100	-4.94955700	-5.58125400
H	0.25976900	-3.45518100	-5.77501500
Cl	2.91154100	-5.76679300	-2.70279300

INT-B

P	-0.62063200	0.20054600	1.43763200
H	1.51499600	-0.62995400	1.49689700
O	-0.84278800	-0.03205600	-0.09759900
C	-0.82531500	2.03803100	1.67119000
C	-1.43961200	2.81042000	0.67918800
C	-0.30027900	2.69527300	2.78991600
C	-1.54888200	4.19490900	0.80823700
H	-1.83375800	2.30090100	-0.19644800
C	-0.40575300	4.07940400	2.92810000
H	0.20453700	2.11400900	3.56048200
C	-1.03189400	4.83479500	1.93574000
H	-2.04099600	4.77670100	0.03192400
H	0.00227000	4.57024900	3.80866100

H	-1.11469000	5.91363000	2.03990700
C	-2.19127400	-0.42418000	2.29287800
C	-3.44926500	0.16089800	1.64522600
H	-4.35024700	-0.32892300	2.04303600
H	-3.53968000	1.23578900	1.84030400
H	-3.42656600	0.01064400	0.55932700
C	-2.15145000	-0.09776800	3.78850000
H	-3.00161800	-0.56776400	4.30299800
H	-1.23097800	-0.47079500	4.25700700
H	-2.21151400	0.98198300	3.96868800
C	-2.17964700	-1.94684900	2.09360600
H	-1.28656300	-2.40451800	2.53993300
H	-3.06285000	-2.40363300	2.56310300
H	-2.19299000	-2.19558900	1.02701600
Na	1.09280100	-0.67684100	-1.08638300
S	0.00911600	0.61478800	-4.01923000
C	1.47481200	1.62827400	-4.33722600
H	1.16076000	2.60539400	-4.71766200
H	2.03455800	1.73098700	-3.40176400
H	2.07173700	1.11150300	-5.09220800
C	-0.71200000	1.64135600	-2.71781100
H	0.06354300	1.90893400	-1.99464000
H	-1.16617300	2.52561900	-3.17667000
H	-1.46021200	1.03225800	-2.20323300
O	0.52477300	-0.64807700	-3.33657100
S	2.39548600	1.98724900	0.30072700
C	2.21375000	3.78119100	0.23543500
H	2.51872100	4.21018800	1.19413900
H	2.81268000	4.18144400	-0.58806200
H	1.15150700	3.98567100	0.07907400
C	4.18197500	1.90223300	0.54371900
H	4.68847200	2.39366500	-0.29202400
H	4.44347100	2.37314500	1.49600900
H	4.43178200	0.83970500	0.57694700
O	2.16324200	1.50330400	-1.13128400
O	2.32349600	-0.94230600	1.01323100
H	2.36787900	-1.89072500	1.19325700
C	0.10390900	-4.06990500	0.02464800
C	-0.97072500	-4.92493900	0.25227500
C	-2.11153400	-4.71812600	-0.51340800
C	-2.12435500	-3.68583200	-1.44788700
C	-0.99068200	-2.89389300	-1.57910400
N	0.12025100	-3.08641000	-0.85059800
H	-2.98112600	-5.35340300	-0.37369200

H	-0.91008100	-5.70849100	0.99818700
H	-2.99796000	-3.48797900	-2.05925100
H	-0.94342600	-2.07321800	-2.28877800
Cl	1.57017700	-4.31633800	0.96341600

INT-C

P	-0.72865300	-1.12752100	0.37990600
C	-1.33145200	-1.39396800	2.12508700
C	-1.90201200	-0.34950600	2.86129200
C	-1.14628100	-2.62567700	2.76342200
C	-2.29493300	-0.53156600	4.18621800
H	-2.02635100	0.61652200	2.37560600
C	-1.54415800	-2.81998000	4.08624100
H	-0.67675000	-3.44285300	2.21741300
C	-2.11973300	-1.77103100	4.80317800
H	-2.73976800	0.29227500	4.74008900
H	-1.39654100	-3.78693400	4.56144000
H	-2.42385800	-1.91647900	5.83662200
C	-2.30601800	-1.64317500	-0.55957000
C	-3.52400100	-0.81160500	-0.15178900
H	-4.38313000	-1.03986700	-0.80039900
H	-3.82146700	-1.01343800	0.88347800
H	-3.30649600	0.25934700	-0.23926500
C	-2.58335800	-3.13409800	-0.34281400
H	-3.40338900	-3.46861500	-0.99466100
H	-1.70040600	-3.74358100	-0.57738900
H	-2.87548100	-3.34521600	0.69222300
C	-1.99372100	-1.39643500	-2.04180500
H	-1.12557400	-1.98437300	-2.36645600
H	-2.84997400	-1.68003300	-2.67162000
H	-1.77287300	-0.33870300	-2.22632100
O	-0.69117200	0.45071600	0.24193700
Na	1.02685100	1.52814300	-0.64195100
S	2.78328100	1.69984500	2.36063700
C	4.32159400	0.74856500	2.29179100
H	4.49240000	0.25893200	3.25511400
H	4.24709500	0.01238400	1.48693600
H	5.13251200	1.44907900	2.08096200
C	1.65493100	0.31874700	2.63744800
H	1.79949500	-0.44836200	1.86996100
H	1.81686500	-0.09006300	3.63950600
H	0.63849000	0.70832600	2.55230600
O	2.53940600	2.18190200	0.93784500
C	2.12499100	-1.24526900	-2.33082700

C	1.92897300	-2.30298200	-3.21540300
C	1.14863100	-2.05434800	-4.33773800
C	0.60149400	-0.78636300	-4.52114200
C	0.85067100	0.18519300	-3.56102700
N	1.60933600	-0.03963300	-2.47821700
H	0.96535000	-2.84731200	-5.05673500
H	2.37000800	-3.27384600	-3.02360300
H	-0.02061700	-0.55843000	-5.37974200
H	0.42616600	1.18384400	-3.62454100
Cl	3.13991400	-1.49549300	-0.93057600
S	-1.81053000	2.76392900	-1.91150900
C	-2.40941700	4.35535600	-2.53456500
H	-3.48841800	4.42962900	-2.36913400
H	-1.87917800	5.16304400	-2.02208400
H	-2.19483600	4.39308700	-3.60486600
C	-2.18536900	3.01398300	-0.16643200
H	-1.69270200	3.92841600	0.17872000
H	-3.27048500	3.07812600	-0.03767600
H	-1.78630100	2.11956800	0.33504800
O	-0.29148500	2.84763900	-2.04507700

^{3}[INT-C]

C	-2.29975700	1.82088900	0.03506100
C	-0.88377400	2.41340300	1.74520100
C	-1.94542100	2.56050100	2.60932500
C	-3.26243800	2.29016600	2.18452200
C	-3.42433300	1.95014500	0.79728800
H	0.12612500	2.67921500	2.04872900
H	-1.75092200	2.90540500	3.62285300
H	-4.11931600	2.41888500	2.83569800
H	-4.40492200	1.80778700	0.35652100
N	-1.00991900	1.97791400	0.41674200
Cl	-2.48861700	1.42707300	-1.69460000
Na	0.79097000	1.50161000	-1.01244300
P	0.39621700	-0.40995700	1.55105600
O	1.37837400	-0.60382100	0.39547800
C	-0.99894100	-1.57118400	1.45650400
C	-0.80497300	-2.87435900	0.97877900
C	-2.28115300	-1.14709900	1.81586900
C	-1.88398600	-3.74476800	0.86311800
H	0.19377700	-3.20279000	0.70232800
C	-3.36055500	-2.02110400	1.69309000
H	-2.44660700	-0.13455000	2.17552100

C	-3.16568200	-3.31543200	1.21656700
H	-1.72867100	-4.75907400	0.50365200
H	-4.35600000	-1.67982300	1.96215200
H	-4.00995000	-3.99271700	1.12017500
C	1.22679100	-0.65046000	3.20943700
C	1.83994200	-2.05803700	3.26713300
H	2.36588600	-2.18629900	4.22287000
H	1.07121200	-2.83629700	3.20359000
H	2.56199000	-2.21146000	2.45766700
C	0.18602700	-0.46559900	4.32021300
H	0.68175900	-0.53445900	5.29709000
H	-0.30464300	0.51213700	4.25267100
H	-0.58953400	-1.23833200	4.28207000
C	2.32743000	0.41333600	3.32212200
H	1.90851700	1.42584900	3.30957500
H	2.86641900	0.28430300	4.26945000
H	3.05057200	0.32983200	2.50374600
S	3.94423100	1.63084900	-2.26523200
C	3.33413200	1.30010700	-3.93842800
H	4.06474500	0.68655600	-4.47491100
H	2.36606900	0.79323600	-3.86842700
H	3.22805400	2.26609200	-4.43748500
C	3.87927000	-0.07374000	-1.65990100
H	2.86941000	-0.46305300	-1.81473800
H	4.62855600	-0.67136900	-2.18885500
H	4.10032800	-0.04963100	-0.59092000
O	2.82345400	2.37304300	-1.55320700
S	-0.24973500	-1.25705800	-2.17371600
C	0.79434800	-2.73022200	-2.12278600
H	0.18117100	-3.60933800	-1.90429200
H	1.31162500	-2.83968400	-3.08067500
H	1.50896700	-2.56241300	-1.31342500
C	-1.27566100	-1.72327200	-3.58547400
H	-0.64020500	-1.92974500	-4.45132700
H	-1.87807400	-2.59672200	-3.31839500
H	-1.93201300	-0.87566100	-3.79265700
O	0.66882000	-0.15040900	-2.69589900

^{3}[TS$_2$]

C	-1.83969100	2.38835400	0.35059900
C	-0.10098500	3.14037700	1.67306200
C	-0.88254000	3.16877200	2.80849000
C	-2.24005400	2.70661500	2.70279700
C	-2.68283600	2.26131700	1.47890200

H	0.94442700	3.44942100	1.72576300
H	-0.47518400	3.52646800	3.74909000
H	-2.91167800	2.74873800	3.55674500
H	-3.69409400	1.88440800	1.34511800
N	-0.51394200	2.75515000	0.44484700
Cl	-2.15853400	1.16355900	-1.02312800
Na	1.01832400	1.74386700	-1.02632200
P	0.37396800	-0.17897900	1.63185700
O	1.43152500	-0.21859600	0.53566000
C	-0.82671200	-1.53698100	1.45284000
C	-0.41297600	-2.77923800	0.95687800
C	-2.17022800	-1.33087800	1.77473100
C	-1.33469000	-3.80907200	0.79506900
H	0.63257600	-2.93508100	0.70279900
C	-3.09164700	-2.36339700	1.60981900
H	-2.49594300	-0.35583100	2.12977700
C	-2.67624700	-3.60011700	1.12054000
H	-1.00891700	-4.77656700	0.42138700
H	-4.13698700	-2.19604100	1.85217100
H	-3.39704400	-4.40271100	0.99074700
C	1.11772500	-0.30602400	3.34123000
C	1.83291400	-1.66186600	3.46269200
H	2.31084100	-1.72641000	4.44915900
H	1.13425100	-2.50112100	3.37577700
H	2.61230100	-1.77275600	2.70058300
C	-0.00022300	-0.18544900	4.38422400
H	0.44104400	-0.21299900	5.38869200
H	-0.54594500	0.75818100	4.27492500
H	-0.71540400	-1.01252100	4.31183400
C	2.12691700	0.83887400	3.49858100
H	1.62867900	1.81201900	3.45818800
H	2.62461900	0.74897600	4.47254400
H	2.89444600	0.80796900	2.71790400
S	4.28213700	1.67615800	-2.04577500
C	3.80857200	1.19406400	-3.72648100
H	4.56357100	0.51436500	-4.13413100
H	2.82183100	0.72012500	-3.69302600
H	3.77595200	2.10782100	-4.32437200
C	4.12544000	0.03905200	-1.28901400
H	3.11476600	-0.33452800	-1.47364300
H	4.88748500	-0.62649900	-1.70702600
H	4.27763700	0.16015500	-0.21460000
O	3.12490600	2.50004700	-1.50561700
S	0.07846500	-1.15253300	-2.15314300

C	1.06683500	-2.65600700	-2.34139500
H	0.43769100	-3.53206900	-2.15784400
H	1.50391300	-2.68821800	-3.34377800
H	1.85578200	-2.60642500	-1.58708600
C	-1.05929500	-1.42755300	-3.52854900
H	-0.49505000	-1.55840800	-4.45632700
H	-1.67922200	-2.30284900	-3.31267100
H	-1.69104400	-0.53876600	-3.59207100
O	1.00015100	-0.02384500	-2.61505000

^{3}[INT-D]

C	-3.16776200	1.28758700	-0.73922700
C	-1.63194100	2.49063100	0.41445900
C	-2.58394800	2.83998200	1.36223600
C	-3.89290300	2.37009400	1.21566100
C	-4.21864500	1.56805300	0.12410200
H	-0.59851300	2.81725700	0.46855000
H	-2.30635000	3.46351000	2.20584200
H	-4.65157700	2.62414200	1.95176600
H	-5.21831600	1.17954400	-0.03362000
N	-1.94977400	1.69843800	-0.62990800
Cl	-1.04949100	-1.93223400	-1.42163000
Na	0.15123000	0.43992800	-1.29279800
P	0.63762300	-0.09845100	2.33950000
O	0.78465400	0.51378800	0.96184000
C	-1.09173000	-0.37987000	2.81874100
C	-2.01066700	-0.82875600	1.86175400
C	-1.50072800	-0.15275200	4.13791400
C	-3.33042500	-1.05983600	2.23988000
H	-1.70257900	-1.02286600	0.83400300
C	-2.82525300	-0.37903900	4.50427300
H	-0.78873700	0.20725400	4.87768800
C	-3.73897700	-0.83422300	3.55352300
H	-4.03609400	-1.41787900	1.49608900
H	-3.14318300	-0.20070000	5.52776000
H	-4.77188200	-1.01455300	3.83961300
C	1.57987400	-1.70596800	2.49803400
C	0.81478200	-2.80713100	1.74538400
H	1.42784400	-3.71895100	1.74070200
H	-0.13443500	-3.04464800	2.23638900
H	0.59235100	-2.53592500	0.70716400
C	1.71994000	-2.06571100	3.98228200
H	2.25238200	-3.02066100	4.07485700
H	2.28693600	-1.30837200	4.53676000

H	0.74165300	-2.18711400	4.46186300
C	2.95447400	-1.47700900	1.85538800
H	3.51848400	-0.68554000	2.36415500
H	3.53986500	-2.40320200	1.91869600
H	2.85135700	-1.20057400	0.80121600
S	2.51818400	2.98303500	-1.57930500
C	3.24866900	2.27524000	-3.07881700
H	4.33044800	2.44260000	-3.07060800
H	3.01019600	1.20654600	-3.10676100
H	2.80107700	2.79442600	-3.92950100
C	3.34148400	1.89112800	-0.39183700
H	3.13862700	0.85256800	-0.67000000
H	4.41420900	2.11109900	-0.39466900
H	2.91133600	2.09975700	0.58946300
O	1.05415500	2.58004600	-1.60209900
S	2.24728200	-2.15112200	-2.06844900
C	3.96364000	-2.54120900	-2.50875300
H	4.07131800	-3.62119100	-2.64828600
H	4.24015300	-2.00308400	-3.42017300
H	4.59449000	-2.21389800	-1.67872300
C	1.48282000	-2.67313300	-3.61704400
H	1.92742900	-2.10980800	-4.44275700
H	1.63391000	-3.74942200	-3.74421500
H	0.41623700	-2.45933600	-3.50760800
O	2.21160000	-0.62171000	-2.07040000

^{3}[TS$_3$]

C	-0.66540500	-0.07682100	1.00504000
C	0.47660300	1.86947300	0.69761200
C	0.09549800	2.30978600	1.96685600
C	-0.85540200	1.56544200	2.69851600
C	-1.29469300	0.35112900	2.21014500
H	1.11946800	2.44946300	0.04113800
H	0.44651600	3.26978100	2.33085000
H	-1.25657900	1.95855400	3.62945200
H	-2.00430100	-0.27896300	2.73574000
N	-0.01800000	0.73406300	0.19354600
Cl	-0.79698000	-1.98704900	-2.95299800
Na	1.05601100	-0.52881900	-1.76614100
P	0.86354200	-1.70560500	1.49565700
O	1.71454600	-1.75129900	0.24762800
C	-0.37213700	-3.02960000	1.54480600
C	-0.89720000	-3.39682400	0.29687900
C	-0.89849000	-3.60298400	2.71134100

C	-1.91861000	-4.34042600	0.22184800
H	-0.54010600	-2.94047900	-0.62566600
C	-1.91097400	-4.55368800	2.62685600
H	-0.52043800	-3.33034800	3.69065800
C	-2.42310000	-4.92308500	1.38177500
H	-2.31990000	-4.59782900	-0.75380700
H	-2.30238200	-5.00428500	3.53475200
H	-3.22097600	-5.65821900	1.32135900
C	1.94024100	-1.64320800	3.02742300
C	2.52446200	-3.04278200	3.28687300
H	3.25763400	-2.98051200	4.10230200
H	1.75919800	-3.76823700	3.57832700
H	3.03749800	-3.42756100	2.39906100
C	1.19175400	-1.11513200	4.25932900
H	1.86990900	-1.13320200	5.12213600
H	0.86500800	-0.08182700	4.11110100
H	0.31253800	-1.70950300	4.52283000
C	3.08062000	-0.66965600	2.68466600
H	2.69876700	0.32671200	2.43154500
H	3.74011500	-0.57018400	3.55619300
H	3.66930600	-1.03542000	1.83928500
S	3.90792900	1.39616600	-2.02107300
C	4.14894100	0.62922200	-3.64250200
H	5.22215400	0.53615700	-3.83672600
H	3.65975800	-0.35034100	-3.65453400
H	3.69810700	1.29847900	-4.37902400
C	4.56242000	0.04602600	-1.00867400
H	3.98934100	-0.86349800	-1.22055100
H	5.62557300	-0.08934400	-1.23211800
H	4.43692200	0.34598800	0.03404500
O	2.40494900	1.40596800	-1.77568700
S	2.19074000	-3.42993800	-2.42252700
C	3.65160000	-4.45669900	-2.10961600
H	3.36006300	-5.51079400	-2.07093300
H	4.39589500	-4.28681700	-2.89326600
H	4.05210600	-4.15043800	-1.14034800
C	1.80516300	-4.04334200	-4.07447700
H	2.67037500	-3.89517300	-4.72735000
H	1.53150000	-5.10107400	-4.01116500
H	0.94460600	-3.45702600	-4.40770900
O	2.77019300	-2.02986500	-2.63549700

[INT-C]*

C	-2.01810800	2.10295500	0.03203900

C	-0.53444000	2.99474200	1.52843400
C	-1.39602000	2.76018300	2.56922100
C	-2.61954700	2.07254900	2.35568100
C	-2.95802500	1.82814500	0.98244700
H	0.39621200	3.53566100	1.68224500
H	-1.13194700	3.12099600	3.56126800
H	-3.35595400	1.95324100	3.14167500
H	-3.93984600	1.46893200	0.69217800
N	-0.76504600	2.58351200	0.21527400
Cl	-2.45187500	1.85529500	-1.68604300
Na	0.98263300	1.66287300	-1.01100400
P	0.07790800	-0.38014200	1.55317400
O	1.08232900	-0.34498800	0.40678400
C	-1.02669200	-1.83033000	1.42630200
C	-0.52698900	-3.08455200	1.05944400
C	-2.39087200	-1.67699700	1.68647200
C	-1.38368900	-4.17794300	0.95665300
H	0.53399600	-3.20480200	0.85736000
C	-3.24734600	-2.77193300	1.57934600
H	-2.77670600	-0.69942100	1.96165500
C	-2.74548300	-4.01998300	1.21544700
H	-0.98991700	-5.15238600	0.68022900
H	-4.30799100	-2.64780900	1.77662900
H	-3.41461700	-4.87228700	1.13566300
C	0.91075800	-0.44778500	3.21957300
C	1.74622500	-1.73358000	3.32448200
H	2.28195700	-1.73381700	4.28258200
H	1.11965000	-2.63149100	3.29533400
H	2.48887000	-1.79580200	2.52128200
C	-0.16668500	-0.41434100	4.31088700
H	0.31671100	-0.39659400	5.29573600
H	-0.79990600	0.47352700	4.22168900
H	-0.81060900	-1.30042800	4.27197000
C	1.82600900	0.78035000	3.32305200
H	1.25147500	1.70852800	3.26691400
H	2.35407500	0.75697600	4.28464700
H	2.57447100	0.79023800	2.52344200
S	4.14194100	1.21596800	-2.19671600
C	3.52419600	0.95236900	-3.87894800
H	4.14627100	0.20336700	-4.37906600
H	2.47903500	0.62965400	-3.82689700
H	3.60616500	1.90722600	-4.40325600
C	3.76059600	-0.43379100	-1.55591000
H	2.69983700	-0.63627900	-1.72651700

H	4.39793100	-1.17094800	-2.05457500
H	3.96513200	-0.42146900	-0.48340900
O	3.15416200	2.16119900	-1.53204900
S	-0.47223700	-0.94476300	-2.23419000
C	0.31990200	-2.56858500	-2.24811200
H	-0.42130700	-3.34231700	-2.02851000
H	0.79261600	-2.73501500	-3.22071900
H	1.07183900	-2.54680100	-1.45725600
C	-1.53833900	-1.18144700	-3.67254200
H	-0.92891000	-1.44689000	-4.54094700
H	-2.27350500	-1.96094800	-3.45138100
H	-2.05010400	-0.23308400	-3.84543200
O	0.61919700	0.01966000	-2.69766400

^{3}[4]

C	-3.75707800	-0.65556400	-0.01282400
C	-3.40624600	0.14130700	1.06496700
C	-3.39961300	1.53700200	0.89392800
C	-3.95398100	2.08904000	-0.31667600
C	-4.24961800	1.25745100	-1.35326400
N	-3.70479700	-0.01903500	-1.24966100
H	-3.19529700	2.18753800	1.73699000
H	-3.28076800	-0.32039700	2.04024200
H	-4.18516900	3.14731500	-0.38796400
H	-4.71047000	1.55510700	-2.28869200
P	-4.28599000	-2.37541300	0.22476400
O	-4.29510700	-2.74268700	1.67911700
C	-3.14247300	-3.41433600	-0.74019100
C	-2.84201600	-4.67691100	-0.21769300
C	-2.58304600	-3.01845100	-1.95959300
C	-2.00543000	-5.54237100	-0.91877500
H	-3.25613800	-4.96596600	0.74436600
C	-1.74687500	-3.88646900	-2.65761400
H	-2.78384000	-2.02772900	-2.35902500
C	-1.46097800	-5.14941700	-2.14029000
H	-1.77408800	-6.52088400	-0.50772800
H	-1.31128600	-3.57245400	-3.60189900
H	-0.80595600	-5.82337300	-2.68545700
C	-5.95773300	-2.51460300	-0.57043700
C	-6.45574600	-3.94679600	-0.31475900
H	-7.47093400	-4.05450200	-0.71703200
H	-5.81979200	-4.69024000	-0.80940100
H	-6.48302600	-4.17222200	0.75588000
C	-5.91848500	-2.23912400	-2.07901900

H	-6.93822300	-2.29798300	-2.48026200
H	-5.53408200	-1.23814100	-2.31042300
H	-5.30560400	-2.97199900	-2.61369800
C	-6.87980100	-1.50415500	0.13148500
H	-6.56876800	-0.47030300	-0.06116100
H	-7.90378900	-1.62383900	-0.24415500
H	-6.89057600	-1.66164600	1.21481700

4

C	-3.98941500	-0.58728300	-0.01814200
C	-3.71660800	0.14431200	1.13747100
C	-3.40315900	1.49459100	1.00654900
C	-3.37705200	2.05530200	-0.26598500
C	-3.66631500	1.23911900	-1.35855000
N	-3.96876800	-0.05558300	-1.24844700
H	-3.18352000	2.09737000	1.88309000
H	-3.75426800	-0.35285300	2.10168400
H	-3.13799800	3.10331500	-0.41688700
H	-3.65611500	1.64155400	-2.36899200
P	-4.41923600	-2.36348700	0.18490500
O	-4.39133600	-2.74118400	1.63997300
C	-3.21181500	-3.31345200	-0.79294300
C	-2.70221500	-4.47178200	-0.19716900
C	-2.79629900	-2.95532100	-2.08108100
C	-1.79409000	-5.27205400	-0.88764100
H	-3.01425800	-4.72893700	0.81143600
C	-1.88579900	-3.75638000	-2.76545900
H	-3.17494800	-2.04666400	-2.53929700
C	-1.38740000	-4.91617900	-2.17208500
H	-1.40099200	-6.17013800	-0.41964000
H	-1.56292300	-3.47307600	-3.76335900
H	-0.67768200	-5.53898900	-2.70978400
C	-6.09630700	-2.61491000	-0.57290100
C	-6.51241600	-4.05318500	-0.21911700
H	-7.52068100	-4.24139100	-0.60920400
H	-5.83713700	-4.78992600	-0.67043400
H	-6.52222900	-4.21264700	0.86320700
C	-6.11770900	-2.42765700	-2.09563700
H	-7.14948700	-2.54769200	-2.45065500
H	-5.76568800	-1.43504400	-2.39158200
H	-5.50350100	-3.17892100	-2.60307300
C	-7.05229400	-1.61419300	0.09788900
H	-6.81813700	-0.58140700	-0.18477000
H	-8.08012200	-1.82341400	-0.22409700

H	-7.01489900	-1.69406500	1.18959100

5. References

1 (*a*) F. A. Kortmann, M.-C. Chang, E. Otten, E. P. A. Couzijn, M. Lutzc and A. J. Minnaard, *Chem. Sci.*, 2014, **5**, 1322; (*b*) G. Tran, D. G. Pardo, T. Tsuchiya, S. Hillebrand, J.-P. Vors and J. Cossy, *Org. Lett.*, 2013, **15**, 21, 5550; (*c*) Y. Niu, P.-B. Bai, Q.-X. Lou and S.-D. Yang, *ChemCatChem*, 2020, **12**, 3644. (*d*) B. Varga, P. Szemesi, P. Nagy, R. Herbay, T. Holczbauer, E. Fogassy, G. Keglevich and P. Bagi, *J. Org. Chem.*, 2021, **86**, 14493.

2 G. M. Sheldrick, SHELXS-90/96, Program for Structure Solution, *Acta Crystallogr. Sect A*, 1990, **46**, 467.

3 G. M. Sheldrick, SHELXL 97, Program for Crystal structure Refinement, University of Goettingen:Geottingen, Germany, 1997.

4 M. J. Frisch, G. W. Trucks, H. B. Schlegel, G. E. Scuseria, M. A. Robb, J. R. Cheeseman, G. Scalmani, V. Barone, G. A. Petersson, H. Nakatsuji, X. Li, M. Caricato, A. V. Marenich, J. Bloino, B. G. Janesko, R. Gomperts, B. Mennucci, H. P. Hratchian, J. V. Ortiz, A. F. Izmaylov, J. L. Sonnenberg, D. Williams-Young, F. Ding, F. Lipparini, F. Egidi, J. Goings, B. Peng, A. Petrone, T. Henderson, D. Ranasinghe, V. G. Zakrzewski, J. Gao, N. Rega, G. Zheng, W. Liang, M. Hada, M. Ehara, K. Toyota, R. Fukuda, J. Hasegawa, M. Ishida, T. Nakajima, Y. Honda, O. Kitao, H. Nakai, T. Vreven, K. Throssell, J. A. Montgomery, Jr., J. E. Peralta, F. Ogliaro, M. J. Bearpark, J. J. Heyd, E. N. Brothers, K. N. Kudin, V. N. Staroverov, T. A. Keith, R. Kobayashi, J. Normand, K. Raghavachari, A. P. Rendell, J. C. Burant, S. S. Iyengar, J. Tomasi, M. Cossi, J. M. Millam, M. Klene, C. Adamo, R. Cammi, J. W. Ochterski, R. L. Martin, K. Morokuma, O. Farkas, J. B. Foresman, and D. J. Fox, Gaussian, Inc., Wallingford CT, **2016**.

5 (a) S.-Y. Song, Y. Li, Z. Ke, S. Xu, Iridium-Catalyzed Enantioselective C–H Borylation of Diarylphosphinates. *ACS Catal.* **2021**, 11, 13445–13451. (b) A. Maiti, F. Zhang, I. Krummenacher, M. Bhattacharyya, S. Mehta, M. Moos, C. Lambert, B. Engels, A. Mondal, H. Braunschweig, P. Ravat, A. Jana, Anionic Boron- and Carbon-Based Hetero-Diradicaloids Spanned by a p-Phenylene Bridge. *J. Am. Chem. Soc.* **2021**, 143, 3687.

6 P. C. Hariharan, J. A. Pople, The Influence of Polarization Functions on Molecular Orbital Hydrogenation Energies. *Theoret. Chim. Acta.* **1973**, 28, 213.

7 K. Fukui, The Path of Chemical Reactions - the IRC Approach. *Acc. Chem. Res.* **1981**, 14, 363.

8 A. V. Marenich, C. J. Cramer, D. G. Truhlar, Universal Solvation Model Based on Solute Electron Density and on a Continuum Model of the Solvent Defined by the Bulk Dielectric Constant and Atomic Surface Tensions. *J. Phys. Chem. B.* **2009**, 113, 6378.

9 A. Bondi, van der Waals Volumes and Radii. *J. Phys. Chem.* **1964**, 68, 441.

10 C. Y. Legault, CYLview, 1.0b; Universitéde Sherbrooke: Quebec, Canada, **2009** (http://www.cylview.org).

11 (*a*) F. Neese, The ORCA program system, *Wiley Interdiscip. Rev.: Comput. Mol. Sci.* **2012**, 2, 73. (*b*) F. Neese, Software update: the ORCA program system, version 4.0, *Wiley Interdiscip. Rev.: Comput. Mol. Sci.* **2017**, 8, e1327.

6. ^{1}H, ^{13}C, ^{19}F and ^{31}P NMR spectra for all products.

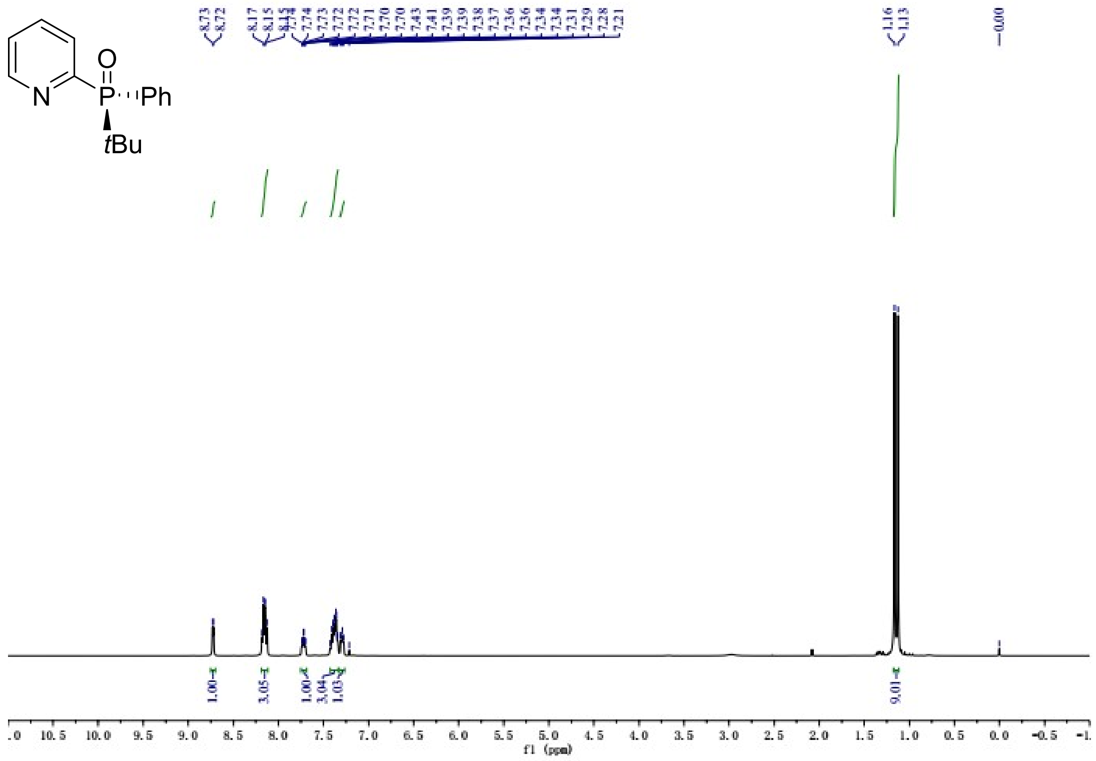

Figure S3. ^{1}H NMR spectrum of **4** in $CDCl_3$

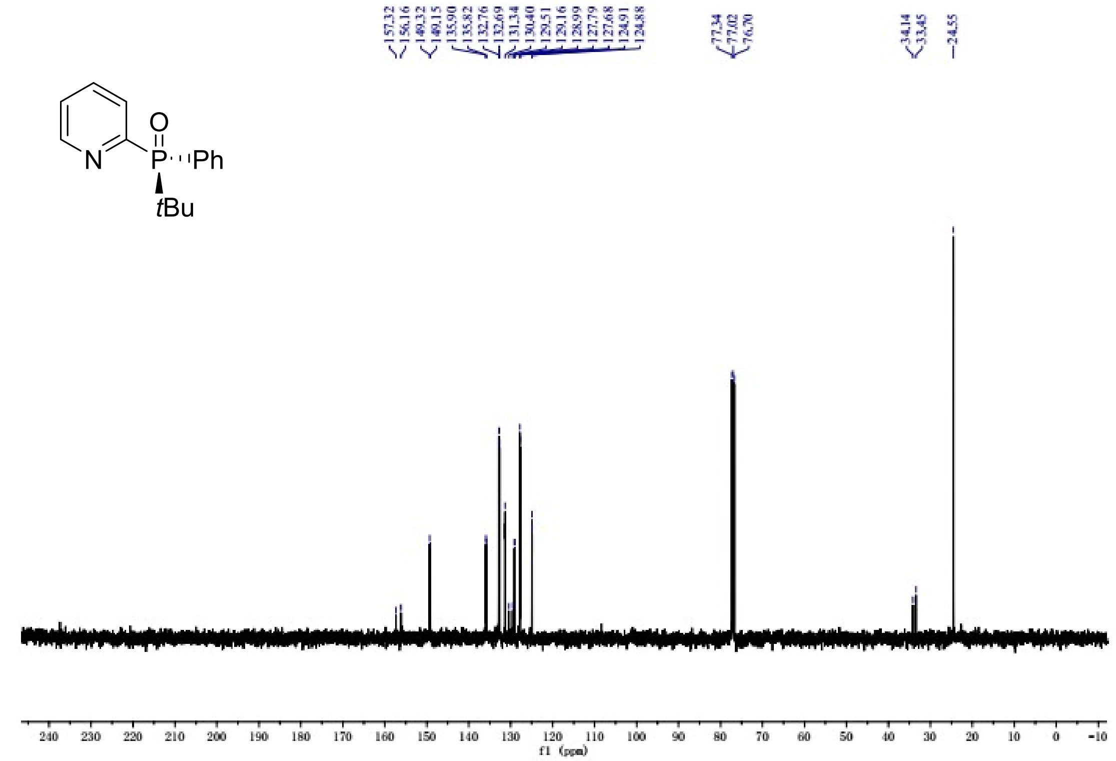

Figure S4. ^{13}C NMR spectrum of **4** in $CDCl_3$

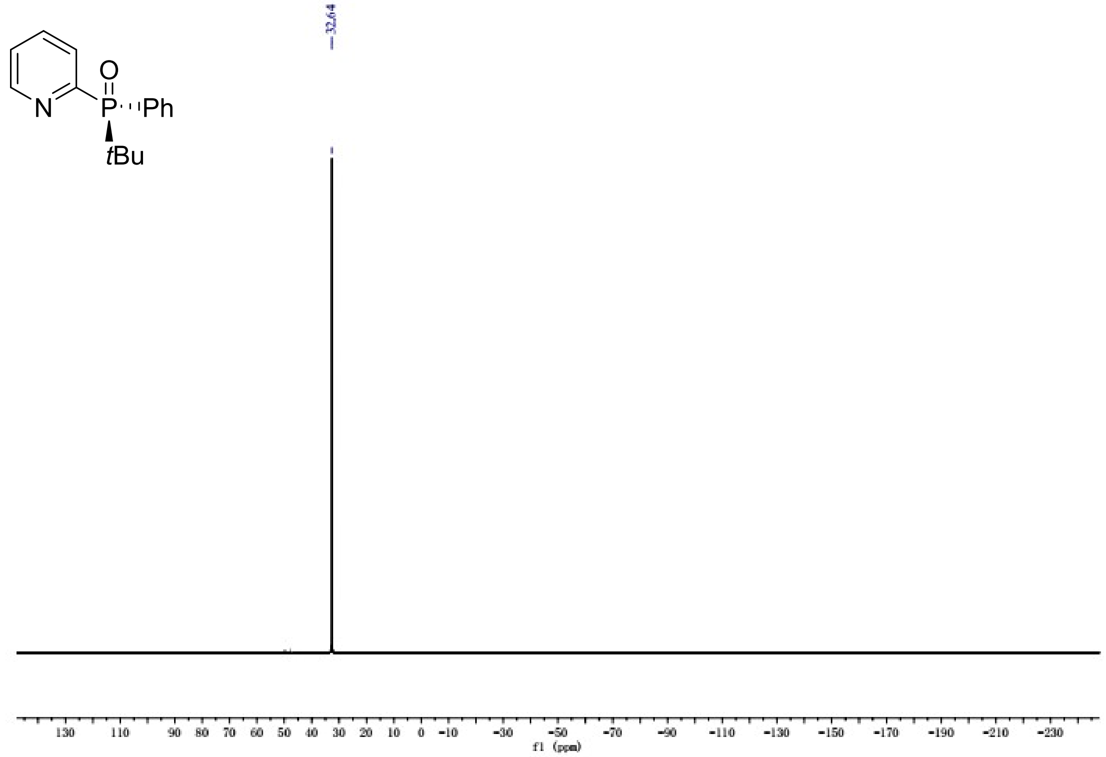

Figure S5. ^{31}P NMR spectrum of **4** in $CDCl_3$

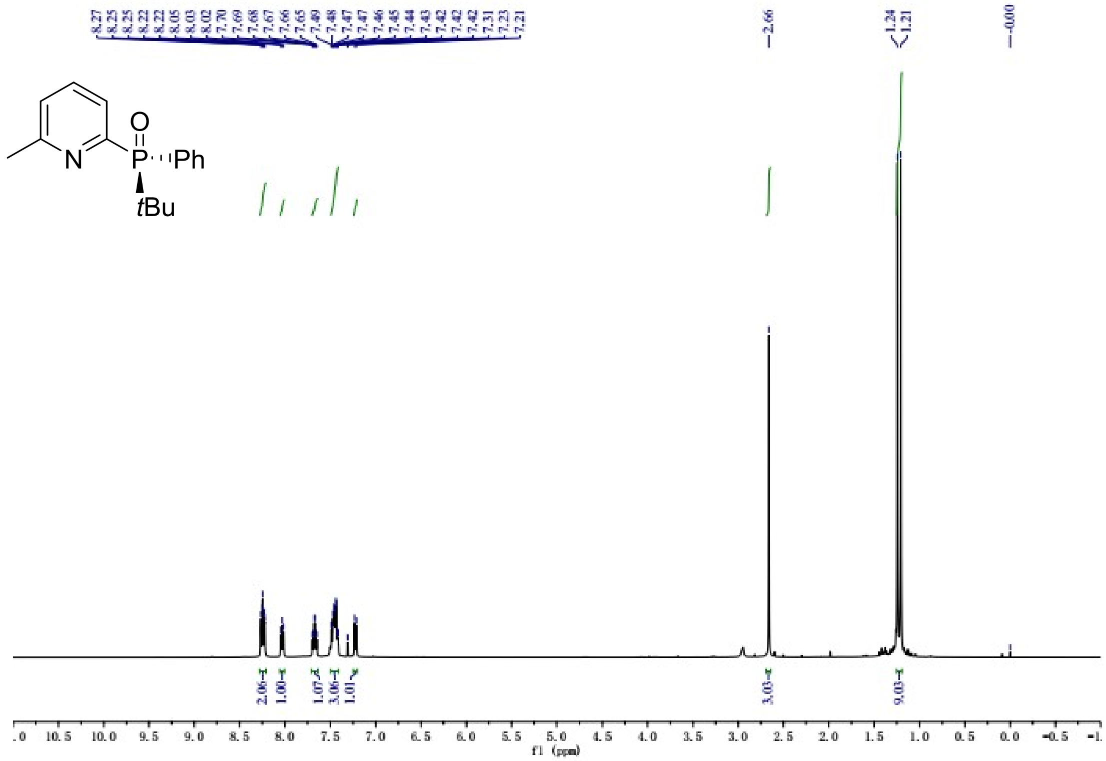

Figure S6. ^{1}H NMR spectrum of **5** in $CDCl_3$

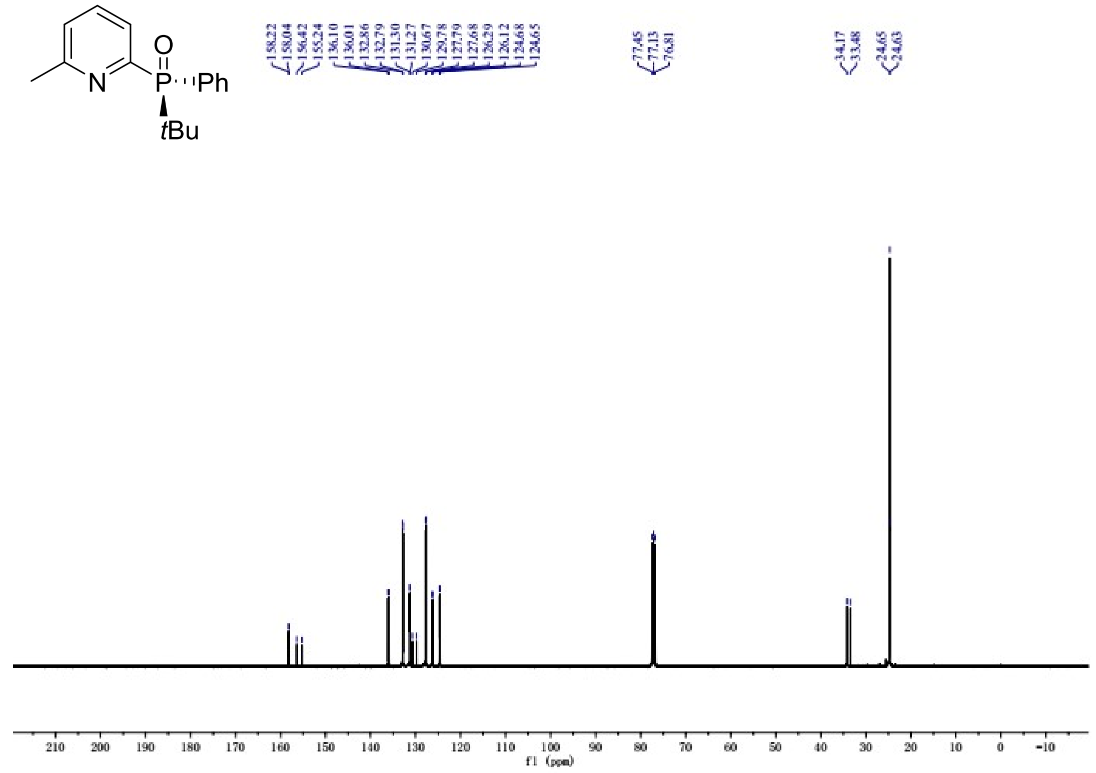

Figure S7. ^{13}C NMR spectrum of **5** in $CDCl_3$

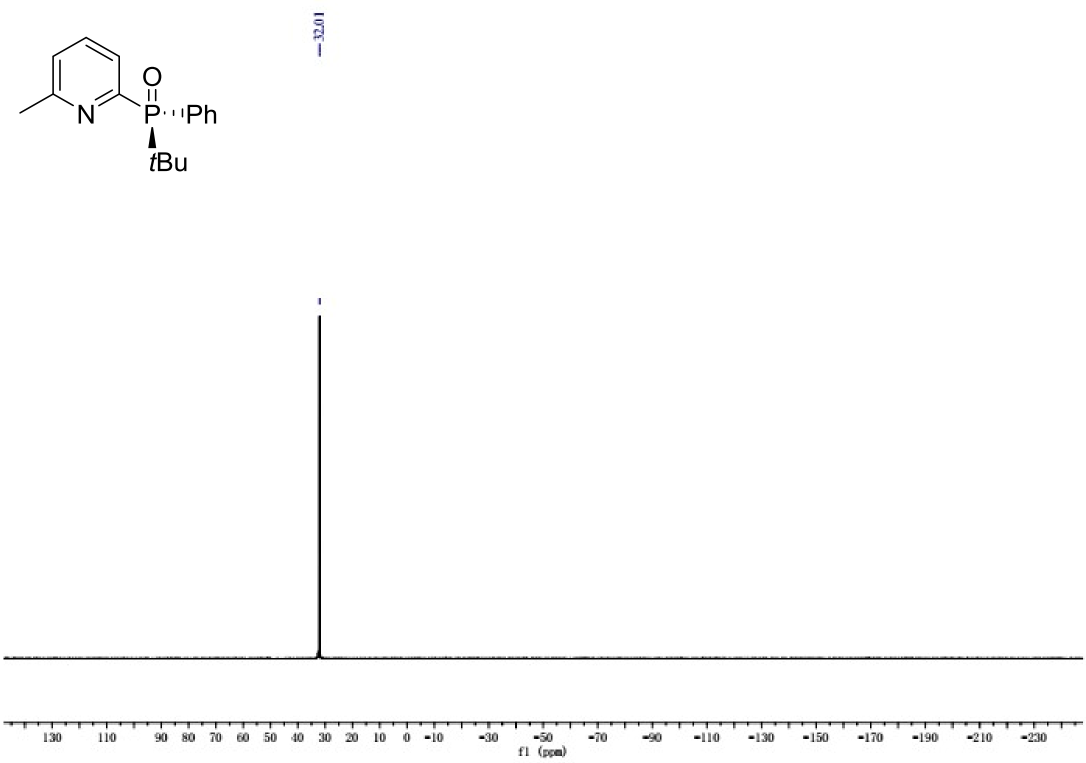

Figure S8. ^{31}P NMR spectrum of **5** in $CDCl_3$

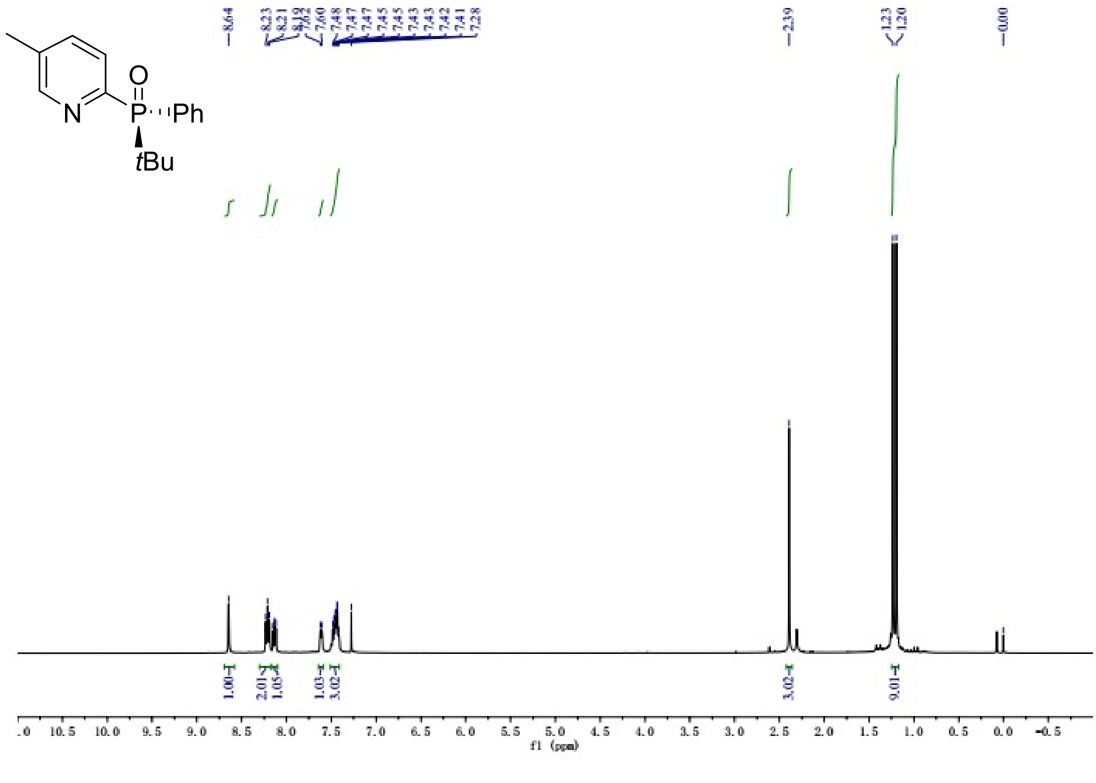

Figure S9. ^{1}H NMR spectrum of **6** in $CDCl_3$

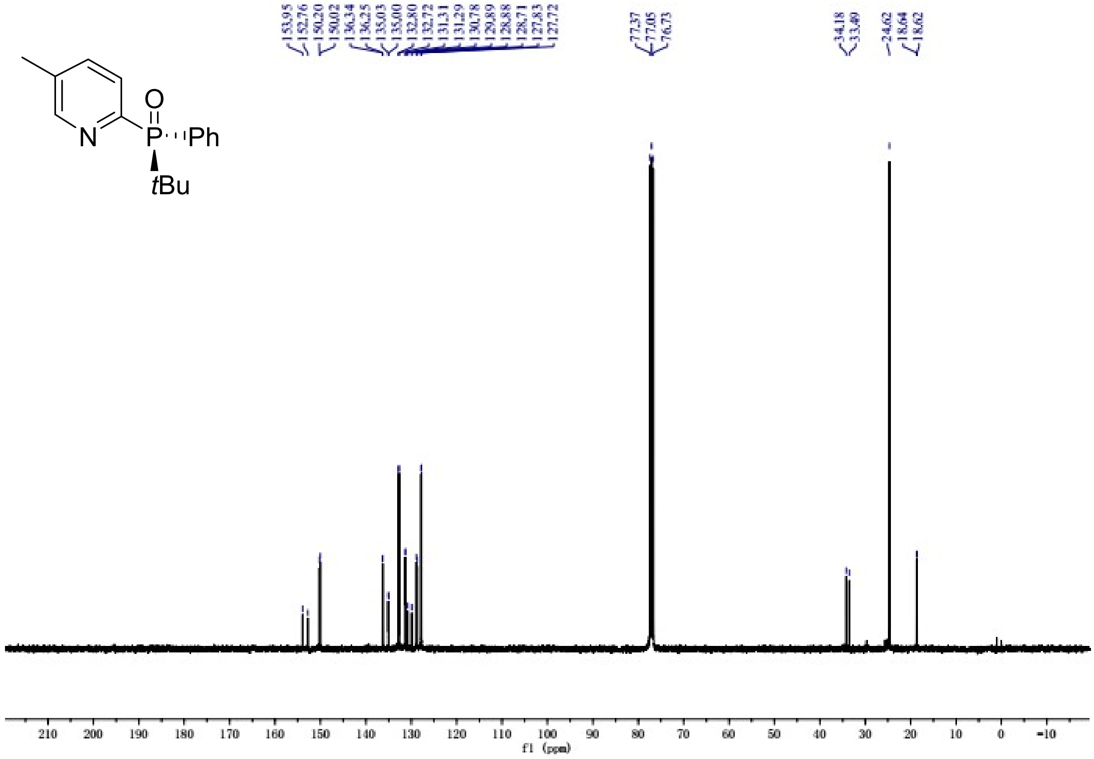

Figure S10. ^{13}C NMR spectrum of **6** in $CDCl_3$

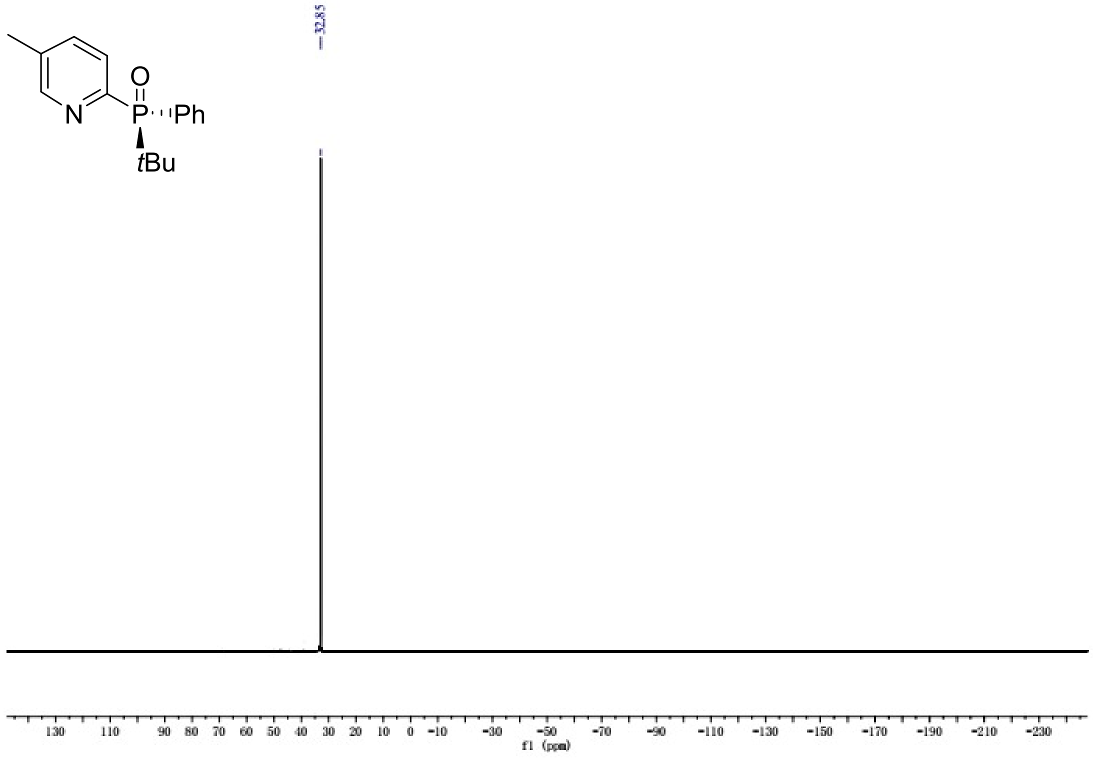

Figure S11. ^{31}P NMR spectrum of **6** in $CDCl_3$

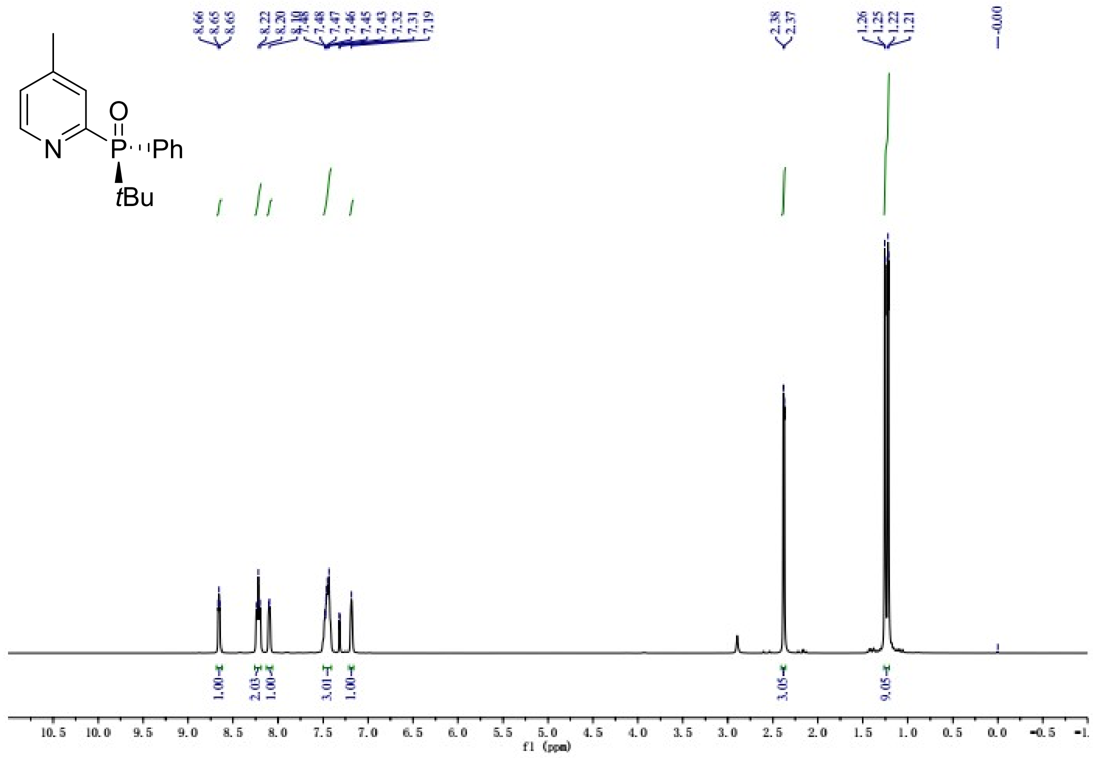

Figure S12. ^{1}H NMR spectrum of **7** in $CDCl_3$

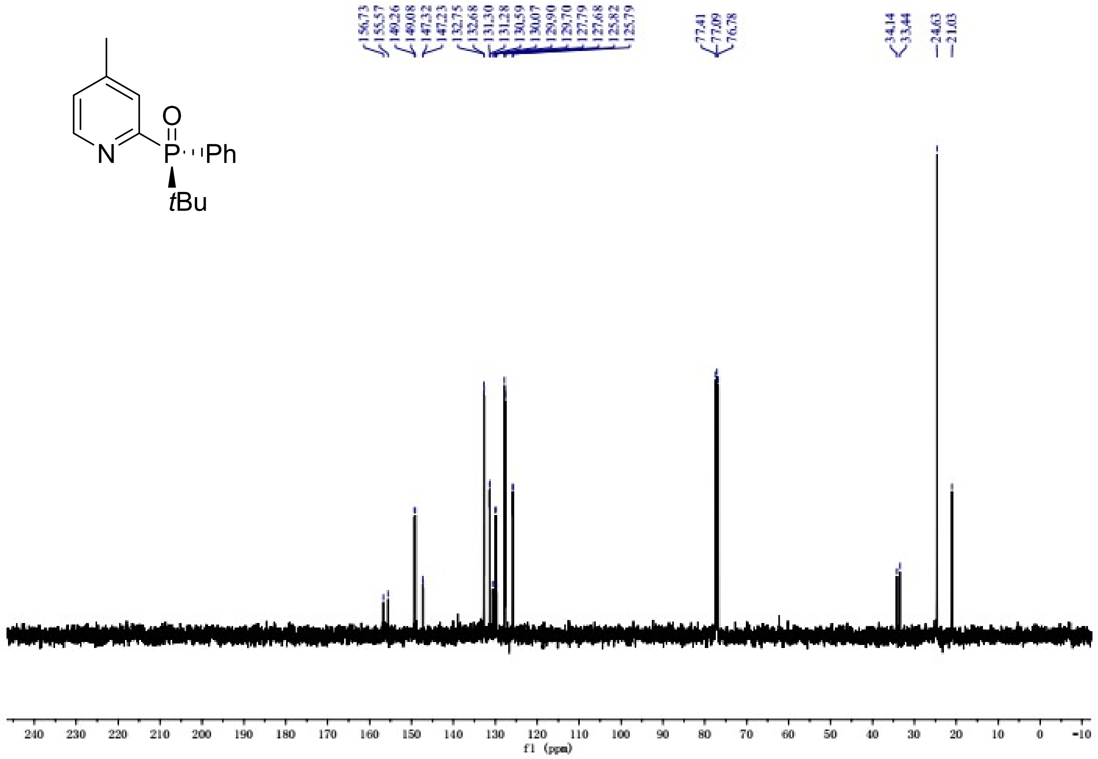

Figure S13. ^{13}C NMR spectrum of **7** in $CDCl_3$

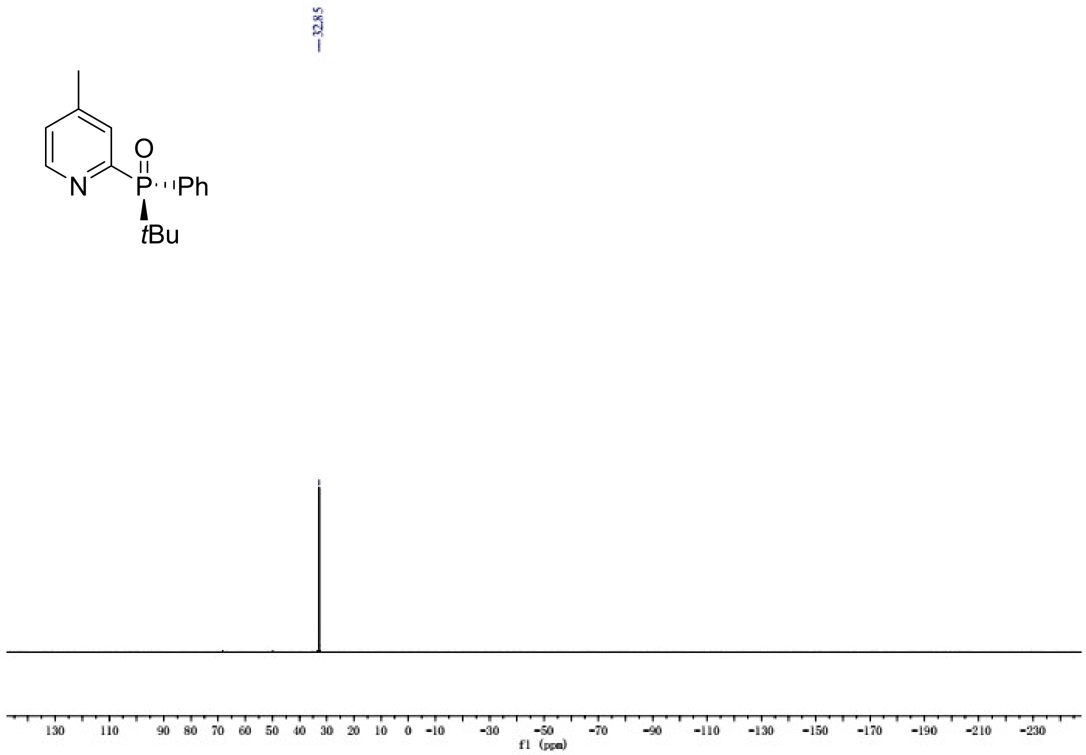

Figure S14. ^{31}P NMR spectrum of **7** in $CDCl_3$

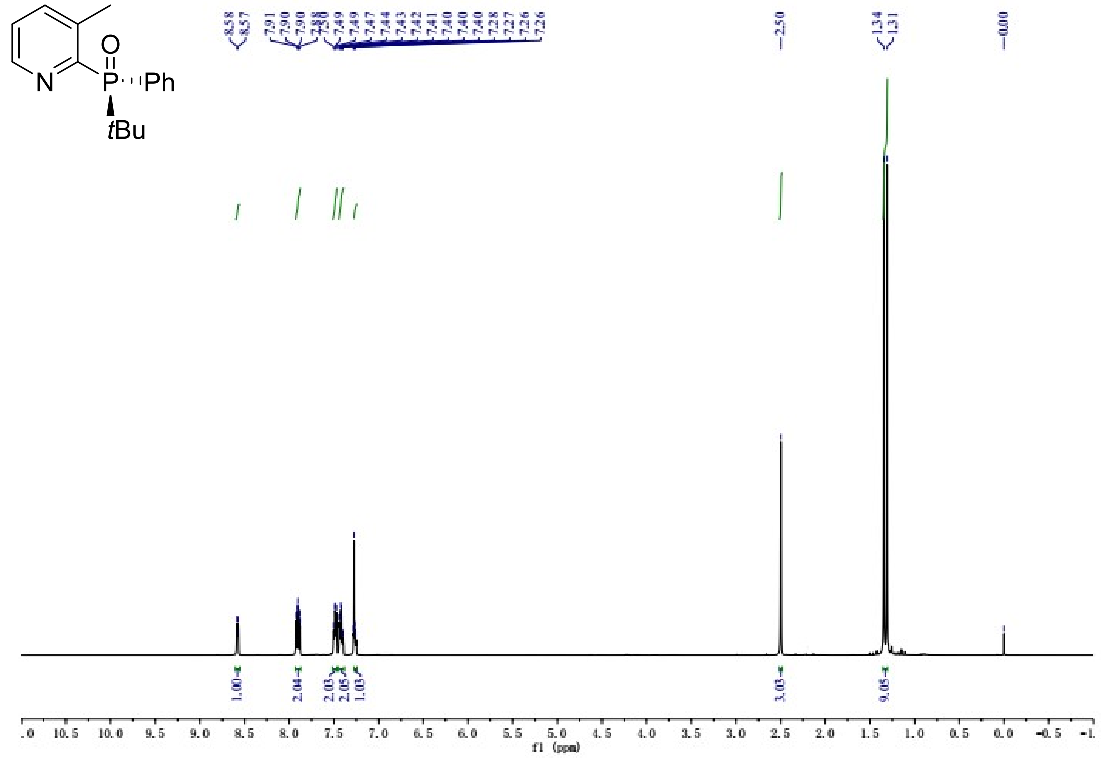

Figure S15. ^{1}H NMR spectrum of **8** in $CDCl_3$

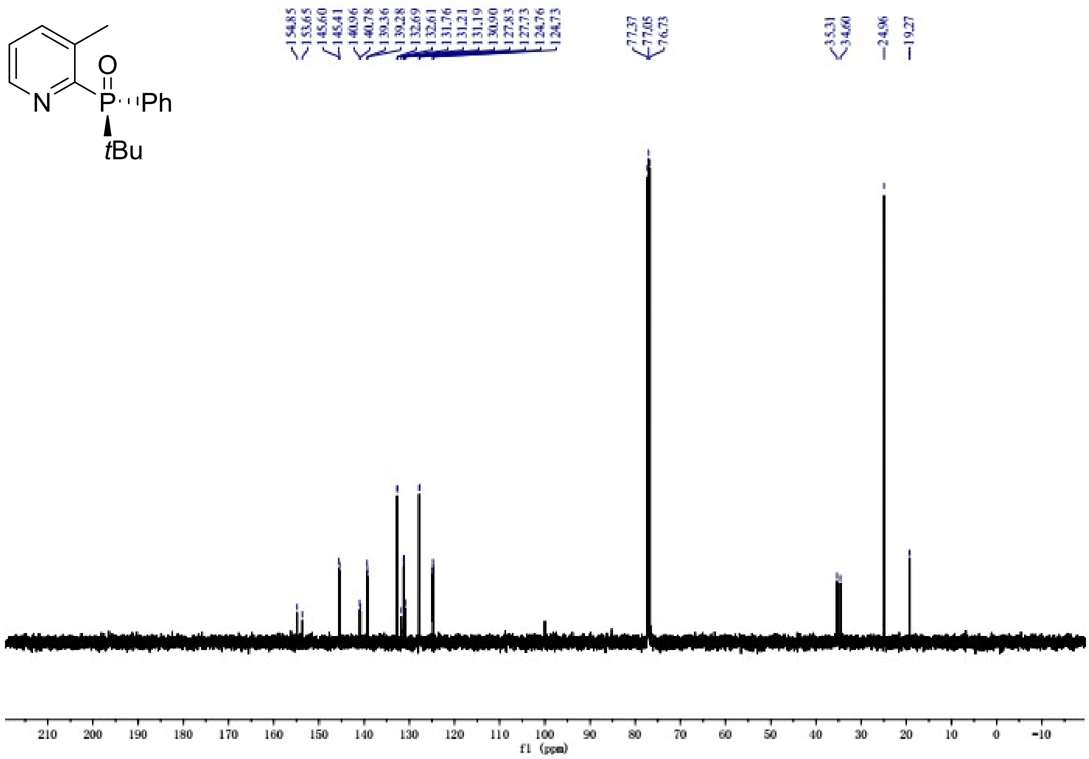

Figure S16. ^{13}C NMR spectrum of **8** in $CDCl_3$

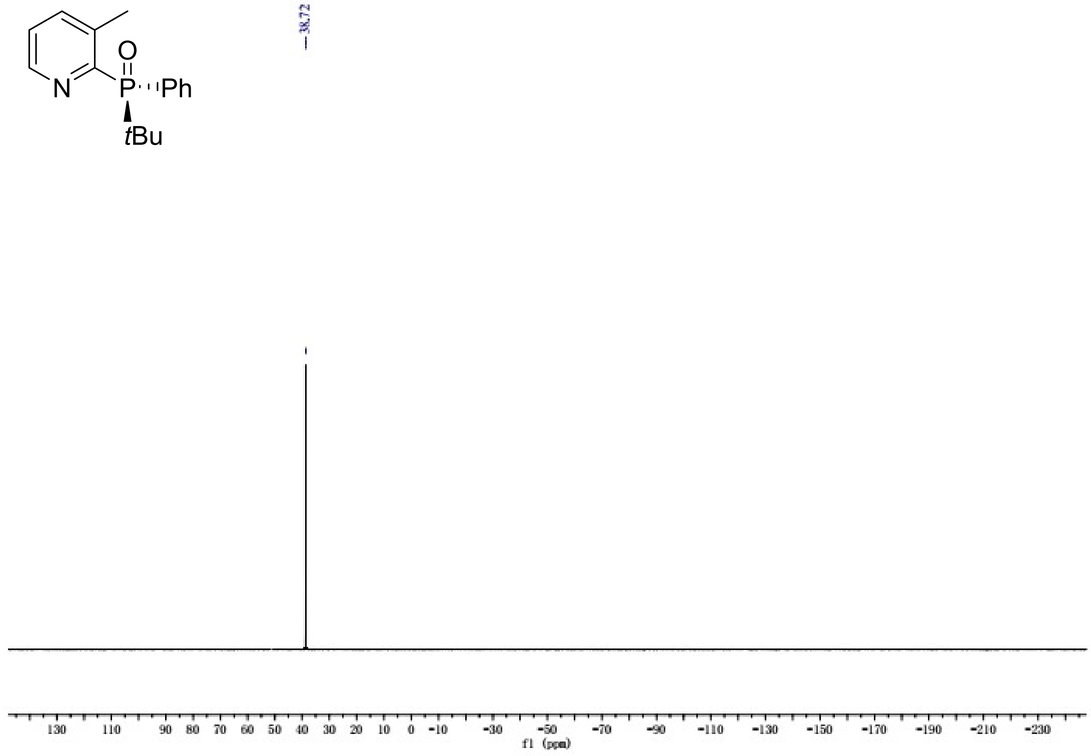

Figure S17. ^{31}P NMR spectrum of **8** in $CDCl_3$

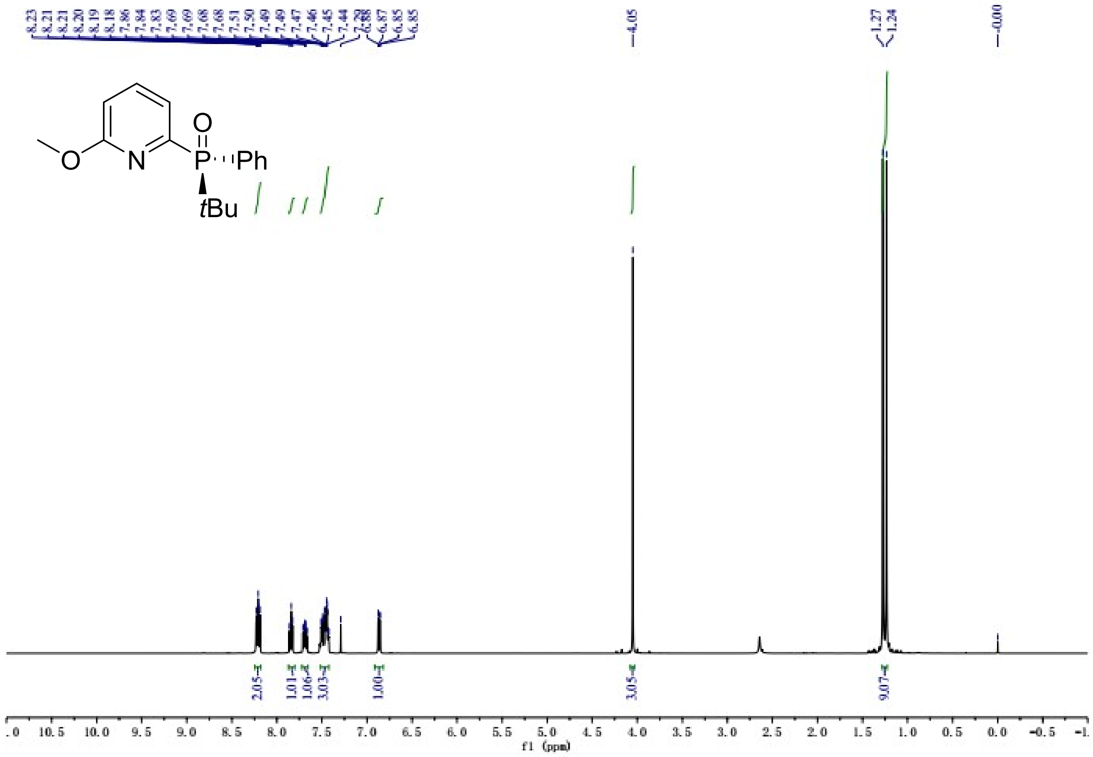

Figure S18. ^{1}H NMR spectrum of **9** in $CDCl_3$

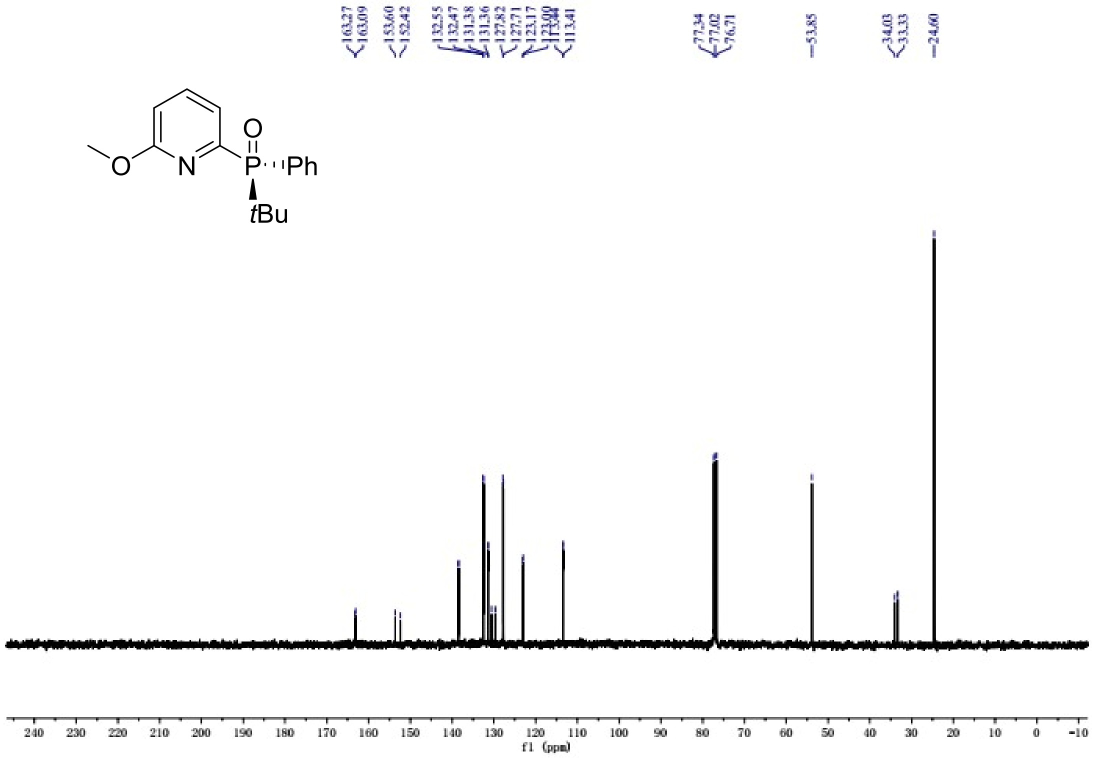

Figure S19. ^{13}C NMR spectrum of **9** in $CDCl_3$

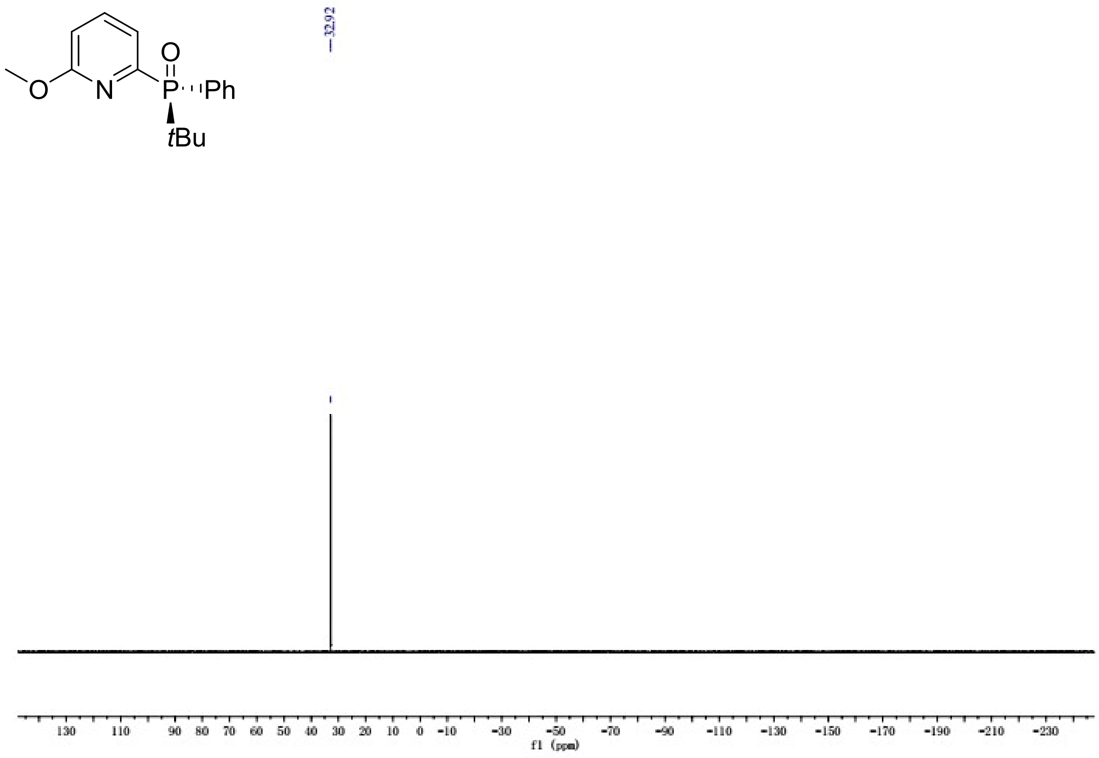

Figure S20. ^{31}P NMR spectrum of **9** in $CDCl_3$

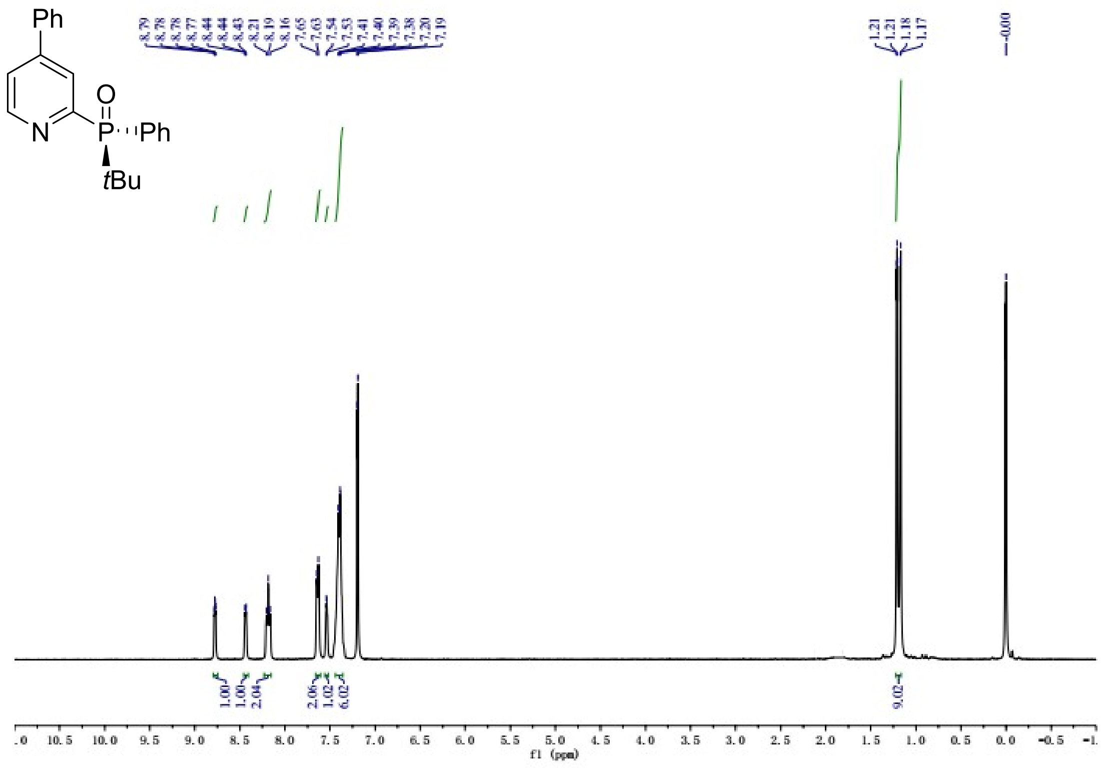

Figure S21. ^{1}H NMR spectrum of **12** in $CDCl_3$

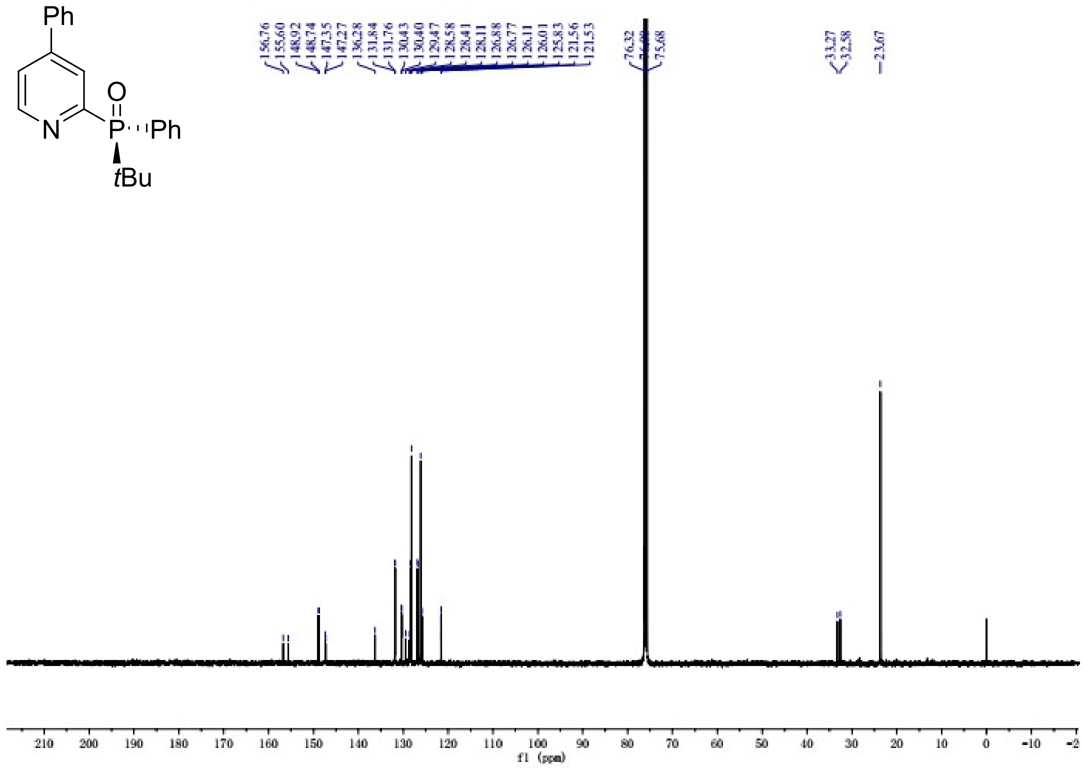

Figure S22. ^{13}C NMR spectrum of **12** in $CDCl_3$

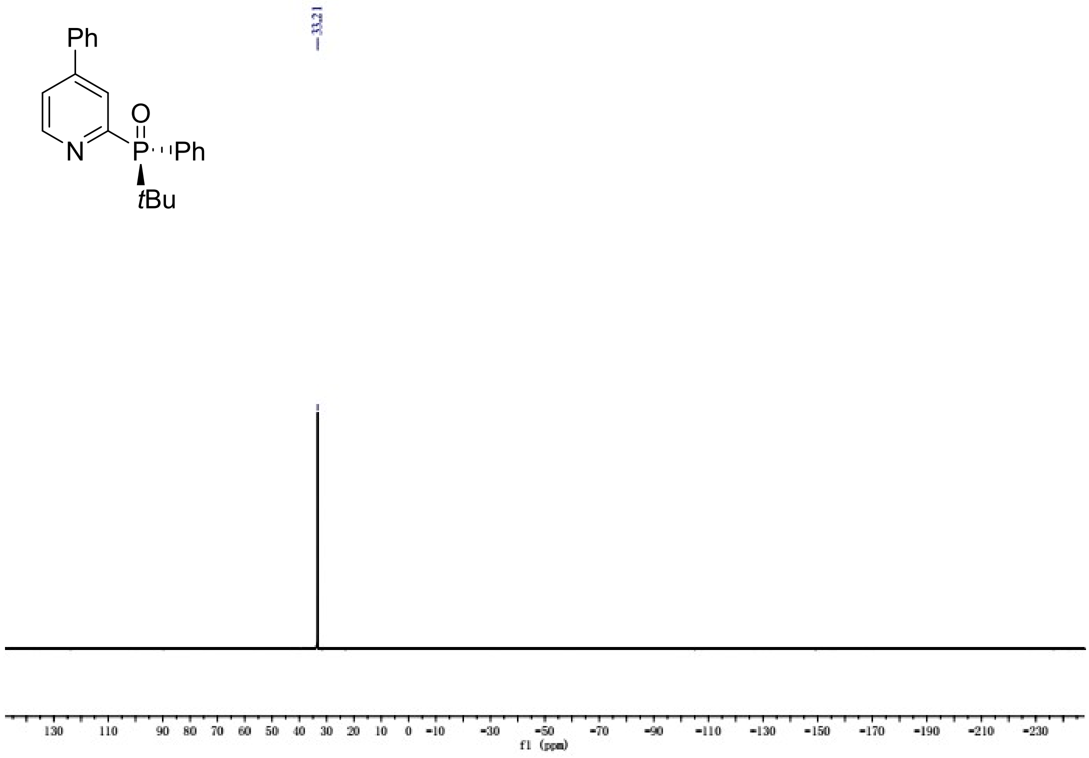

Figure S23. ^{31}P NMR spectrum of **12** in $CDCl_3$

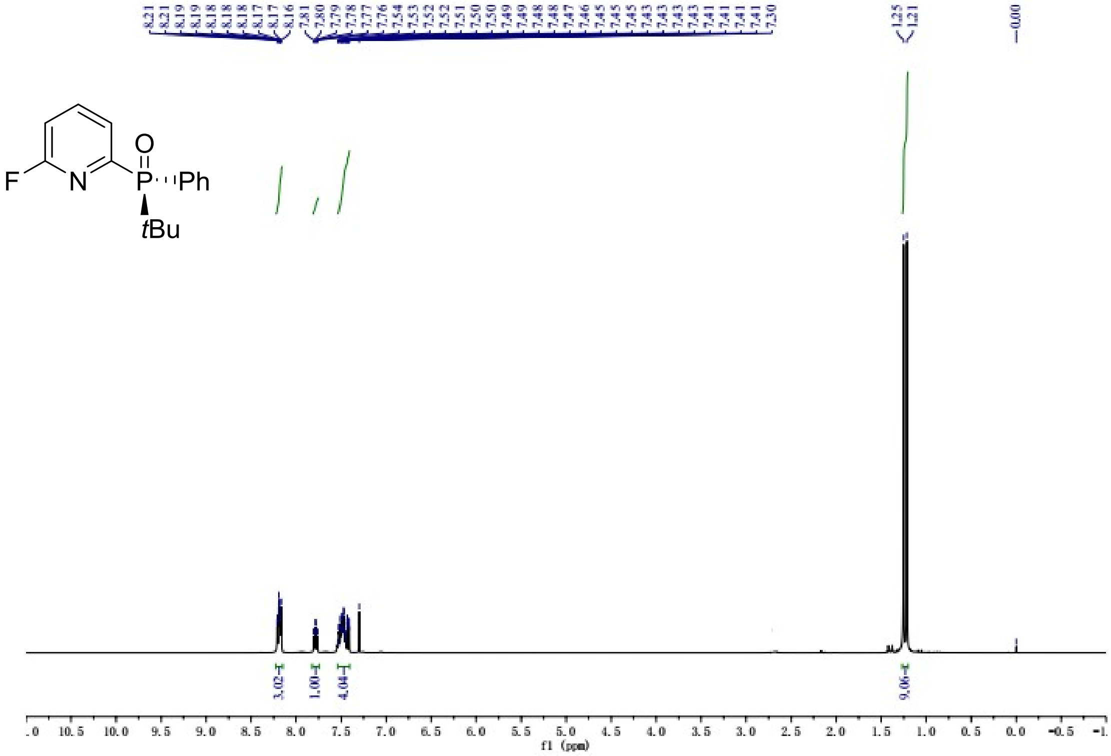

Figure S24. ^{1}H NMR spectrum of **13** in $CDCl_3$

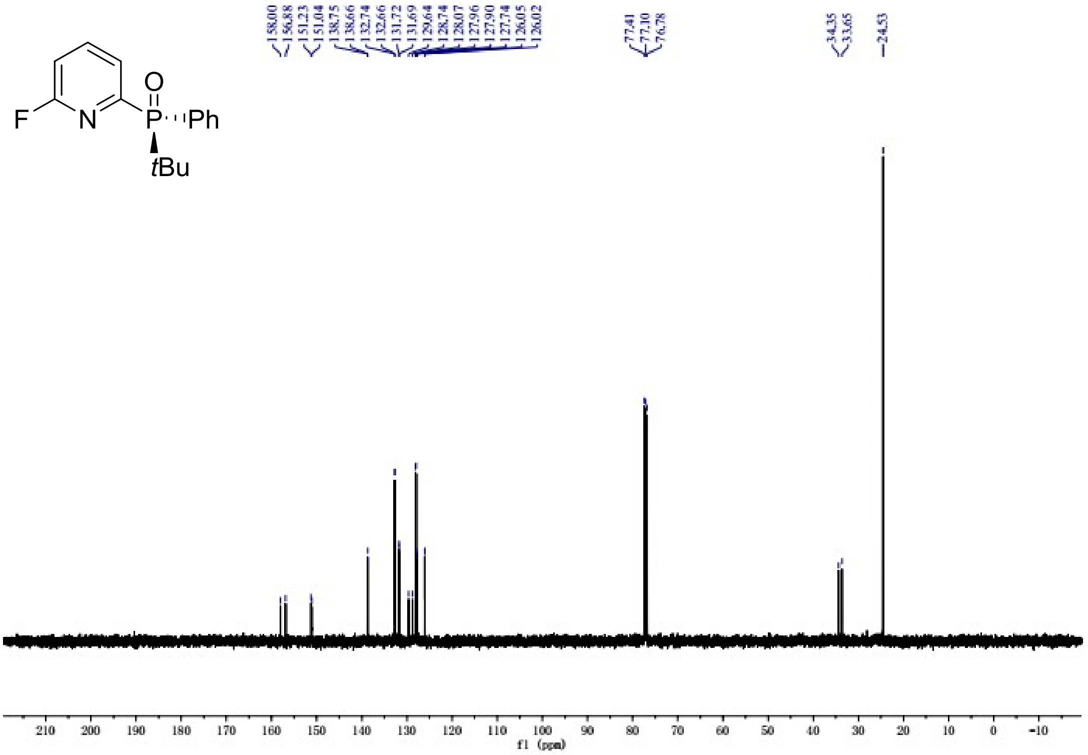

Figure S25. ^{13}C NMR spectrum of **13** in $CDCl_3$

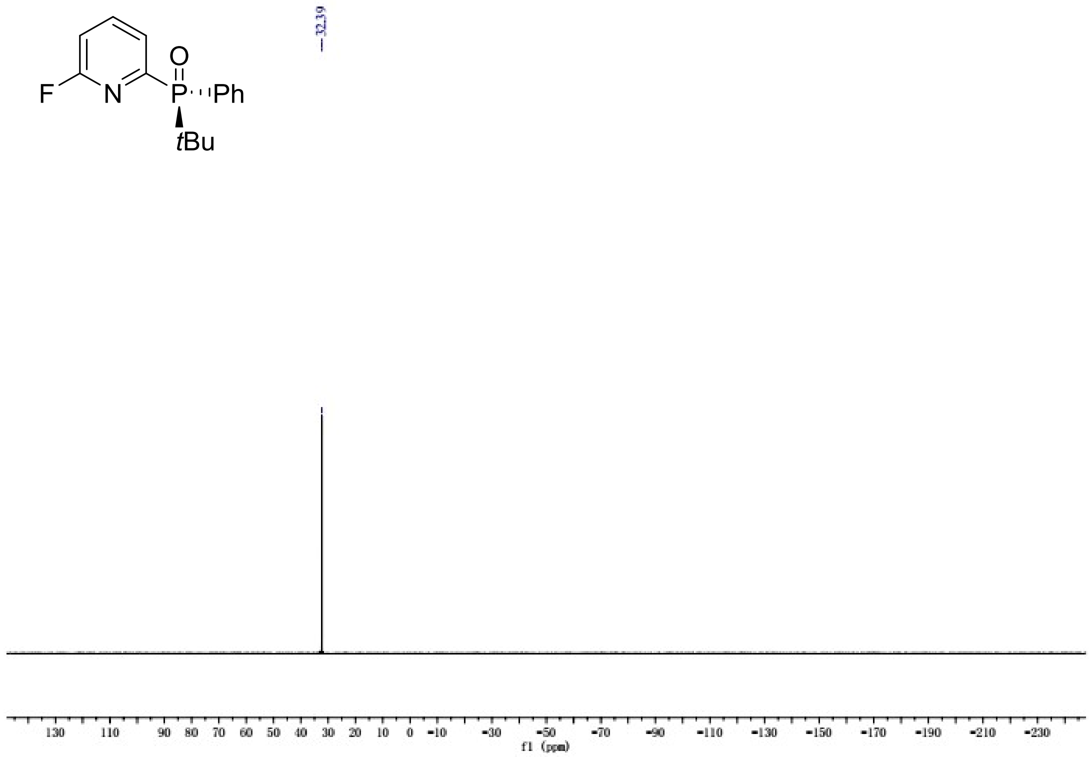

Figure S26. ^{31}P NMR spectrum of **13** in $CDCl_3$

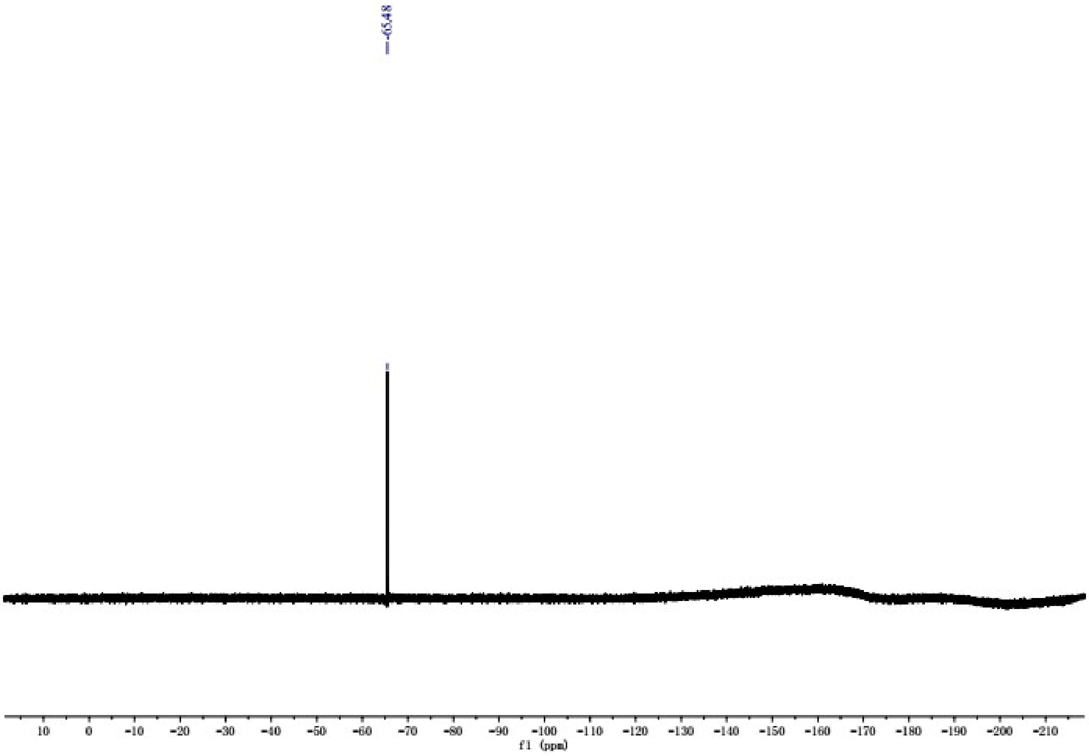

Figure S27. ^{19}F NMR spectrum of **13** in $CDCl_3$

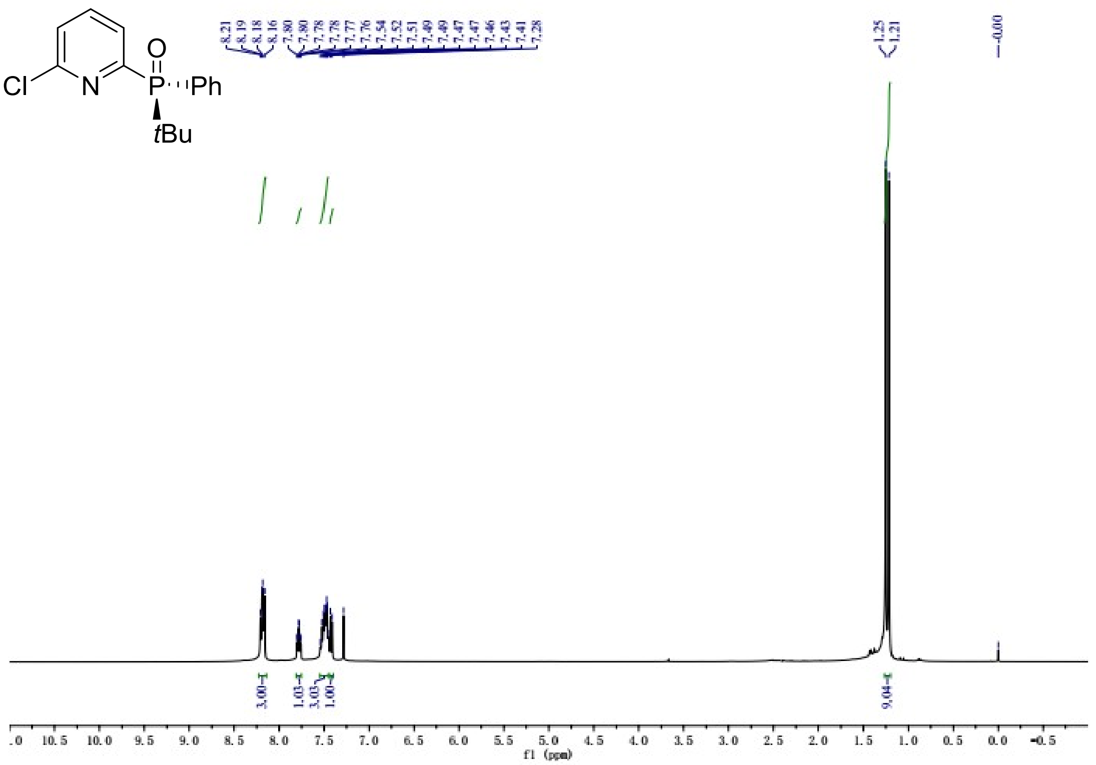

Figure S28. ^{1}H NMR spectrum of **14** in $CDCl_3$

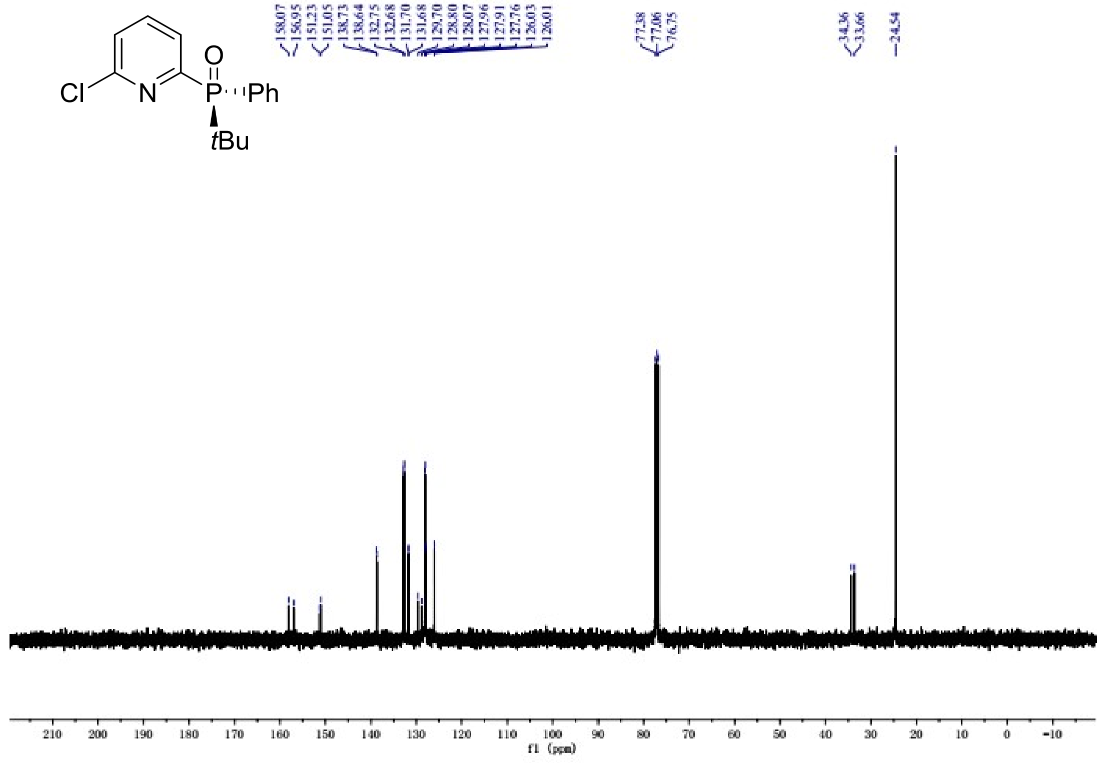

Figure S29. ^{13}C NMR spectrum of **14** in $CDCl_3$

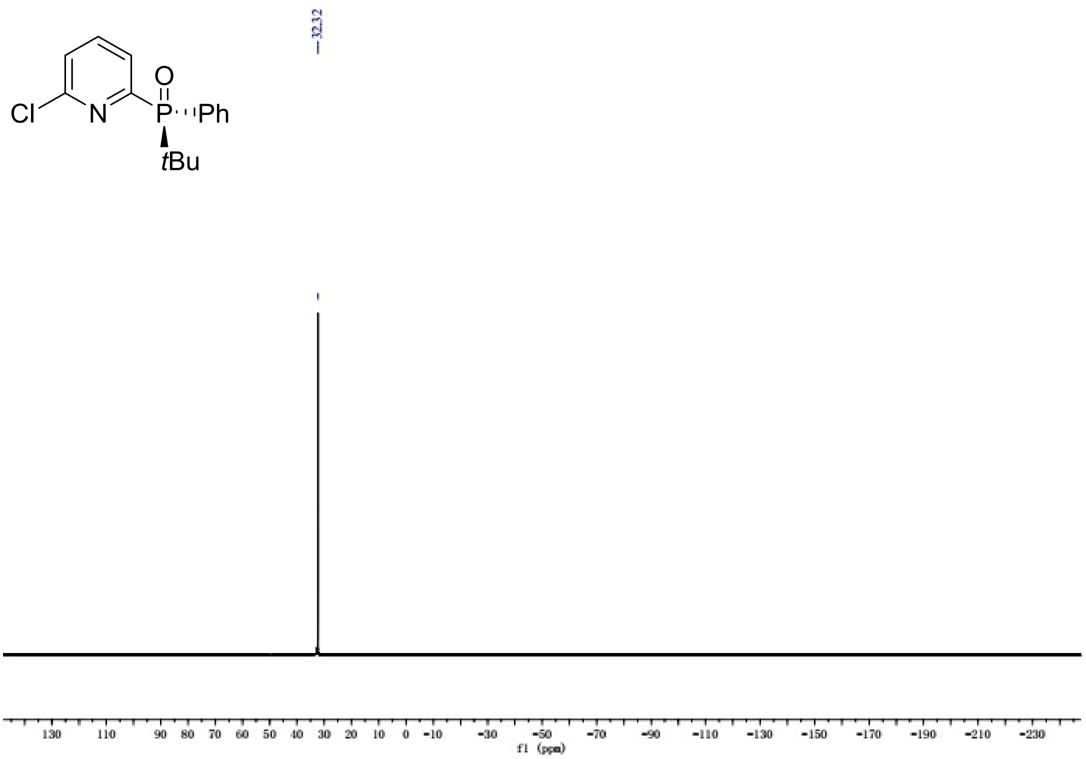

Figure S30. ^{31}P NMR spectrum of **14** in $CDCl_3$

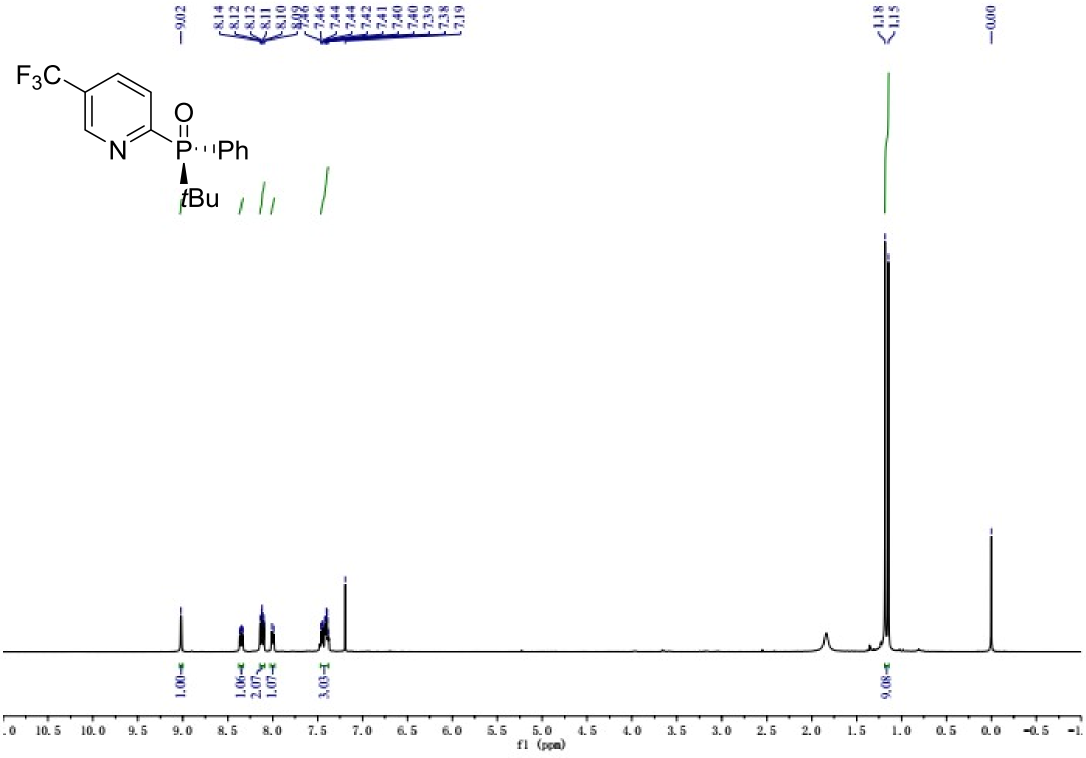

Figure S31. ^{1}H NMR spectrum of **15** in $CDCl_3$

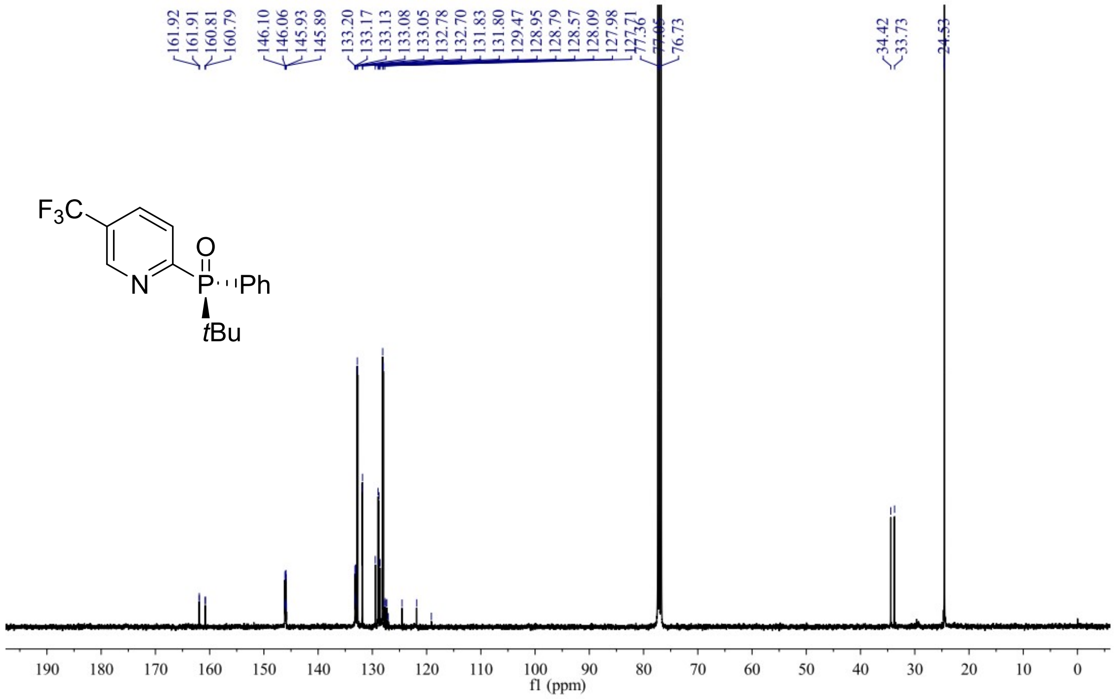

Figure S32. ^{13}C NMR spectrum of **15** in $CDCl_3$

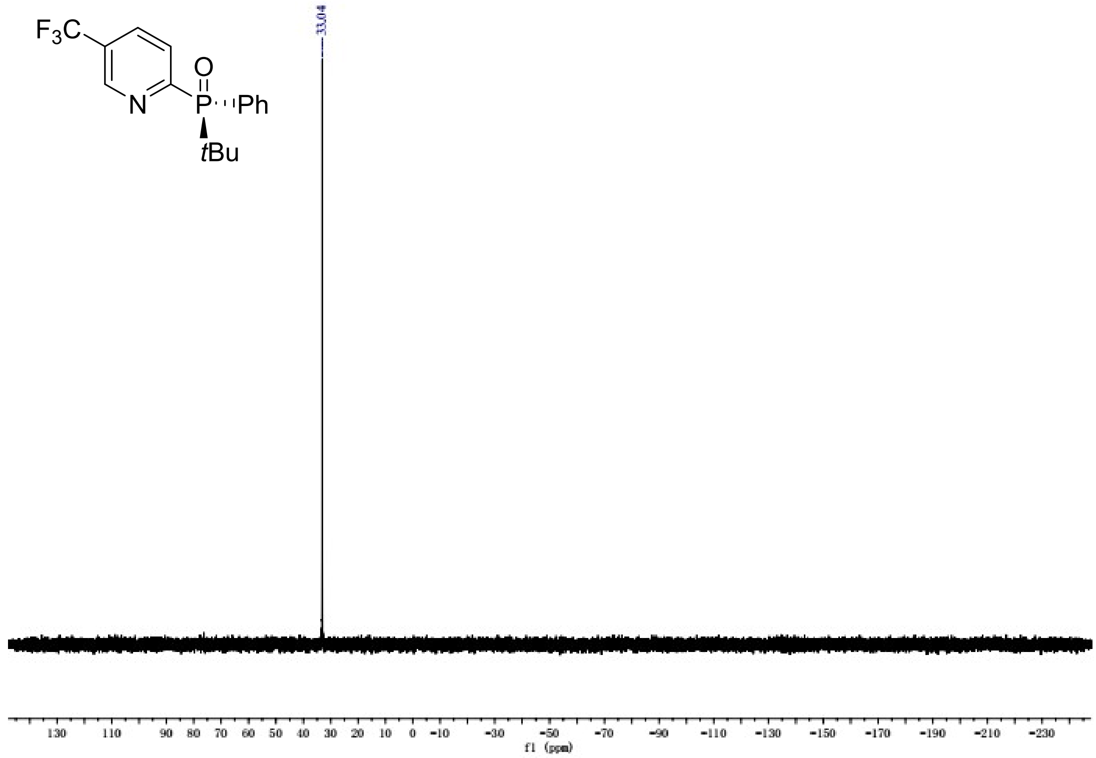

Figure S33. ^{31}P NMR spectrum of **15** in $CDCl_3$

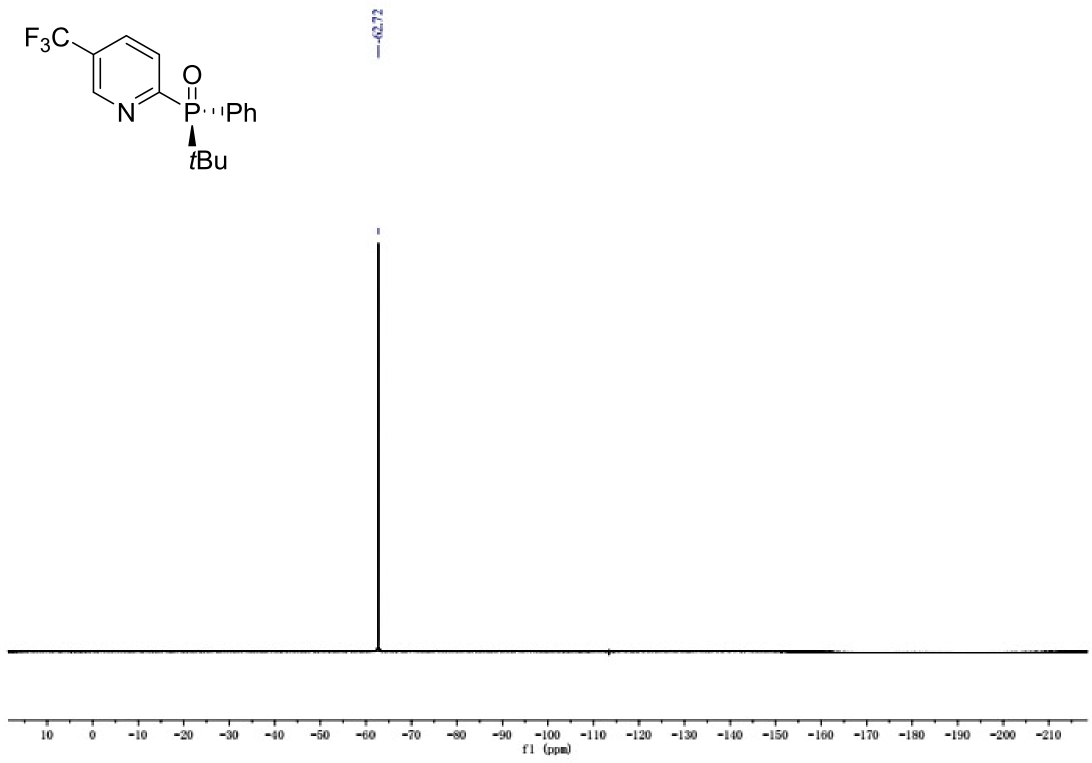

Figure S34. ^{19}F NMR spectrum of **15** in $CDCl_3$

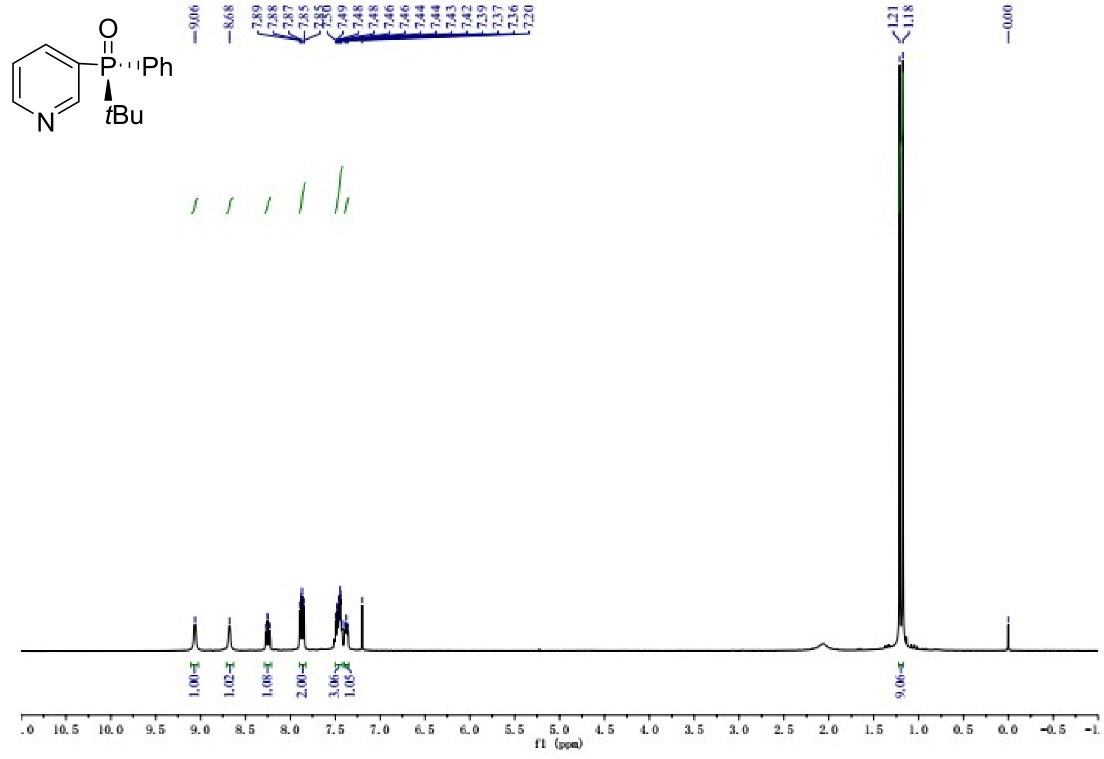

Figure S35. ^{1}H NMR spectrum of **16** in $CDCl_3$

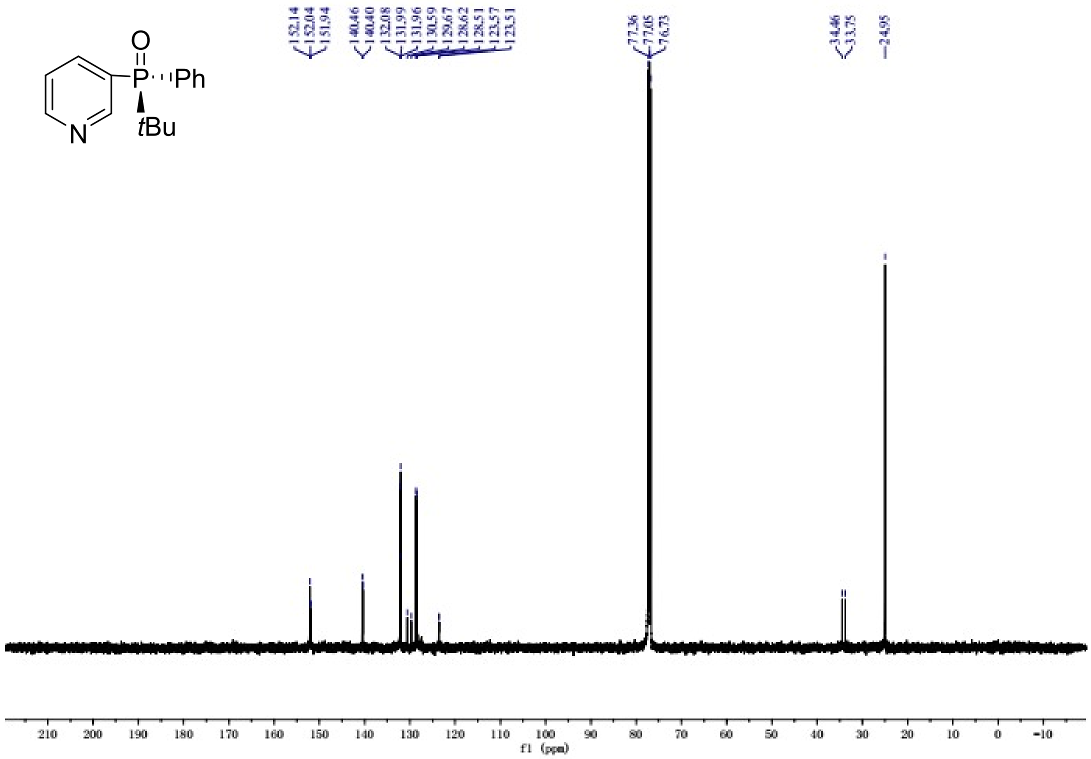

Figure S36. ^{13}C NMR spectrum of **16** in $CDCl_3$

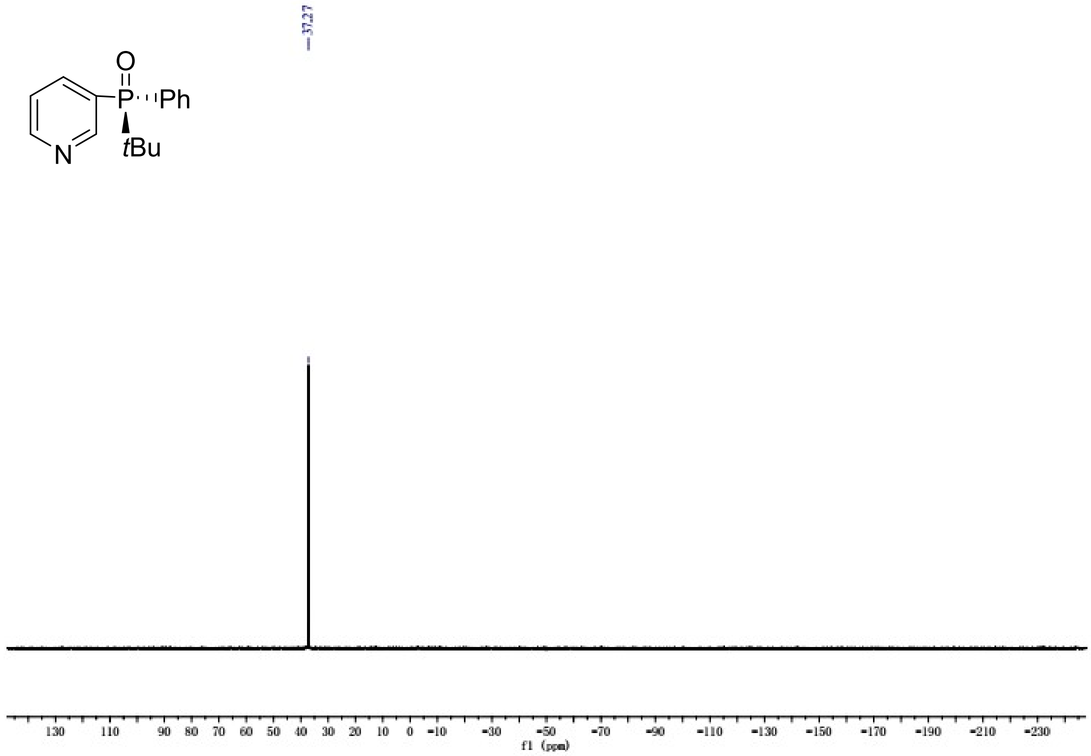

Figure S37. ^{31}P NMR spectrum of **16** in $CDCl_3$

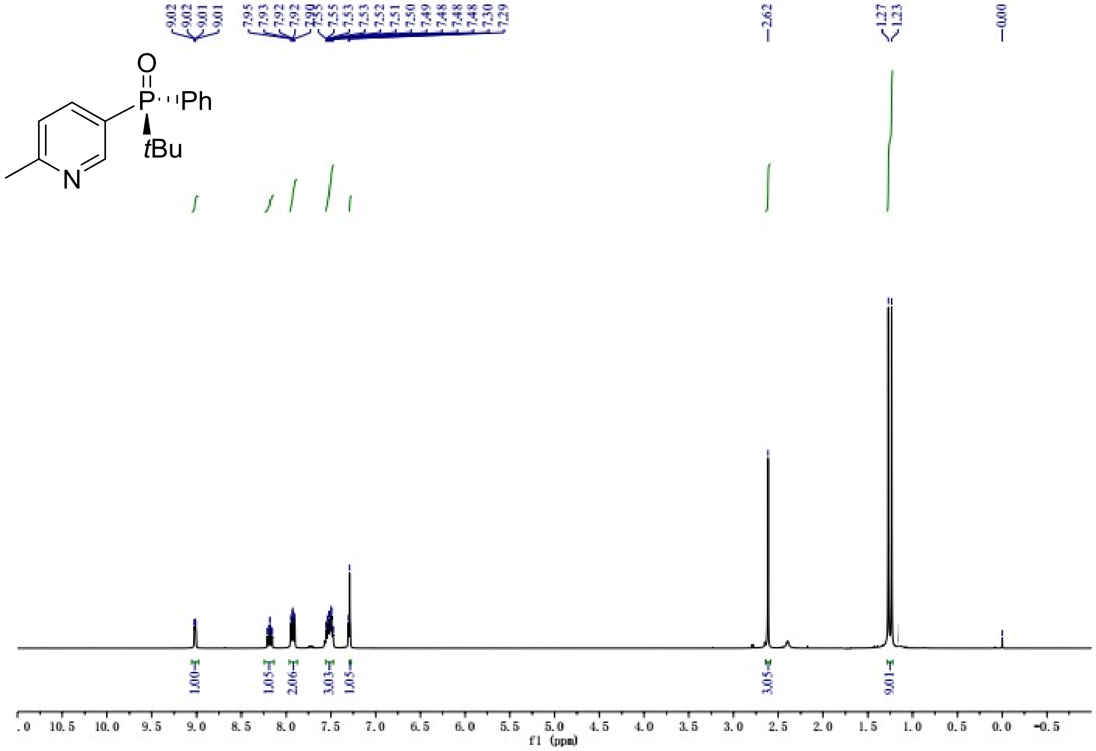

Figure S38. ^{1}H NMR spectrum of **17** in $CDCl_3$

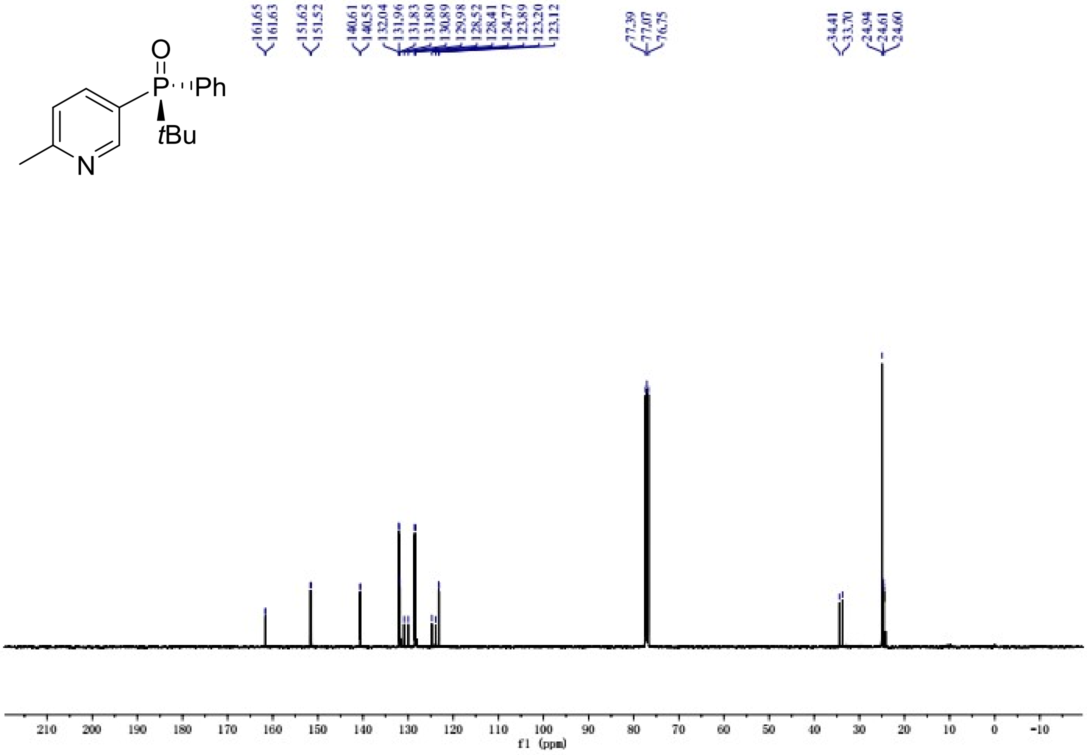

Figure S39. ^{13}C NMR spectrum of **17** in $CDCl_3$

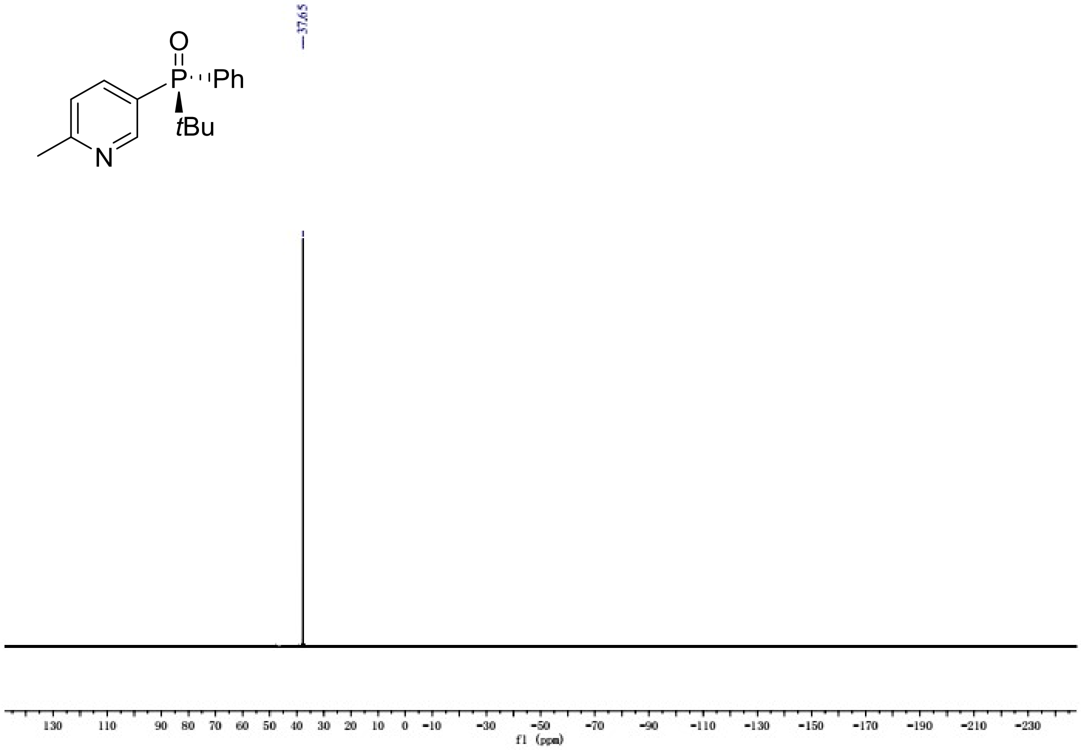

Figure S40. ^{31}P NMR spectrum of **17** in $CDCl_3$

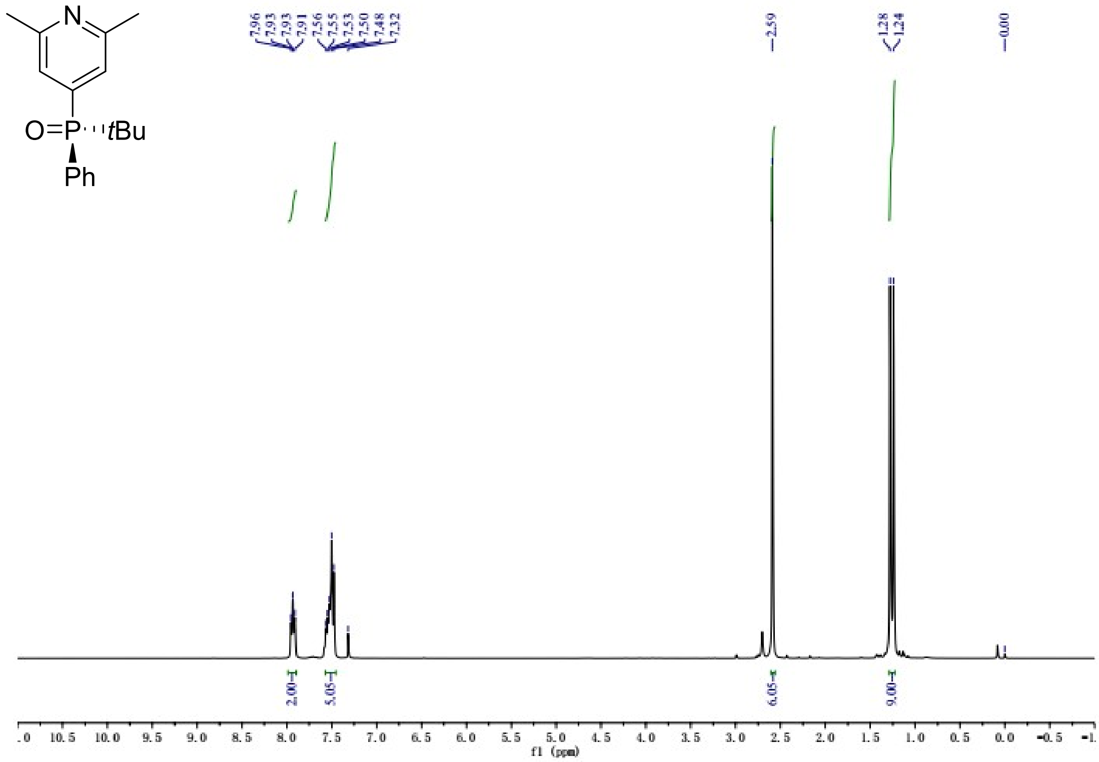

Figure S41. ^{1}H NMR spectrum of **18** in $CDCl_3$

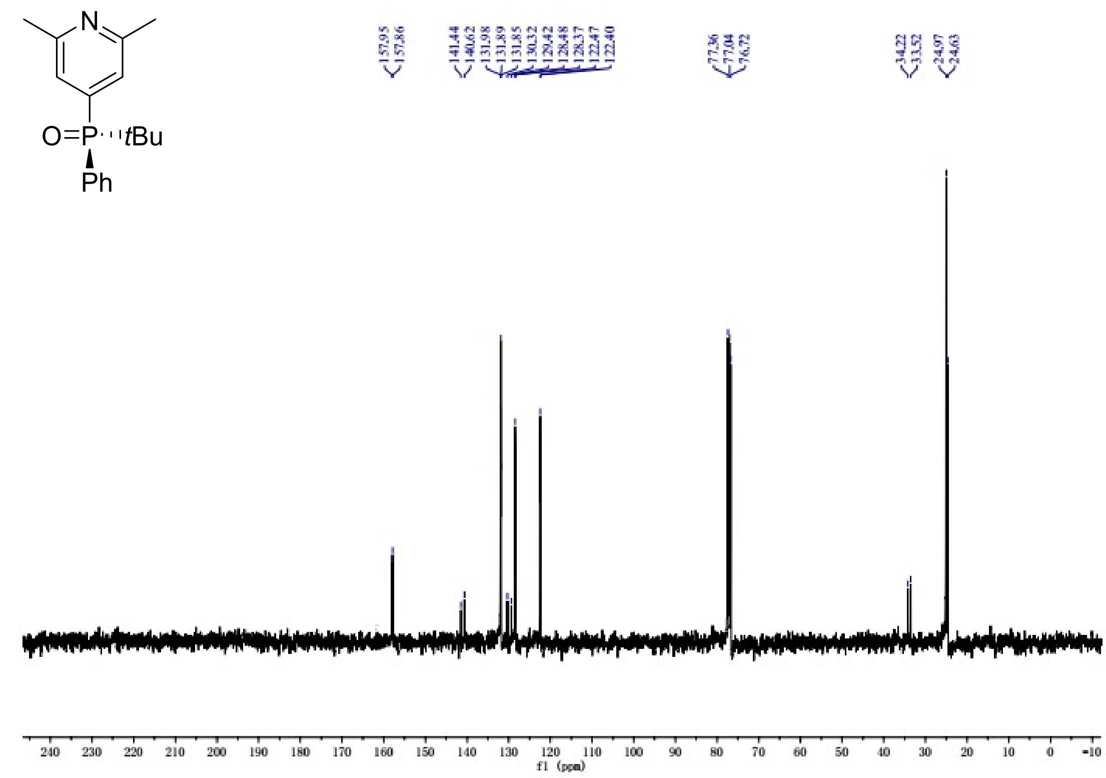

Figure S42. ^{13}C NMR spectrum of **18** in $CDCl_3$

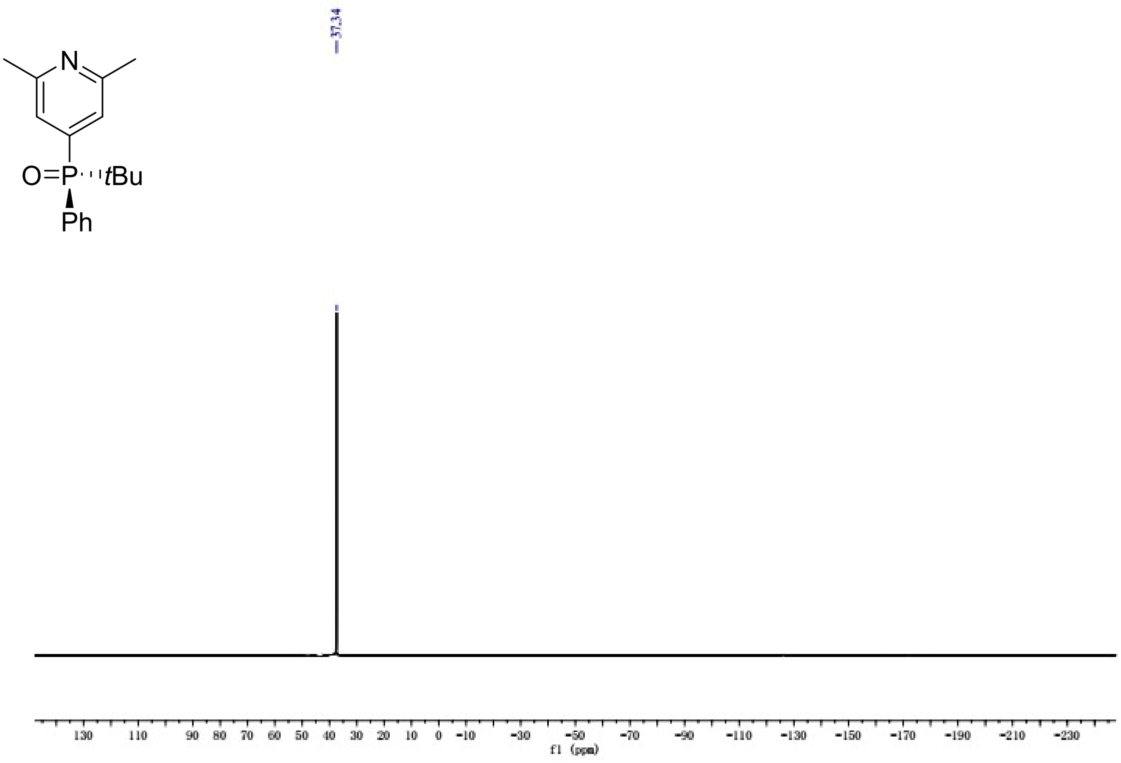

Figure S43. ^{31}P NMR spectrum of **18** in $CDCl_3$

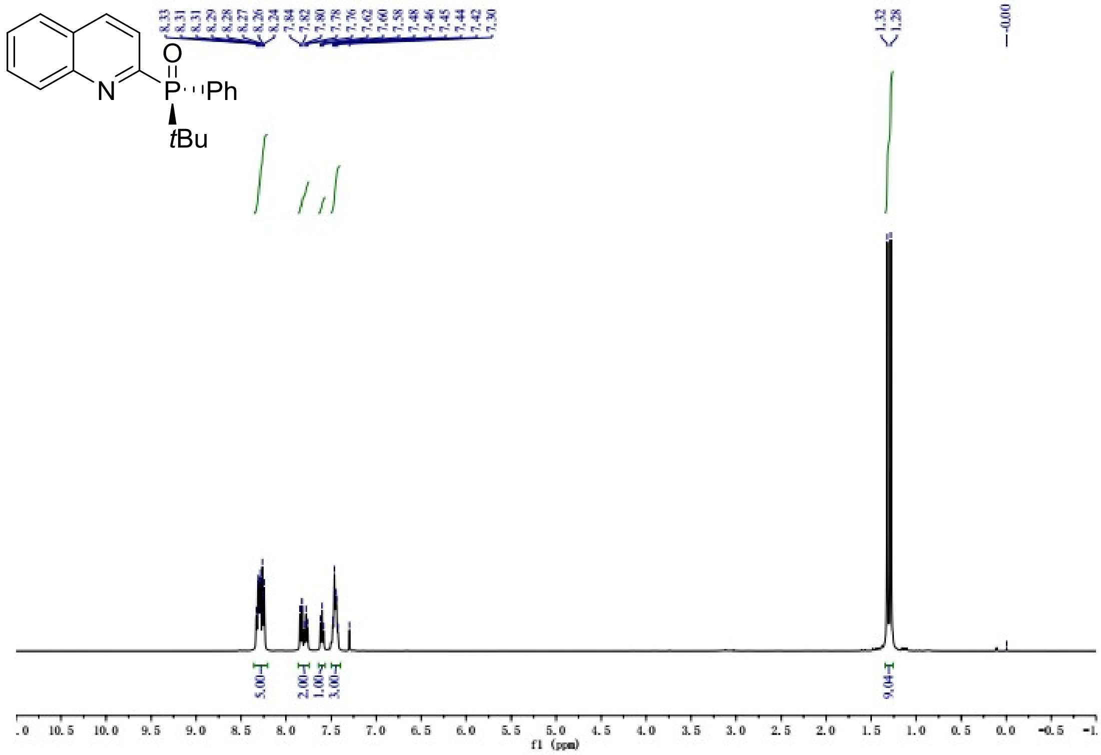

Figure S44. ^{1}H NMR spectrum of **19** in $CDCl_3$

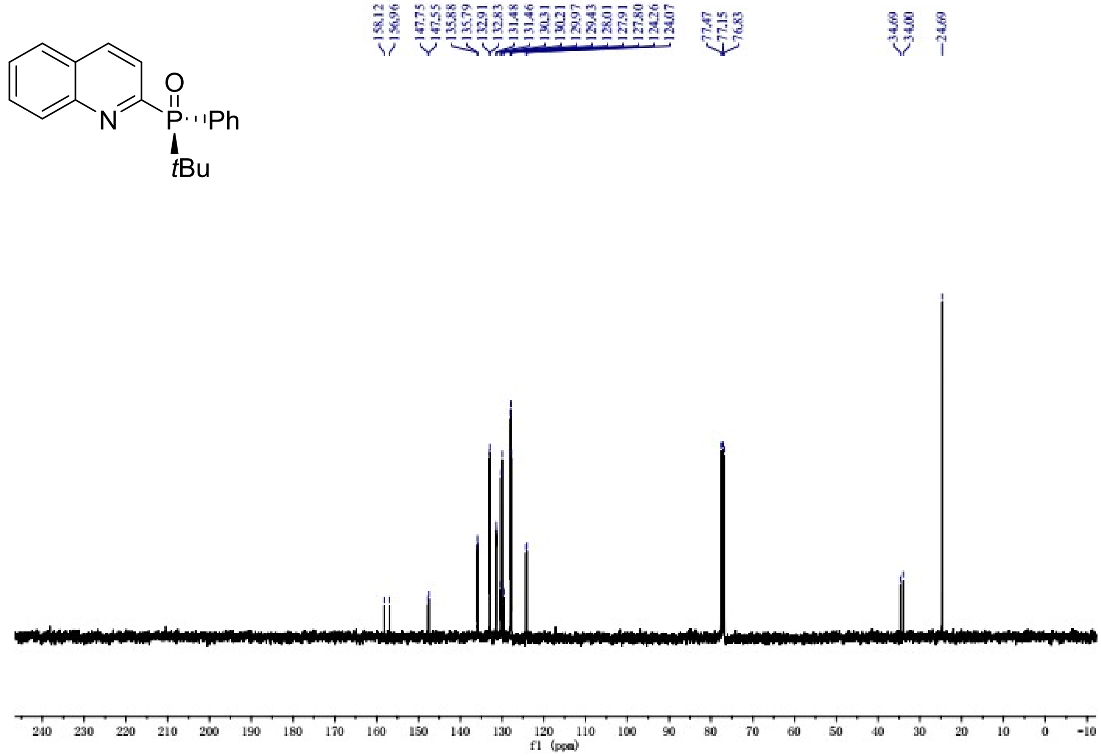

Figure S45. ^{13}C NMR spectrum of **19** in $CDCl_3$

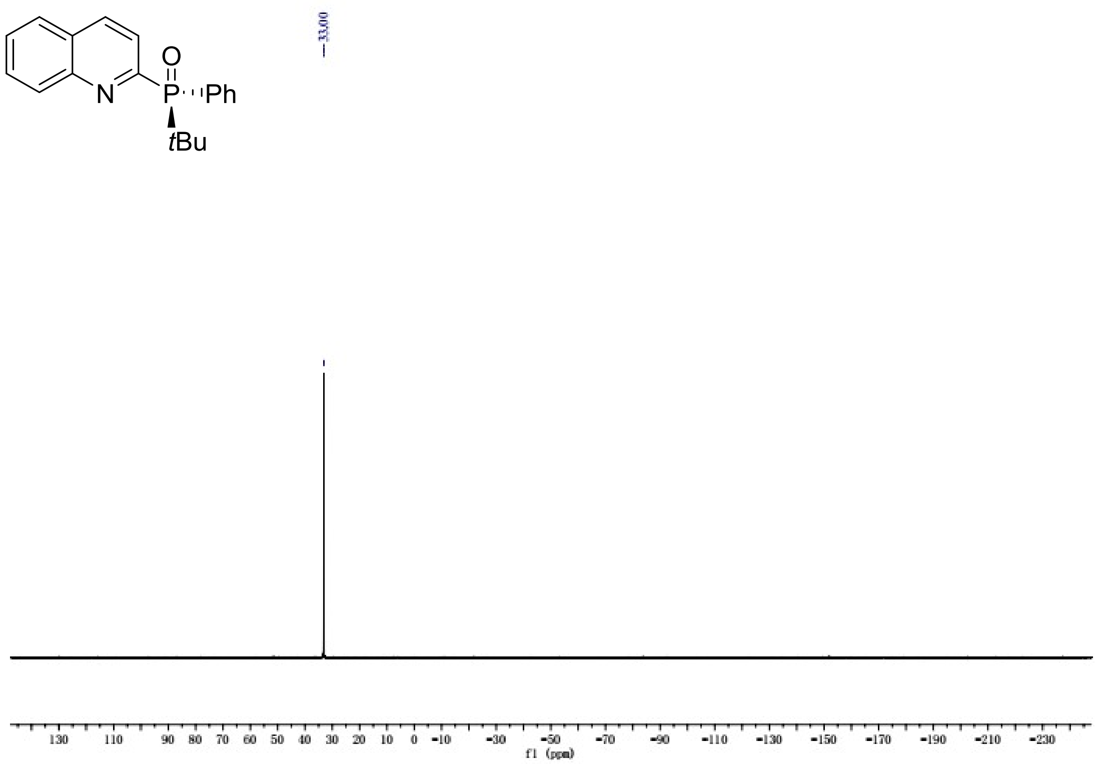

Figure S46. ^{31}P NMR spectrum of **19** in $CDCl_3$

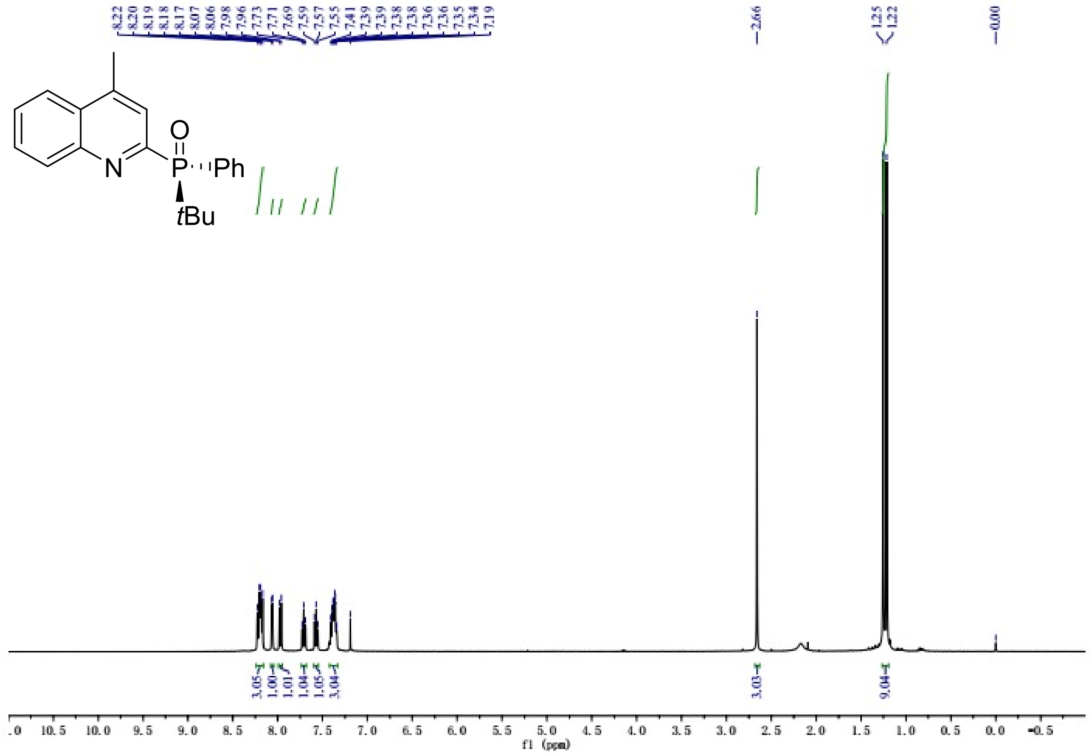

Figure S47. ^{1}H NMR spectrum of **20** in $CDCl_3$

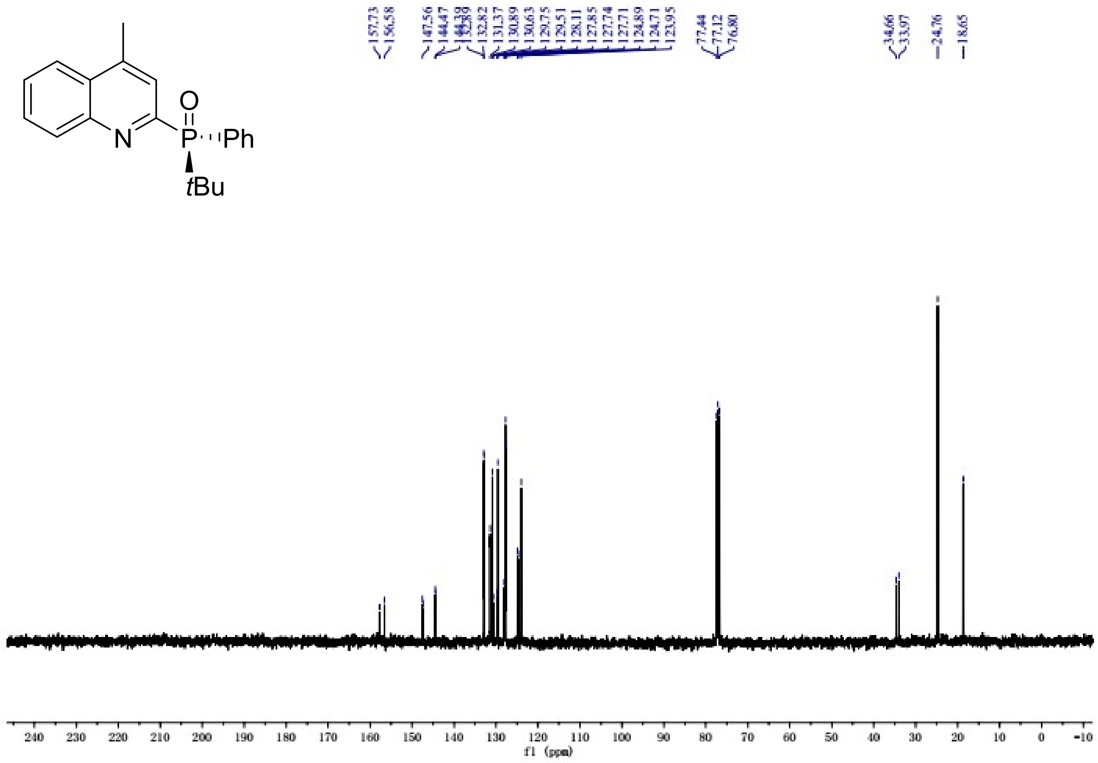

Figure S48. ^{13}C NMR spectrum of **20** in $CDCl_3$

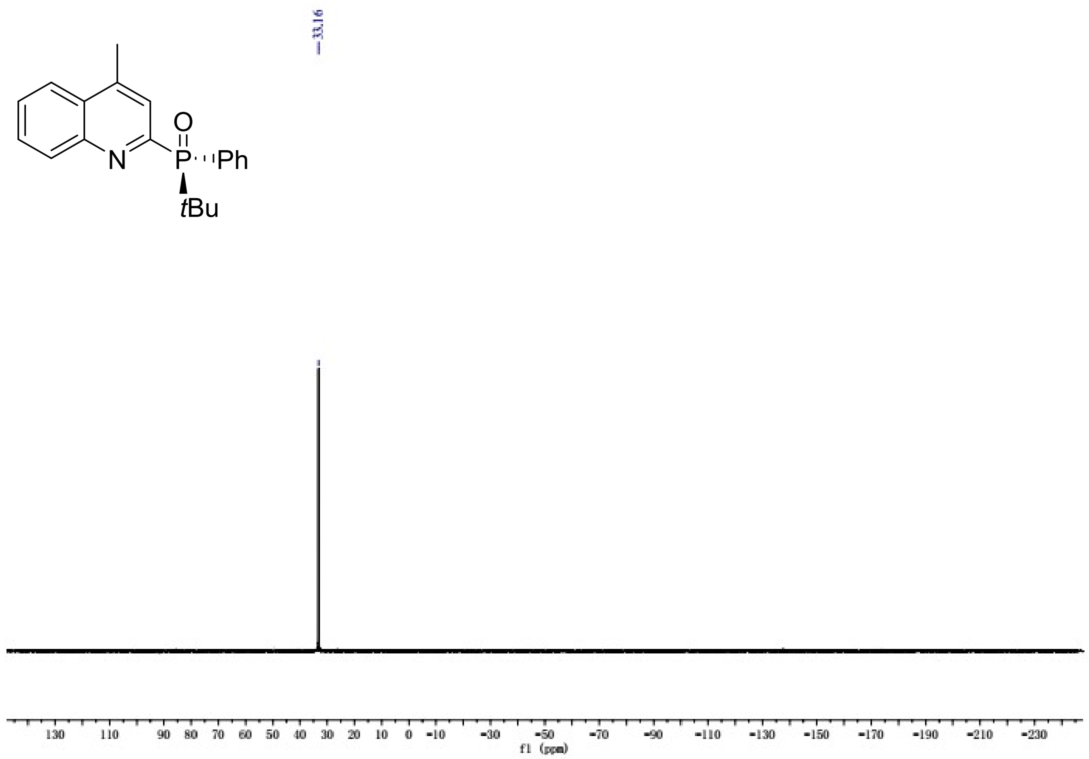

Figure S49. ^{31}P NMR spectrum of **20** in $CDCl_3$

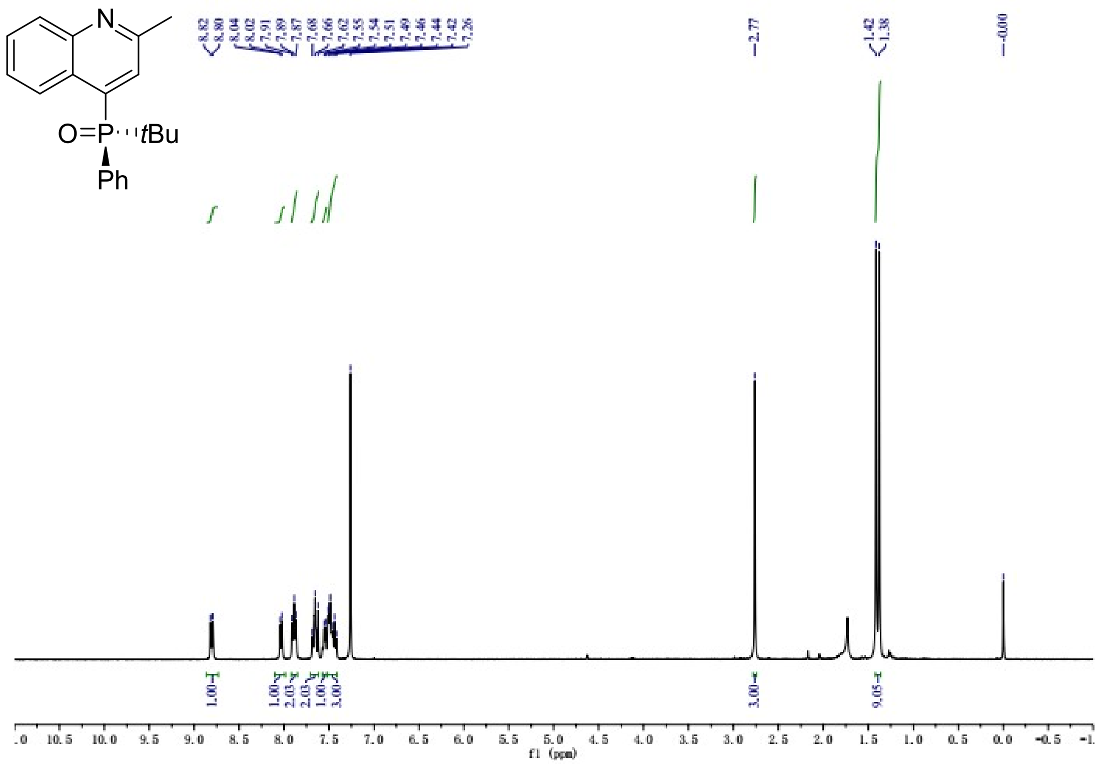

Figure S50. ^{1}H NMR spectrum of **21** in $CDCl_3$

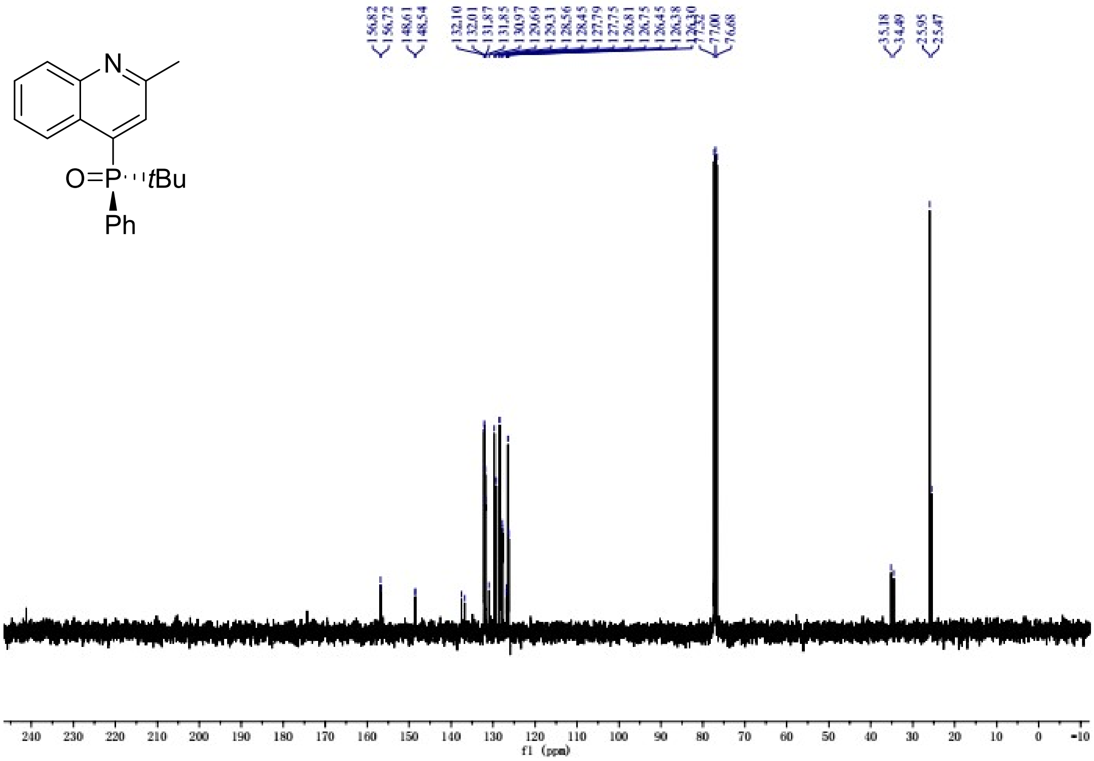

Figure S51. ^{13}C NMR spectrum of **21** in $CDCl_3$

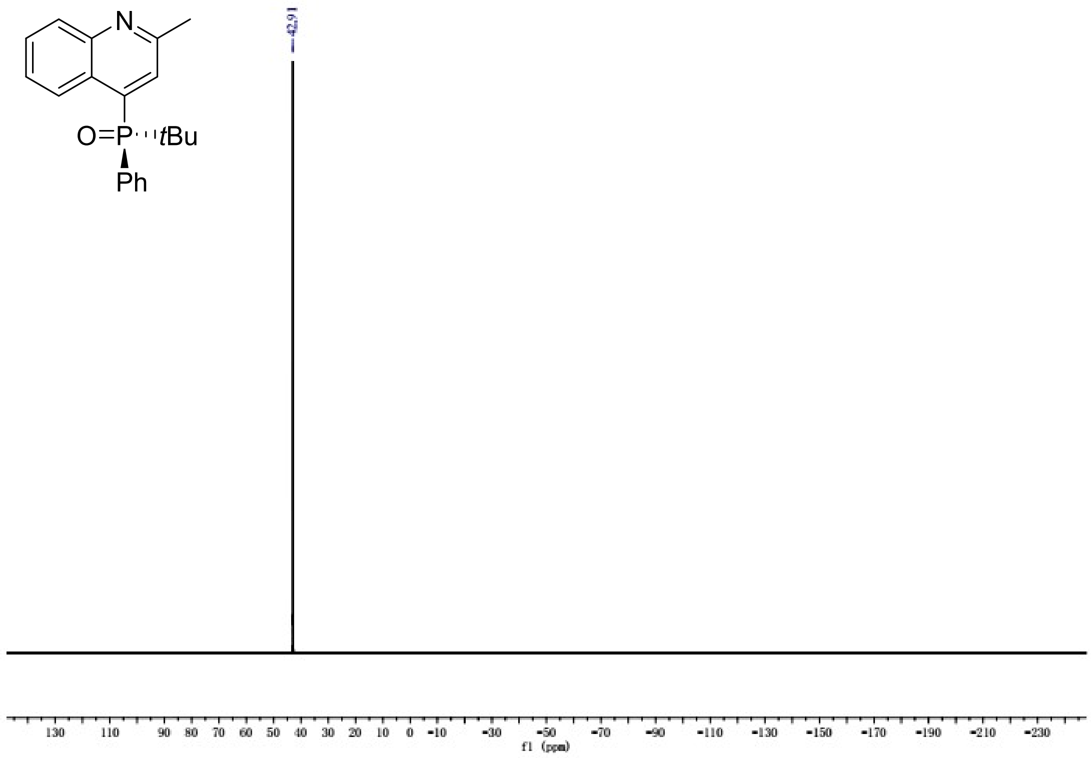

Figure S52. ^{31}P NMR spectrum of **21** in $CDCl_3$

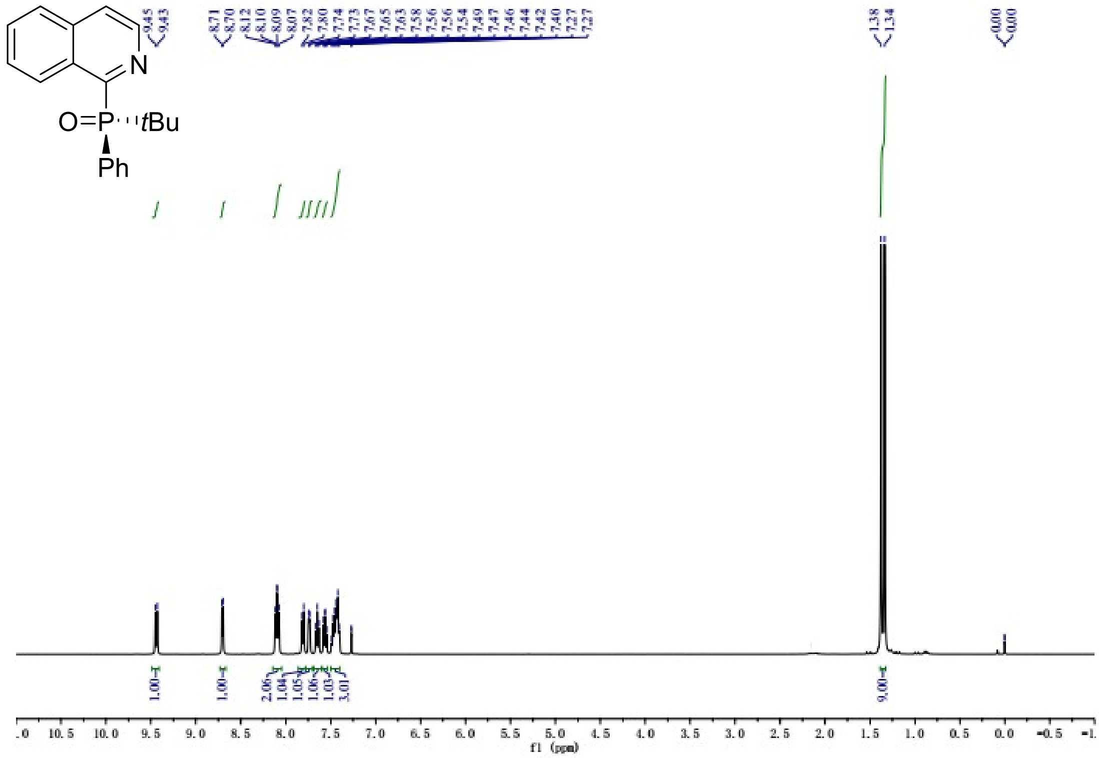

Figure S53. ^{1}H NMR spectrum of **22** in $CDCl_3$

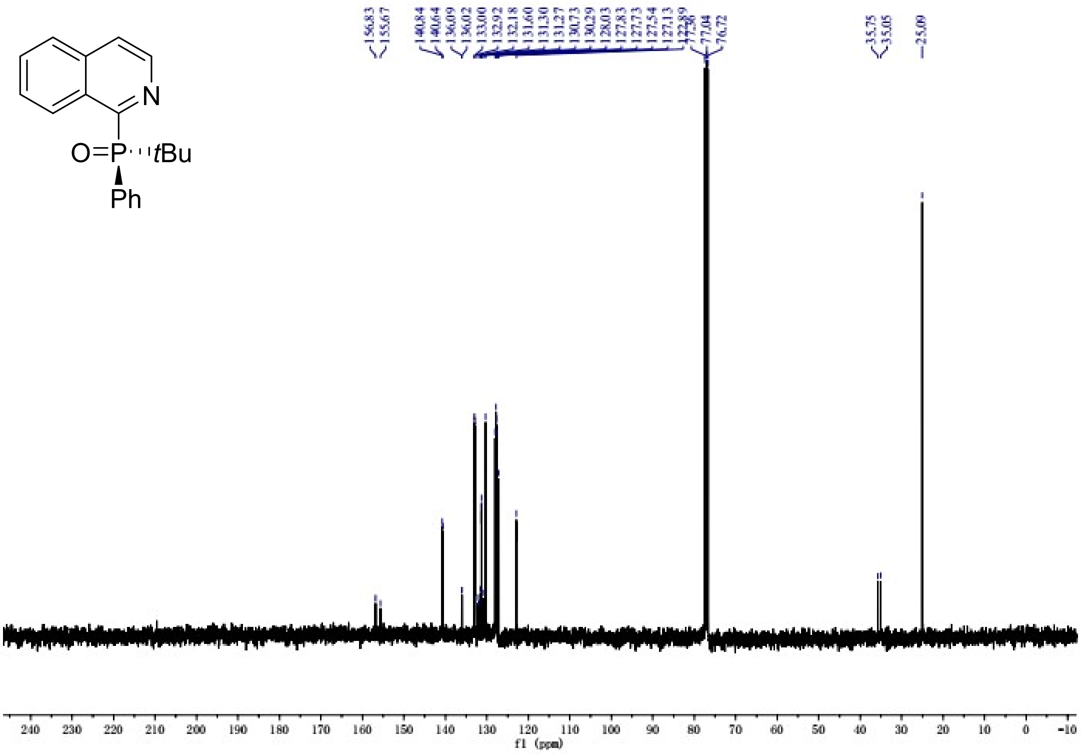

Figure S54. ^{13}C NMR spectrum of **22** in $CDCl_3$

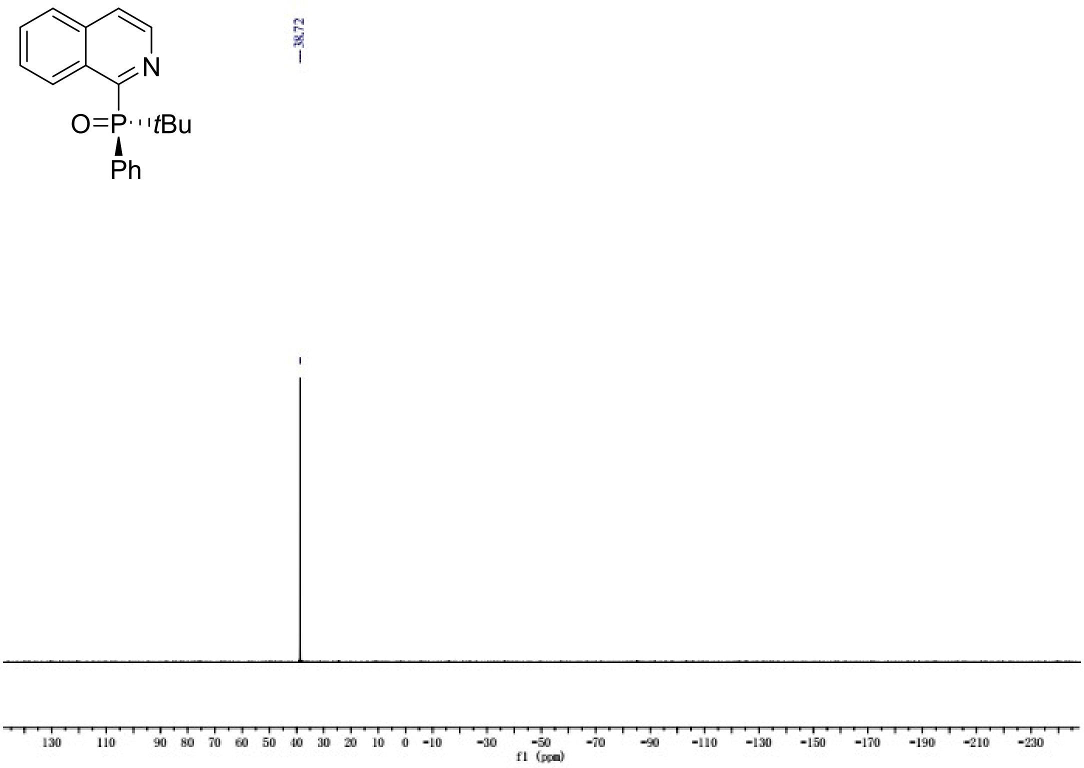

Figure S55. ^{31}P NMR spectrum of **22** in $CDCl_3$

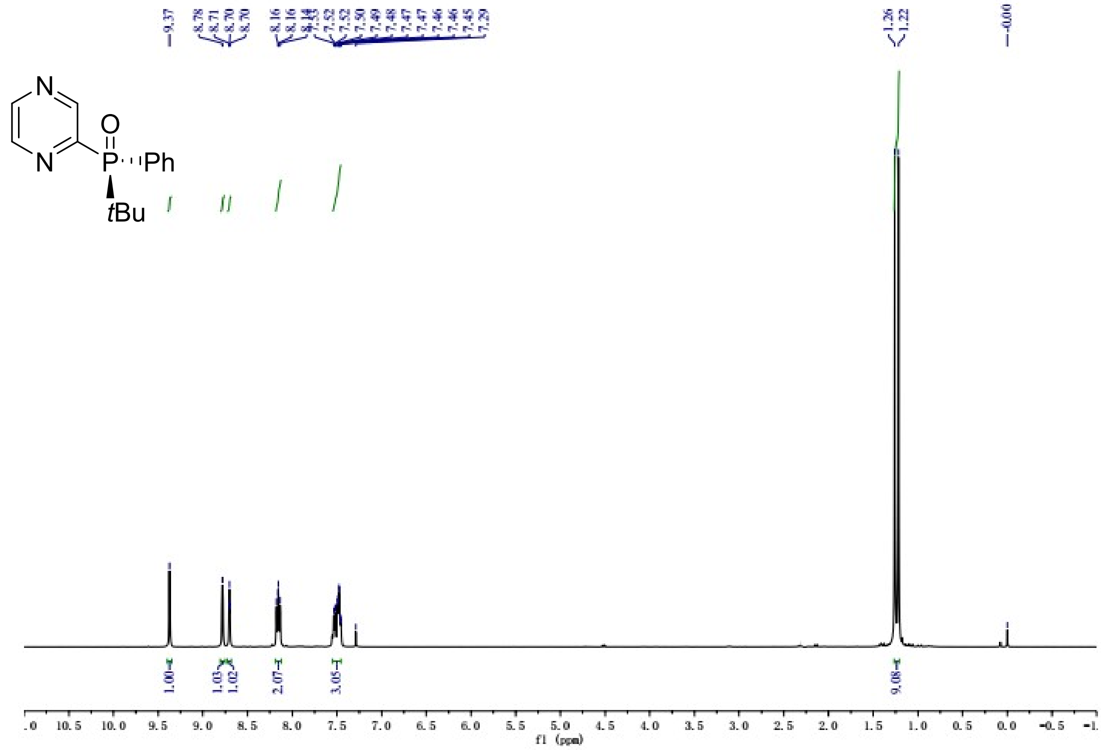

Figure S56. ^{1}H NMR spectrum of **23** in $CDCl_3$

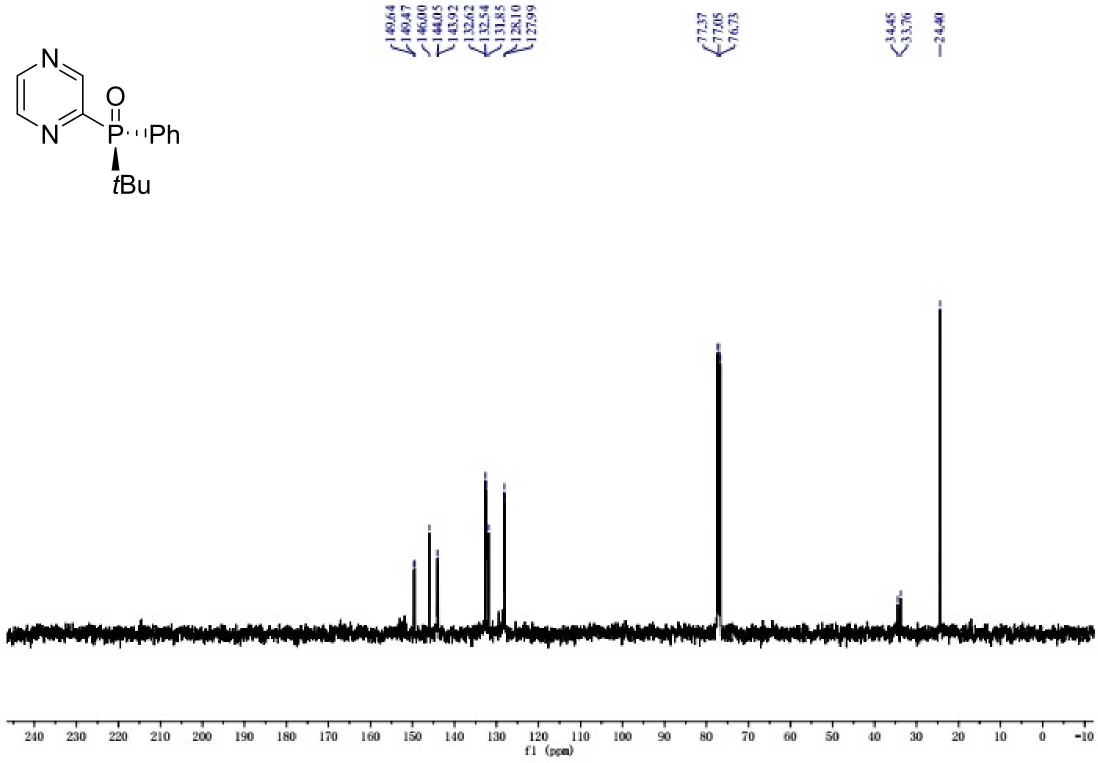

Figure S57. ^{13}C NMR spectrum of **23** in $CDCl_3$

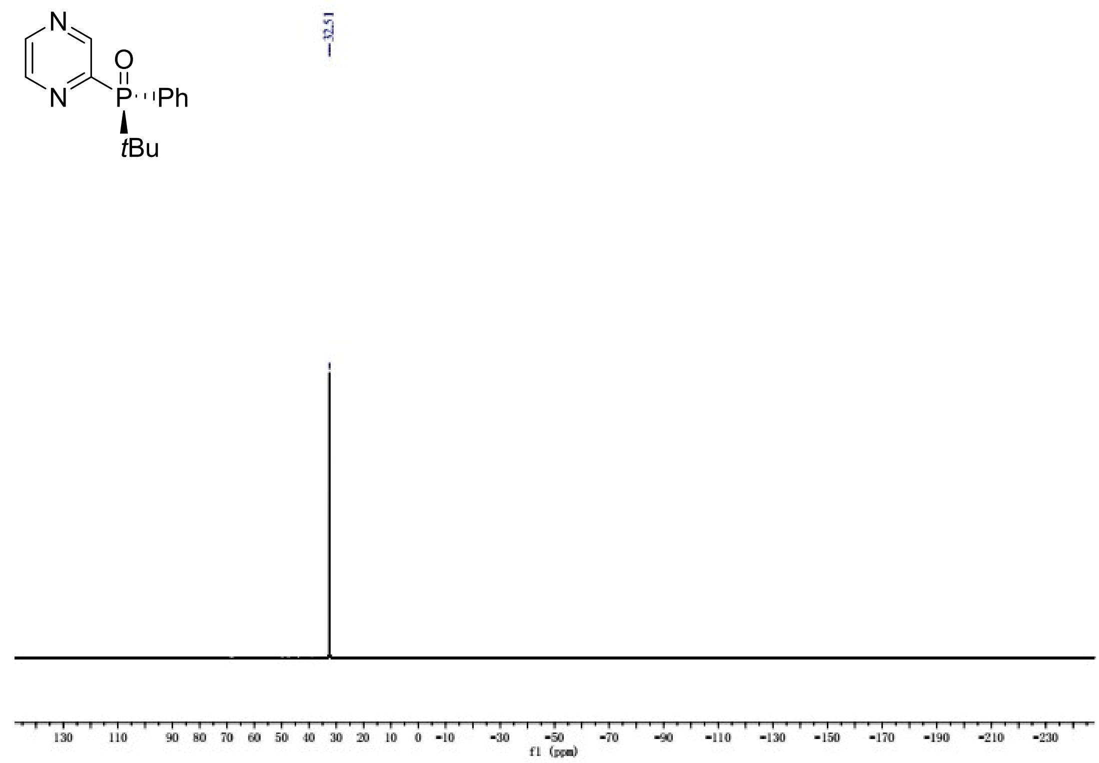

Figure S58. ^{31}P NMR spectrum of **23** in $CDCl_3$

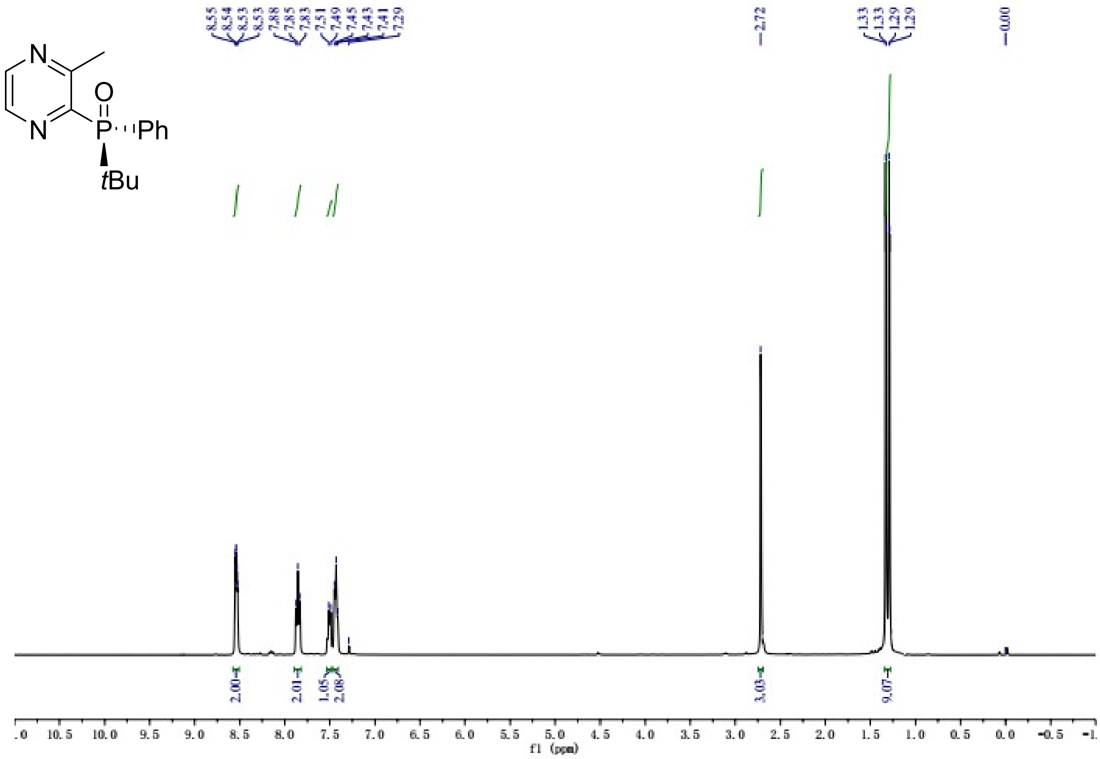

Figure S59. ^{1}H NMR spectrum of **24** in $CDCl_3$

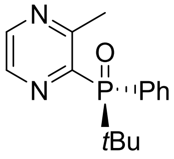

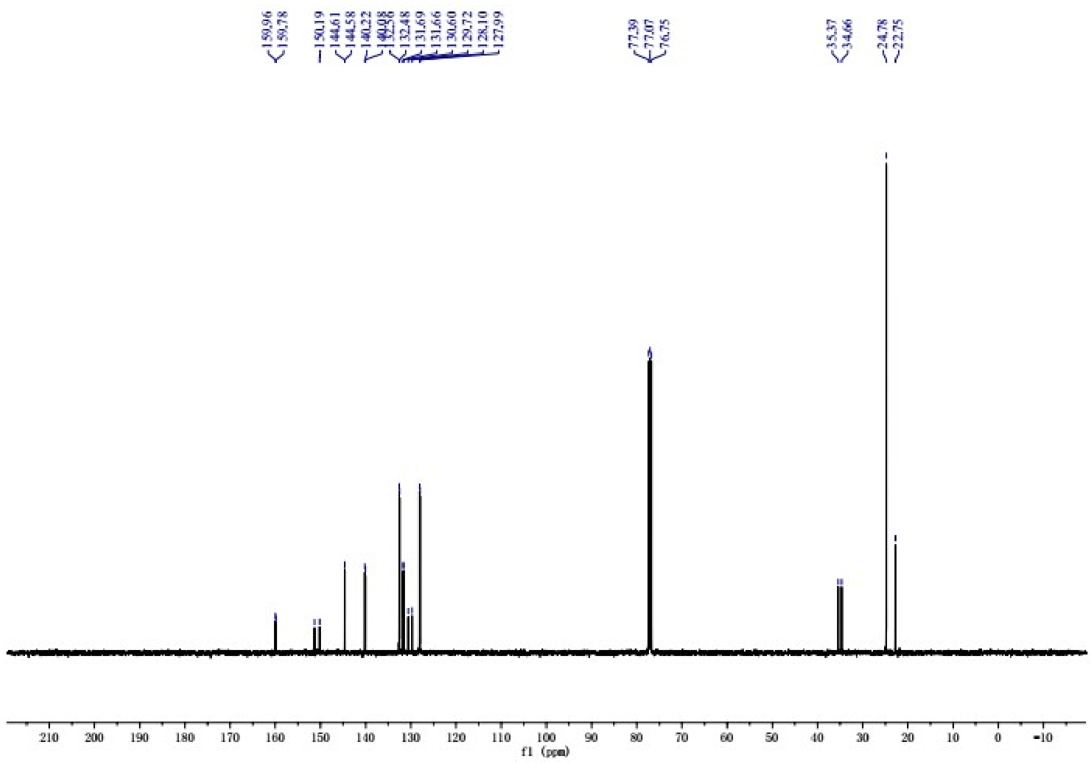

Figure S60. ^{13}C NMR spectrum of **24** in $CDCl_3$

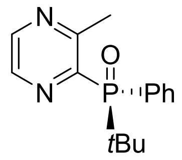

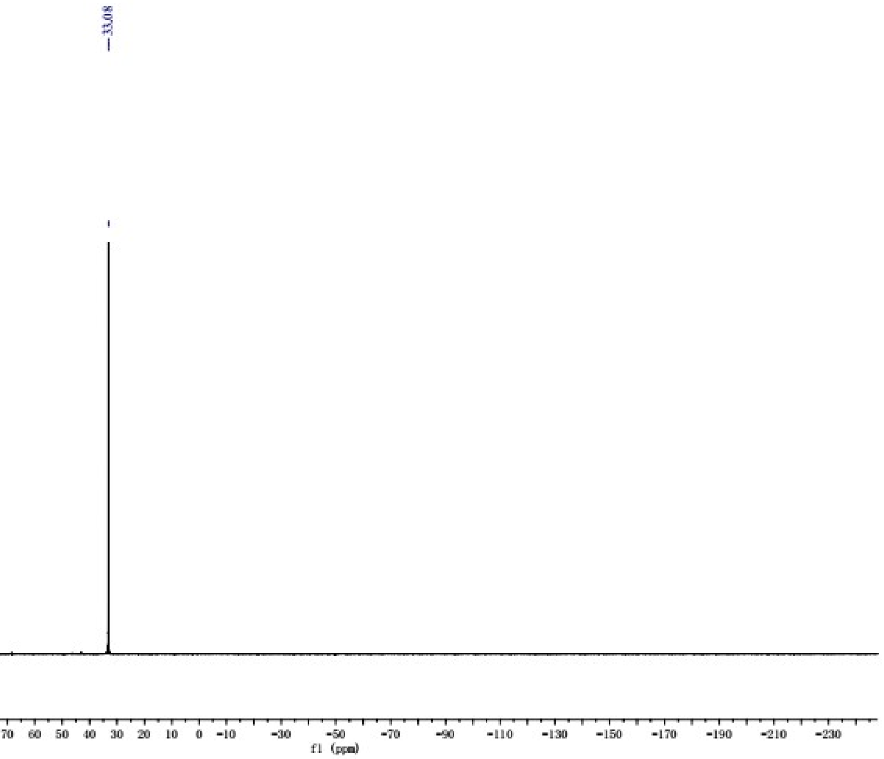

Figure S61. ^{31}P NMR spectrum of **24** in $CDCl_3$

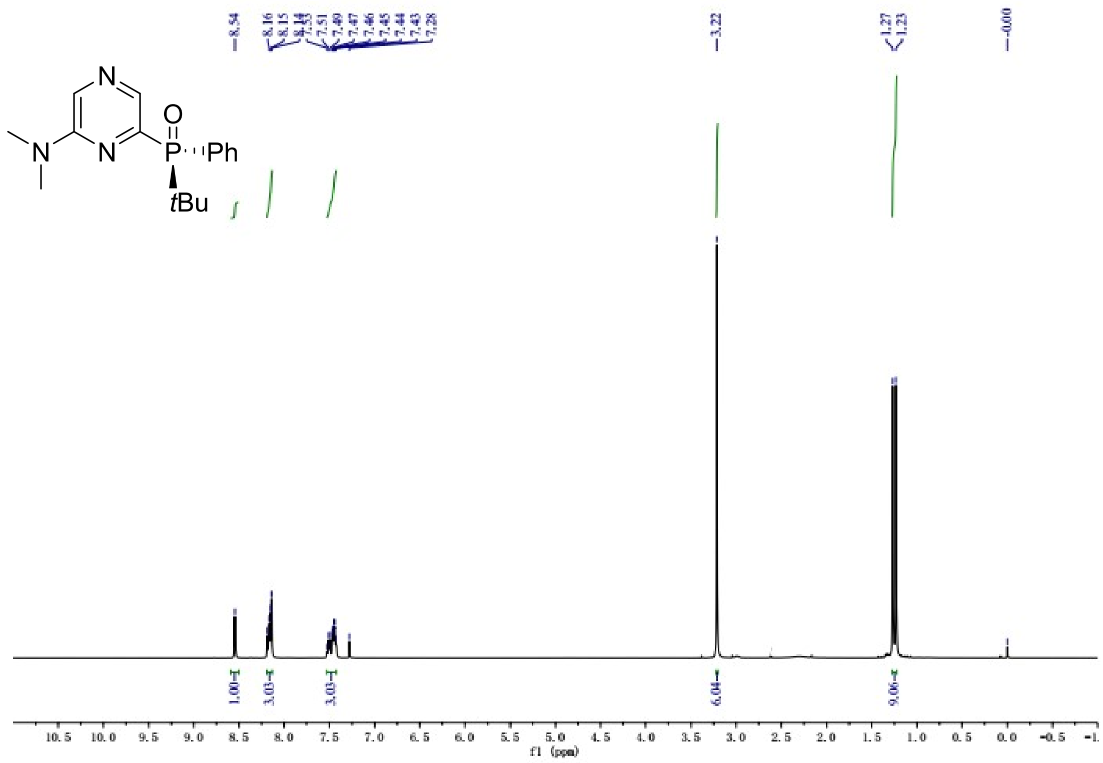

Figure S62. ^1H NMR spectrum of **25** in $CDCl_3$

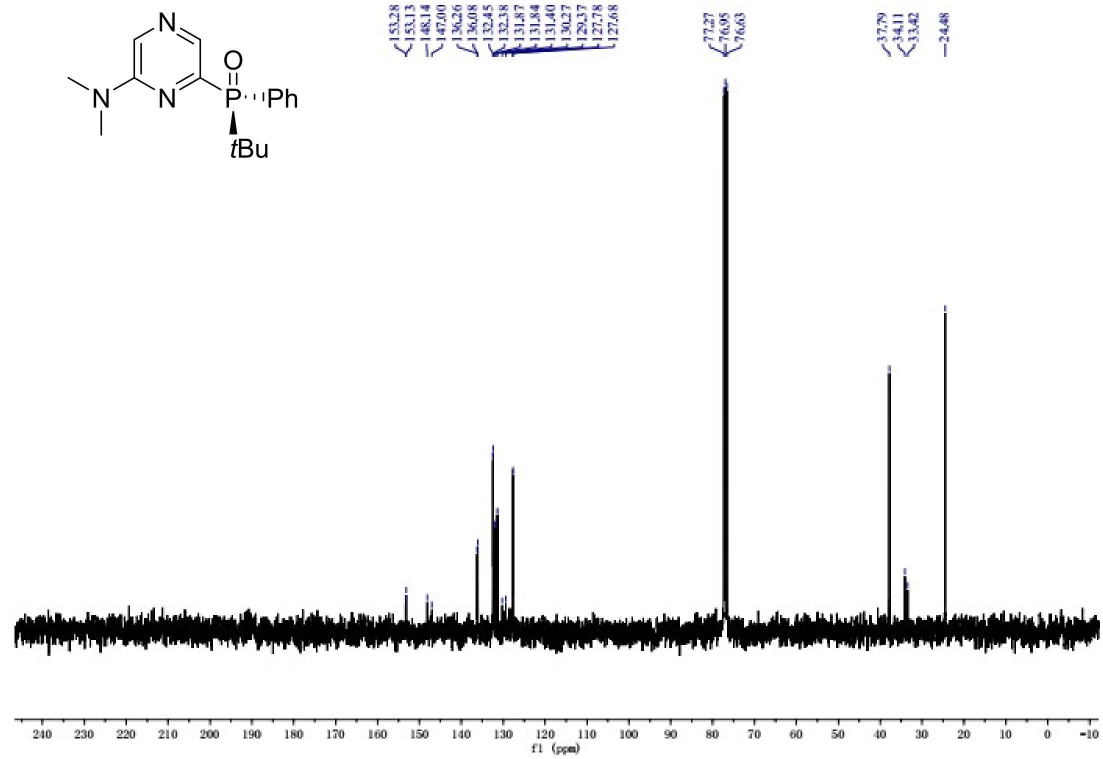

Figure S63. ^{13}C NMR spectrum of **25** in $CDCl_3$

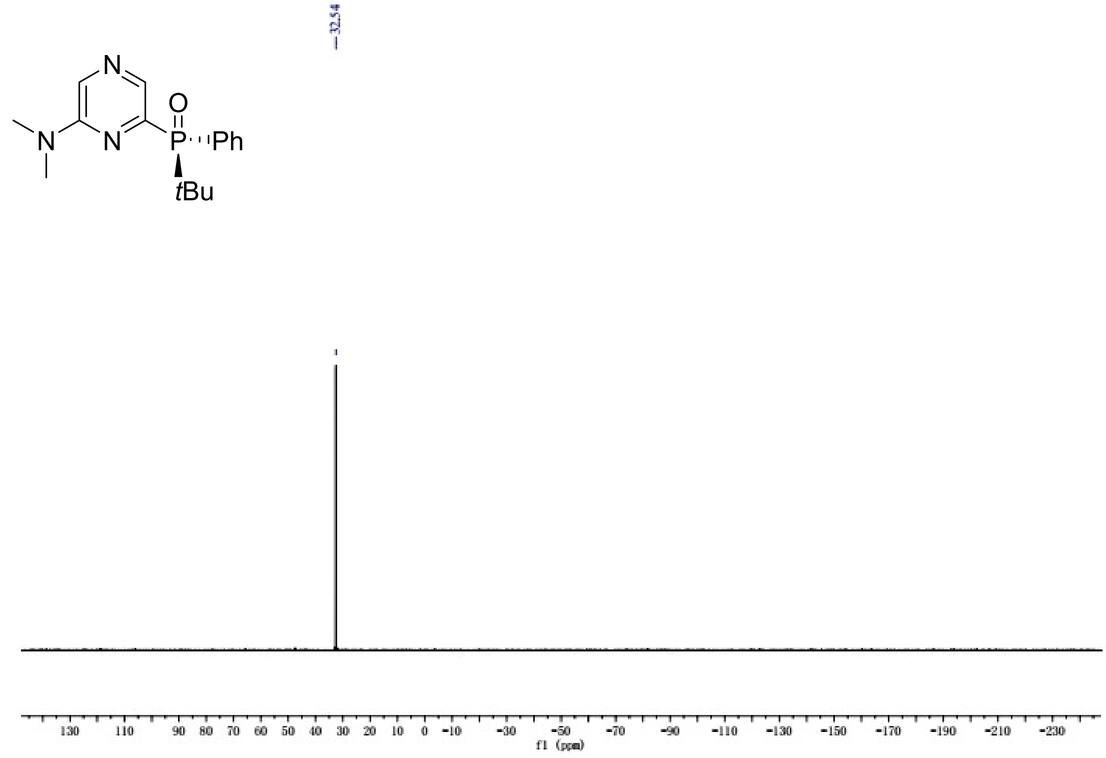

Figure S64. ^{31}P NMR spectrum of **25** in $CDCl_3$

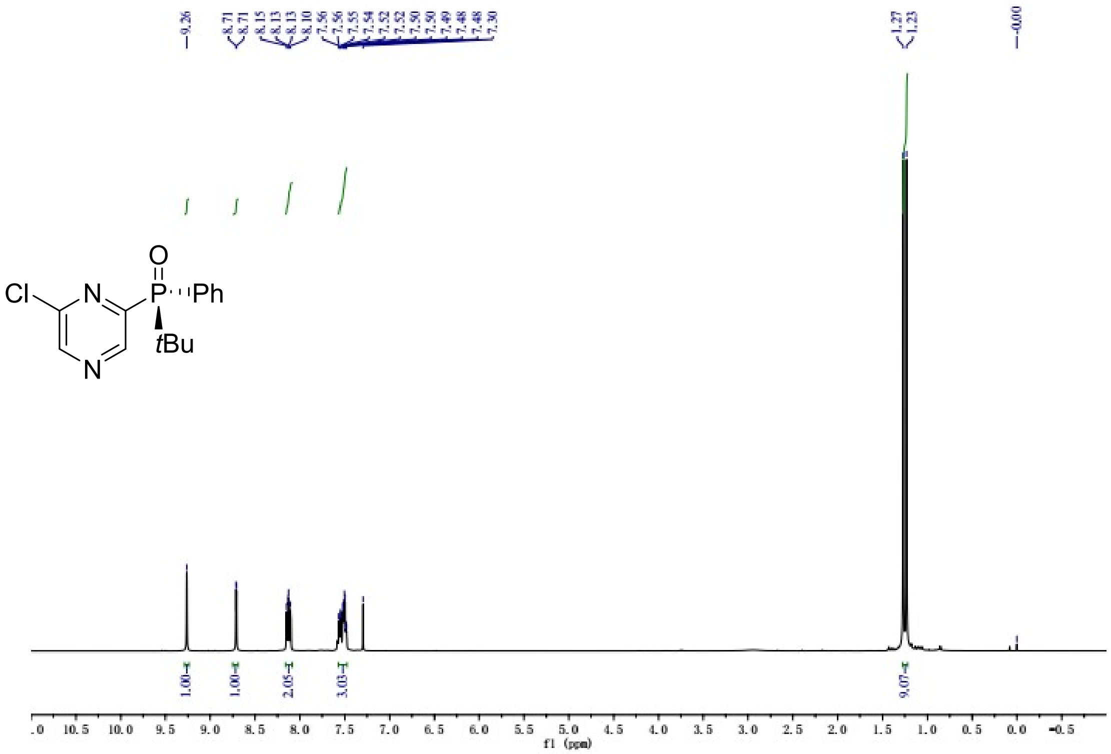

Figure S65. ^{1}H NMR spectrum of **26** in $CDCl_3$

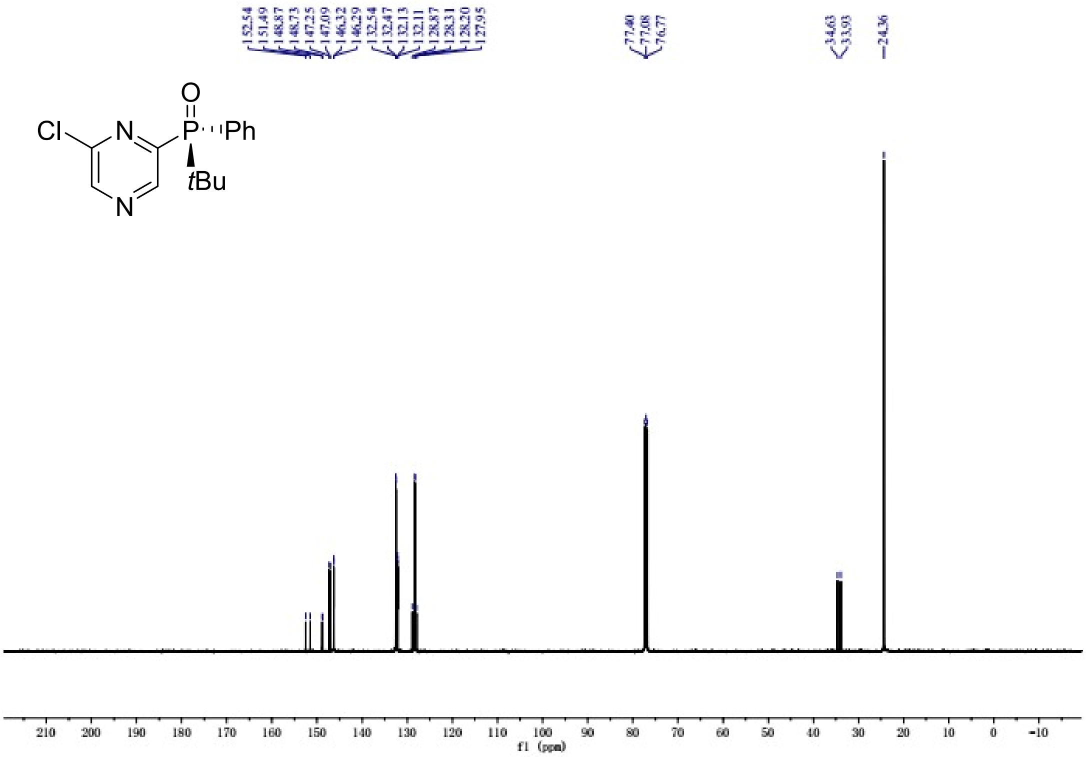

Figure S66. ^{13}C NMR spectrum of **26** in $CDCl_3$

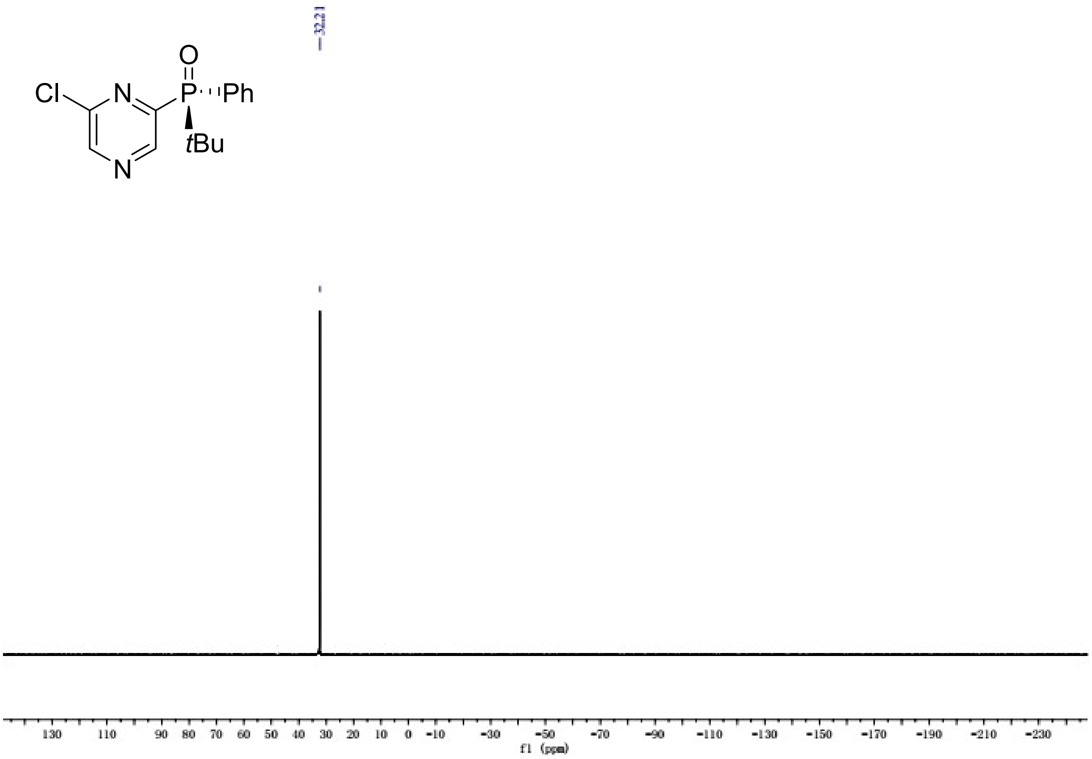

Figure S67. ^{31}P NMR spectrum of **26** in $CDCl_3$

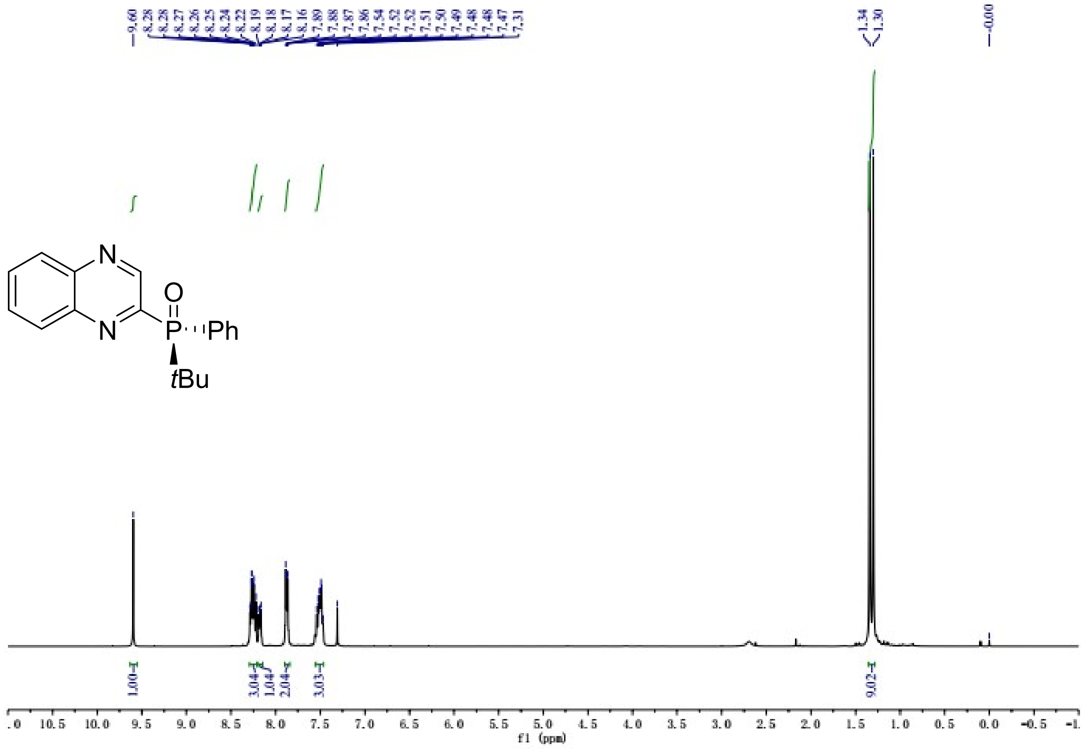

Figure S68. ^{1}H NMR spectrum of **27** in $CDCl_3$

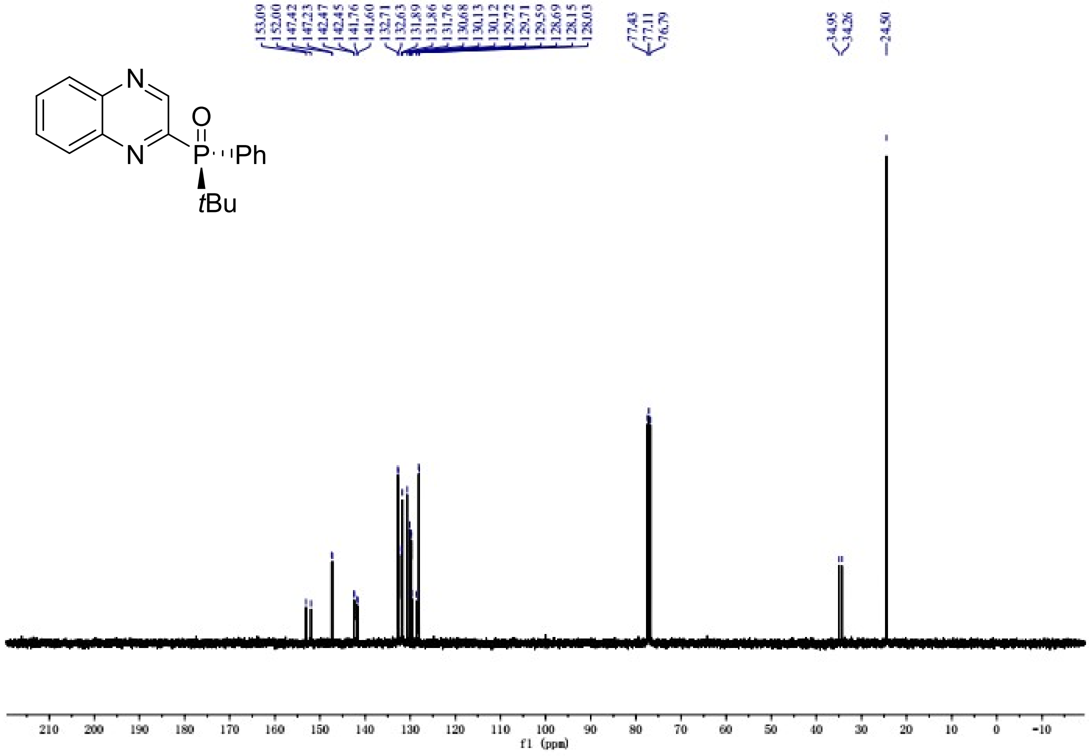

Figure S69. ^{13}C NMR spectrum of **27** in $CDCl_3$

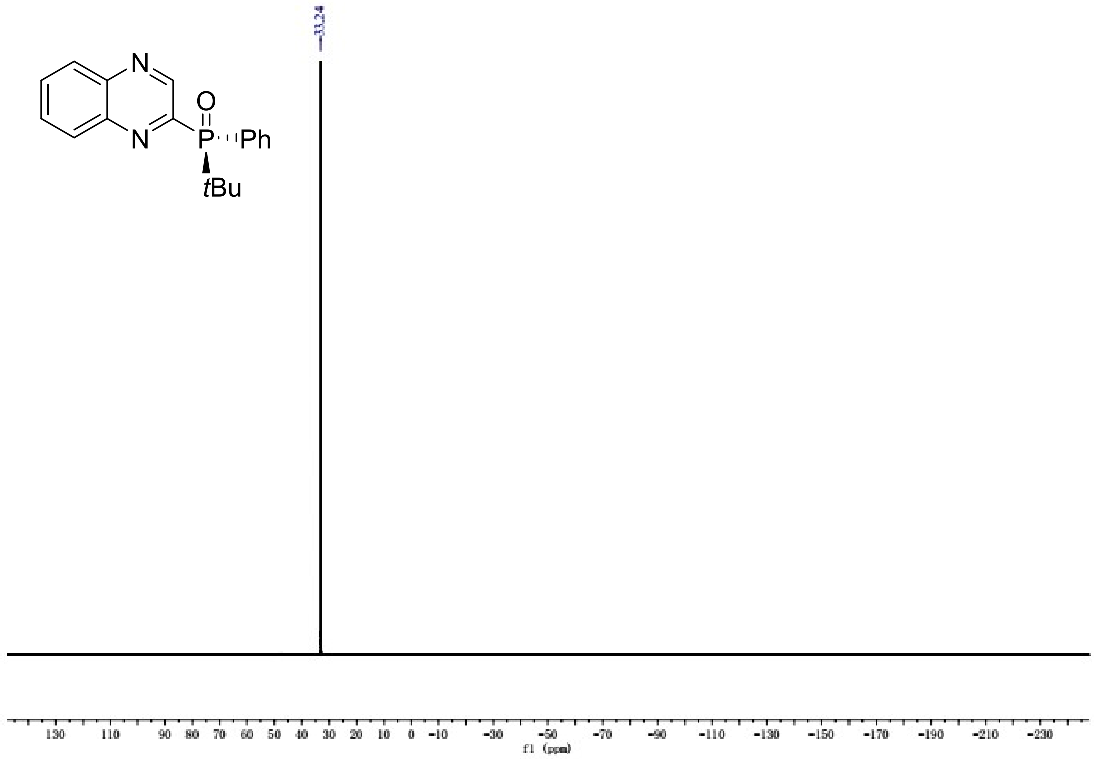

Figure S70. ^{31}P NMR spectrum of **27** in $CDCl_3$

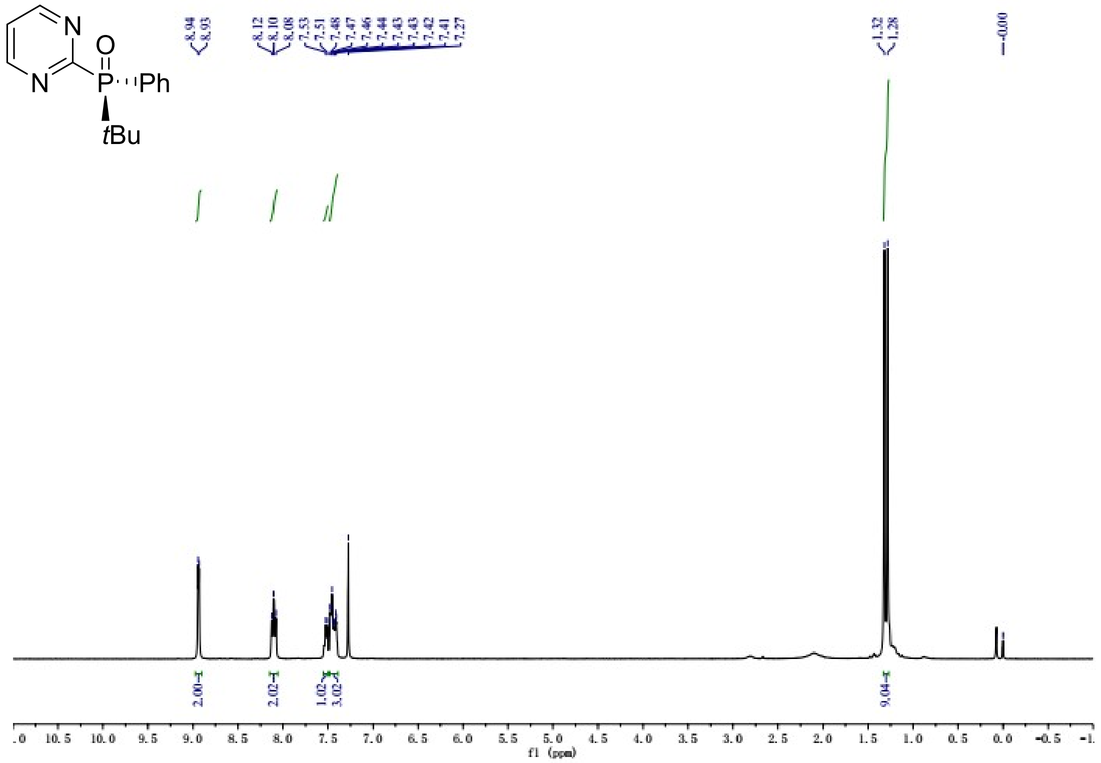

Figure S71. ^{1}H NMR spectrum of **28** in $CDCl_3$

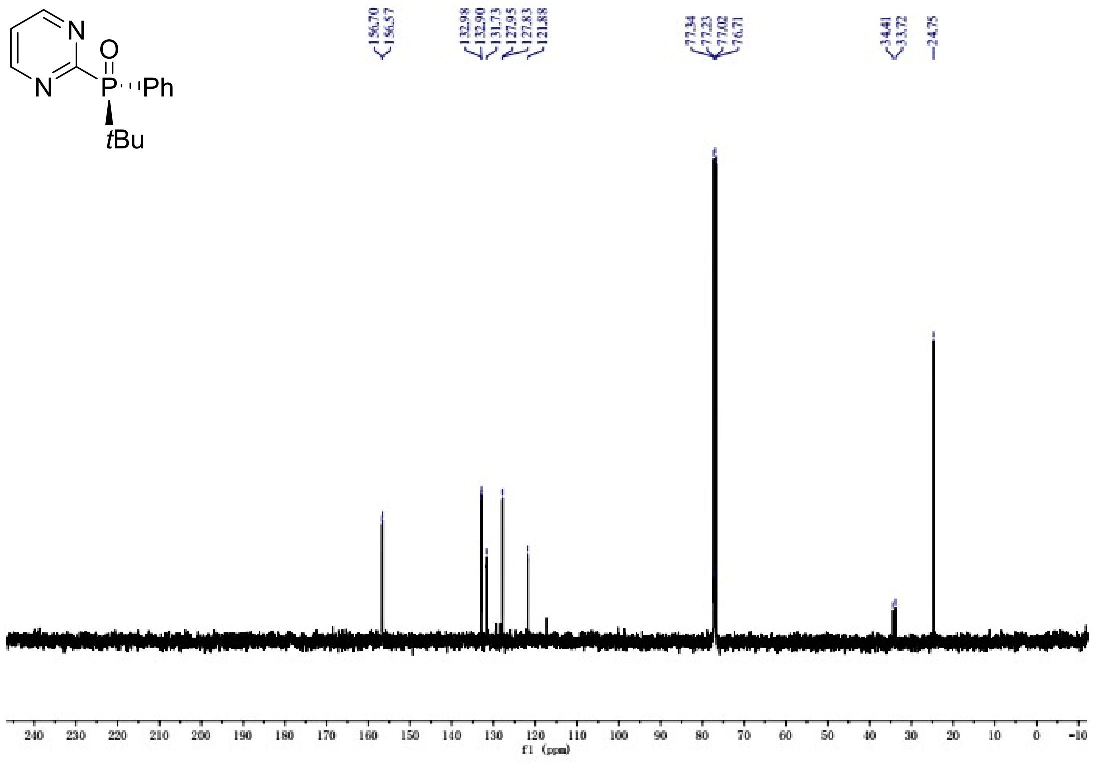

Figure S72. ^{13}C NMR spectrum of **28** in $CDCl_3$

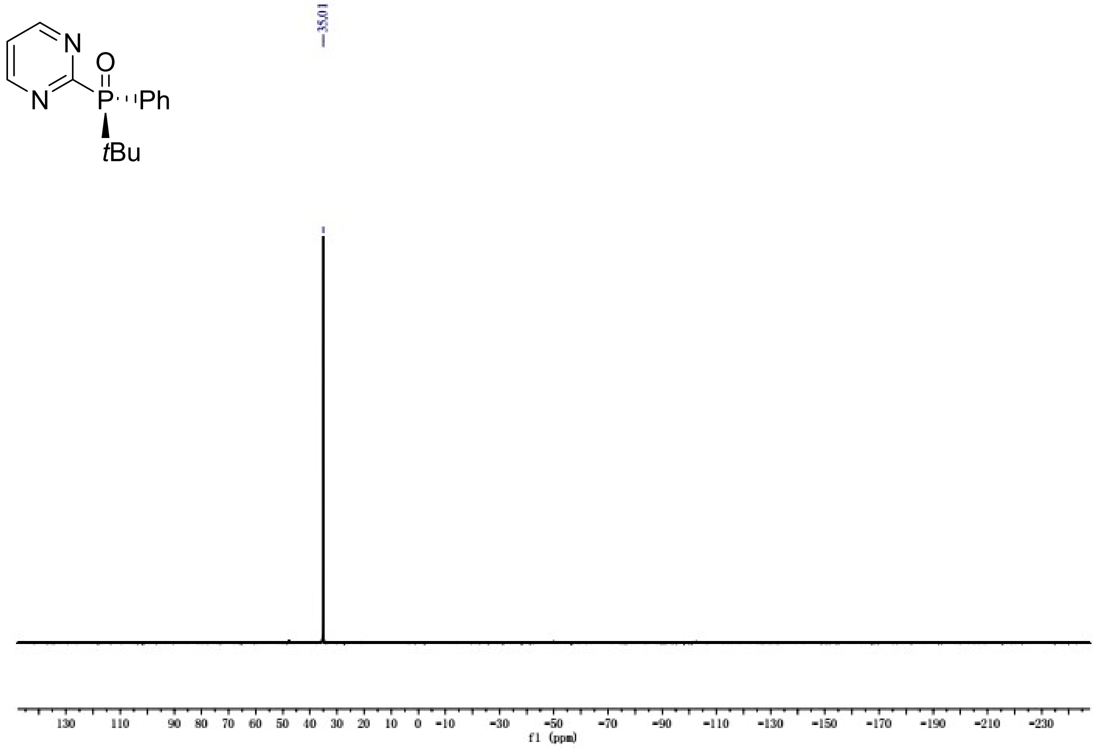

Figure S73. ^{31}P NMR spectrum of **28** in $CDCl_3$

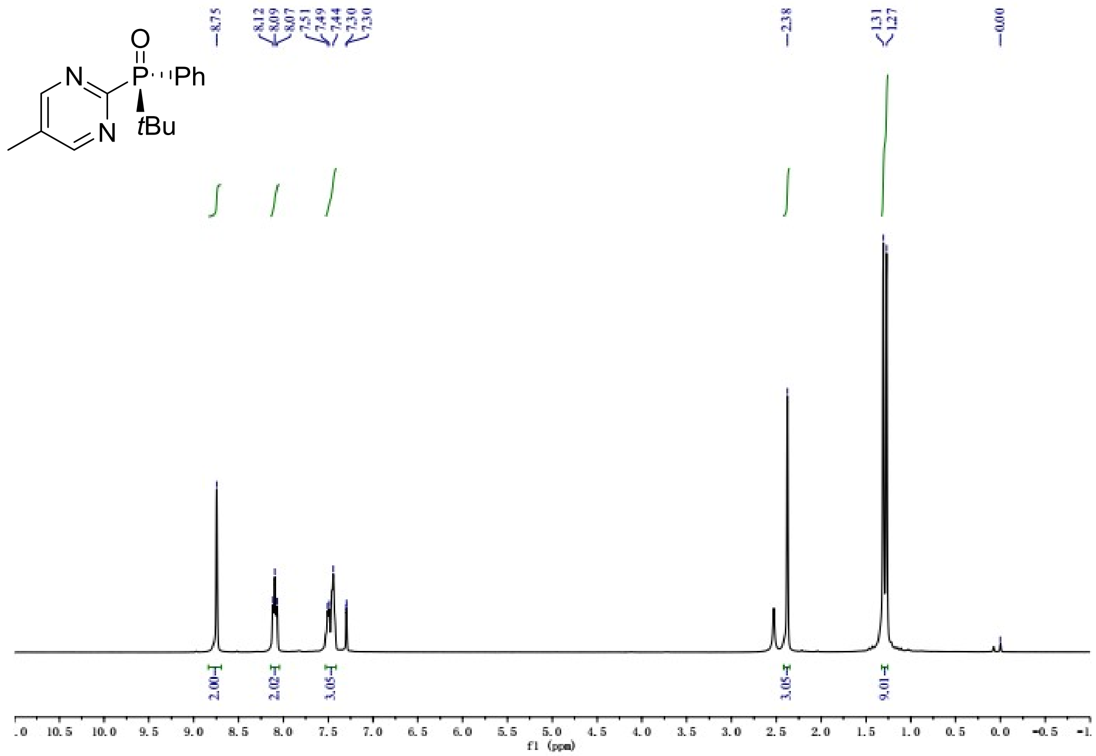

Figure S74. ^{1}H NMR spectrum of **29** in $CDCl_3$

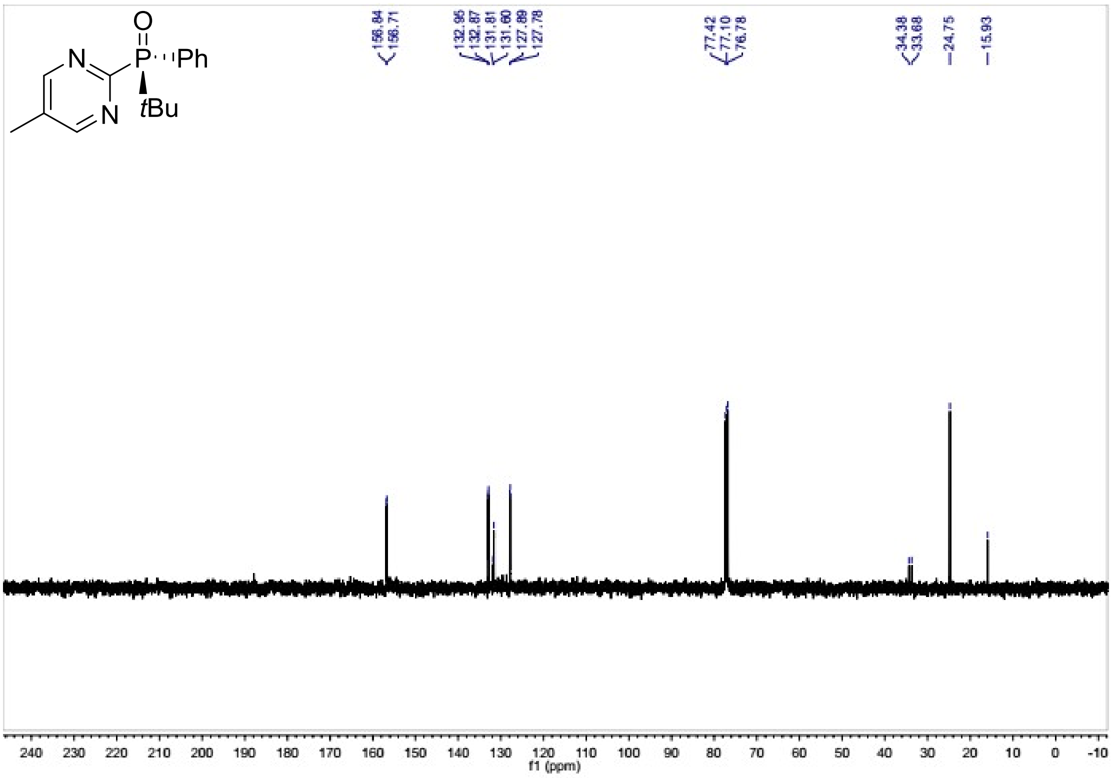

Figure S75. ^{13}C NMR spectrum of **29** in $CDCl_3$

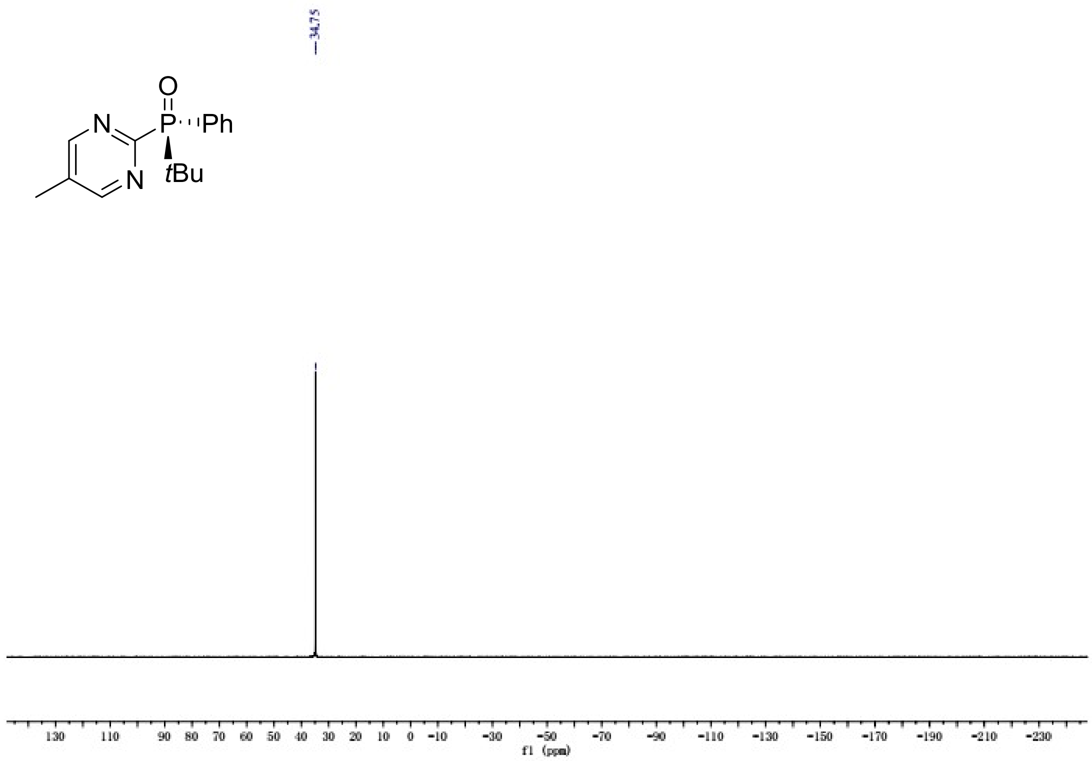

Figure S76. ^{31}P NMR spectrum of **29** in $CDCl_3$

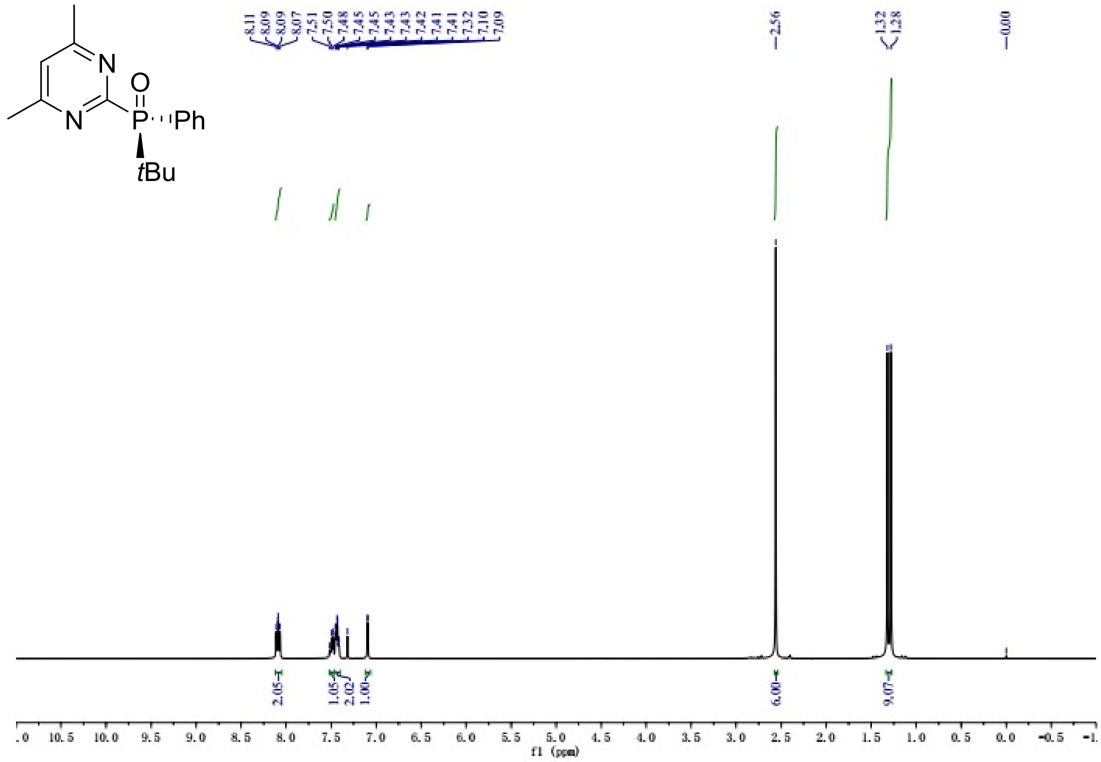

Figure S77. ^{1}H NMR spectrum of (*R*)-**30** in $CDCl_3$

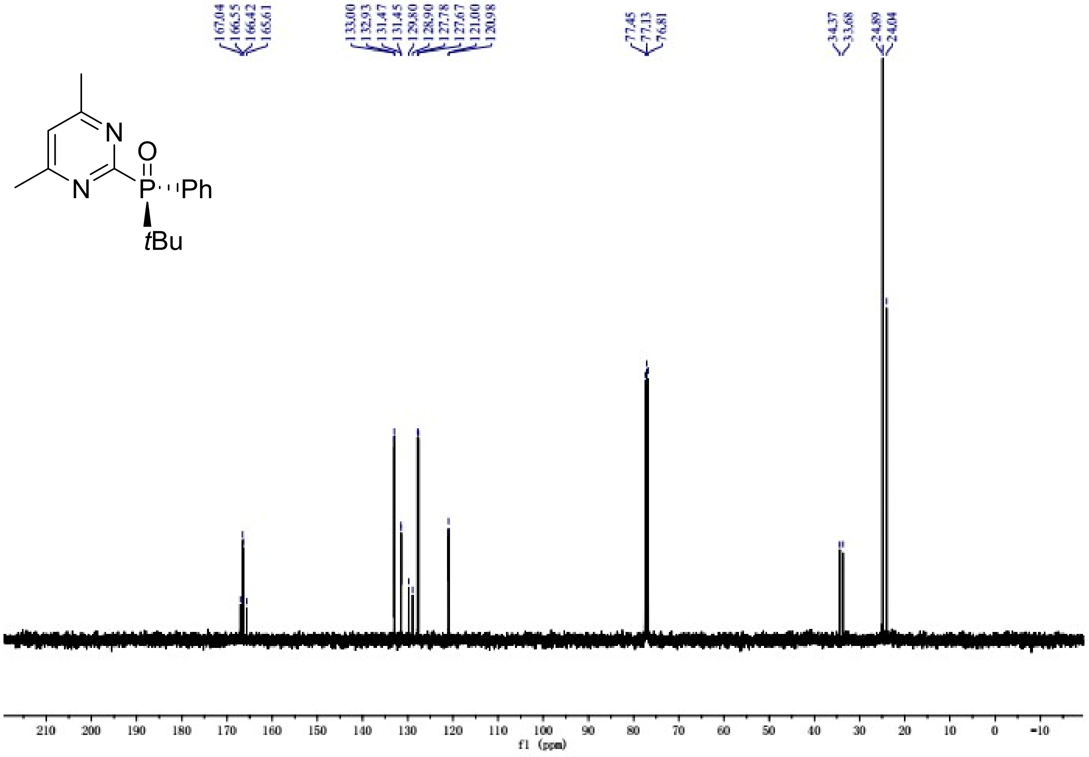

Figure S78. ^{13}C NMR spectrum of **30** in $CDCl_3$

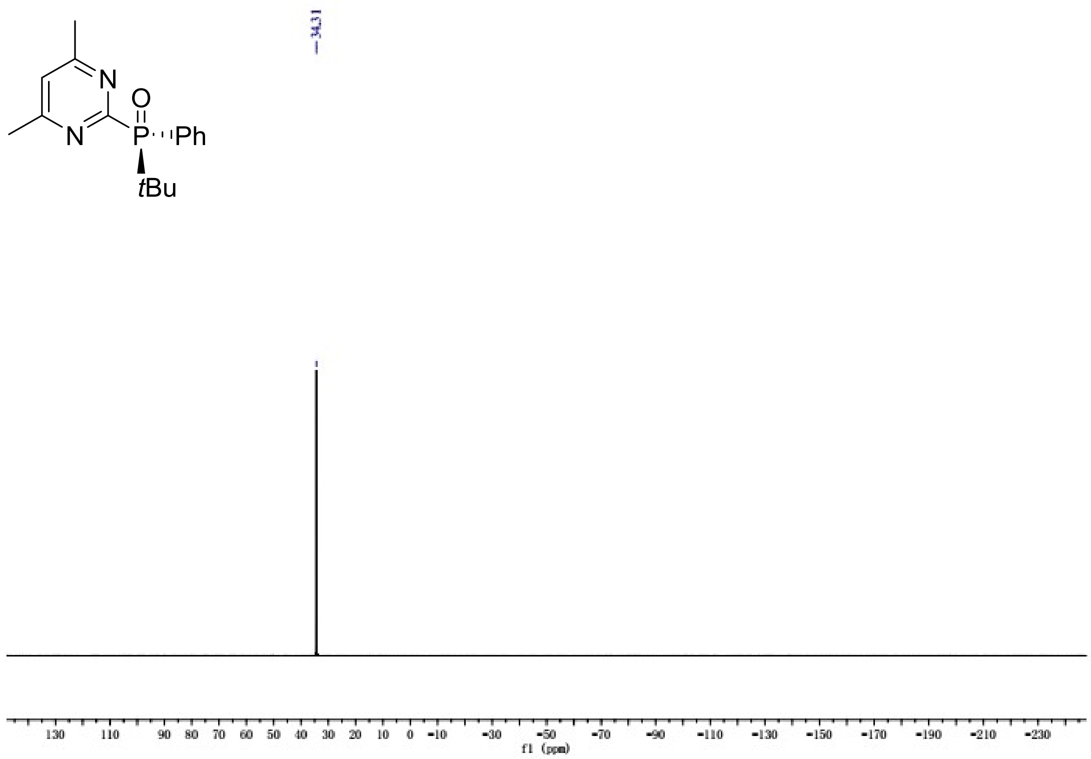

Figure S79. ^{31}P NMR spectrum of **30** in $CDCl_3$

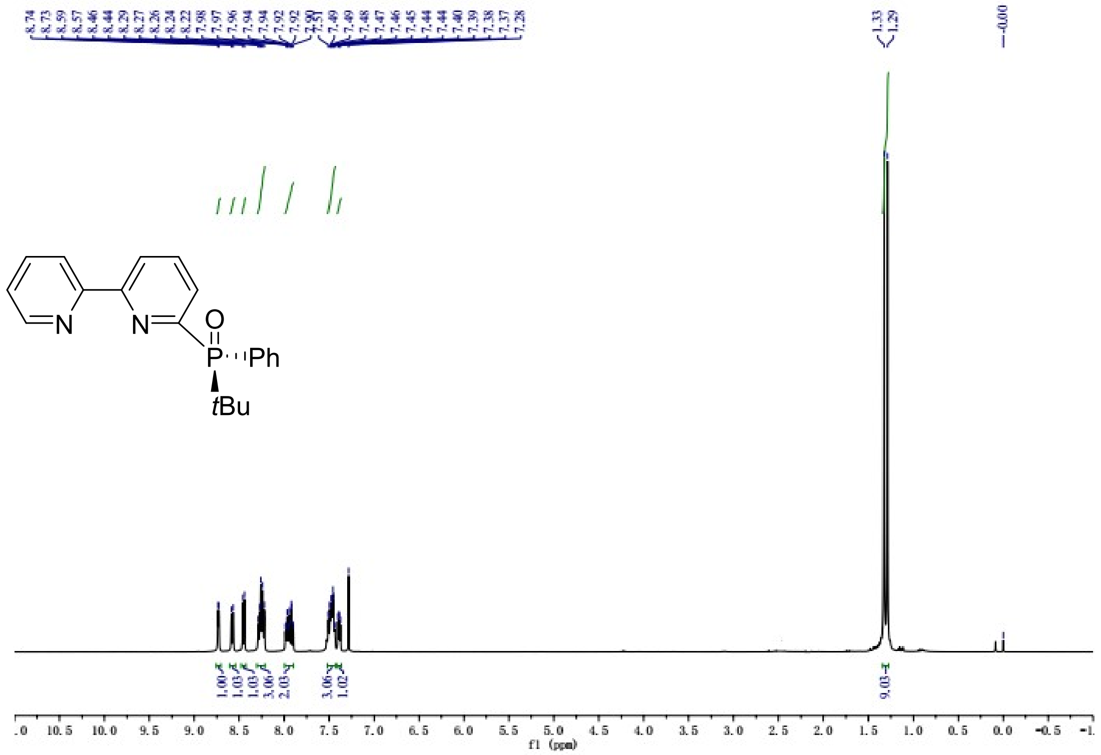

Figure S80. ^1H NMR spectrum of **31** in $CDCl_3$

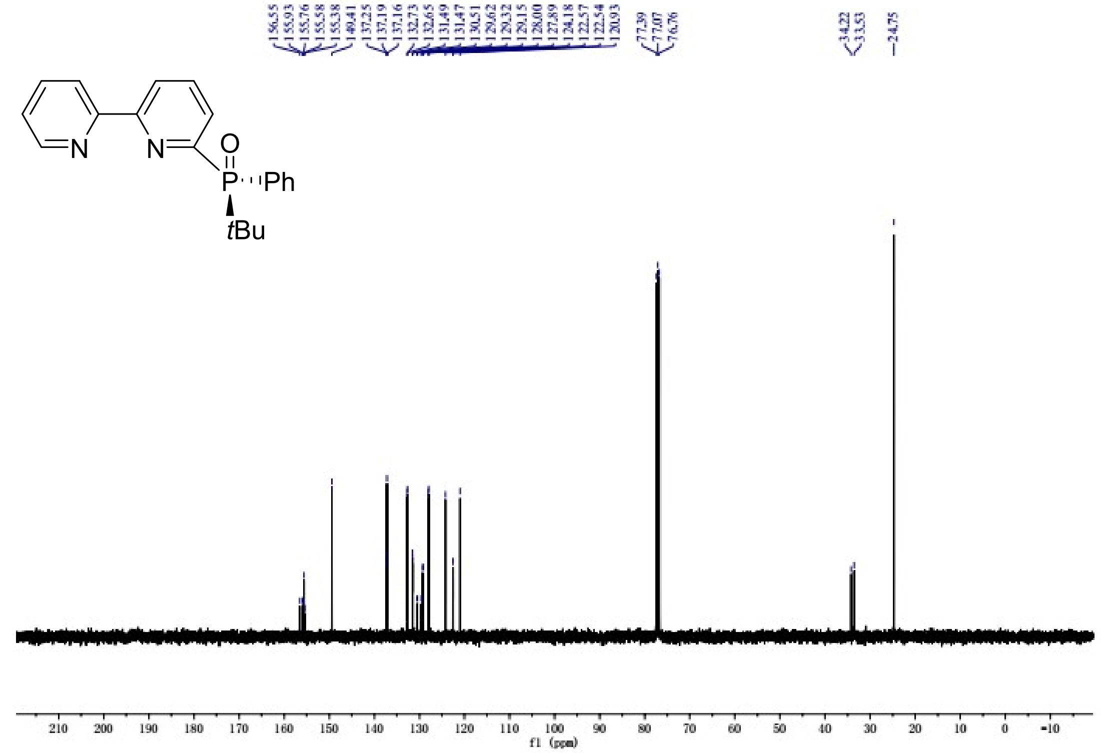

Figure S81. ^{13}C NMR spectrum of **31** in $CDCl_3$

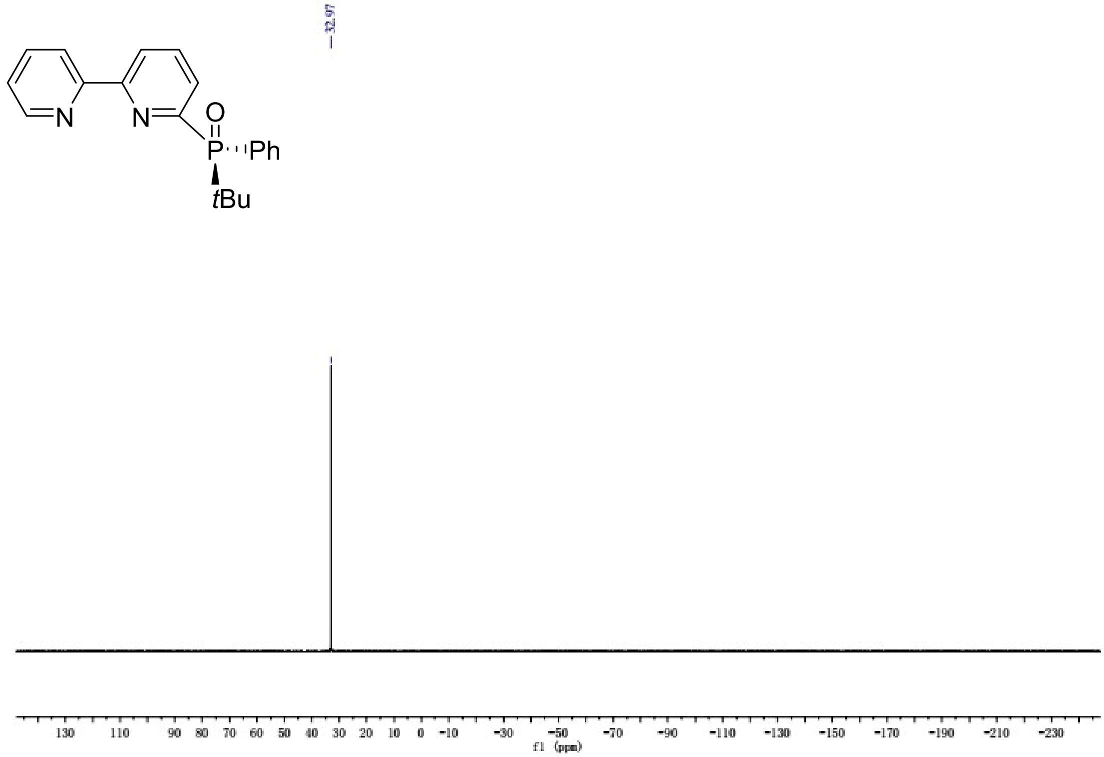

Figure S82. ^{31}P NMR spectrum of **31** in $CDCl_3$

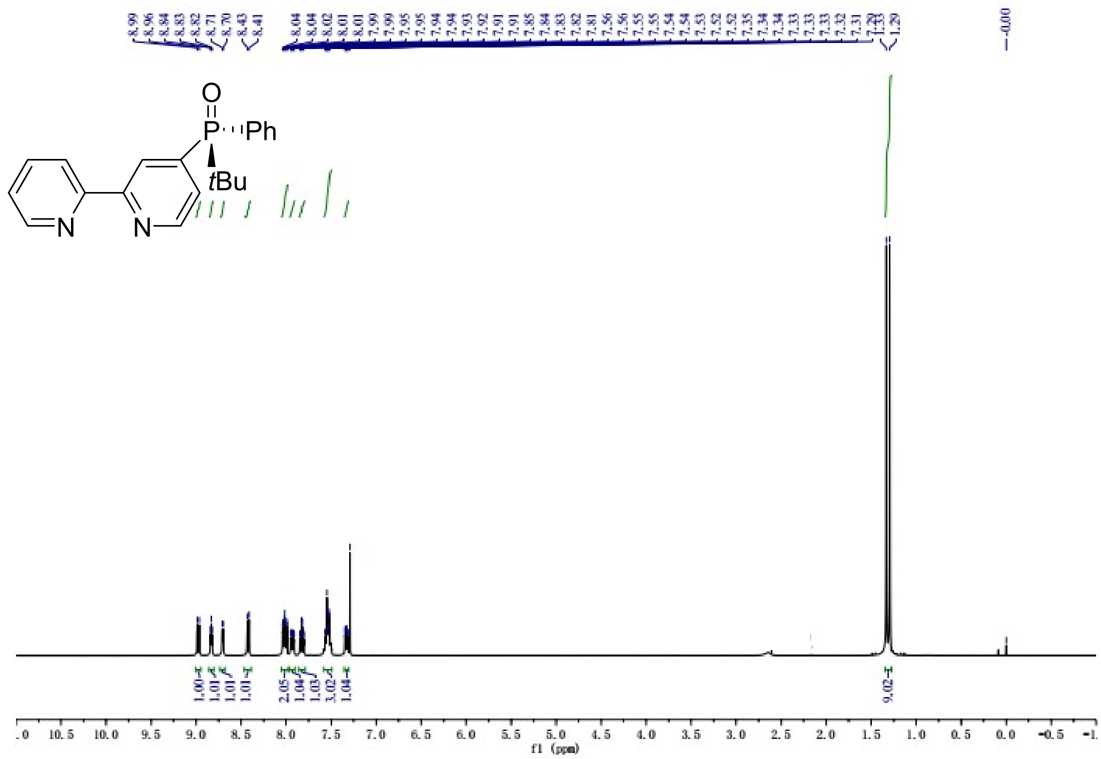

Figure S83. ^{1}H NMR spectrum of **32** in $CDCl_3$

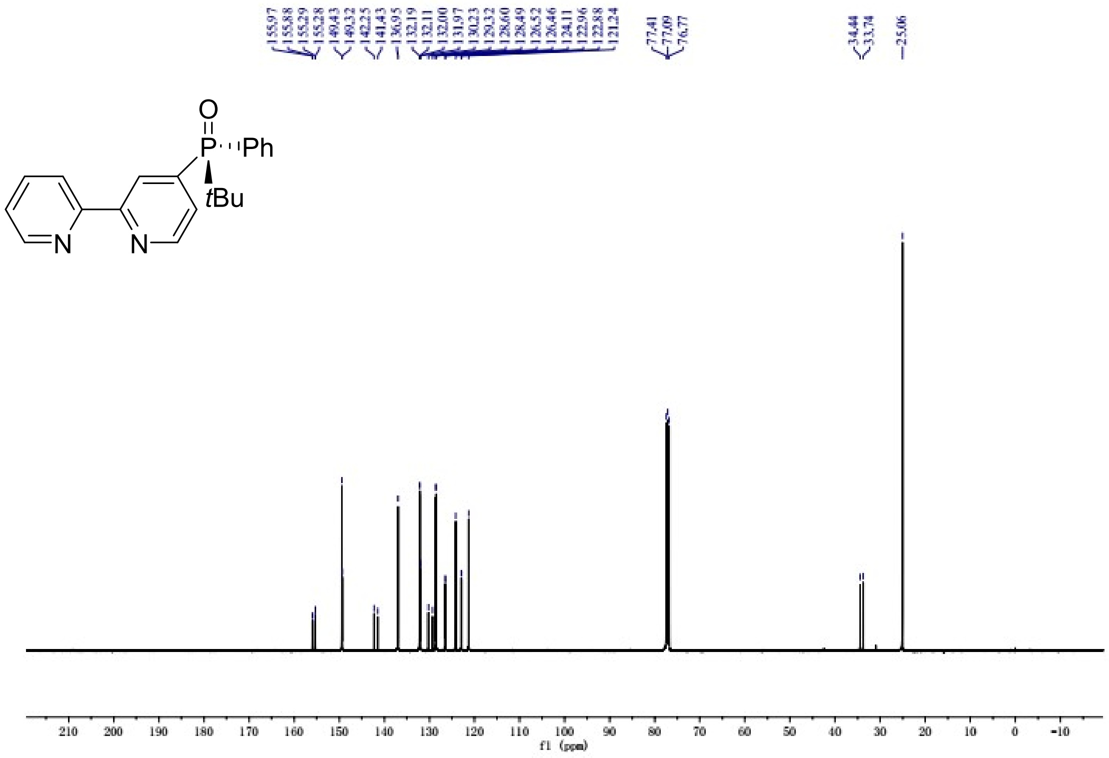

Figure S84. ^{13}C NMR spectrum of **32** in $CDCl_3$

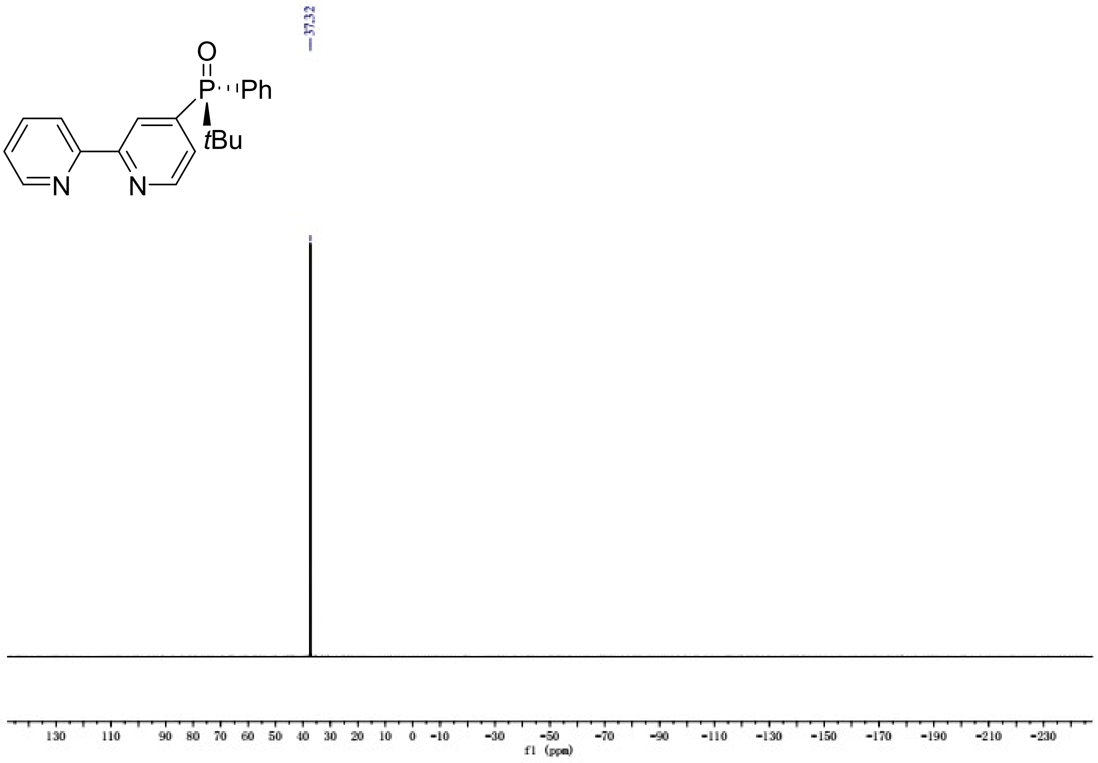

Figure S85. ^{31}P NMR spectrum of **32** in $CDCl_3$

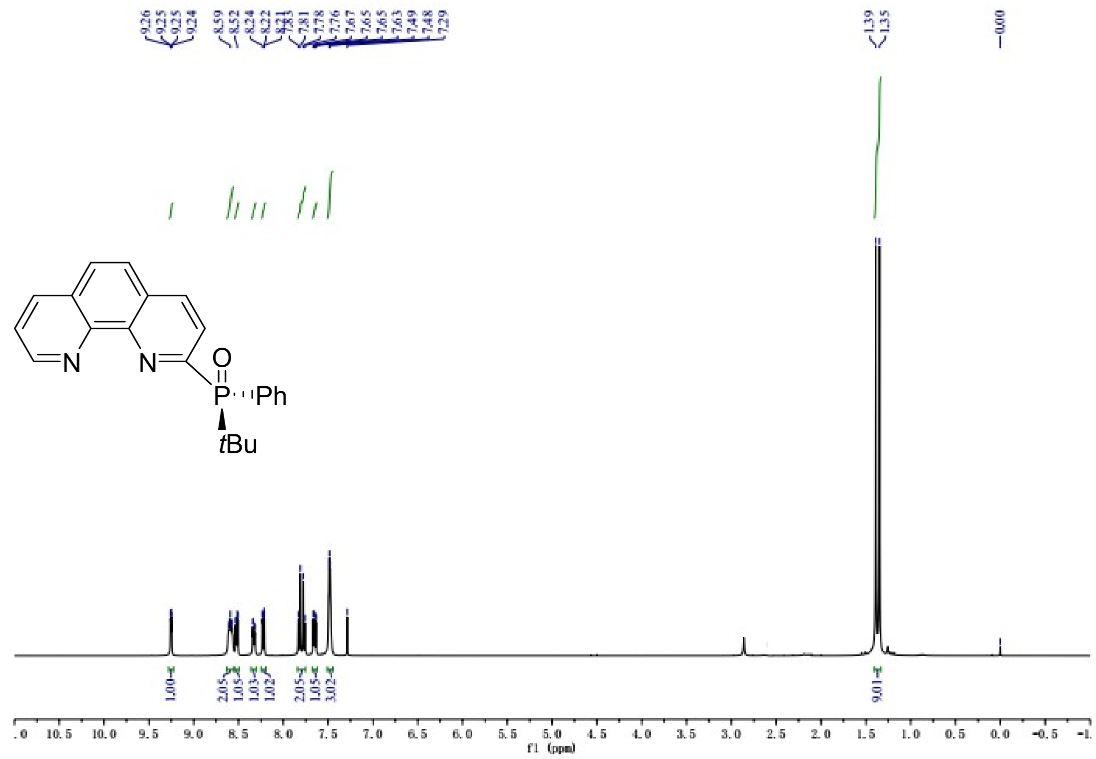

Figure S86. ^{1}H NMR spectrum of **33** in $CDCl_3$

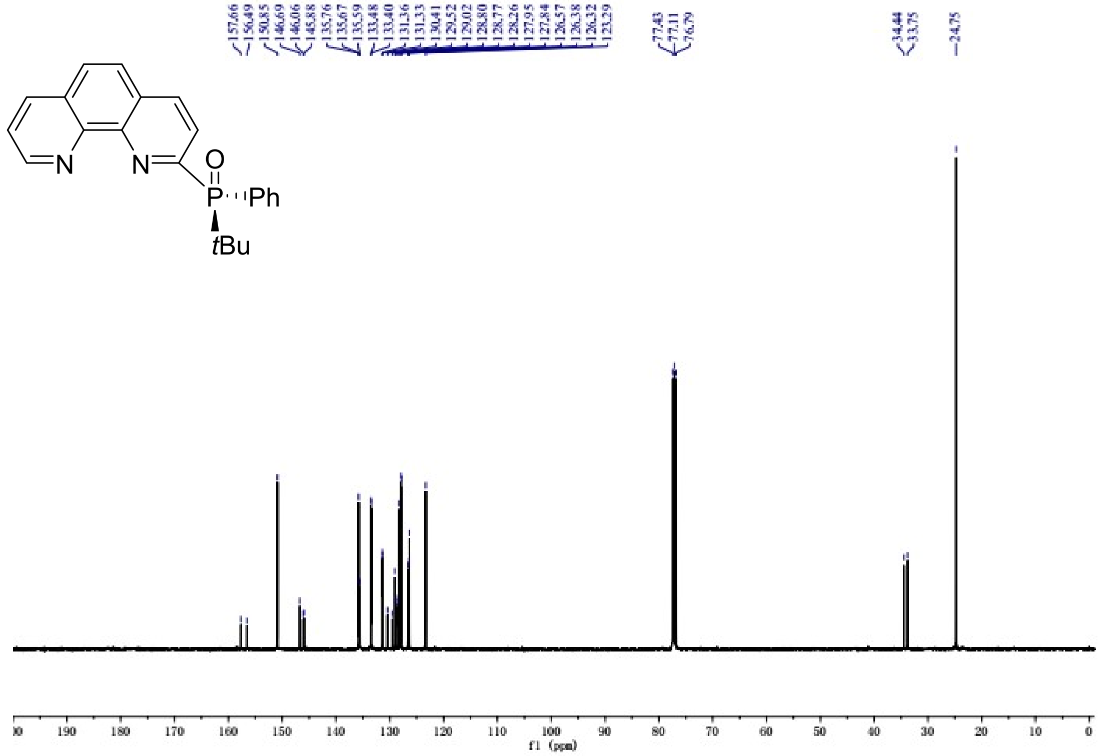

Figure S87. ^{13}C NMR spectrum of **33** in $CDCl_3$

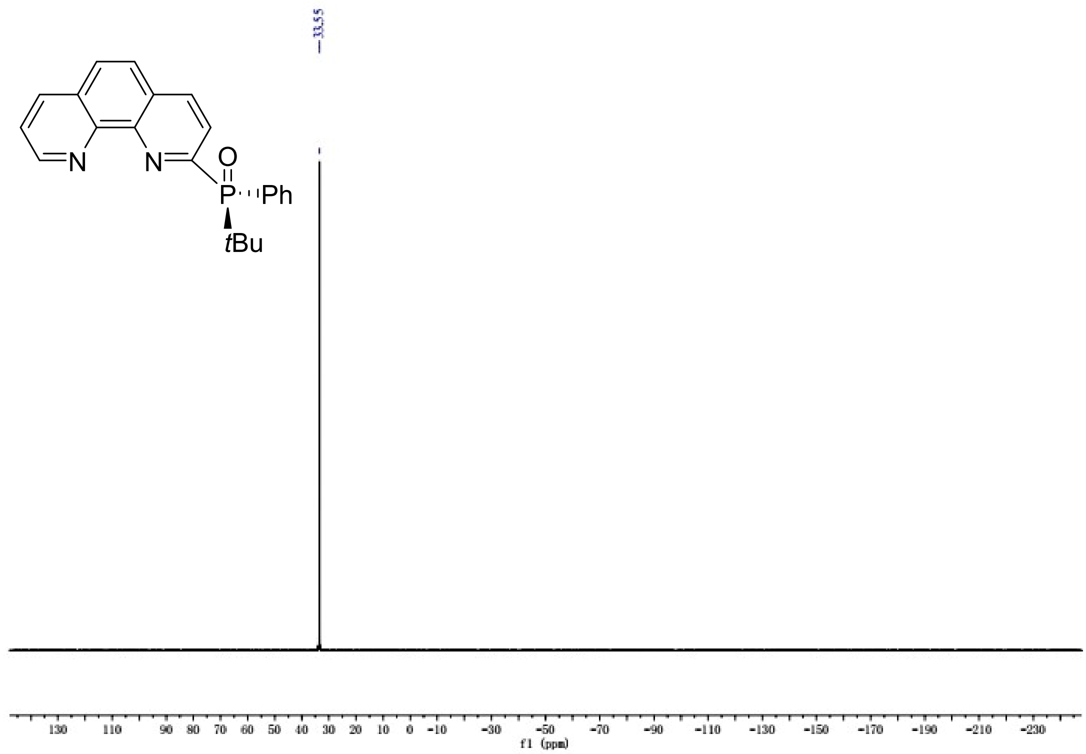

Figure S88. ^{31}P NMR spectrum of **33** in $CDCl_3$

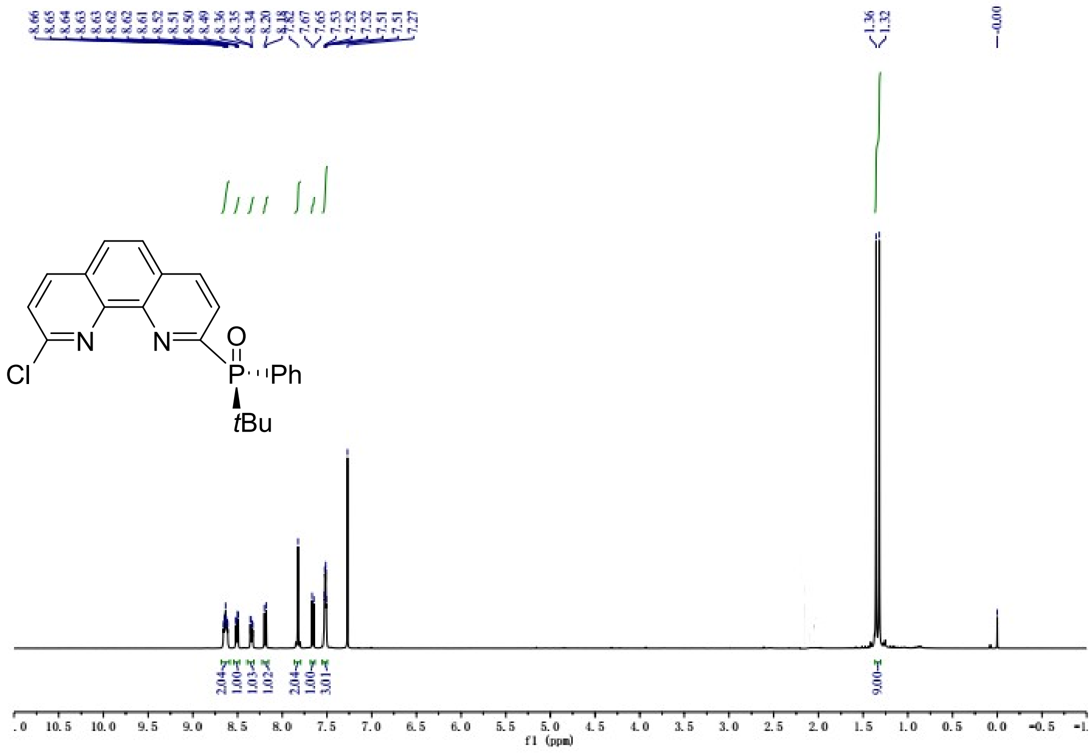

Figure S89. ^{1}H NMR spectrum of **34** in $CDCl_3$

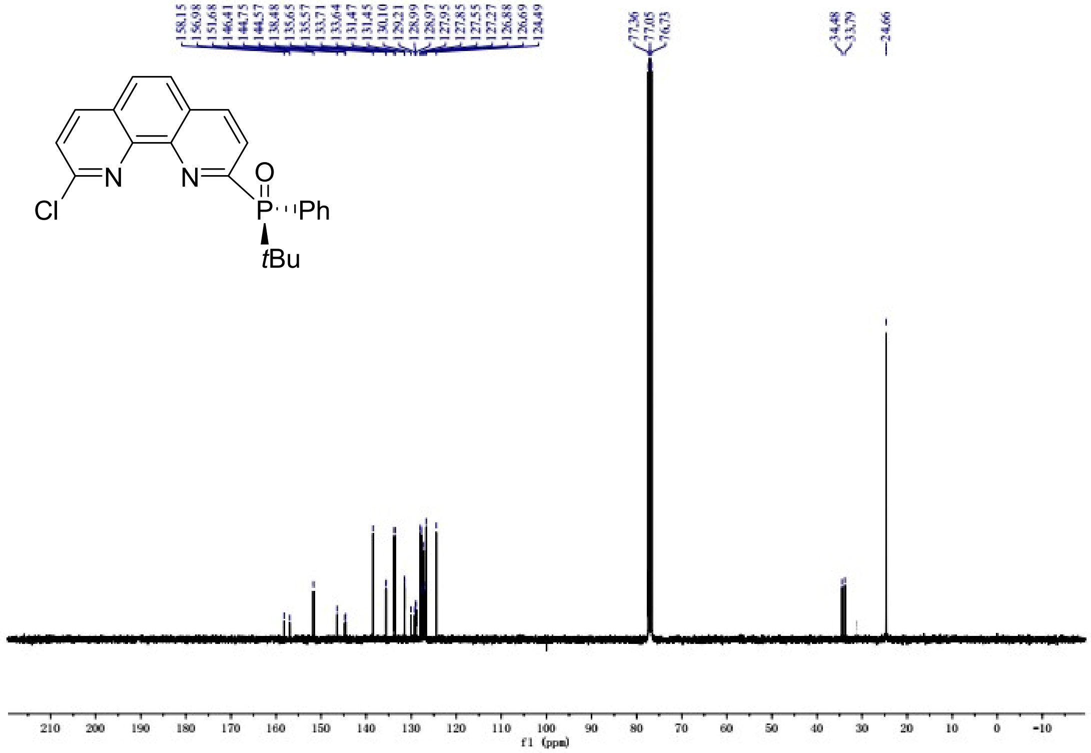

Figure S90. ^{13}C NMR spectrum of **34** in $CDCl_3$

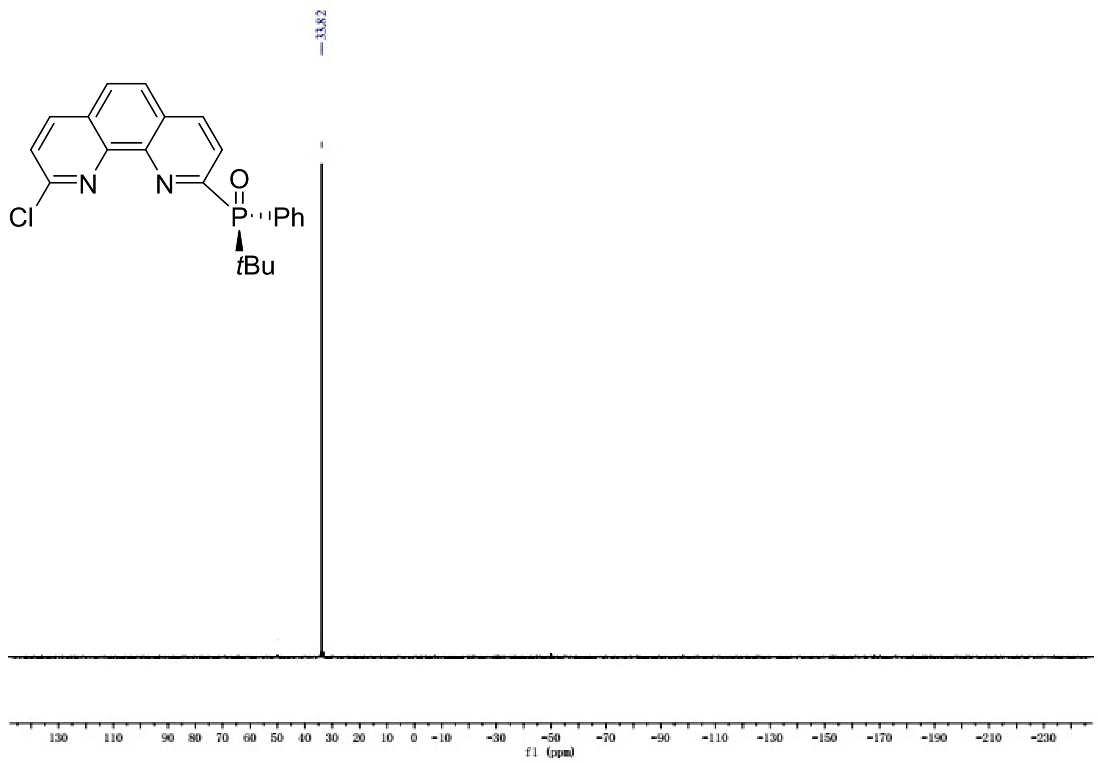

Figure S91. ^{31}P NMR spectrum of **34** in $CDCl_3$

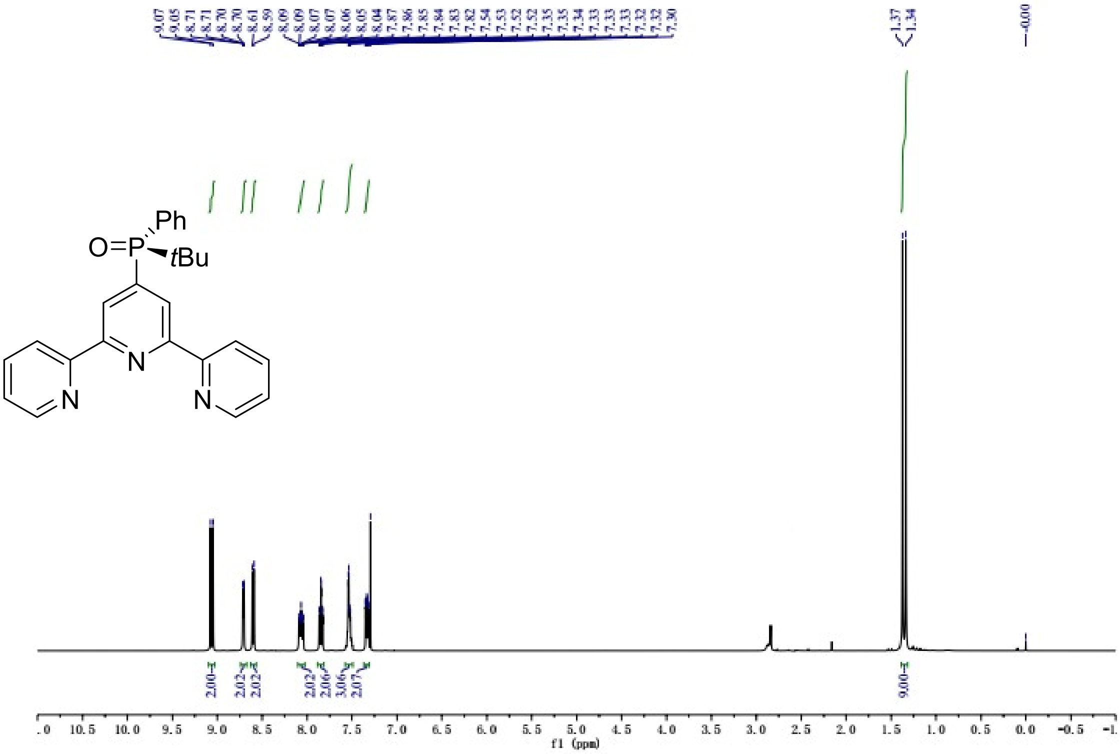

Figure S92. ^{1}H NMR spectrum of **35** in $CDCl_3$

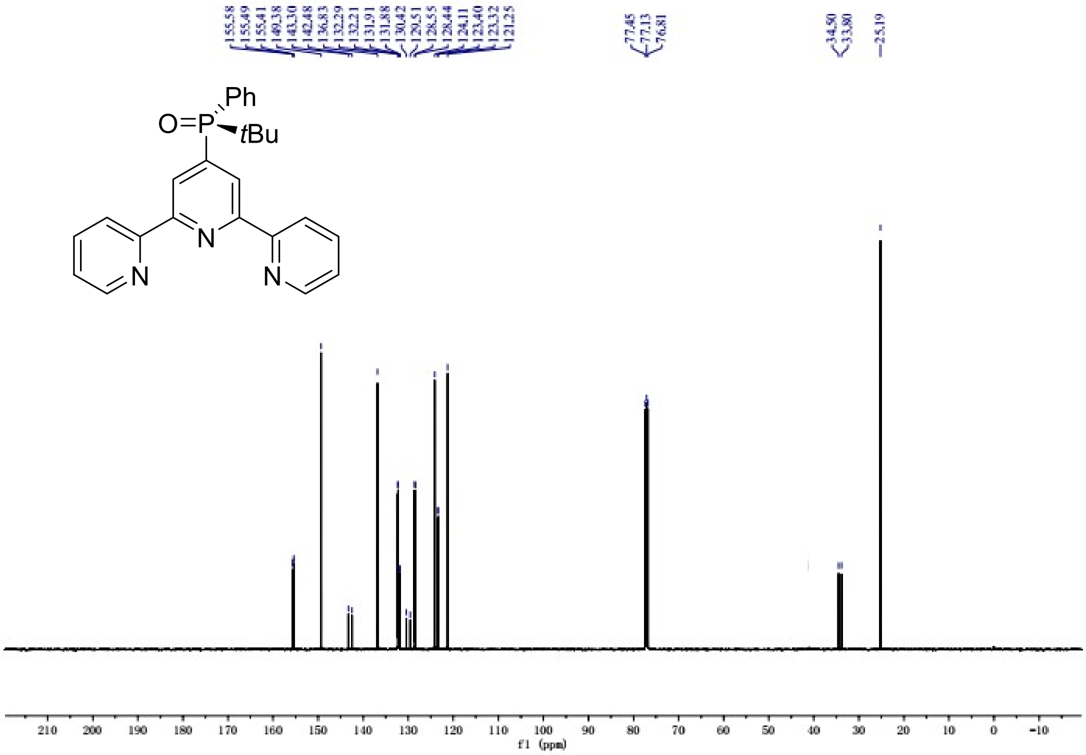

Figure S93. ^{13}C NMR spectrum of **35** in $CDCl_3$

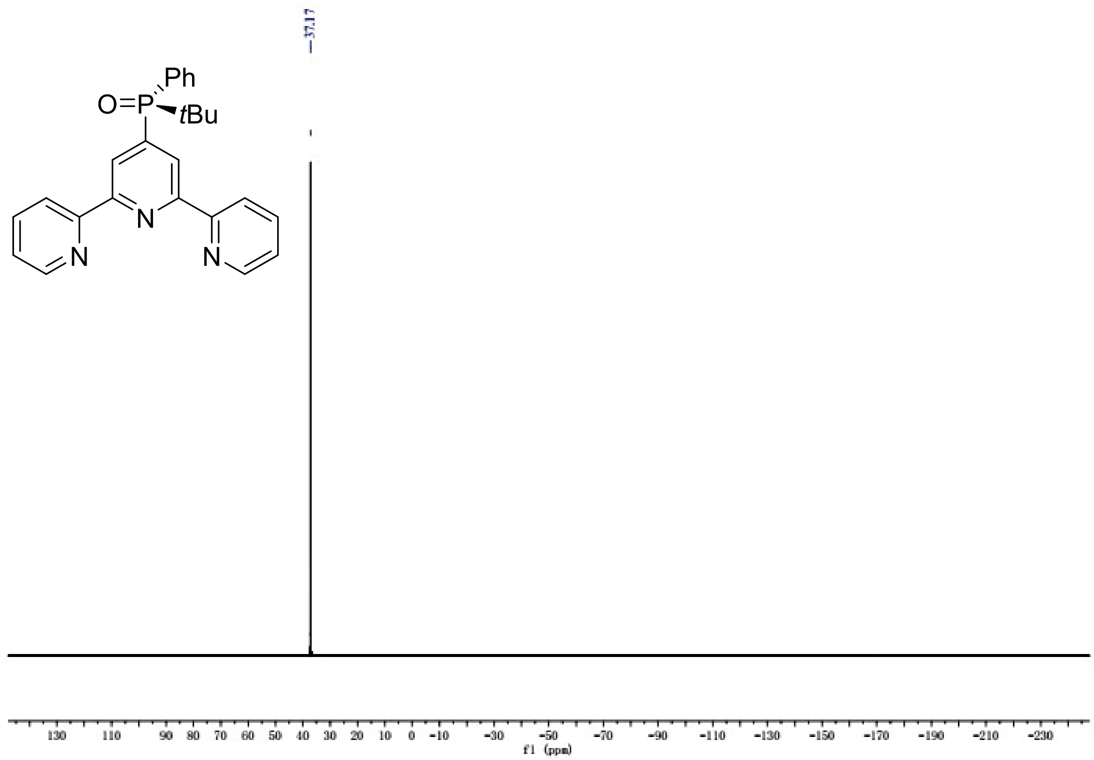

Figure S94. ^{31}P NMR spectrum of **35** in $CDCl_3$

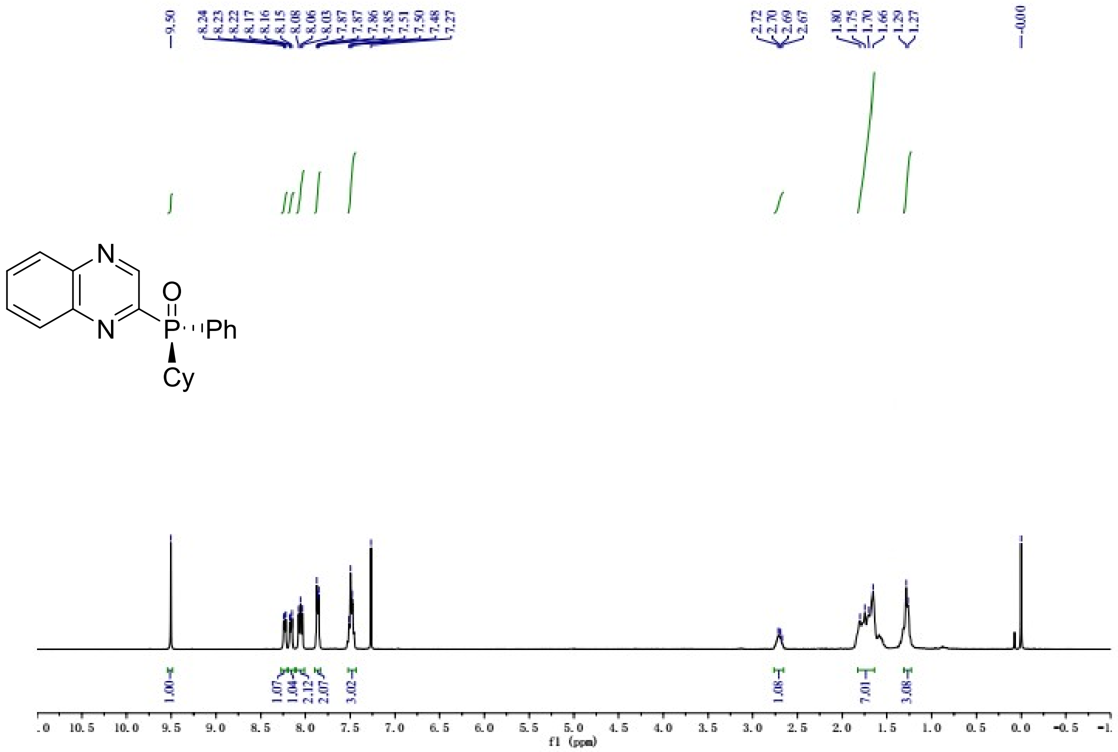

Figure S95. ^{1}H NMR spectrum of **39** in $CDCl_3$

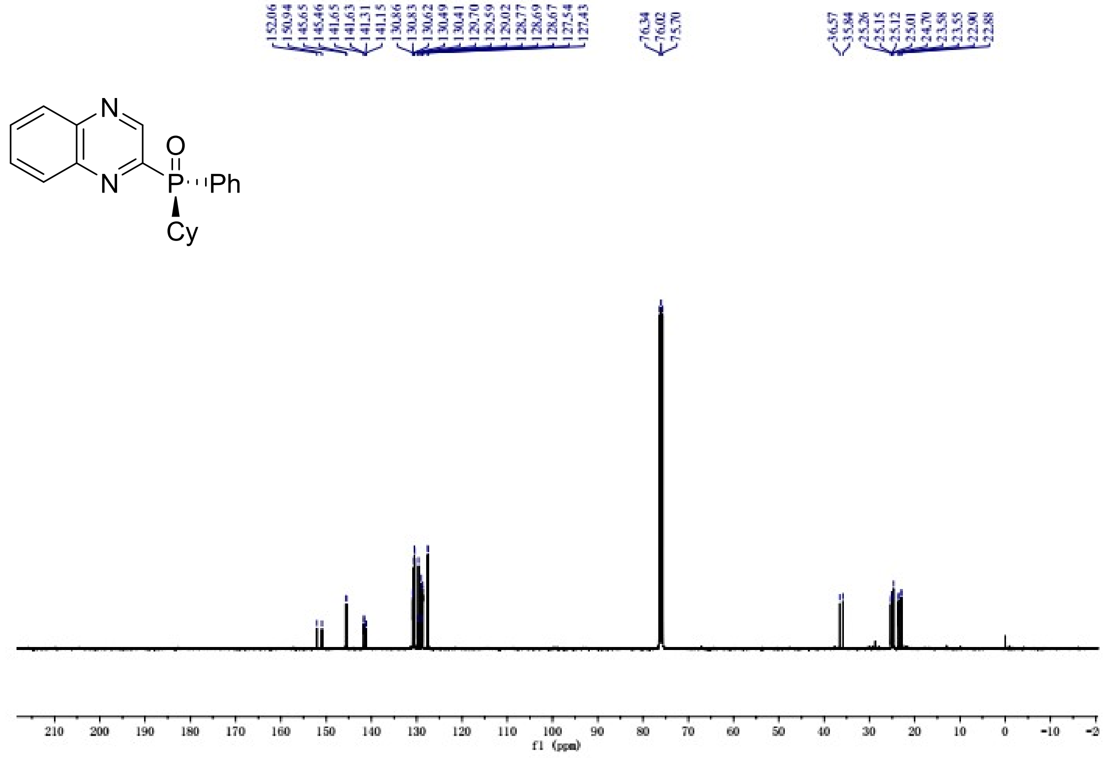

Figure S96. ^{13}C NMR spectrum of **39** in $CDCl_3$

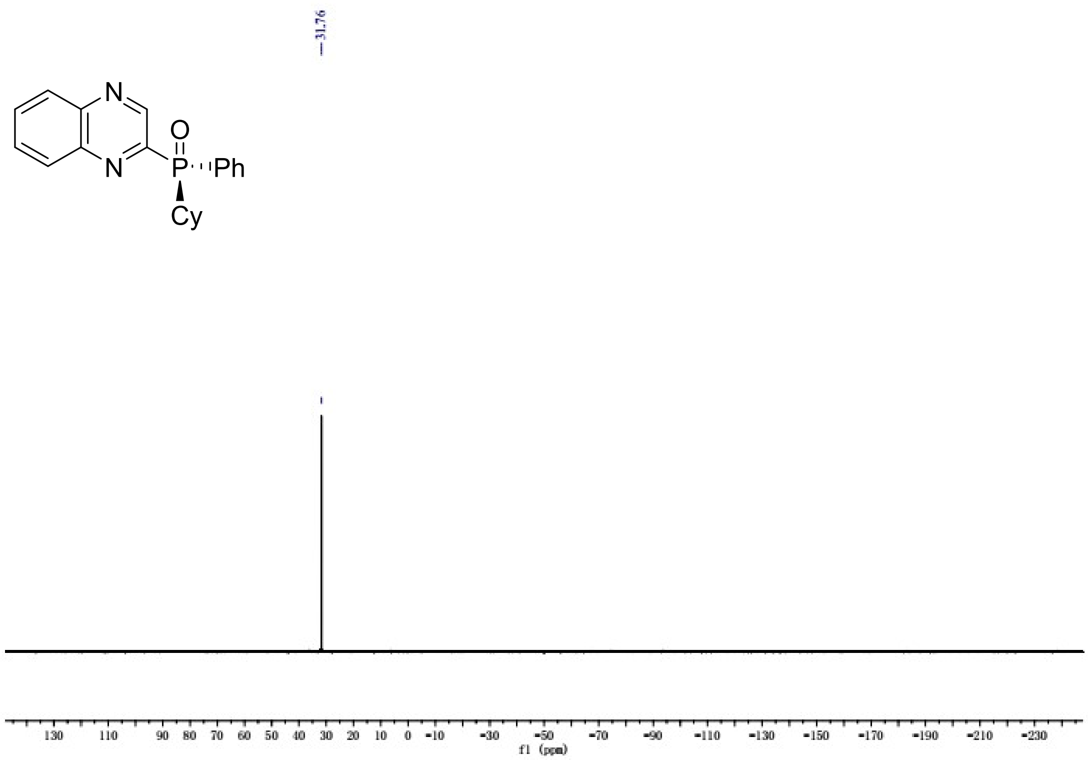

Figure S97. ^{31}P NMR spectrum of **39** in $CDCl_3$

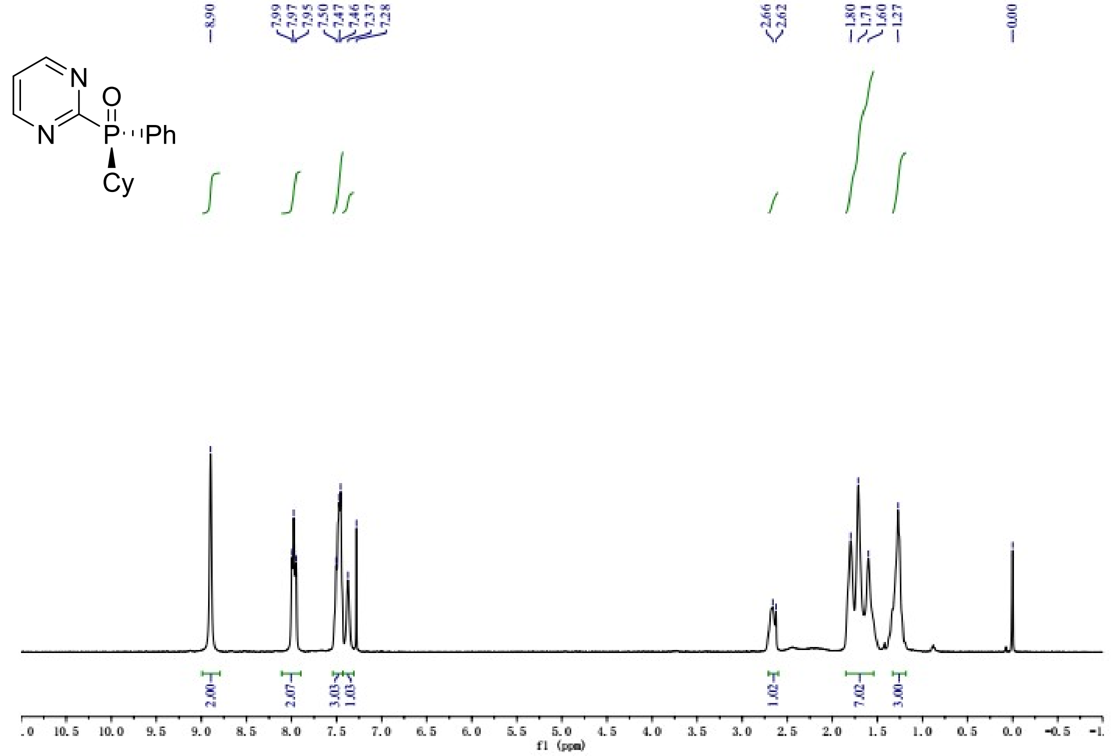

Figure S98. ^{1}H NMR spectrum of **40** in $CDCl_3$

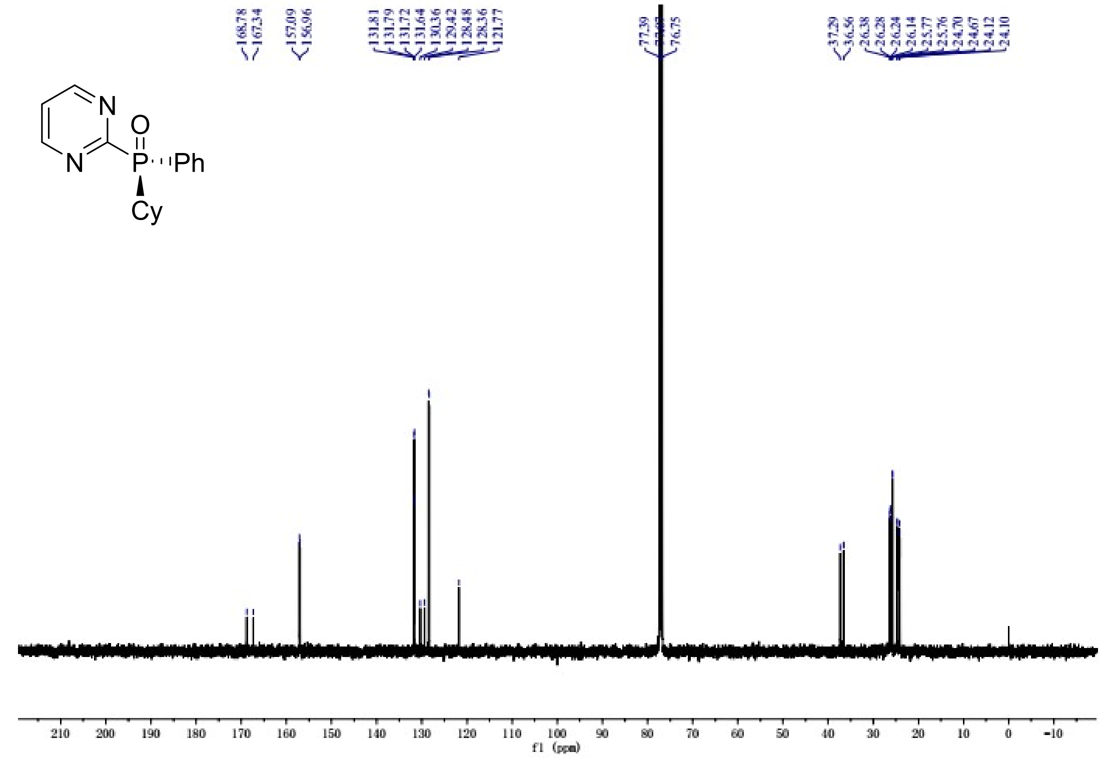

Figure S99. ^{13}C NMR spectrum of **40** in $CDCl_3$

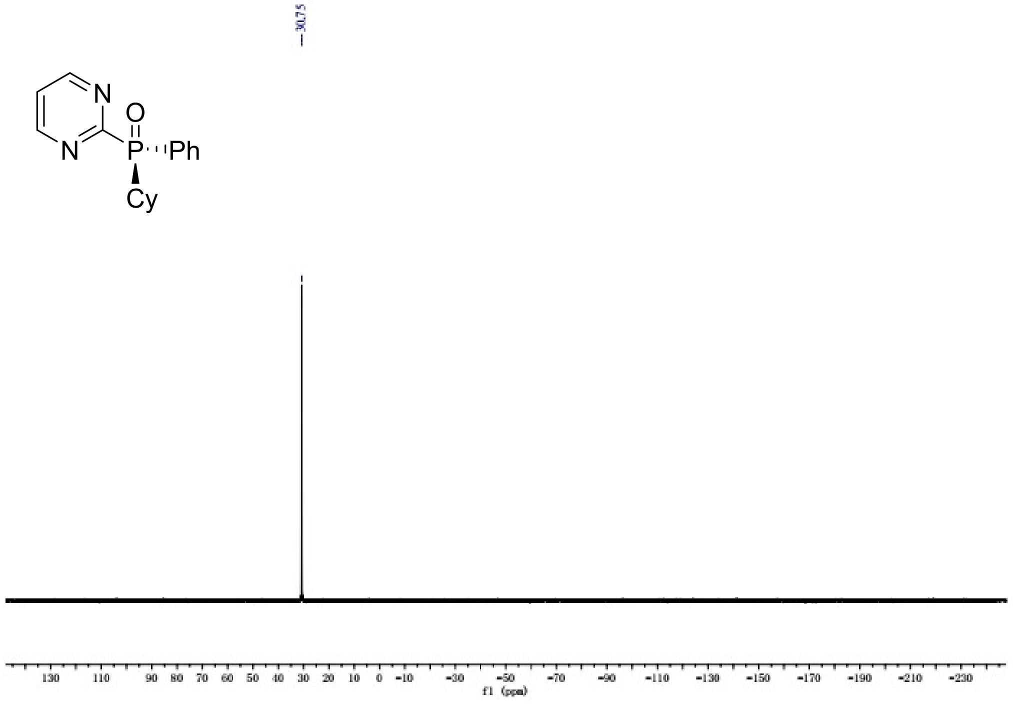

Figure S100. ^{31}P NMR spectrum of **40** in $CDCl_3$

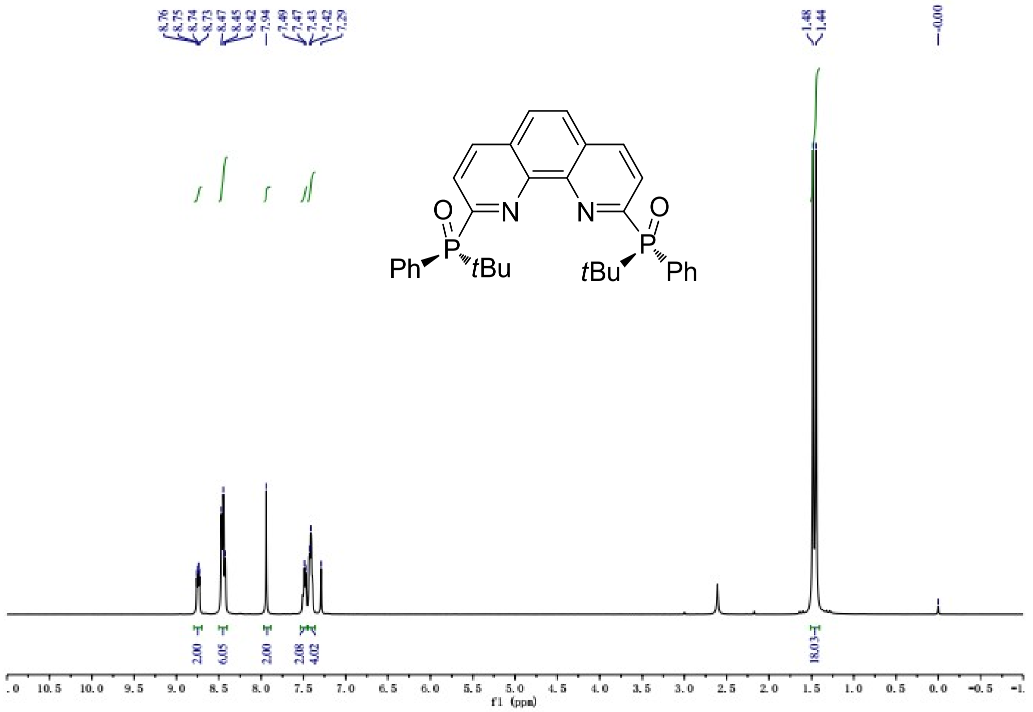

Figure S101. ^{1}H NMR spectrum of **41** in $CDCl_3$

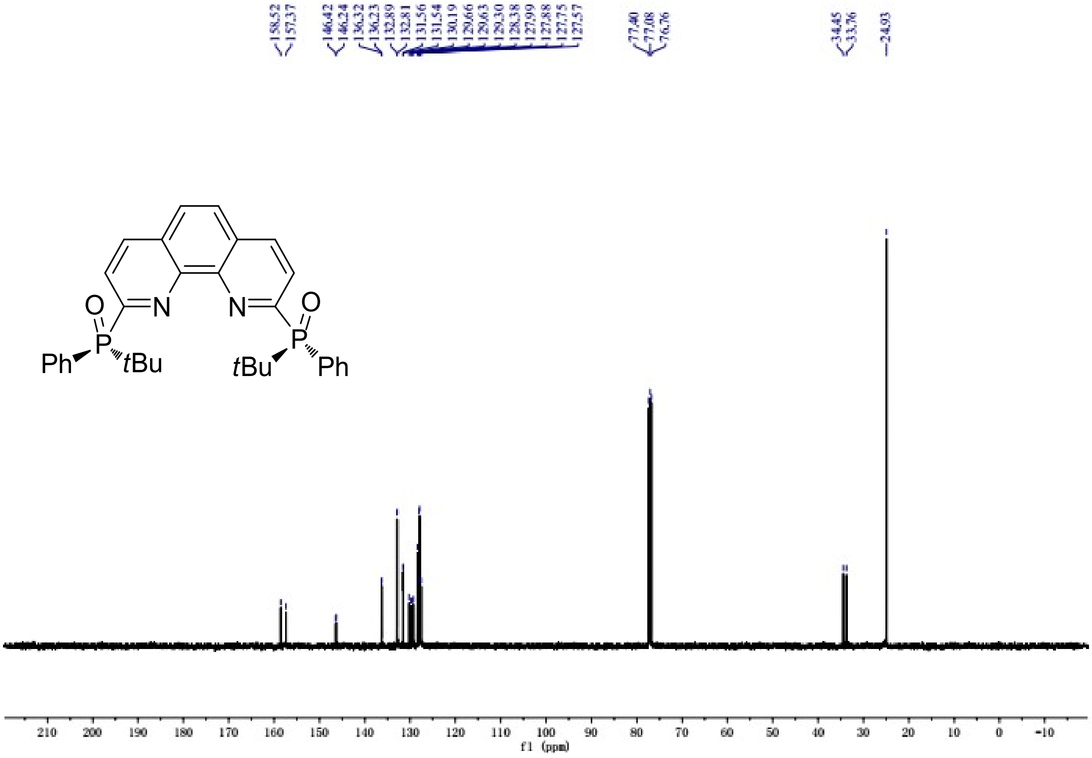

Figure S102. ^{13}C NMR spectrum of **41** in $CDCl_3$

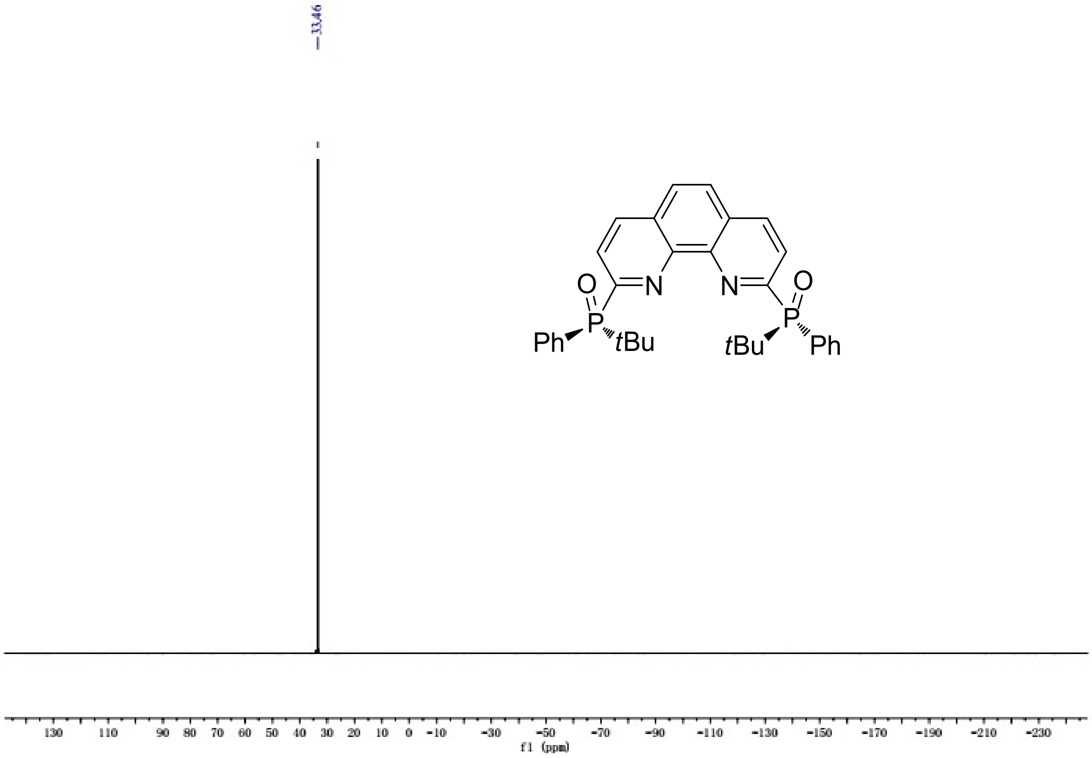

Figure S103. ^{31}P NMR spectrum of **41** in $CDCl_3$

7. HPLC spectra for all products.

Chiral HPLC chromatographic analysis of **4**

Condition: Daicel Chiralcel AD-H, *n*-hexane/*i*-PrOH = 90/10, UV = 254 nm, flow rate: 1.0 mL/min, retention time: t (minor) =12.392 min, t (major) = 13.512 min, ee = 98%.

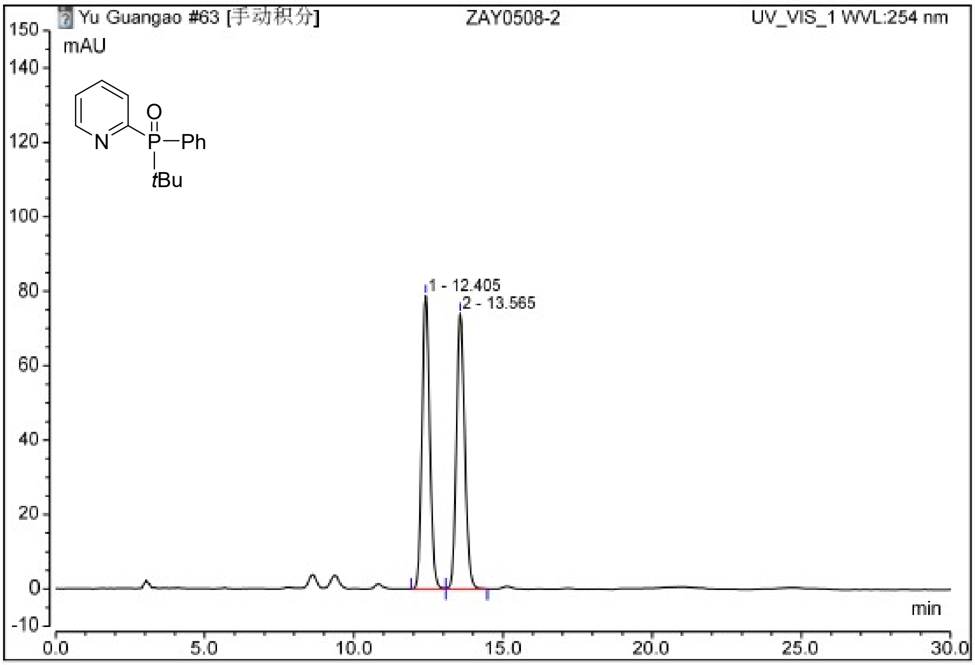

peak.number	peak.name min	peak.area mAU*min	peak.height mAU	peak.rel_area %	peak.rel_height %
1	12.405	23.774	78.791	49.53	51.55
2	13.565	24.225	74.052	50.47	48.45

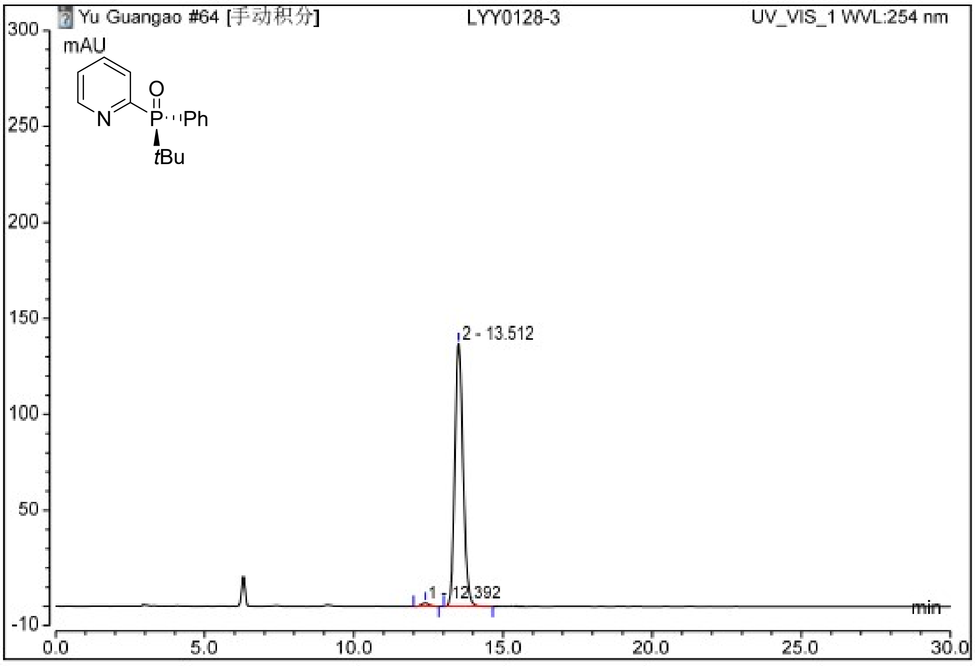

peak.number	peak.name min	peak.area mAU*min	peak.height mAU	peak.rel_area %	peak.rel_height %
1	12.392	0.532	1.780	1.19	1.28
2	13.512	44.040	136.898	98.81	98.72

Chiral HPLC chromatographic analysis of **5**

Condition: Daicel Chiralcel AD-H, *n*-hexane/*i*-PrOH = 85/15, UV = 254 nm, flow rate: 1.0 mL/min, retention time: t (major) =5.965 min, t (minor) = 6.979 min, ee = 97%.

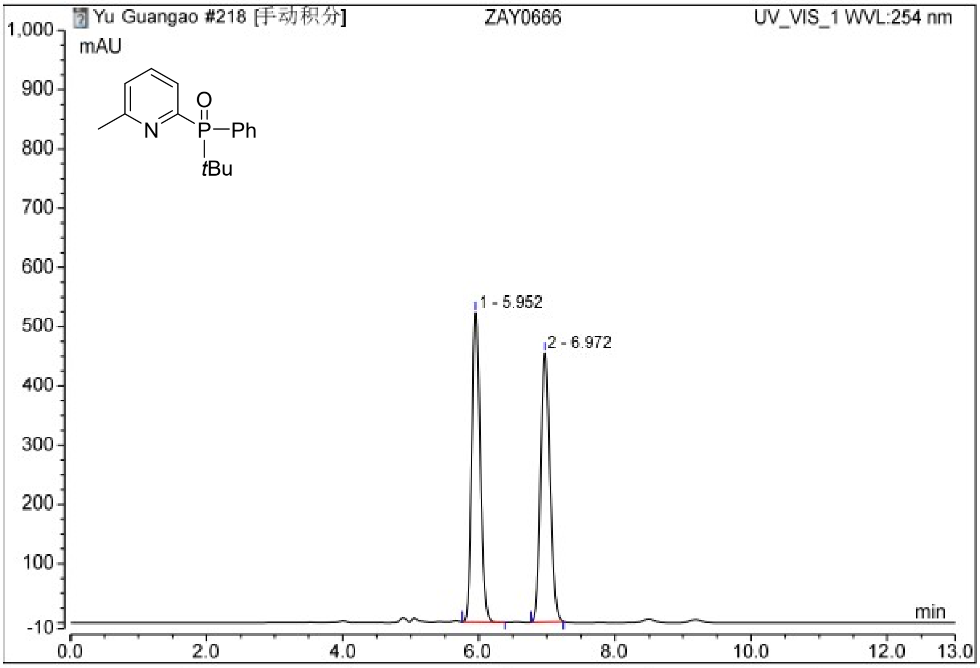

peak.number	peak.name<br>min	peak.area<br>mAU*min	peak.height<br>mAU	peak.rel_area<br>%	peak.rel_height<br>%
1	5.952	73.008	522.497	50.06	53.52
2	6.972	72.839	453.699	49.94	46.48

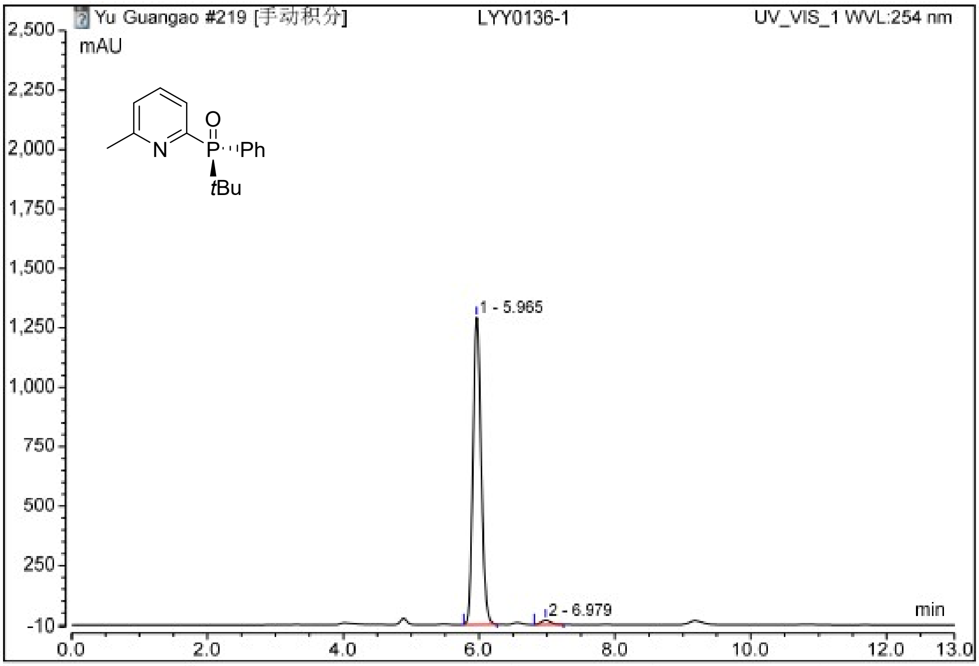

peak.number	peak.name<br>min	peak.area<br>mAU*min	peak.height<br>mAU	peak.rel_area<br>%	peak.rel_height<br>%
1	5.965	180.567	1292.598	98.42	98.55
2	6.979	2.896	18.955	1.58	1.45

Chiral HPLC chromatographic analysis of **6**

Condition: Daicel Chiralcel AD-H, *n*-hexane/*i*-PrOH = 85/15, UV = 254 nm, flow rate: 1.0 mL/min, retention time: t (major) =11.099 min, t (minor) = 22.752 min, ee = 99%.

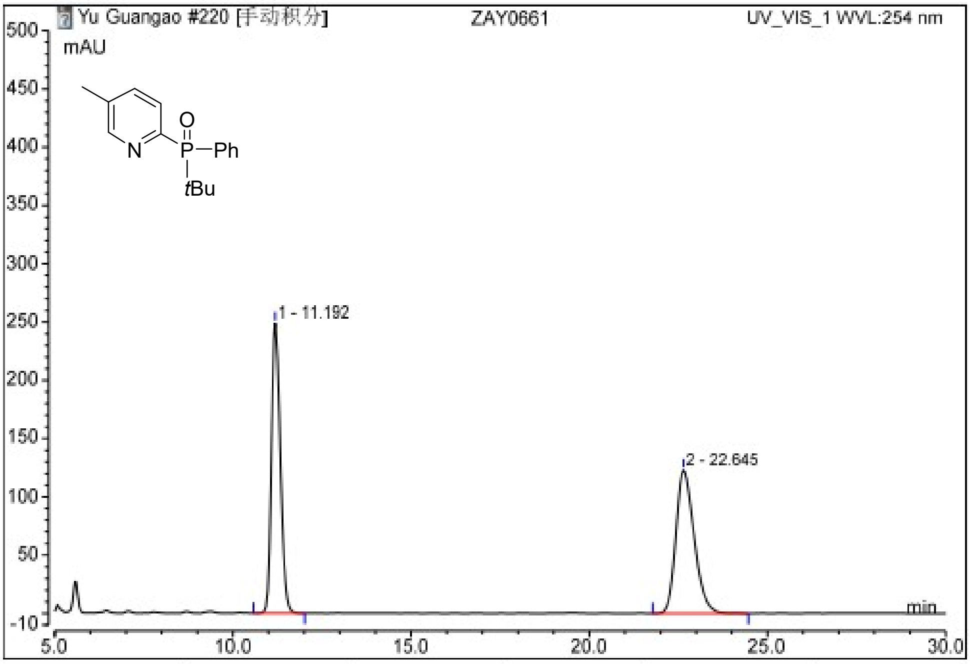

peak.number	peak.name min	peak.area mAU*min	peak.height mAU	peak.rel_area %	peak.rel_height %
1	11.192	72.488	249.047	49.91	66.94
2	22.645	72.753	123.020	50.09	33.06

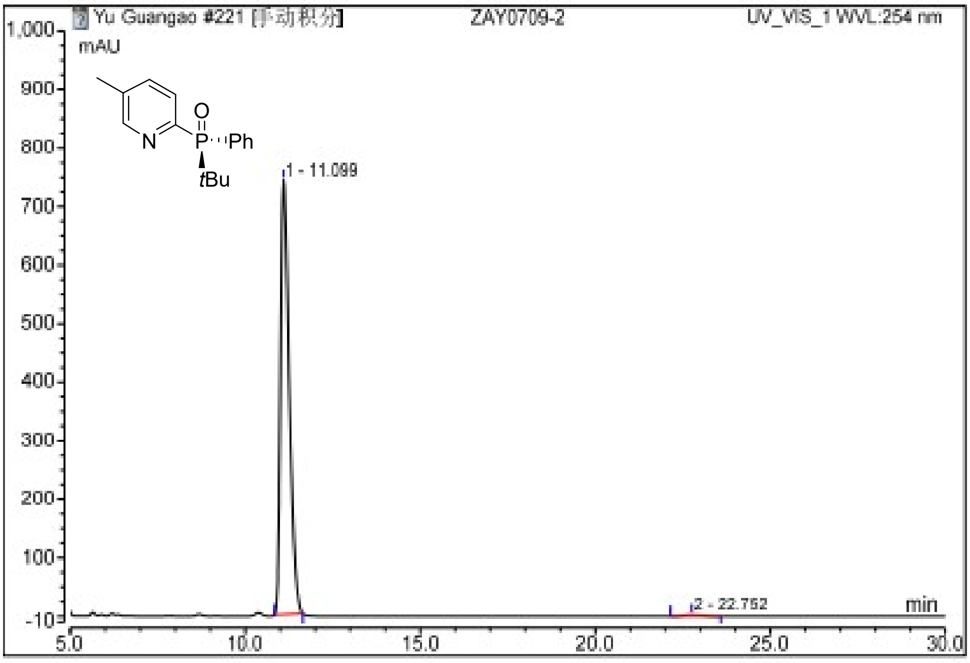

peak.number	peak.name min	peak.area mAU*min	peak.height mAU	peak.rel_area %	peak.rel_height %
1	11.099	213.330	741.574	99.34	99.69
2	22.752	1.423	2.330	0.66	0.31

Chiral HPLC chromatographic analysis of **7**

Condition: Daicel Chiralcel AD-H, *n*-hexane/*i*-PrOH = 98/2, UV = 254 nm, flow rate: 1.0 mL/min, retention time: t (minor) =42.386 min, t (major) = 45.219 min, ee = 99%.

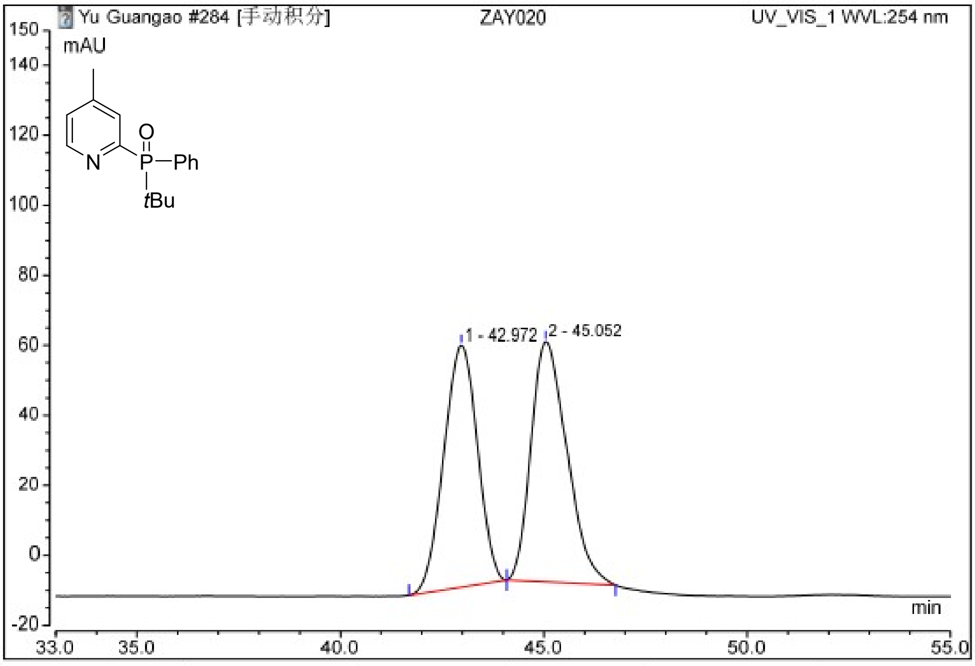

peak.number	peak.name min	peak.area mAU*min	peak.height mAU	peak.rel_area %	peak.rel_height %
1	42.972	66.304	69.123	48.49	50.17
2	45.052	70.443	68.655	51.51	49.83

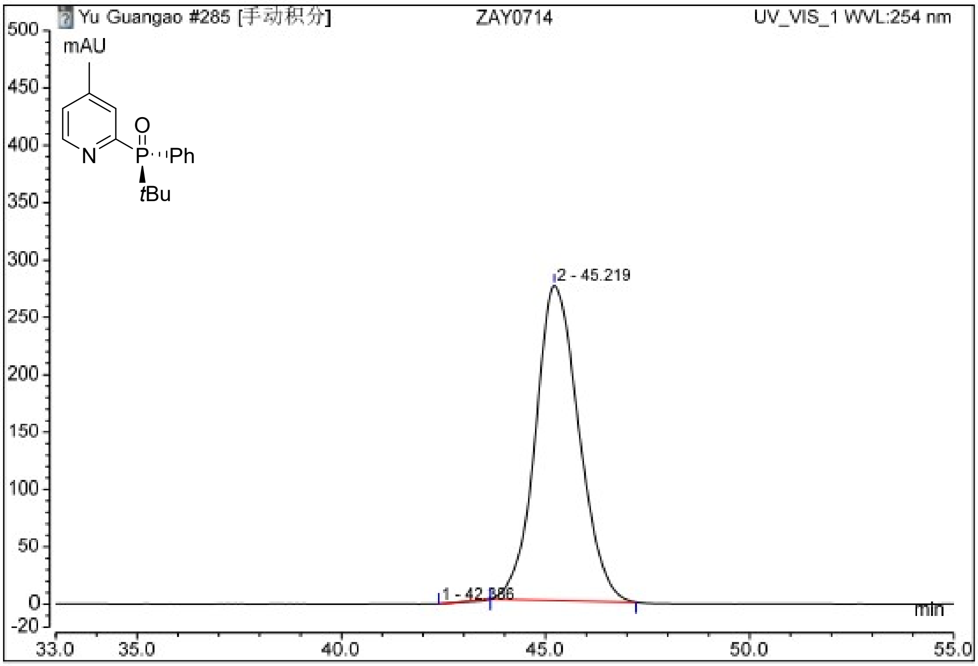

peak.number	peak.name min	peak.area mAU*min	peak.height mAU	peak.rel_area %	peak.rel_height %
1	42.386	0.521	0.000	0.15	0.00
2	45.219	339.277	274.861	99.85	100.00

Chiral HPLC chromatographic analysis of **8**

Condition: Daicel Chiralcel AD-H, *n*-hexane/*i*-PrOH = 85/15, UV = 254 nm, flow rate: 1.0 mL/min, retention time: t (major) =4.832 min, t (minor) = 5.279 min, ee = 99%.

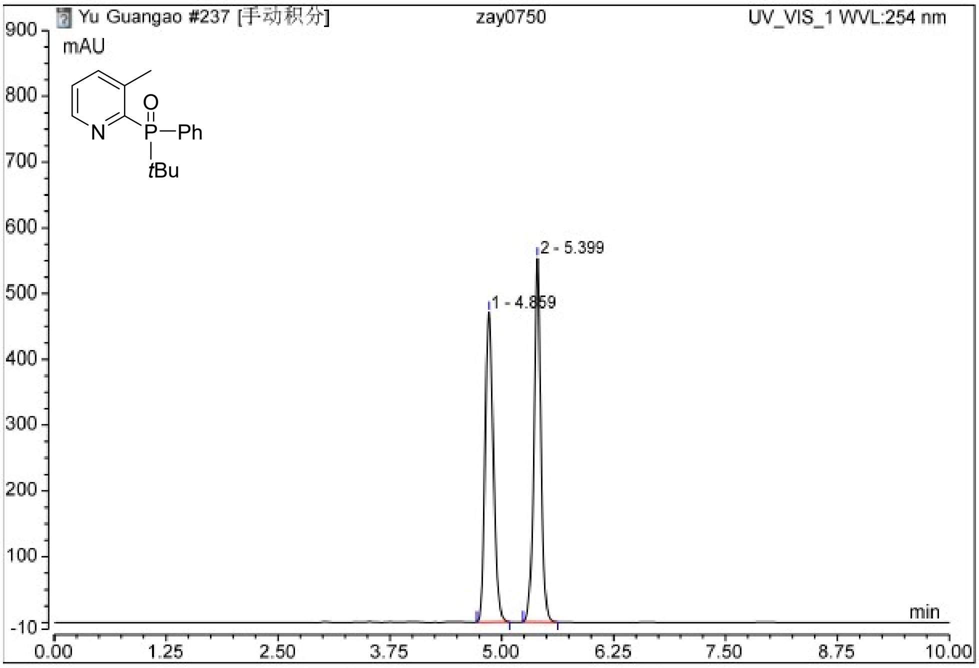

peak.number	peak.name min	peak.area mAU*min	peak.height mAU	peak.rel_area %	peak.rel_height %
1	4.859	50.659	470.927	50.79	46.00
2	5.399	49.093	552.726	49.21	54.00

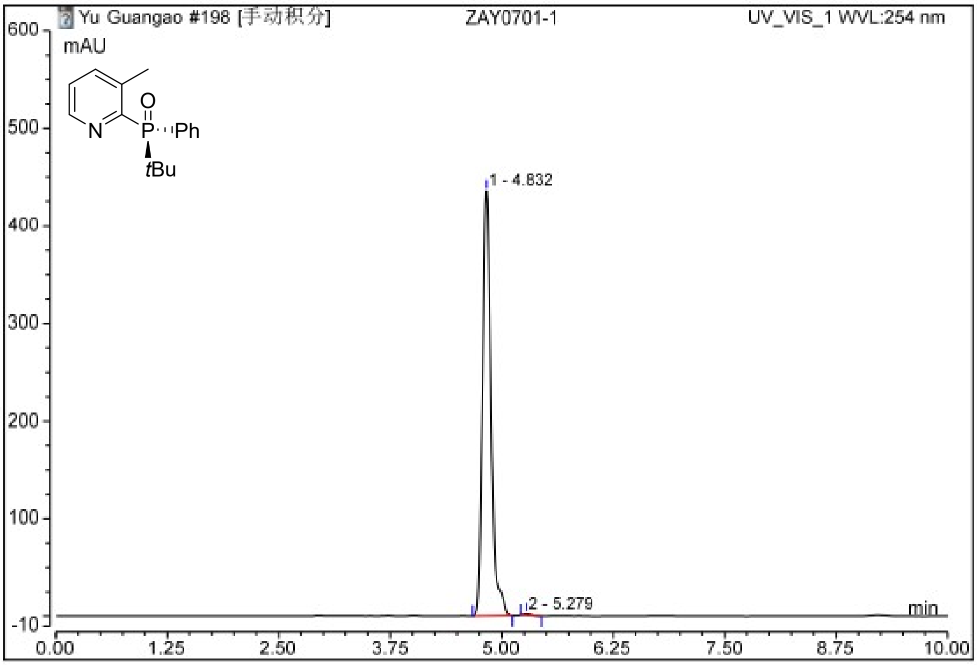

peak.number	peak.name min	peak.area mAU*min	peak.height mAU	peak.rel_area %	peak.rel_height %
1	4.832	48.837	435.702	99.66	99.60
2	5.279	0.166	1.737	0.34	0.40

Chiral HPLC chromatographic analysis of **9**

Condition: Daicel Chiralcel AD-H, *n*-hexane/*i*-PrOH = 85/15, UV = 254 nm, flow rate: 1.0 mL/min, retention time: t (major) =5.652 min, t (minor) = 6.192 min, ee = 99%.

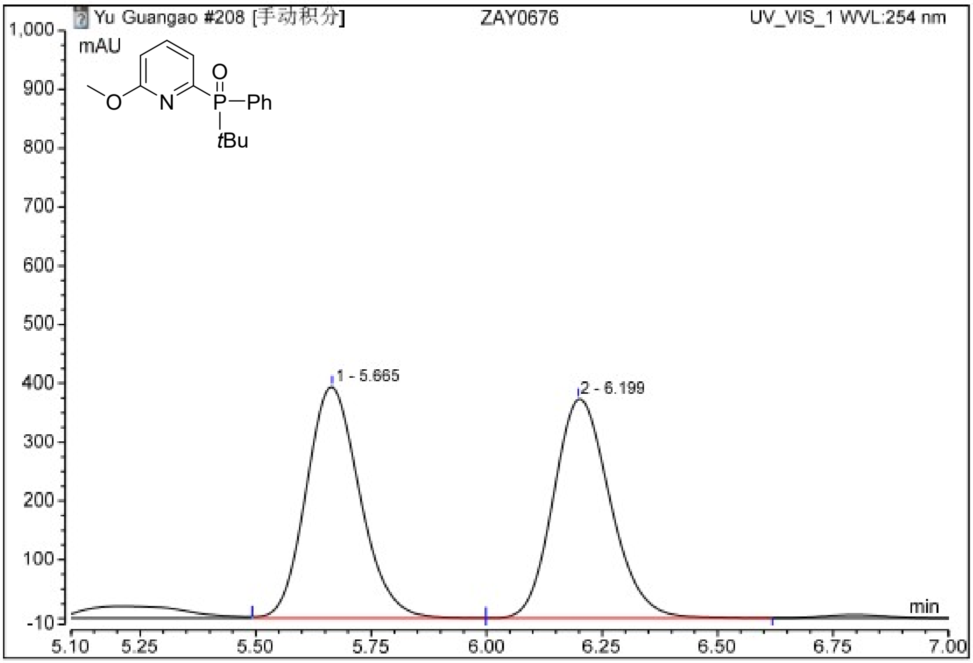

peak.number	peak.name min	peak.area mAU*min	peak.height mAU	peak.rel_area %	peak.rel_height %
1	5.665	53.015	393.735	49.94	51.35
2	6.199	53.132	373.098	50.06	48.65

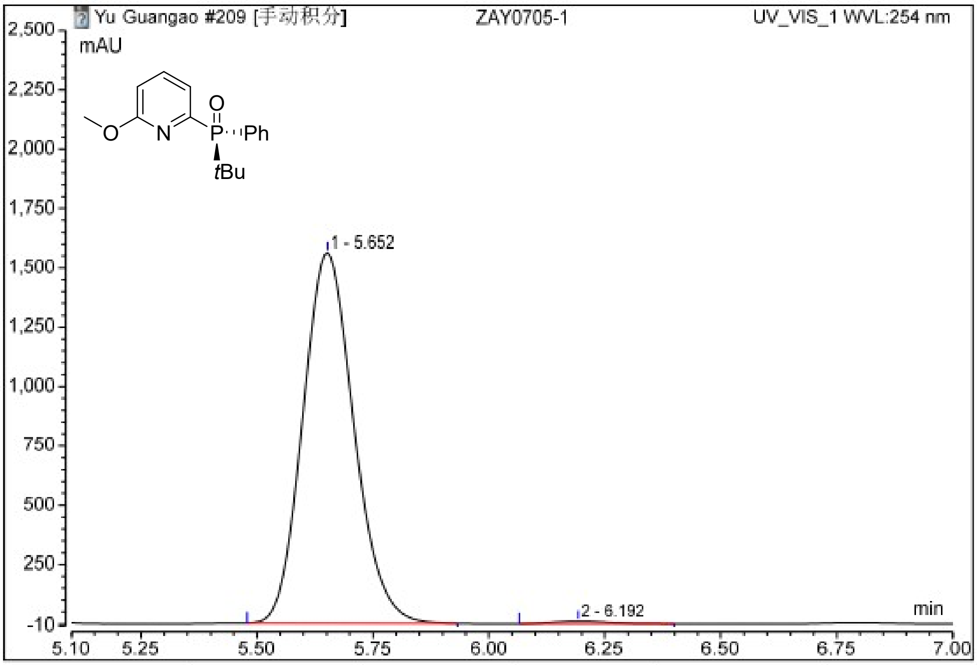

peak.number	peak.name min	peak.area mAU*min	peak.height mAU	peak.rel_area %	peak.rel_height %
1	5.652	198.766	1560.533	99.35	99.38
2	6.192	1.310	9.690	0.65	0.62

Chiral HPLC chromatographic analysis of **12**

Condition: Daicel Chiralcel AD-H, *n*-hexane/*i*-PrOH = 85/15, UV = 254 nm, flow rate: 1.0 mL/min, retention time: t (minor) =9.312 min, t (major) = 19.099 min, ee = 98%.

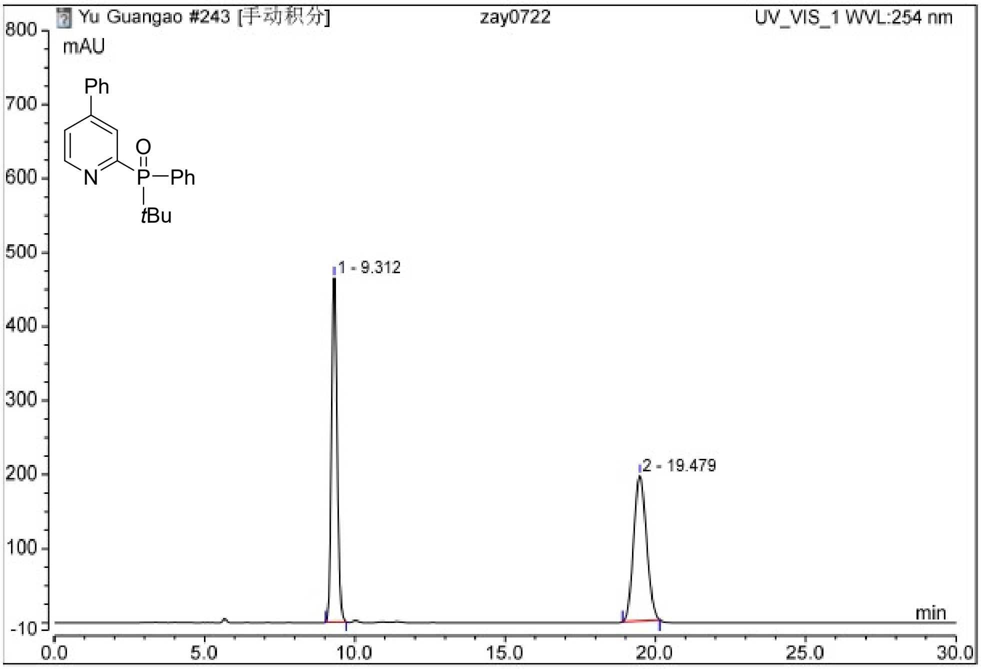

peak.number	peak.name min	peak.area mAU*min	peak.height mAU	peak.rel_area %	peak.rel_height %
1	9.312	99.021	464.847	50.54	70.32
2	19.479	96.886	196.241	49.46	29.68

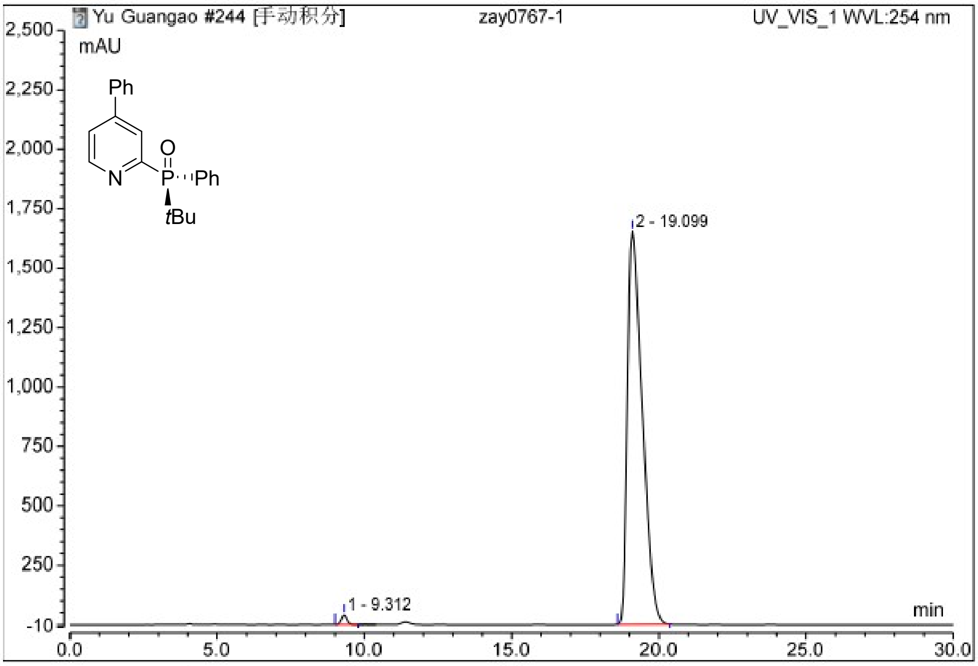

peak.number	peak.name min	peak.area mAU*min	peak.height mAU	peak.rel_area %	peak.rel_height %
1	9.312	9.157	41.088	0.94	2.42
2	19.099	968.020	1654.277	99.06	97.58

Chiral HPLC chromatographic analysis of **13**

Condition: Daicel Chiralcel AD-H, *n*-hexane/*i*-PrOH = 85/15, UV = 254 nm, flow rate: 1.0 mL/min, retention time: t (major) =7.739 min, t (minor) = 8.505 min, ee = 99%.

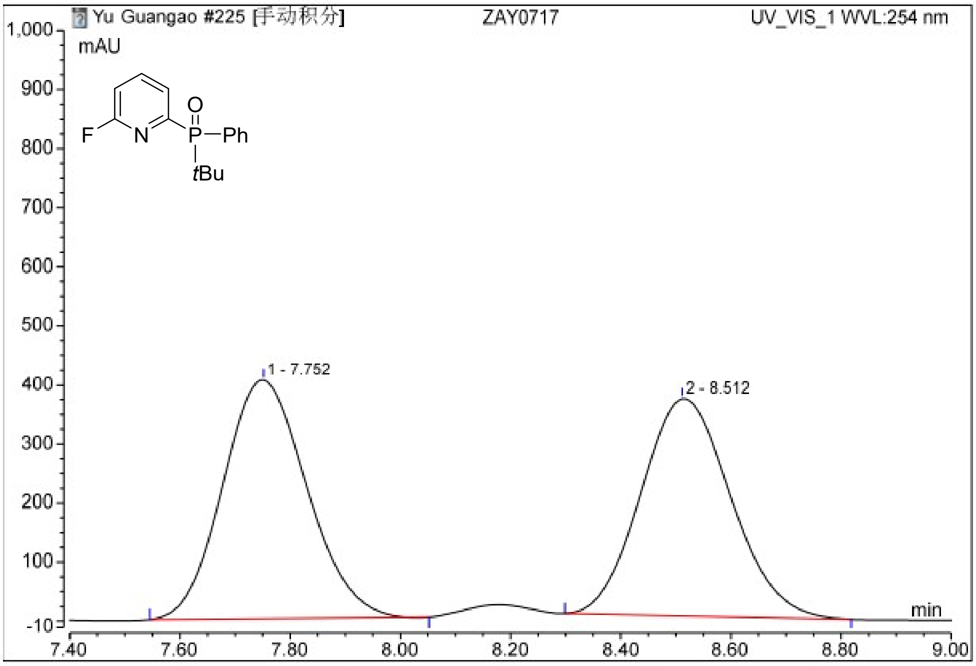

peak.number	peak.name min	peak.area mAU*min	peak.height mAU	peak.rel_area %	peak.rel_height %
1	7.752	71.321	404.364	50.60	52.37
2	8.512	69.638	367.818	49.40	47.63

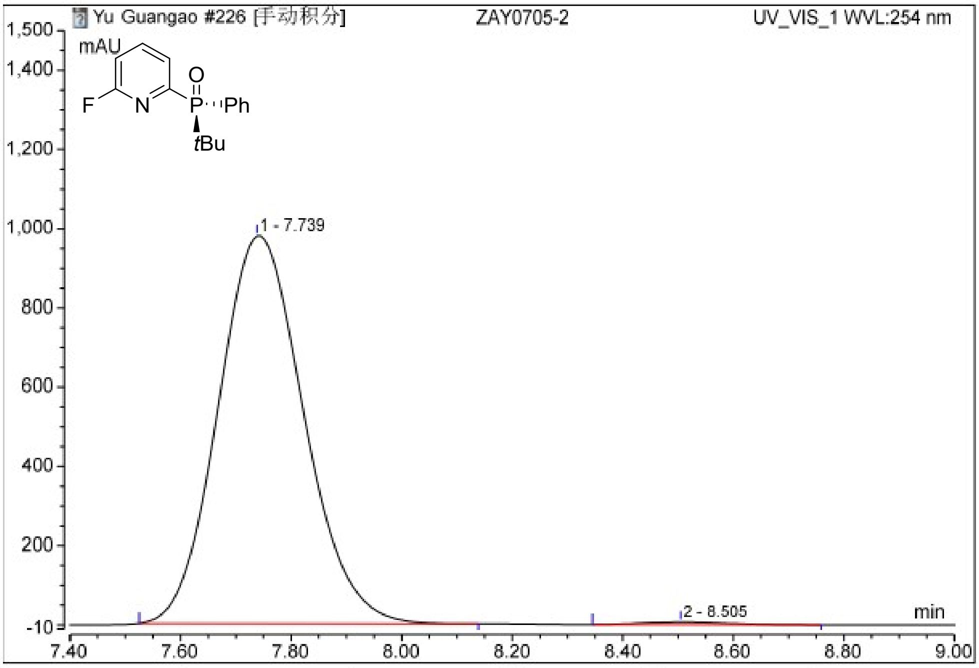

peak.number	peak.name min	peak.area mAU*min	peak.height mAU	peak.rel_area %	peak.rel_height %
1	7.739	175.439	978.481	99.37	99.36
2	8.505	1.112	6.305	0.63	0.64

Chiral HPLC chromatographic analysis of **14**

Condition: Daicel Chiralcel AD-H, *n*-hexane/*i*-PrOH = 85/15, UV = 254 nm, flow rate: 1.0 mL/min, retention time: t (major) =5.925 min, t (minor) = 6.412 min, ee = 99%.

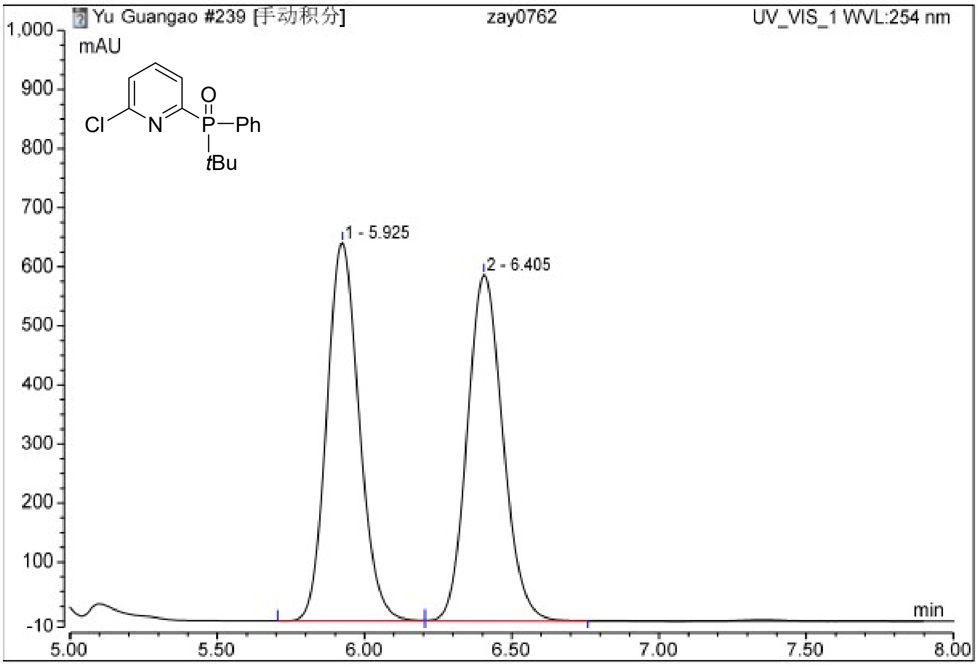

peak.number	peak.name min	peak.area mAU*min	peak.height mAU	peak.rel_area %	peak.rel_height %
1	5.925	81.647	640.136	49.88	52.20
2	6.405	82.048	586.116	50.12	47.80

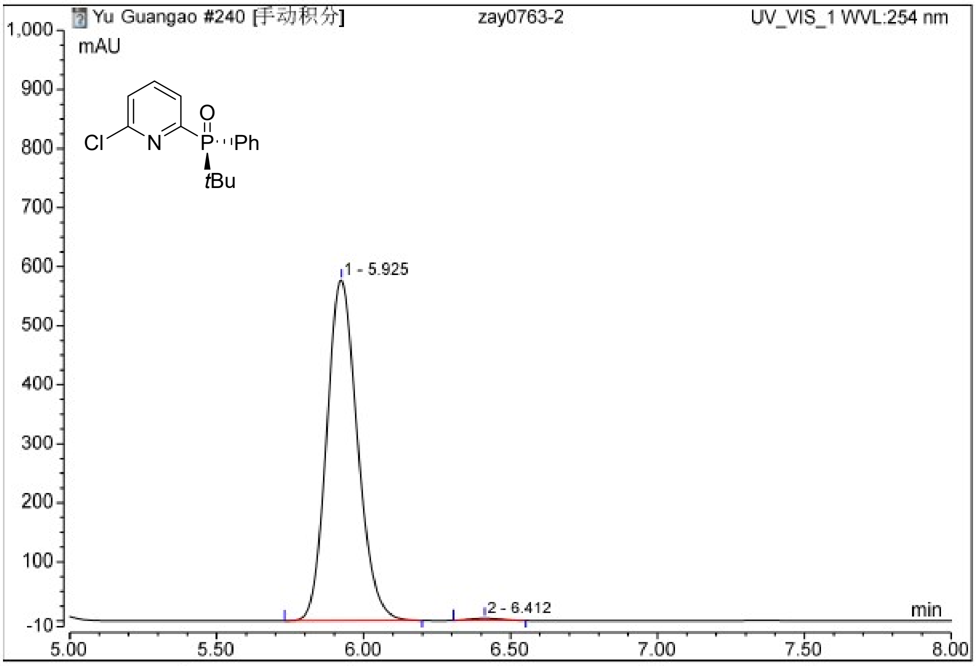

peak.number	peak.name min	peak.area mAU*min	peak.height mAU	peak.rel_area %	peak.rel_height %
1	5.925	70.166	577.040	99.35	99.34
2	6.412	0.461	3.845	0.65	0.66

Chiral HPLC chromatographic analysis of **15**

Condition: Daicel Chiralcel AD-H, *n*-hexane/*i*-PrOH = 90/10, UV = 254 nm, flow rate: 1.0 mL/min, retention time: t (major) =7.925 min, t (minor) = 22.659 min, ee = 97%.

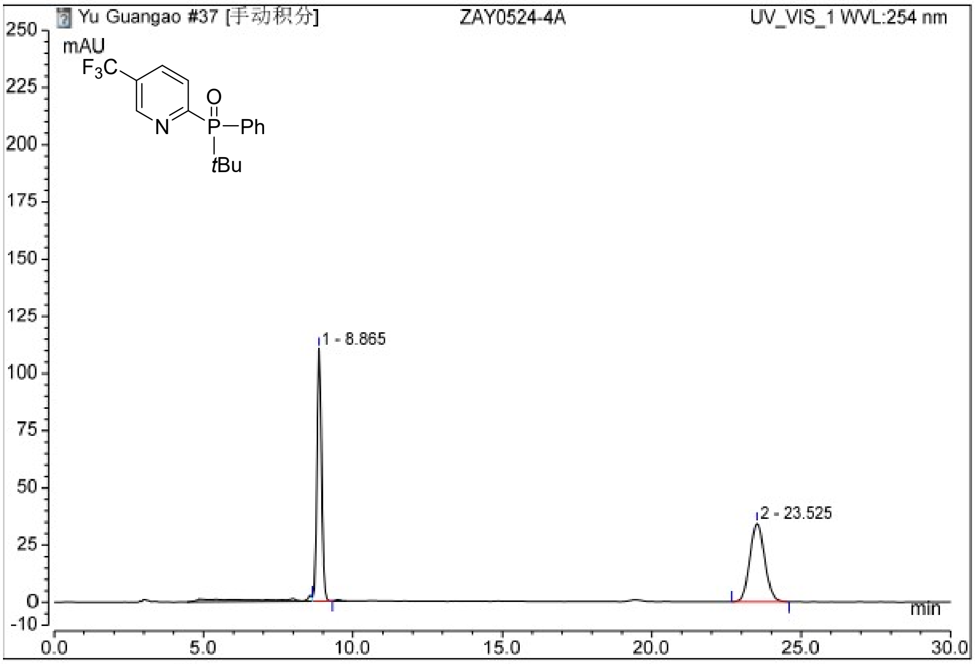

peak.number	peak.name<br>min	peak.area<br>mAU*min	peak.height<br>mAU	peak.rel_area<br>%	peak.rel_height<br>%
1	8.865	19.877	110.436	50.11	76.32
2	23.525	19.790	34.269	49.89	23.68

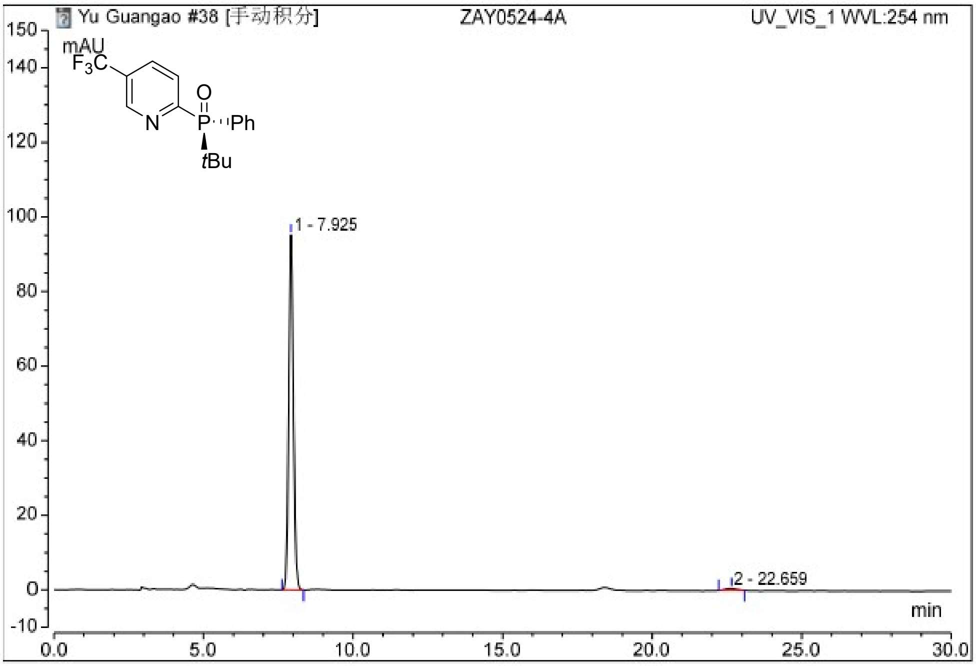

peak.number	peak.name<br>min	peak.area<br>mAU*min	peak.height<br>mAU	peak.rel_area<br>%	peak.rel_height<br>%
1	7.925	17.466	95.217	98.73	99.44
2	22.659	0.225	0.533	1.27	0.56

Chiral HPLC chromatographic analysis of **16**

Condition: Daicel Chiralcel OJ-H, *n*-hexane/*i*-PrOH = 95/5, UV = 254 nm, flow rate: 1.0 mL/min, retention time: t (minor) =11.859 min, t (major) = 13.179 min, ee = 98%.

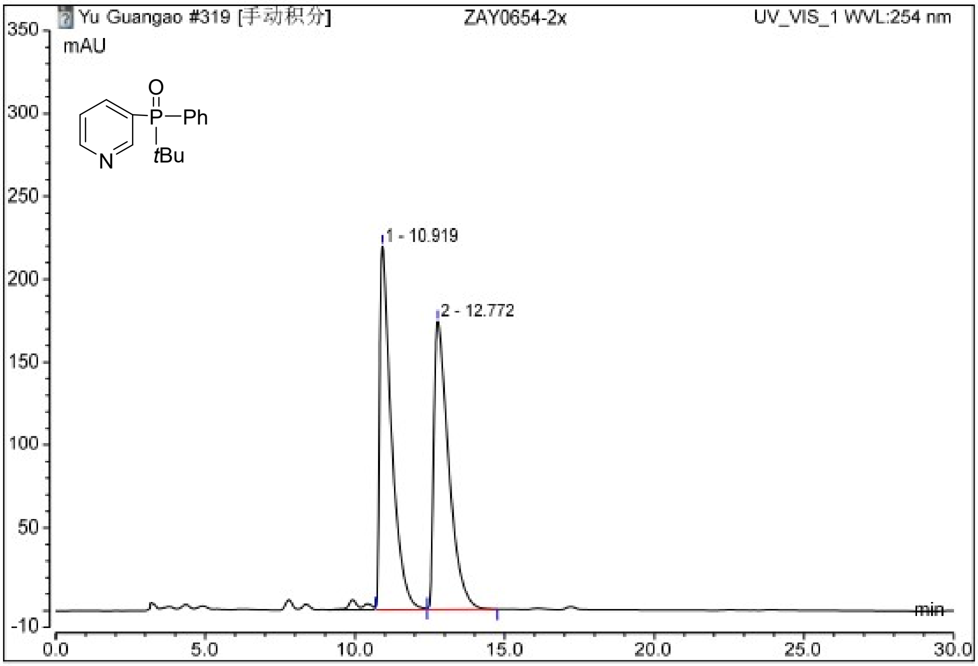

peak.number	peak.name min	peak.area mAU*min	peak.height mAU	peak.rel_area %	peak.rel_height %
1	10.919	101.357	219.363	50.21	55.80
2	12.772	100.529	173.777	49.79	44.20

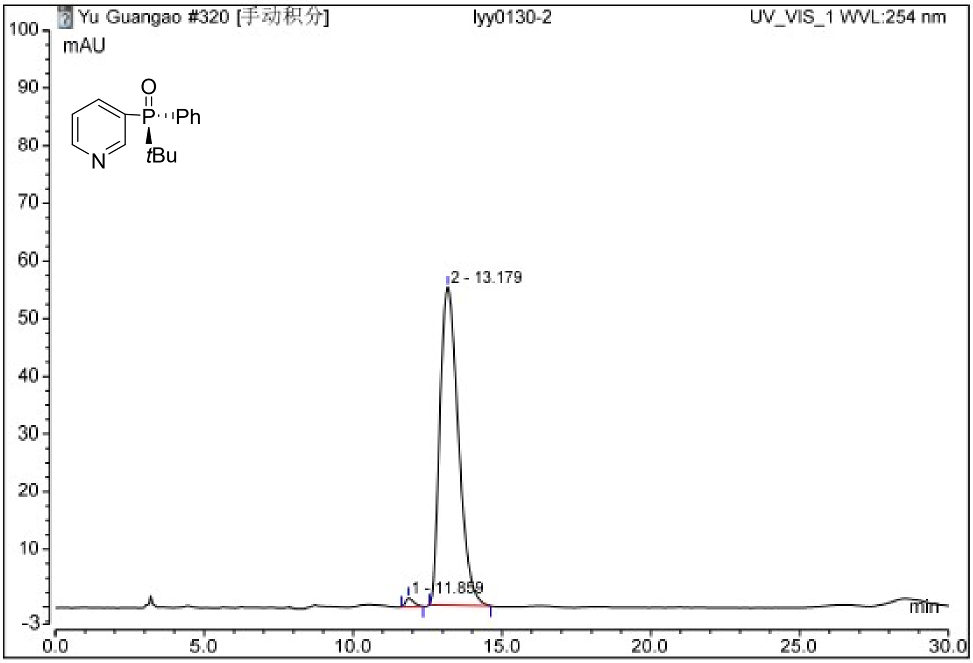

peak.number	peak.name min	peak.area mAU*min	peak.height mAU	peak.rel_area %	peak.rel_height %
1	11.859	0.467	1.493	1.16	2.64
2	13.179	39.766	55.160	98.84	97.36

Chiral HPLC chromatographic analysis of **17**

Condition: Daicel Chiralcel AD-H, *n*-hexane/*i*-PrOH = 85/15, UV = 254 nm, flow rate: 1.0 mL/min, retention time: t (minor) =17.125 min, t (major) = 17.919 min, ee = 99%.

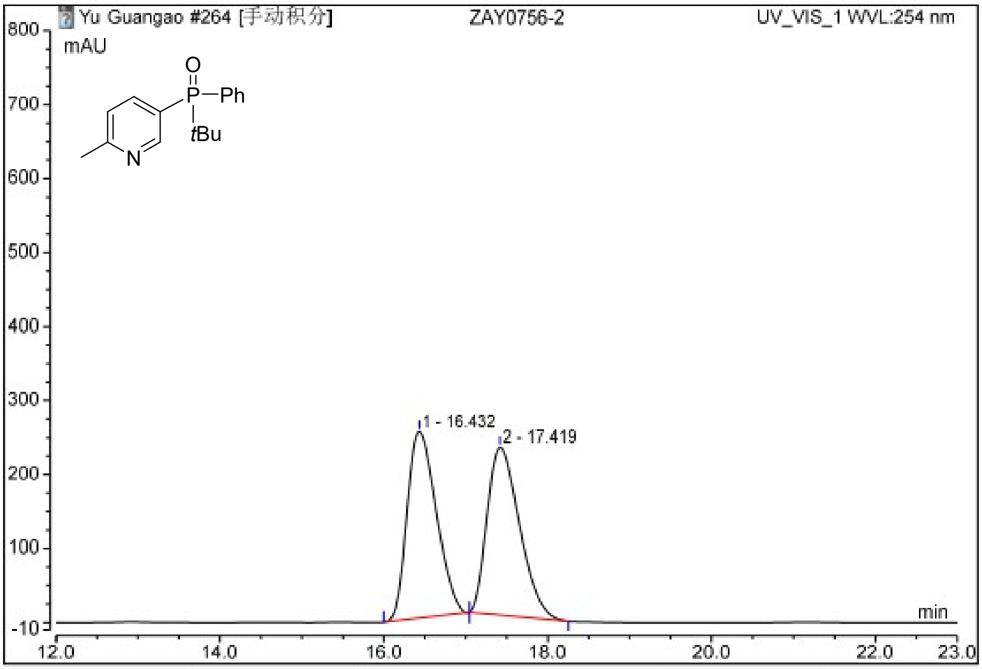

peak.number	peak.name min	peak.area mAU*min	peak.height mAU	peak.rel_area %	peak.rel_height %
1	16.432	103.992	252.309	50.09	52.68
2	17.419	103.628	226.643	49.91	47.32

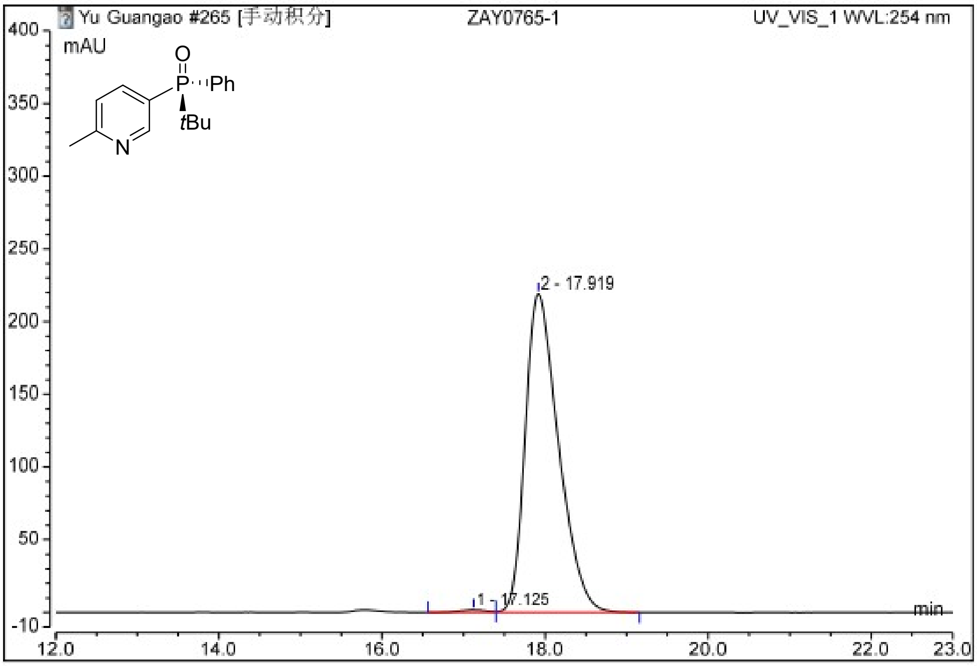

peak.number	peak.name min	peak.area mAU*min	peak.height mAU	peak.rel_area %	peak.rel_height %
1	17.125	0.637	1.674	0.61	0.76
2	17.919	103.846	219.020	99.39	99.24

Chiral HPLC chromatographic analysis of **18**

Condition: Daicel Chiralcel AD-H, *n*-hexane/*i*-PrOH = 85/15, UV = 254 nm, flow rate: 1.0 mL/min, retention time: t (major) =5.639 min, t (minor) = 7.379 min, ee = 99%.

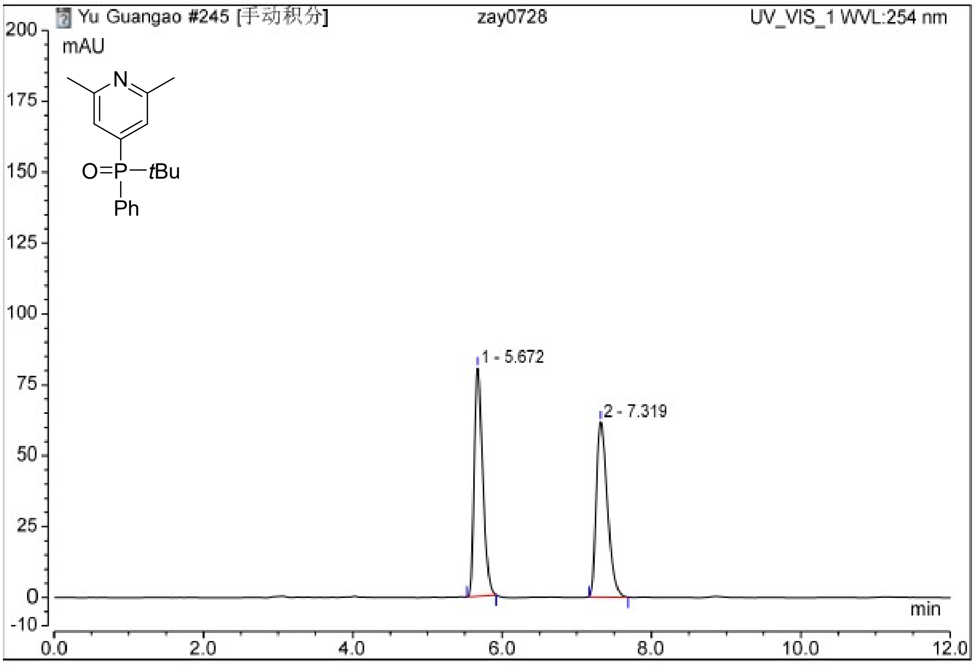

peak.number	peak.name min	peak.area mAU*min	peak.height mAU	peak.rel_area %	peak.rel_height %
1	5.672	10.530	80.469	49.93	56.58
2	7.319	10.561	61.754	50.07	43.42

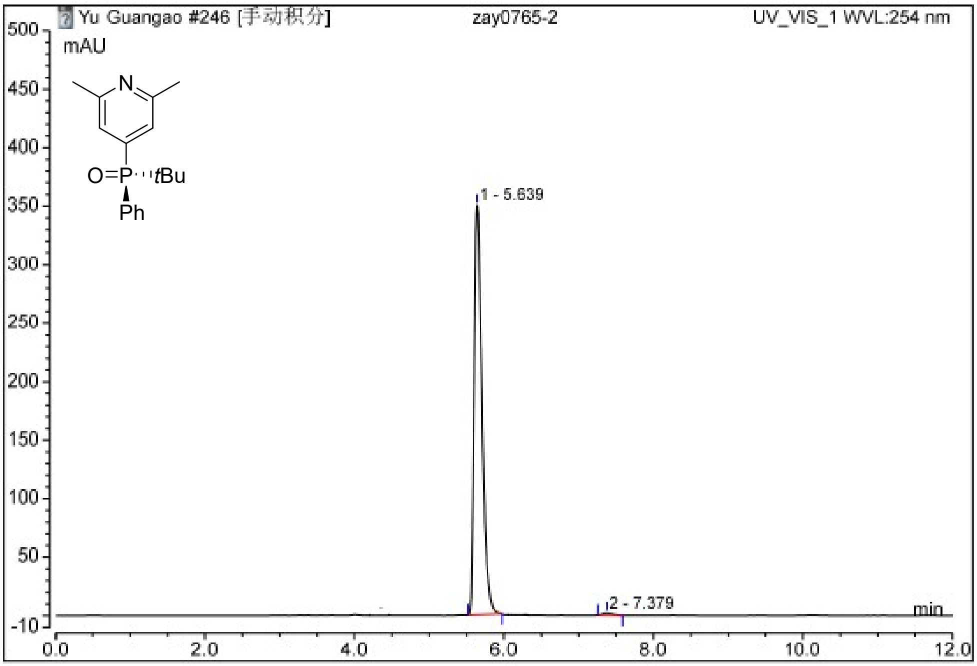

peak.number	peak.name min	peak.area mAU*min	peak.height mAU	peak.rel_area %	peak.rel_height %
1	5.639	42.747	349.980	99.32	99.46
2	7.379	0.291	1.901	0.68	0.54

Chiral HPLC chromatographic analysis of **19**

Condition: Daicel Chiralcel AD-H, *n*-hexane/*i*-PrOH = 80/20, UV = 254 nm, flow rate: 1.0 mL/min, retention time: t (major) =6.879 min, t (minor) = 22.645 min, ee = 98%.

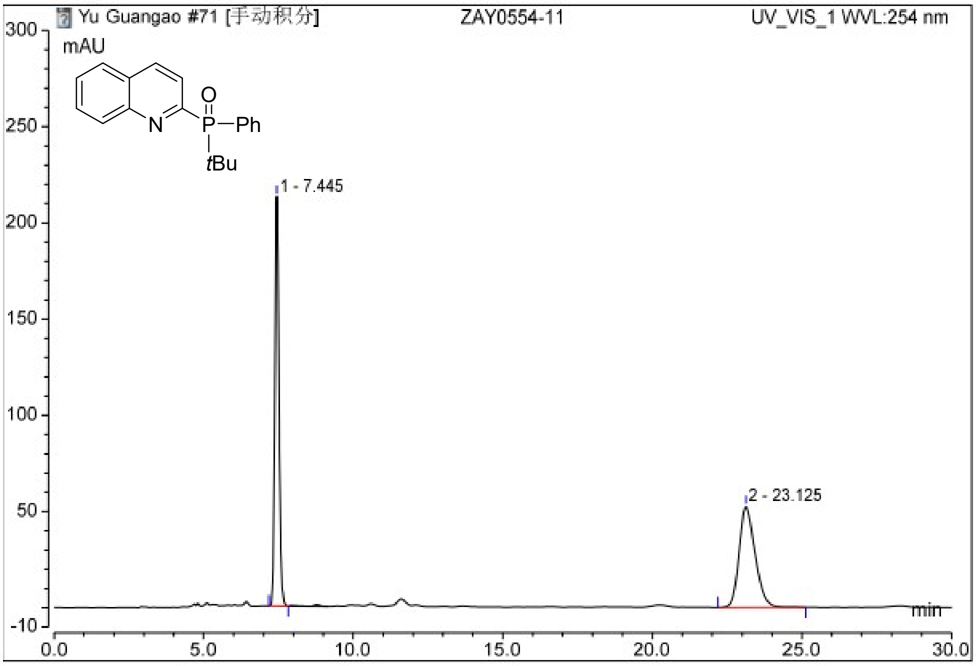

peak.number	peak.name min	peak.area mAU*min	peak.height mAU	peak.rel_area %	peak.rel_height %
1	7.445	33.393	212.856	50.66	80.21
2	23.125	32.528	52.516	49.34	19.79

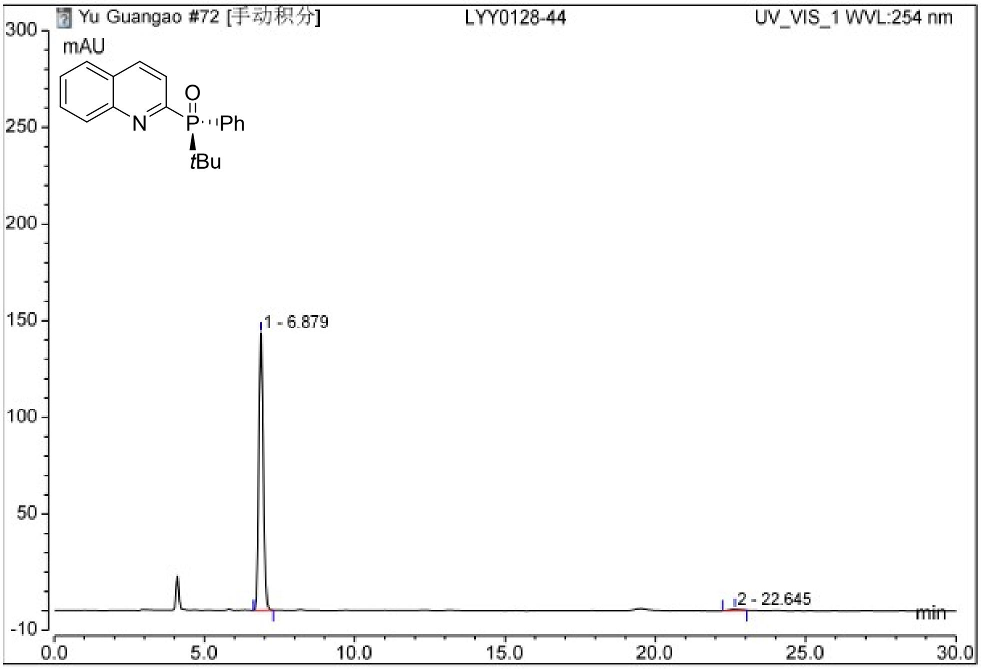

peak.number	peak.name min	peak.area mAU*min	peak.height mAU	peak.rel_area %	peak.rel_height %
1	6.879	24.387	143.461	98.84	99.55
2	22.645	0.287	0.652	1.16	0.45

Chiral HPLC chromatographic analysis of **20**

Condition: Daicel Chiralcel AD-H, *n*-hexane/*i*-PrOH = 85/15, UV = 254 nm, flow rate: 1.0 mL/min, retention time: t (major) =5.285 min, t (minor) = 9.705 min, ee = 99%.

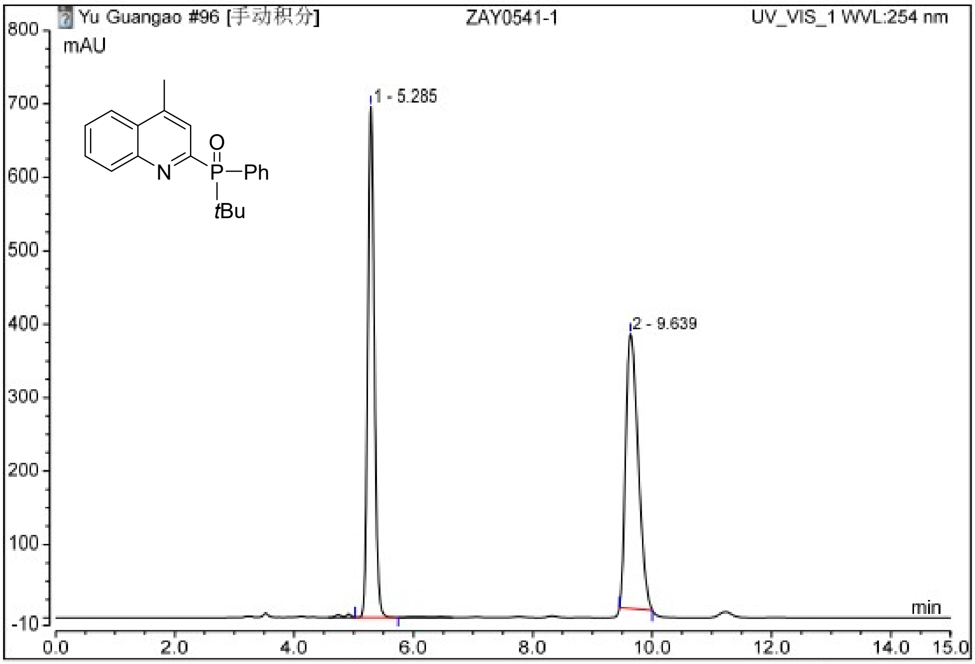

peak.number	peak.name min	peak.area mAU*min	peak.height mAU	peak.rel_area %	peak.rel_height %
1	5.285	88.594	695.872	49.47	65.07
2	9.639	90.505	373.592	50.53	34.93

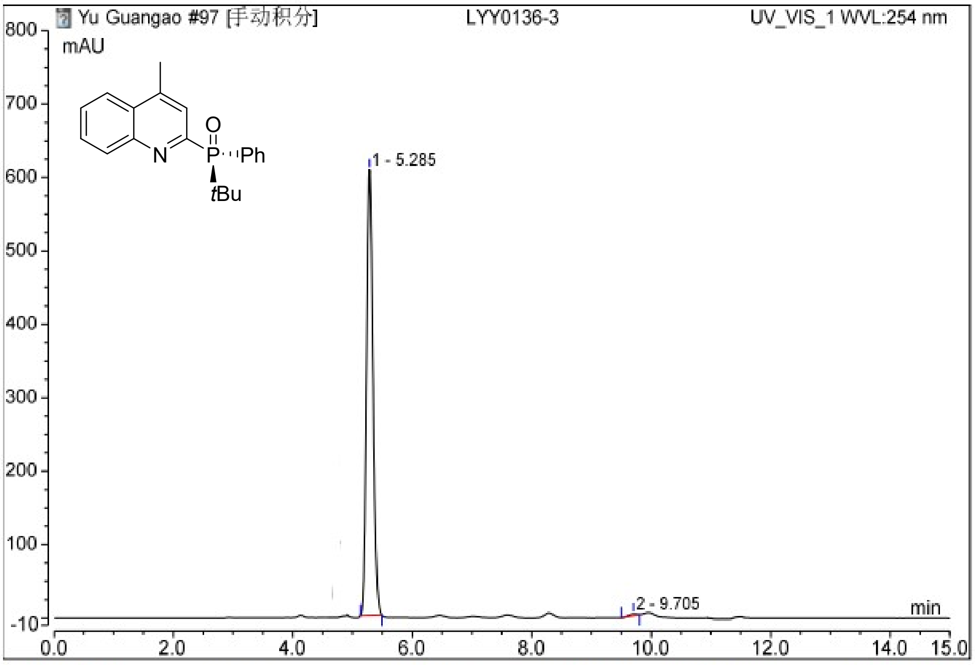

peak.number	peak.name min	peak.area mAU*min	peak.height mAU	peak.rel_area %	peak.rel_height %
1	5.285	74.828	606.846	99.59	99.66
2	9.705	0.310	2.065	0.41	0.34

Chiral HPLC chromatographic analysis of **21**

Condition: Daicel Chiralcel AD-H, *n*-hexane/*i*-PrOH = 85/15, UV = 254 nm, flow rate: 1.0 mL/min, retention time: t (minor) =8.792 min, t (major) = 10.739 min, ee = 98%.

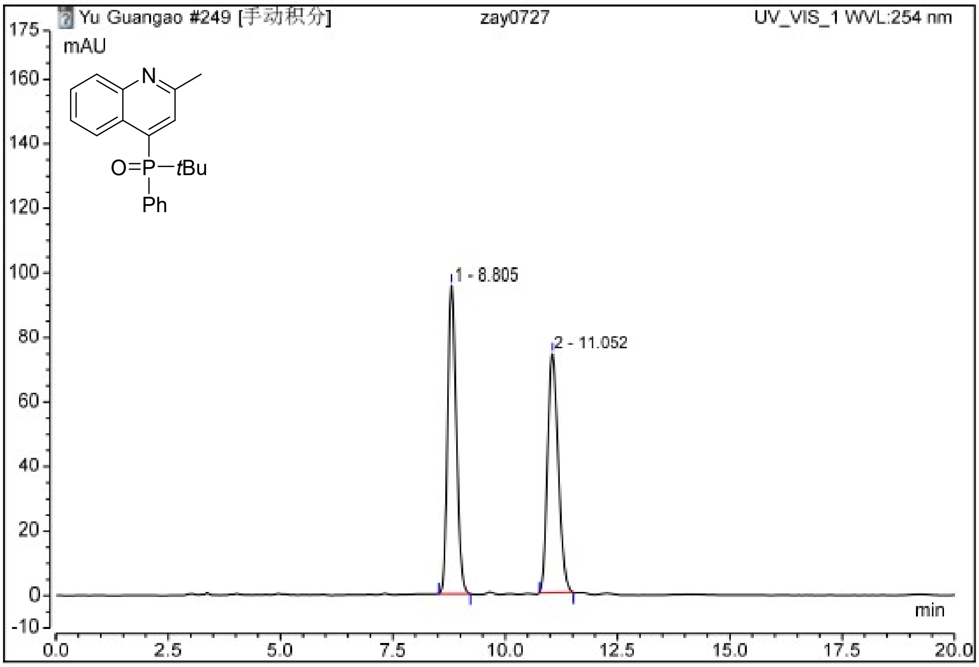

peak.number	peak.name min	peak.area mAU*min	peak.height mAU	peak.rel_area %	peak.rel_height %
1	8.805	21.133	95.664	50.66	56.34
2	11.052	20.584	74.137	49.34	43.66

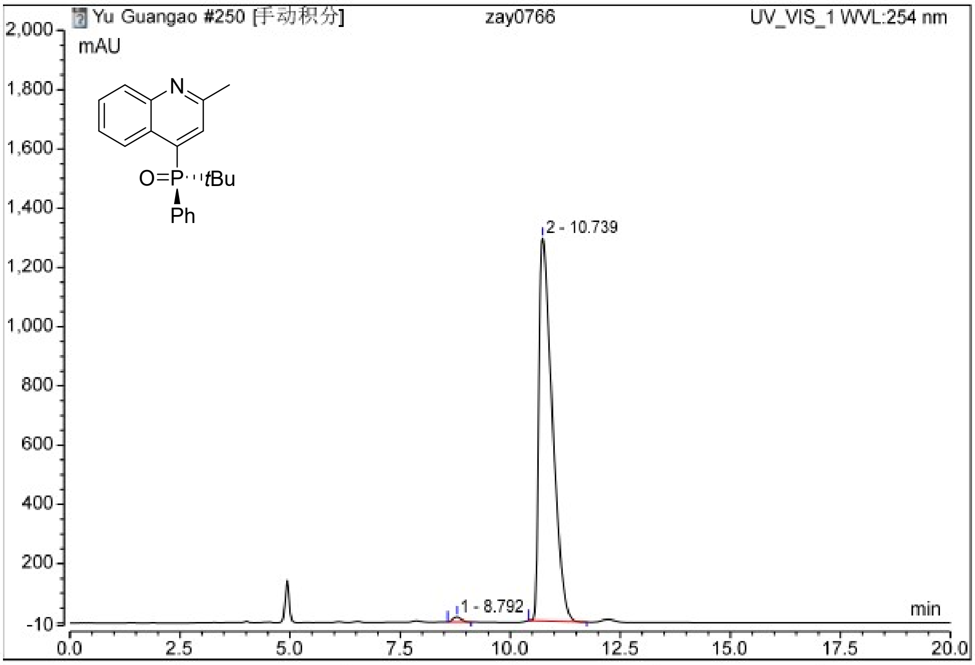

peak.number	peak.name min	peak.area mAU*min	peak.height mAU	peak.rel_area %	peak.rel_height %
1	8.792	3.507	16.599	0.76	1.27
2	10.739	459.330	1291.855	99.24	98.73

Chiral HPLC chromatographic analysis of **22**

Condition: Daicel Chiralcel AD-H, *n*-hexane/*i*-PrOH = 95/05, UV = 254 nm, flow rate: 1.0 mL/min, retention time: t (minor) =26.065 min, t (major) = 28.952 min, ee = 97%.

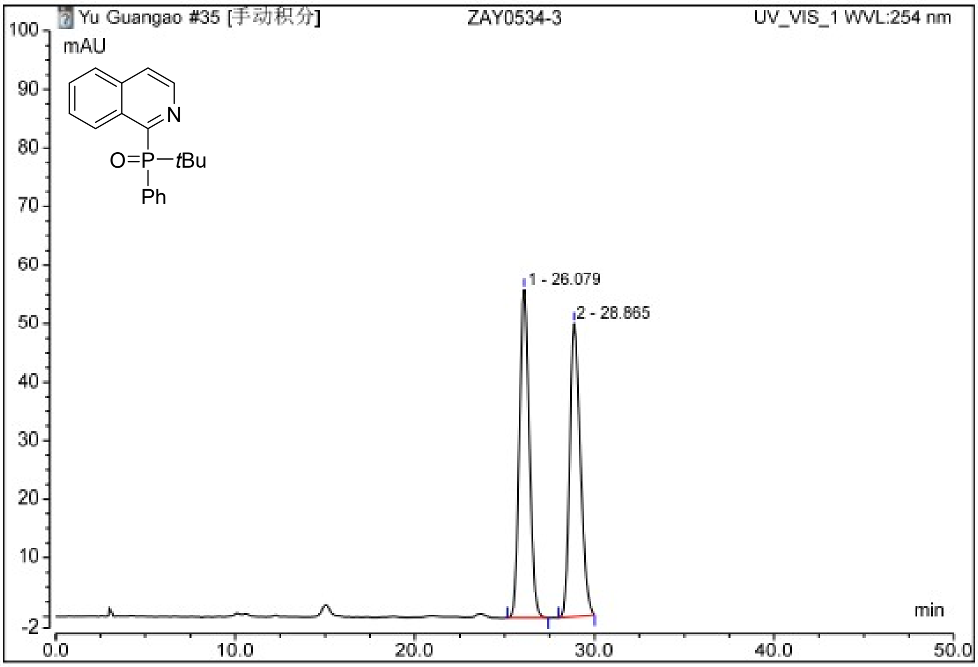

peak.number	peak.name min	peak.area mAU*min	peak.height mAU	peak.rel_area %	peak.rel_height %
1	26.079	35.243	56.133	50.35	52.85
2	28.865	34.751	50.075	49.65	47.15

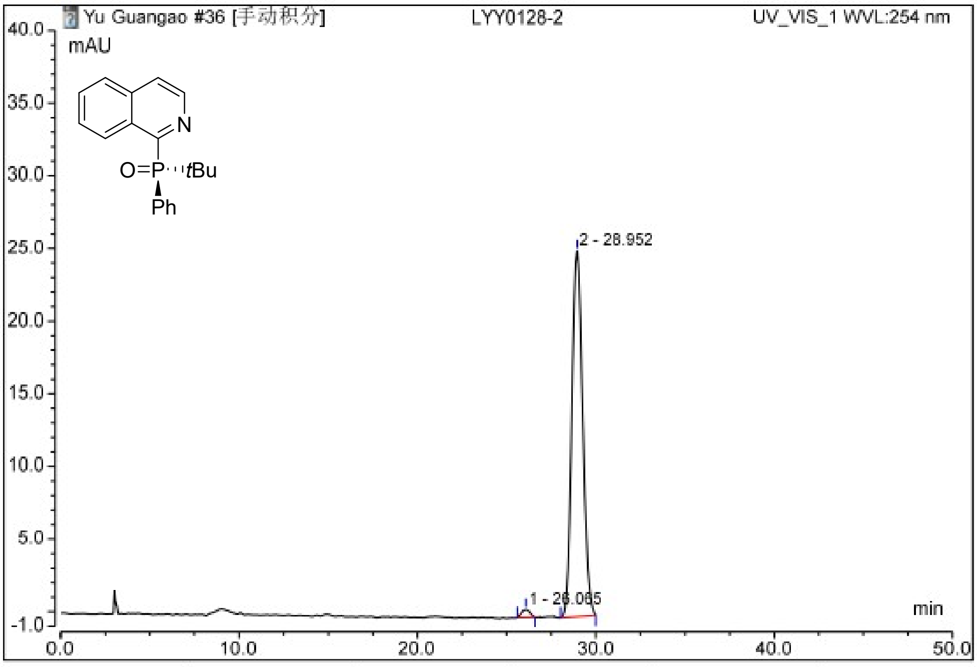

peak.number	peak.name min	peak.area mAU*min	peak.height mAU	peak.rel_area %	peak.rel_height %
1	26.065	0.296	0.543	1.64	2.11
2	28.952	17.760	25.179	98.36	97.89

Chiral HPLC chromatographic analysis of **23**

Condition: Daicel Chiralcel AD-H, *n*-hexane/*i*-PrOH = 85/15, UV = 254 nm, flow rate: 1.0 mL/min, retention time: t (minor) =9.185 min, t (major) = 16.112 min, ee = 97%.

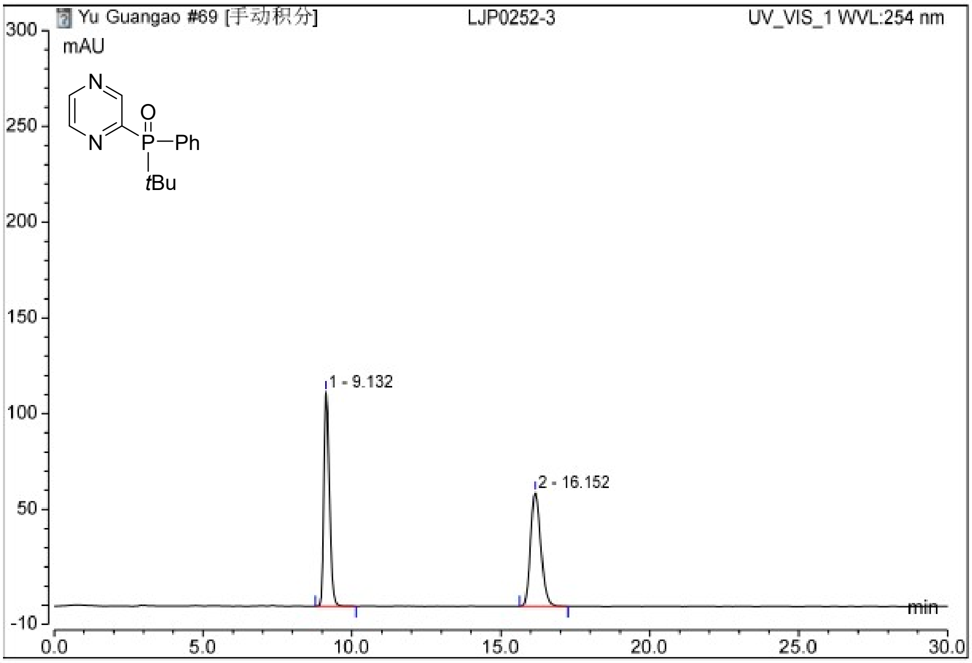

peak.number	peak.name<br>min	peak.area<br>mAU*min	peak.height<br>mAU	peak.rel_area<br>%	peak.rel_height<br>%
1	9.132	23.966	111.931	49.98	65.28
2	16.152	23.989	59.519	50.02	34.72

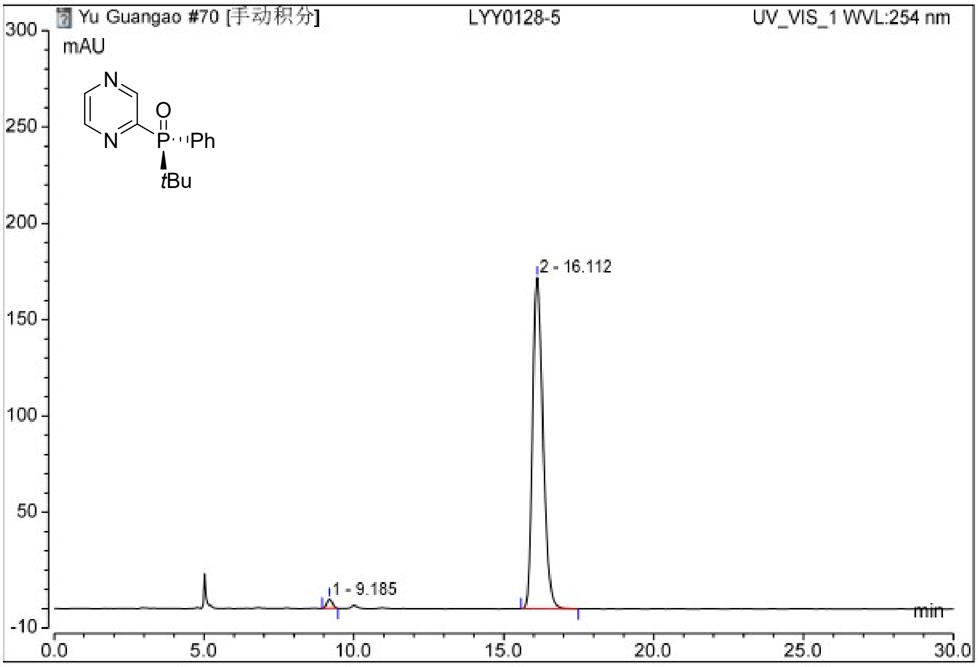

peak.number	peak.name<br>min	peak.area<br>mAU*min	peak.height<br>mAU	peak.rel_area<br>%	peak.rel_height<br>%
1	9.185	1.023	4.886	1.46	2.76
2	16.112	69.209	171.955	98.54	97.24

Chiral HPLC chromatographic analysis of **24**

Condition: Daicel Chiralcel AD-H, *n*-hexane/*i*-PrOH = 90/10, UV = 254 nm, flow rate: 1.0 mL/min, retention time: t (major) =7.519 min, t (minor) = 8.092 min, ee = 97%.

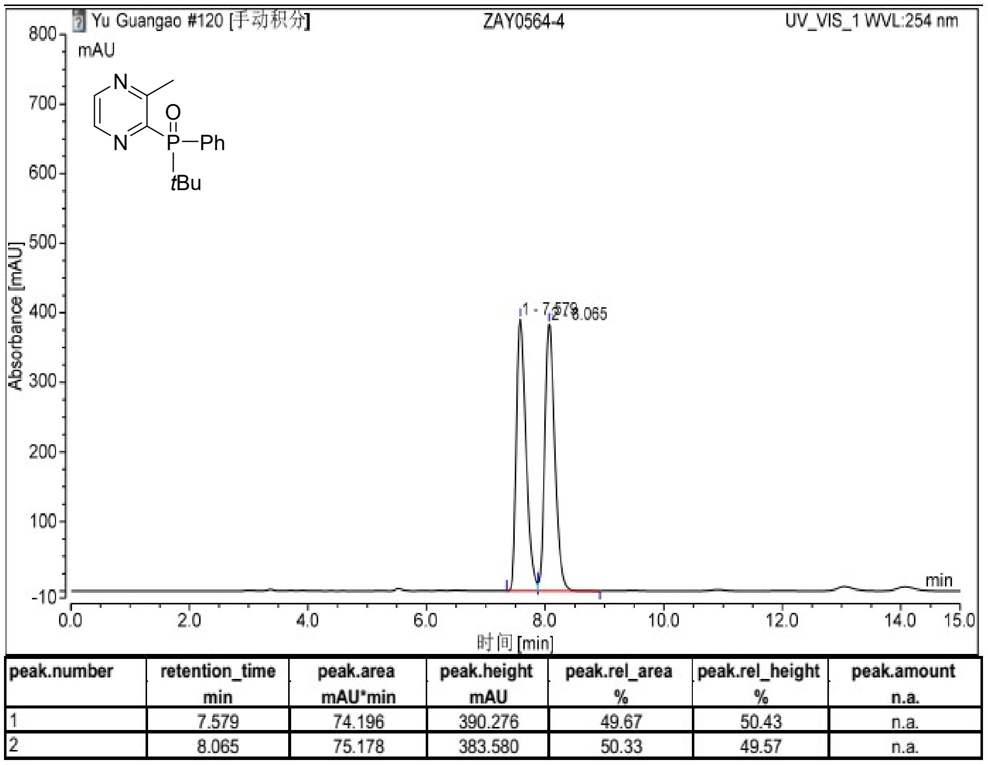

peak.number	retention_time	peak.area	peak.height	peak.rel_area	peak.rel_height	peak.amount
	min	mAU*min	mAU	%	%	n.a.
1	7.579	74.196	390.276	49.67	50.43	n.a.
2	8.065	75.178	383.580	50.33	49.57	n.a.

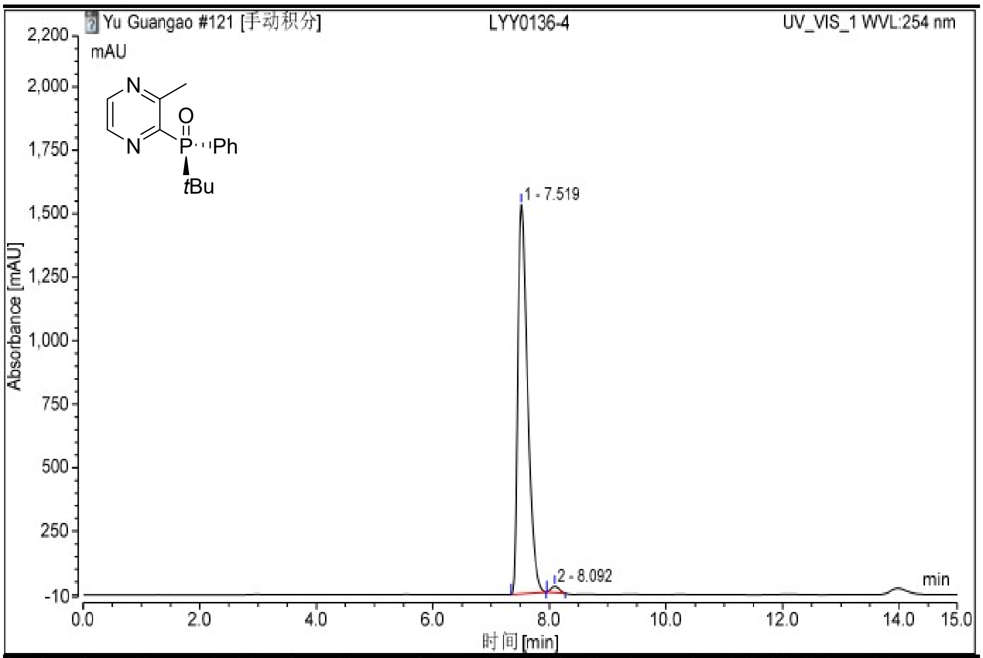

peak.number	retention_time	peak.area	peak.height	peak.rel_area	peak.rel_height	peak.amount
	min	mAU*min	mAU	%	%	n.a.
1	7.519	297.041	1531.724	98.69	98.47	n.a.
2	8.092	3.929	23.780	1.31	1.53	n.a.

Chiral HPLC chromatographic analysis of **25**

Condition: Daicel Chiralcel AD-H, *n*-hexane/*i*-PrOH = 85/15, UV = 254 nm, flow rate: 1.0 mL/min, retention time: t (major) =11.279 min, t (minor) = 13.519 min, ee = 99%.

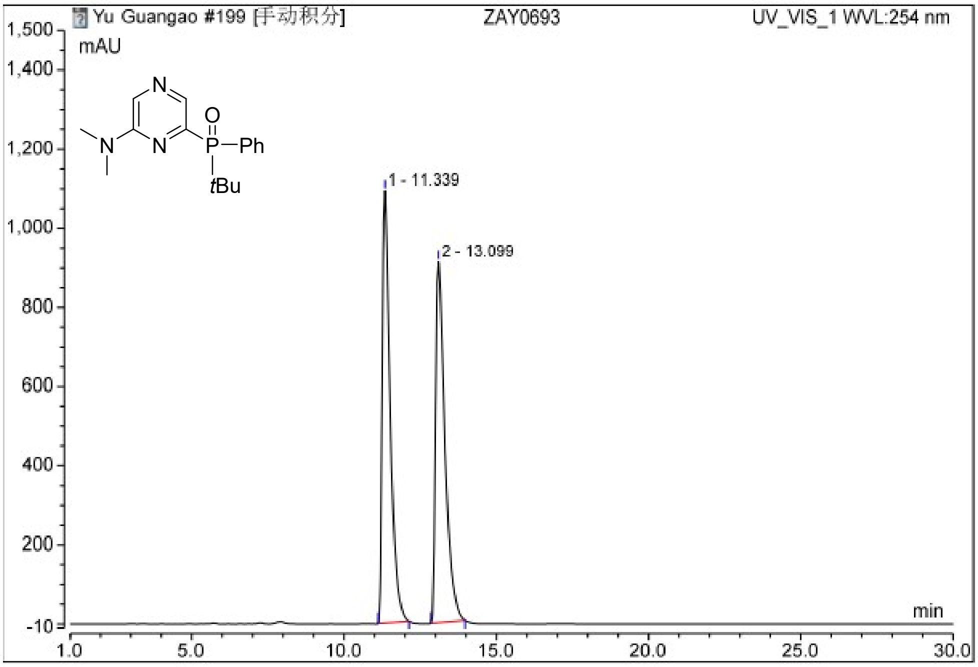

peak.number	peak.name<br>min	peak.area<br>mAU*min	peak.height<br>mAU	peak.rel_area<br>%	peak.rel_height<br>%
1	11.339	330.347	1091.598	50.14	54.48
2	13.099	328.556	912.038	49.86	45.52

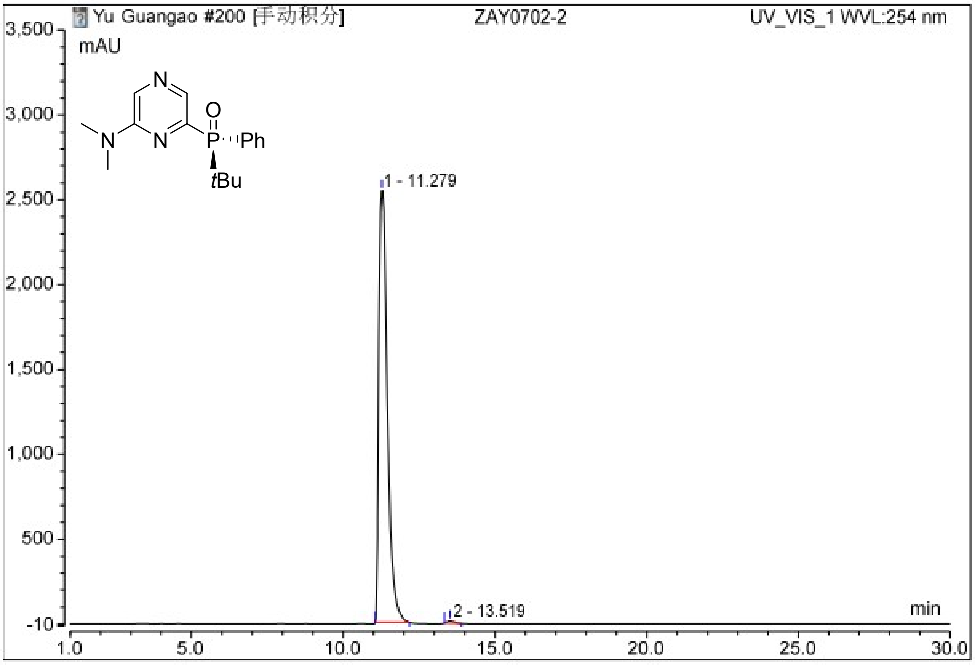

peak.number	peak.name<br>min	peak.area<br>mAU*min	peak.height<br>mAU	peak.rel_area<br>%	peak.rel_height<br>%
1	11.279	829.956	2544.212	99.49	99.41
2	13.519	4.254	15.015	0.51	0.59

Chiral HPLC chromatographic analysis of **26**

Condition: Daicel Chiralcel AD-H, *n*-hexane/*i*-PrOH = 98/02, UV = 254 nm, flow rate: 1.0 mL/min, retention time: t (minor) =38.425 min, t (major) = 42.452 min, ee = 97%.

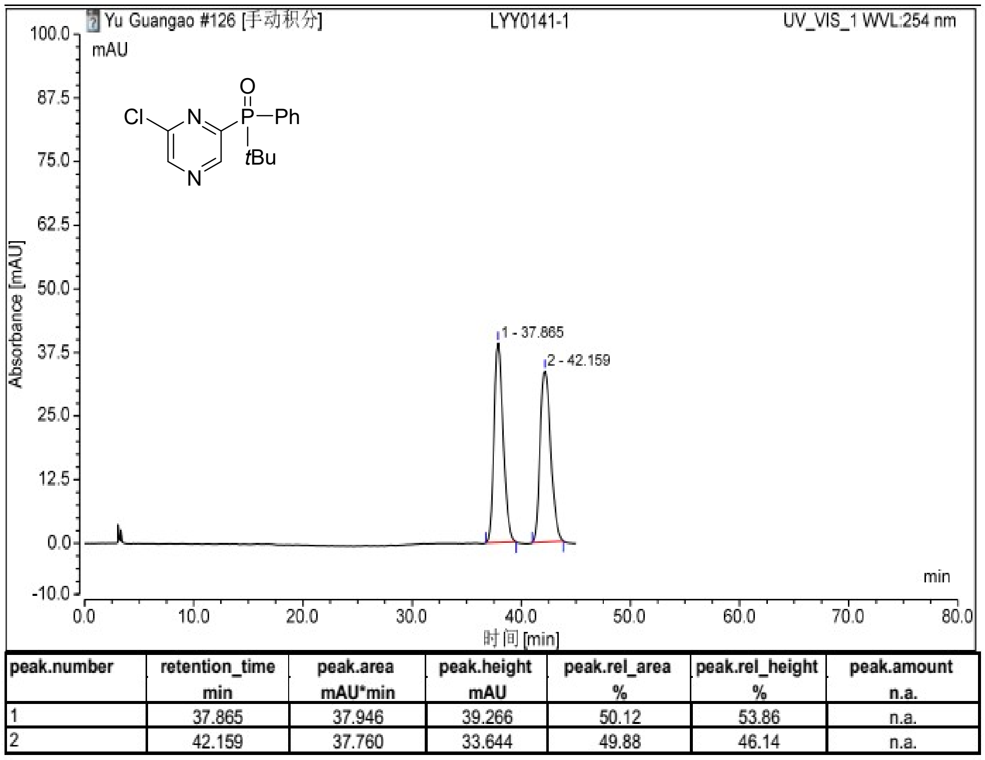

peak.number	retention_time min	peak.area mAU*min	peak.height mAU	peak.rel_area %	peak.rel_height %	peak.amount n.a.
1	37.865	37.946	39.266	50.12	53.86	n.a.
2	42.159	37.760	33.644	49.88	46.14	n.a.

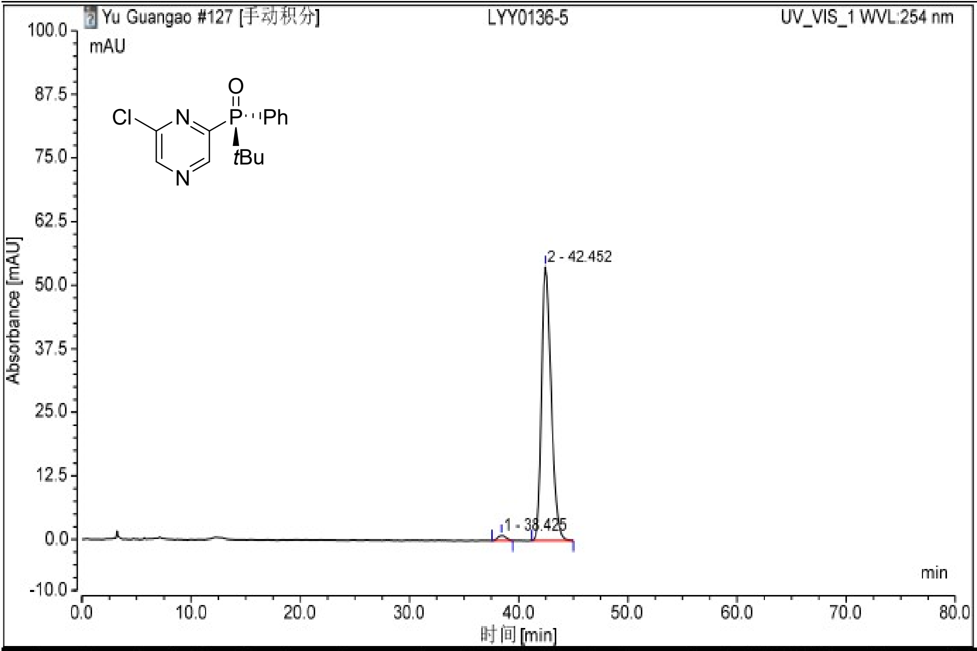

peak.number	retention_time min	peak.area mAU*min	peak.height mAU	peak.rel_area %	peak.rel_height %	peak.amount n.a.
1	38.425	0.898	1.013	1.60	1.85	n.a.
2	42.452	55.104	53.883	98.40	98.15	n.a.

Chiral HPLC chromatographic analysis of **27**

Condition: Daicel Chiralcel AD-H, *n*-hexane/*i*-PrOH = 85/15, UV = 254 nm, flow rate: 1.0 mL/min, retention time: t (major) =13.772 min, t (minor) = 21.719 min, ee = 98%.

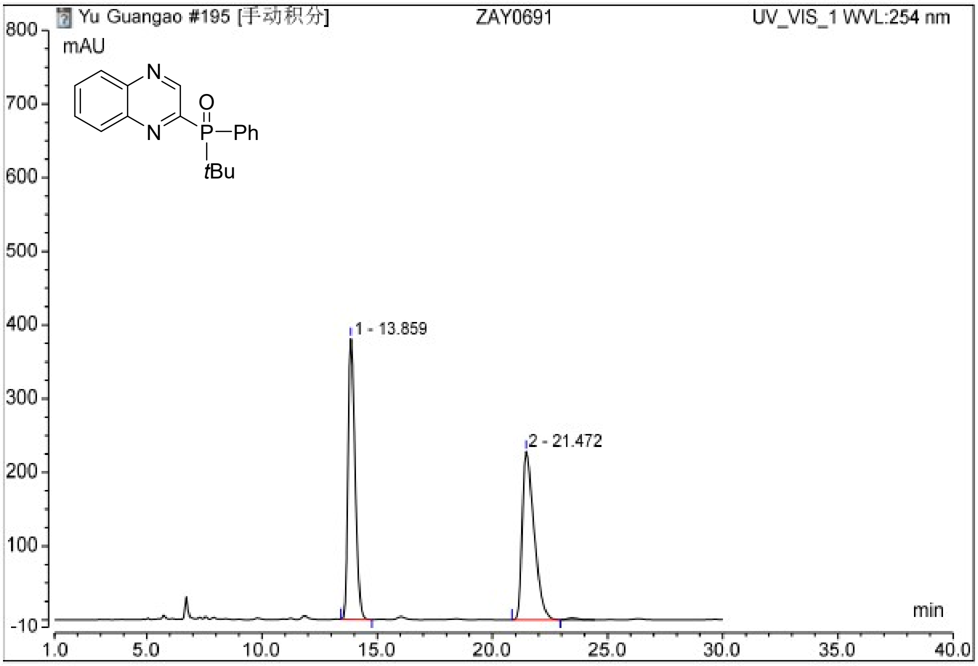

peak.number	peak.name min	peak.area mAU*min	peak.height mAU	peak.rel_area %	peak.rel_height %
1	13.859	133.799	380.904	49.60	62.49
2	21.472	135.963	228.618	50.40	37.51

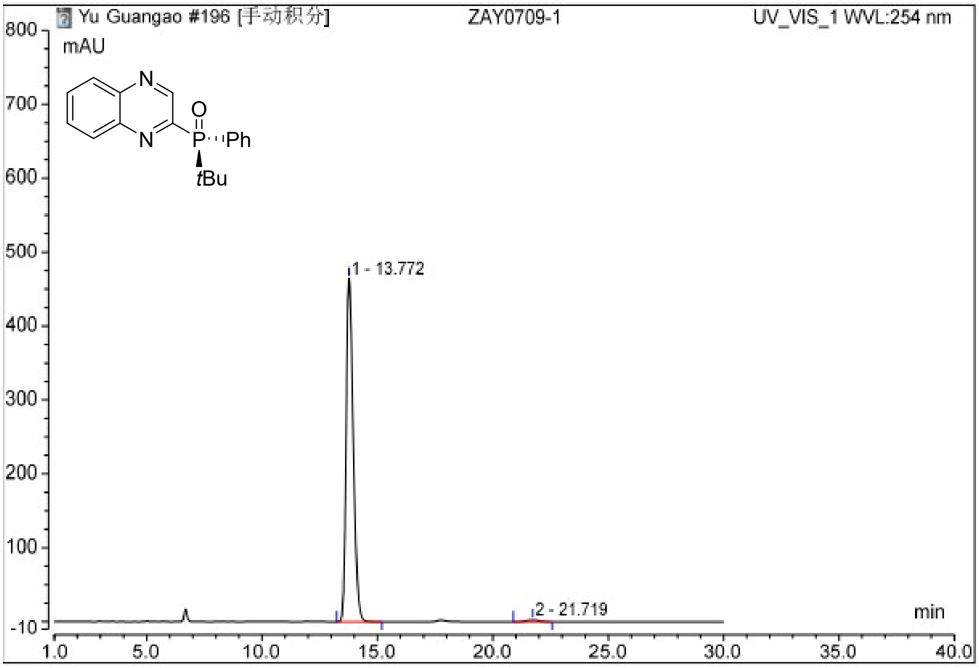

peak.number	peak.name min	peak.area mAU*min	peak.height mAU	peak.rel_area %	peak.rel_height %
1	13.772	164.611	464.518	99.05	99.46
2	21.719	1.585	2.518	0.95	0.54

Chiral HPLC chromatographic analysis of **28**

Condition: Daicel Chiralcel AD-H, *n*-hexane/*i*-PrOH = 85/15, UV = 254 nm, flow rate: 1.0 mL/min, retention time: t (minor) =11.839 min, t (major) = 12.679 min, ee = 98%.

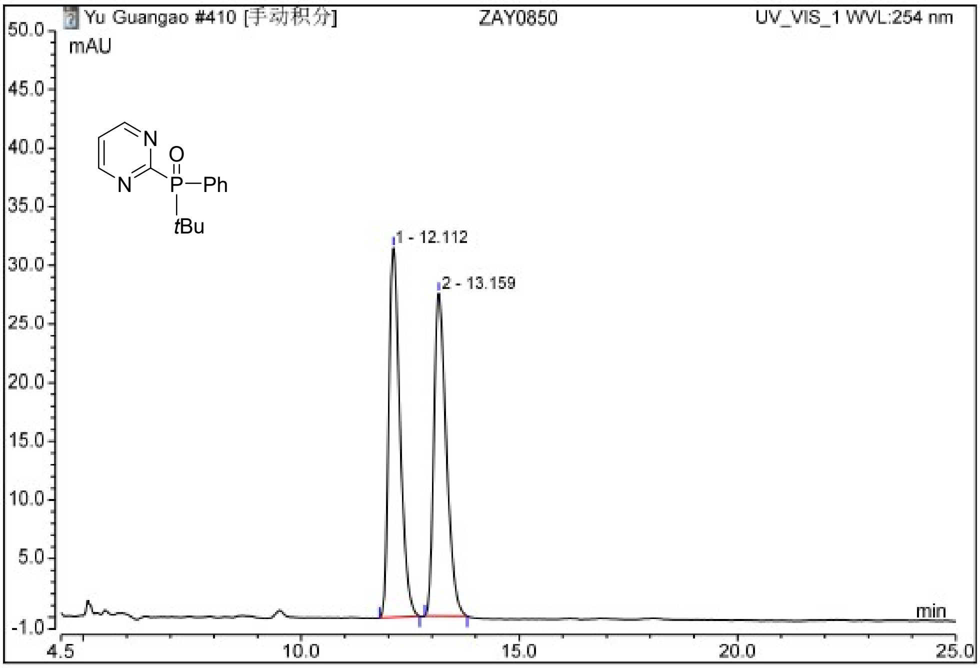

peak.number	peak.name min	peak.area mAU*min	peak.height mAU	peak.rel_area %	peak.rel_height %
1	12.112	9.449	31.518	51.04	53.40
2	13.159	9.064	27.506	48.96	46.60

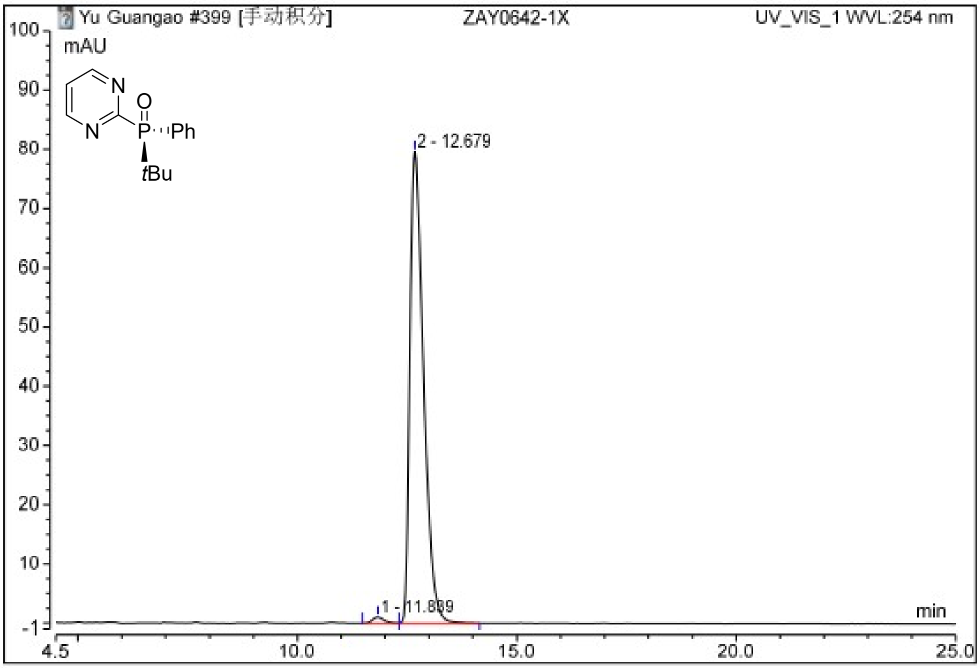

peak.number	peak.name min	peak.area mAU*min	peak.height mAU	peak.rel_area %	peak.rel_height %
1	11.839	0.330	1.067	1.14	1.32
2	12.679	28.503	79.648	98.86	98.68

Chiral HPLC chromatographic analysis of **29**

Condition: Daicel Chiralcel AD-H, *n*-hexane/*i*-PrOH = 85/15, UV = 254 nm, flow rate: 1.0 mL/min, retention time: t (major) =12.525 min, t (minor) = 22.972 min, ee = 99%.

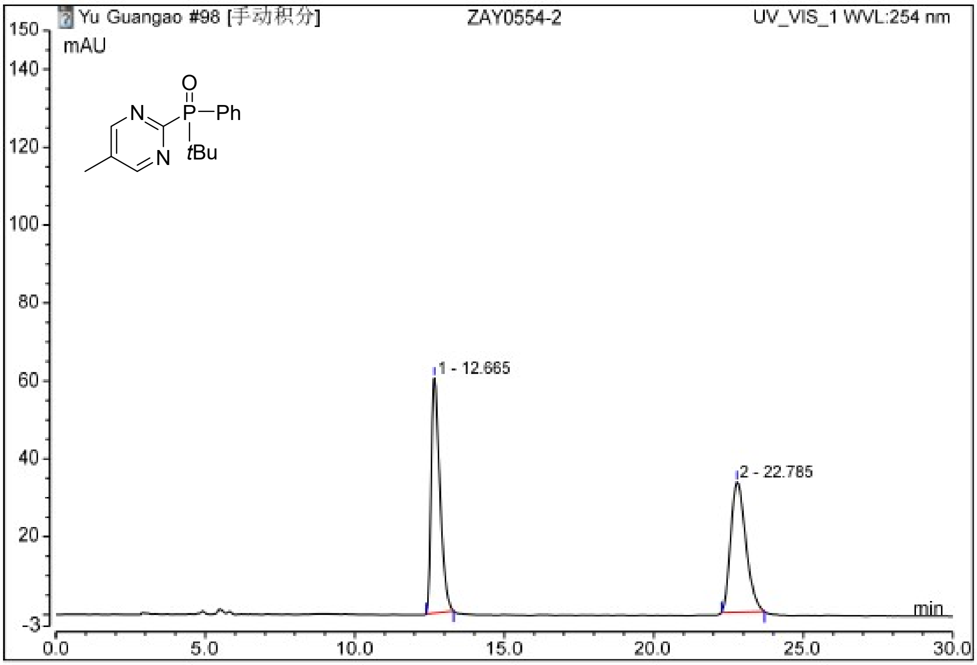

peak.number	peak.name min	peak.area mAU*min	peak.height mAU	peak.rel_area %	peak.rel_height %
1	12.665	20.426	60.355	50.75	64.35
2	22.785	19.821	33.432	49.25	35.65

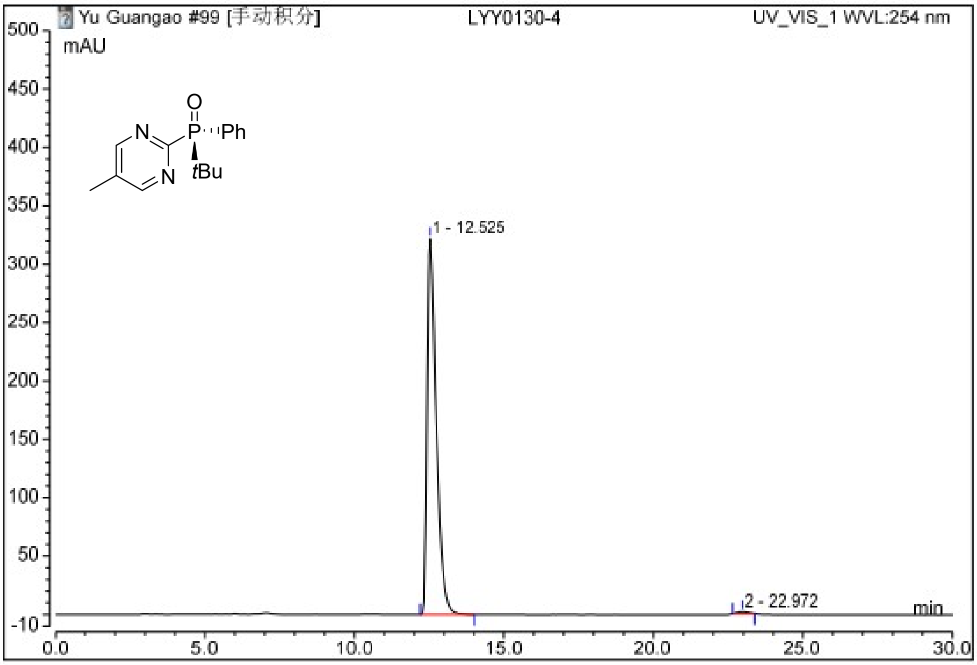

peak.number	peak.name min	peak.area mAU*min	peak.height mAU	peak.rel_area %	peak.rel_height %
1	12.525	115.082	322.388	99.32	99.43
2	22.972	0.784	1.844	0.68	0.57

Chiral HPLC chromatographic analysis of **30**

Condition: Daicel Chiralcel AS-H, *n*-hexane/*i*-PrOH = 90/10, UV = 254 nm, flow rate: 1.0 mL/min, retention time: t (major) =7.879 min, t (minor) = 11.565 min, ee = 99%.

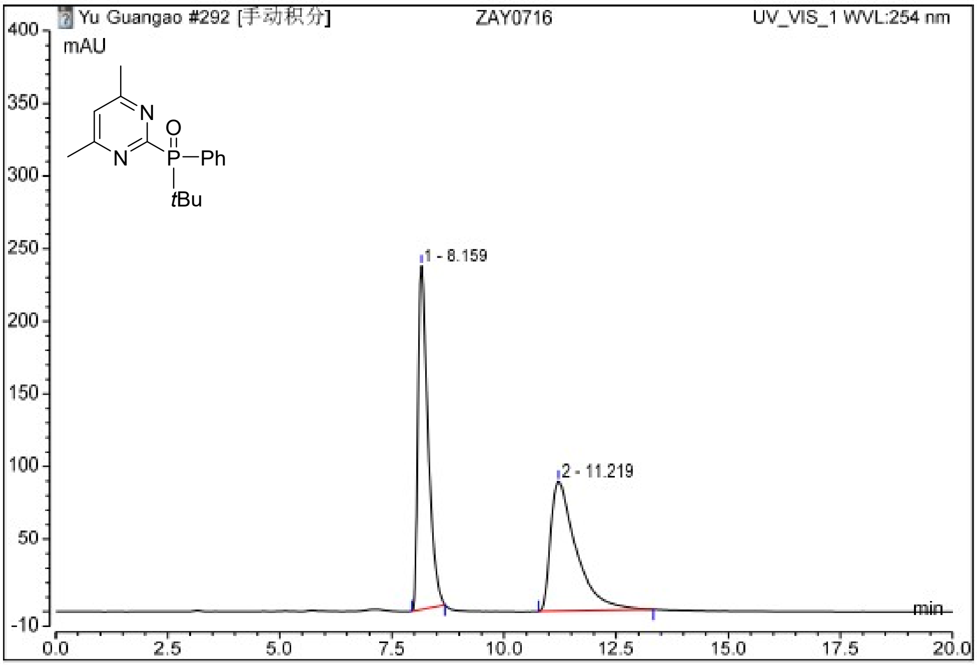

peak.number	peak.name min	peak.area mAU*min	peak.height mAU	peak.rel_area %	peak.rel_height %
1	8.159	61.424	236.562	50.98	72.61
2	11.219	59.062	89.243	49.02	27.39

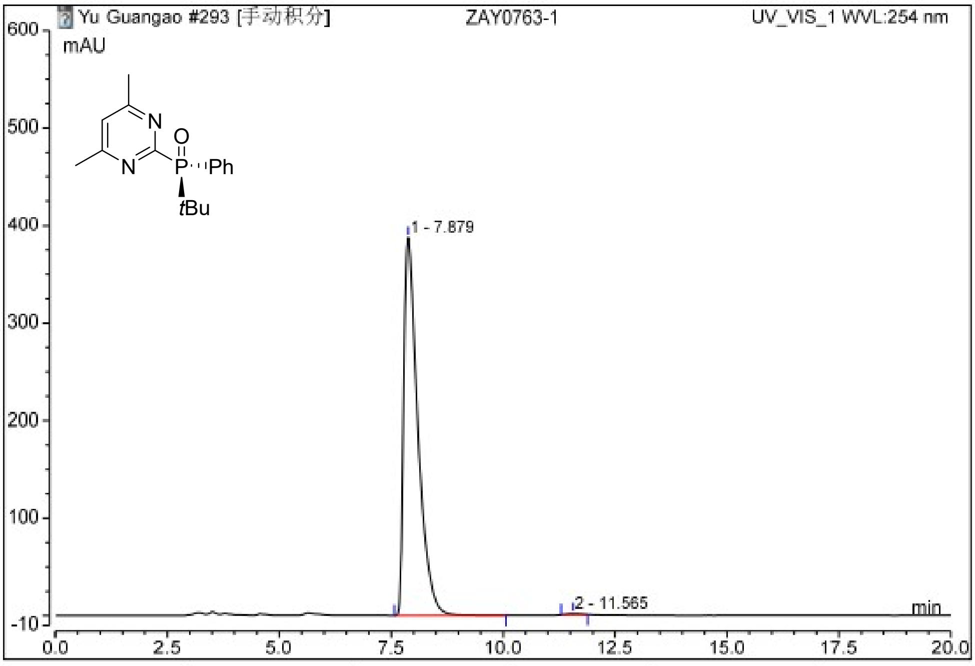

peak.number	peak.name min	peak.area mAU*min	peak.height mAU	peak.rel_area %	peak.rel_height %
1	7.879	140.230	387.629	99.74	99.74
2	11.565	0.362	1.028	0.26	0.26

Chiral HPLC chromatographic analysis of **31**

Condition: Daicel Chiralcel AD-H, *n*-hexane/*i*-PrOH = 80/20, UV = 254 nm, flow rate: 1.0 mL/min, retention time: t (major) =6.365 min, t (minor) = 6.739 min, ee = 99%.

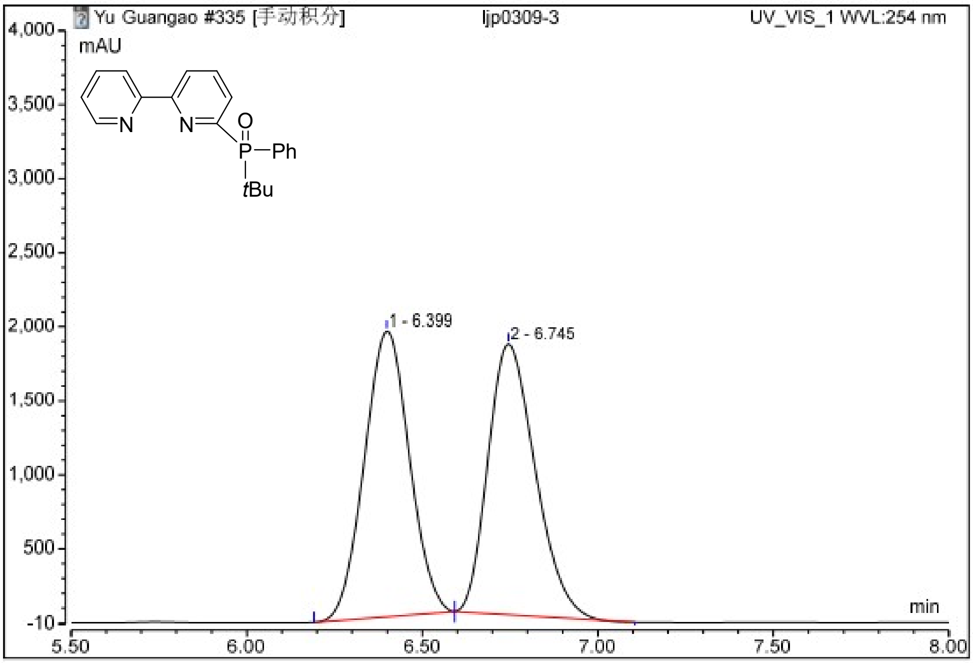

peak.number	peak.name min	peak.area mAU*min	peak.height mAU	peak.rel_area %	peak.rel_height %
1	6.399	285.603	1928.633	49.99	51.34
2	6.745	285.715	1828.092	50.01	48.66

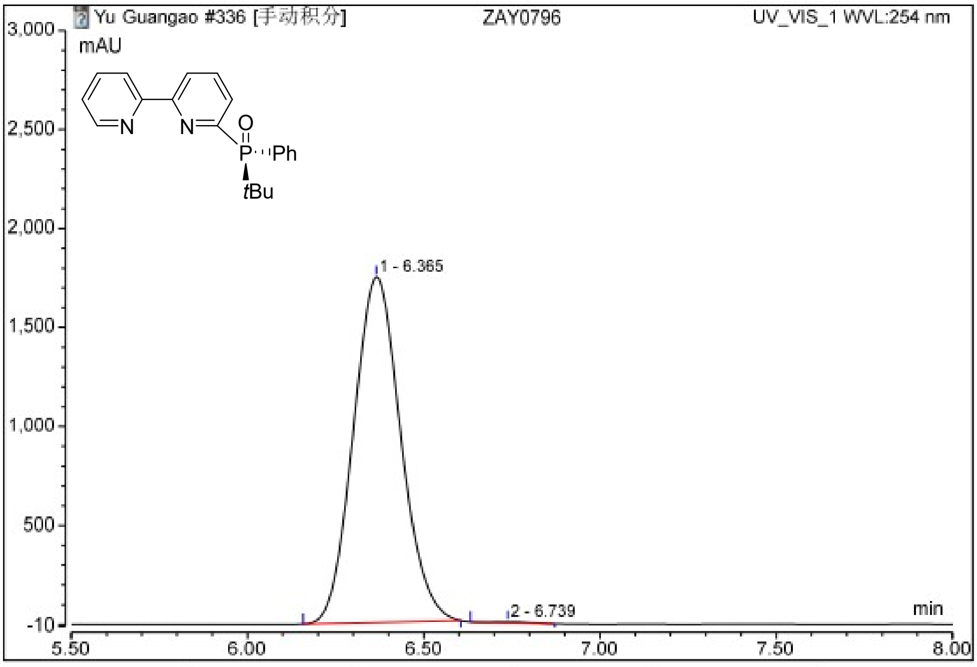

peak.number	peak.name min	peak.area mAU*min	peak.height mAU	peak.rel_area %	peak.rel_height %
1	6.365	271.994	1746.152	99.80	99.73
2	6.739	0.545	4.809	0.20	0.27

Chiral HPLC chromatographic analysis of **32**

Condition: Daicel Chiralcel AD-H, *n*-hexane/*i*-PrOH = 85/15, UV = 254 nm, flow rate: 1.0 mL/min, retention time: t (minor) =9.899 min, t (major) = 11.185 min, ee = 99%.

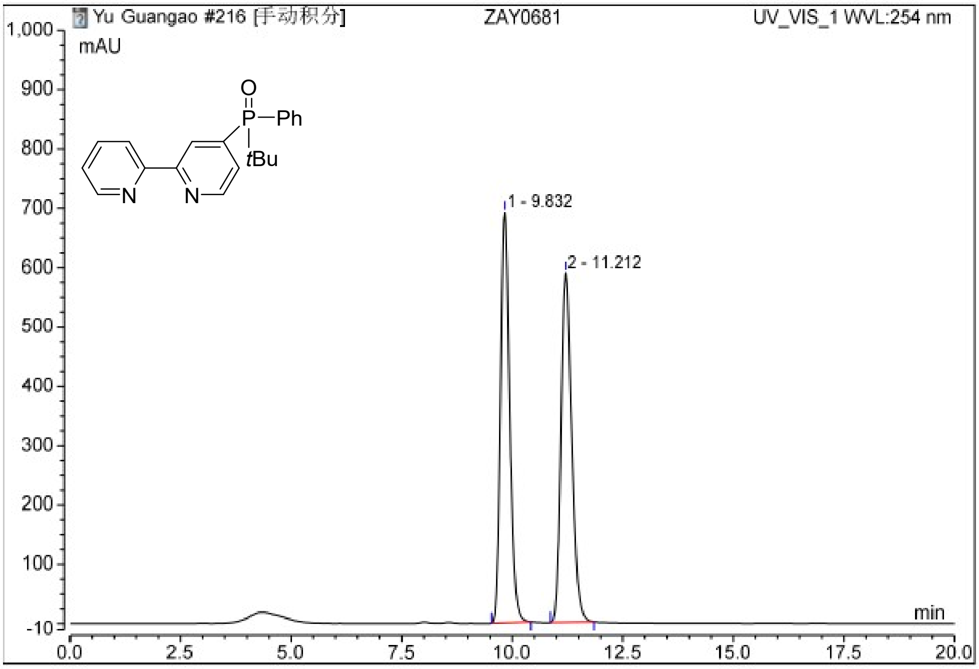

peak.number	peak.name min	peak.area mAU*min	peak.height mAU	peak.rel_area %	peak.rel_height %
1	9.832	164.429	691.855	49.79	54.00
2	11.212	165.794	589.275	50.21	46.00

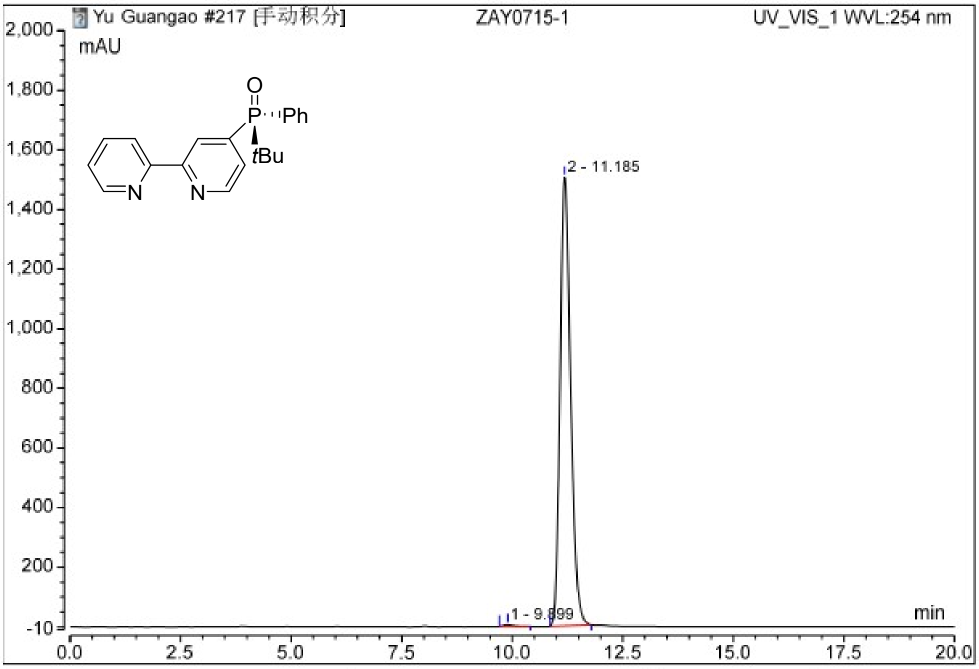

peak.number	peak.name min	peak.area mAU*min	peak.height mAU	peak.rel_area %	peak.rel_height %
1	9.899	1.438	5.715	0.35	0.38
2	11.185	414.966	1504.790	99.65	99.62

Chiral HPLC chromatographic analysis of **33**

Condition: Daicel Chiralcel AD-H, *n*-hexane/*i*-PrOH = 85/15, UV = 254 nm, flow rate: 1.0 mL/min, retention time: t (minor) =13.272 min, t (major) = 14.252 min, ee = 98%.

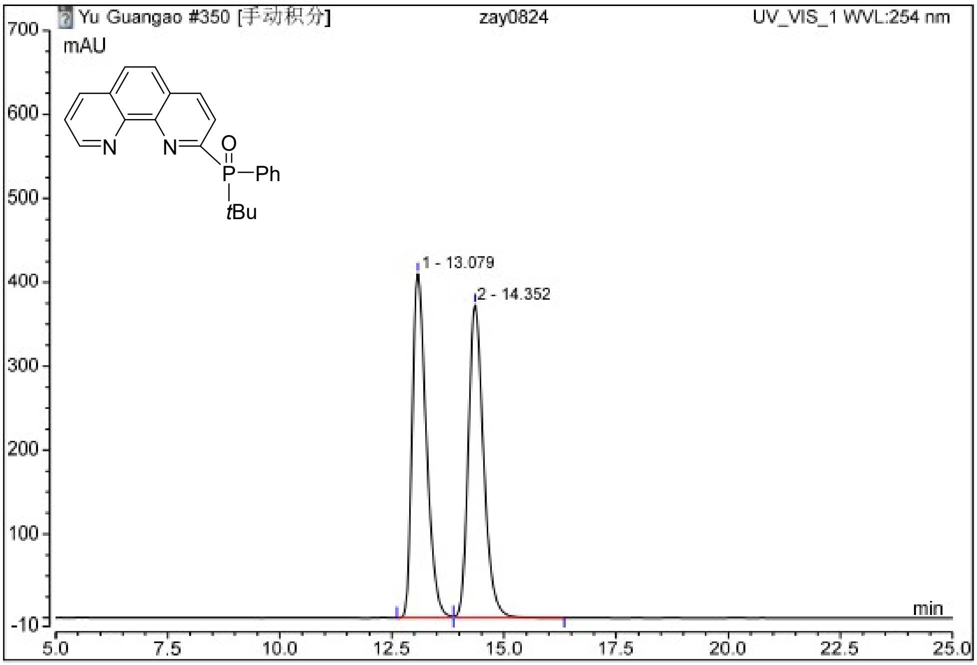

peak.number	peak.name min	peak.area mAU*min	peak.height mAU	peak.rel_area %	peak.rel_height %
1	13.079	148.869	410.438	50.23	52.37
2	14.352	147.500	373.348	49.77	47.63

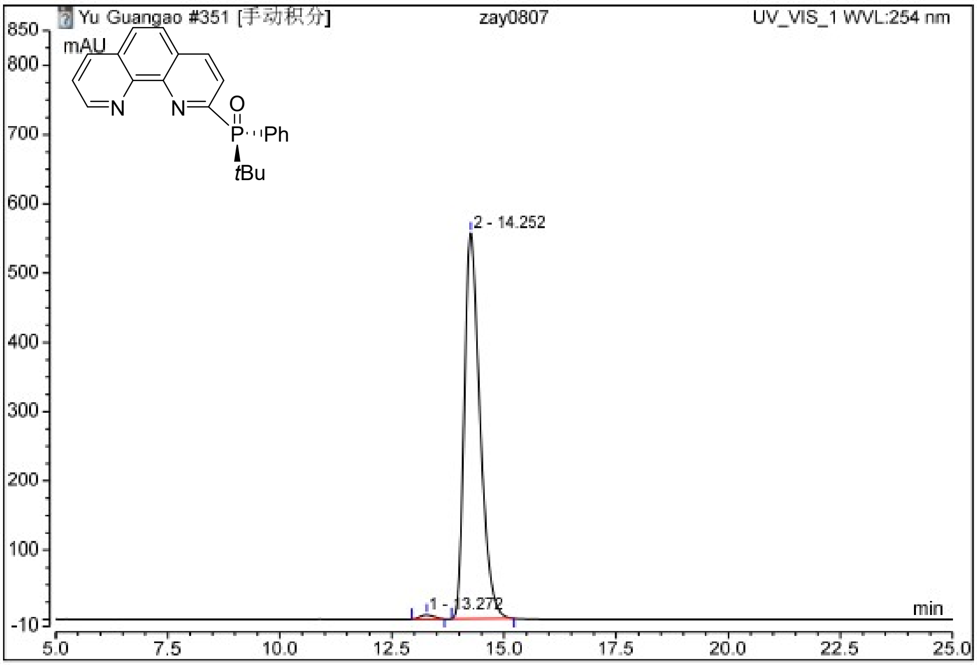

peak.number	peak.name min	peak.area mAU*min	peak.height mAU	peak.rel_area %	peak.rel_height %
1	13.272	2.194	6.224	0.97	1.10
2	14.252	224.872	557.049	99.03	98.90

Chiral HPLC chromatographic analysis of **34**

Condition: Daicel Chiralcel AD-H, *n*-hexane/*i*-PrOH = 85/15, UV = 254 nm, flow rate: 1.0 mL/min, retention time: t (minor) =13.172 min, t (major) = 25.485 min, ee = 97%.

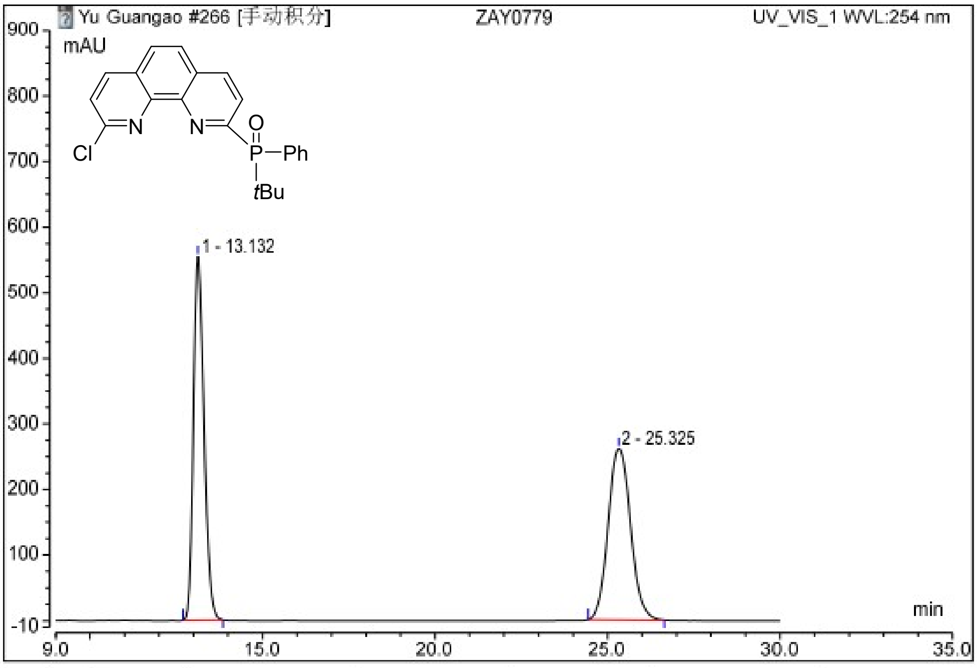

peak.number	peak.name min	peak.area mAU*min	peak.height mAU	peak.rel_area %	peak.rel_height %
1	13.132	195.936	554.348	50.09	68.00
2	25.325	195.225	260.928	49.91	32.00

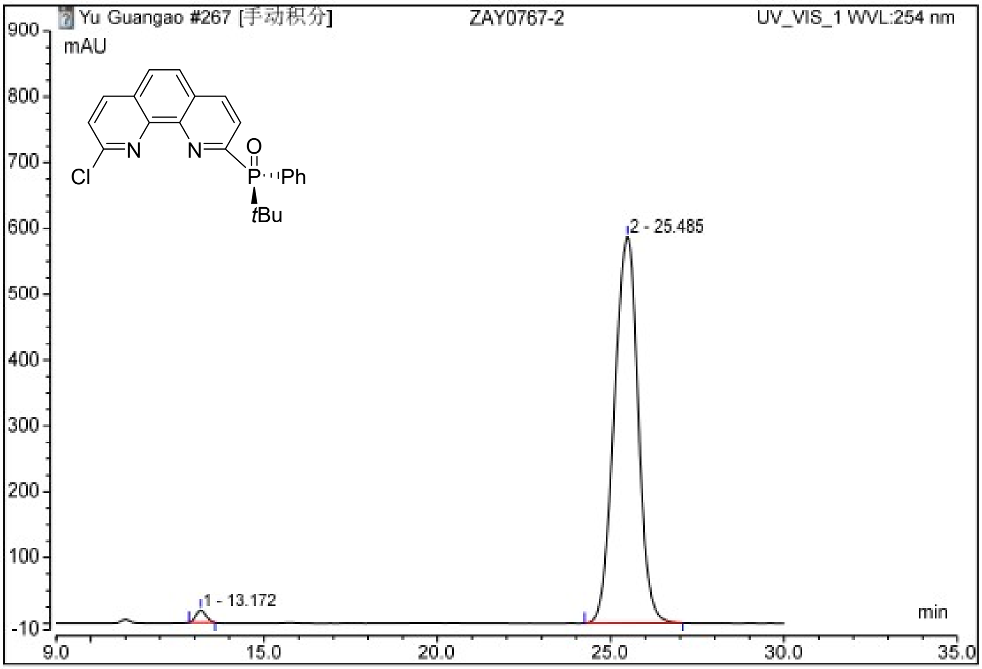

peak.number	peak.name min	peak.area mAU*min	peak.height mAU	peak.rel_area %	peak.rel_height %
1	13.172	6.231	18.697	1.31	3.09
2	25.485	468.307	586.843	98.69	96.91

Chiral HPLC chromatographic analysis of **35**

Condition: Daicel Chiralcel AD-H, *n*-hexane/*i*-PrOH = 85/15, UV = 254 nm, flow rate: 1.0 mL/min, retention time: t (minor) =9.919 min, t (major) = 11.079 min, ee = 99%.

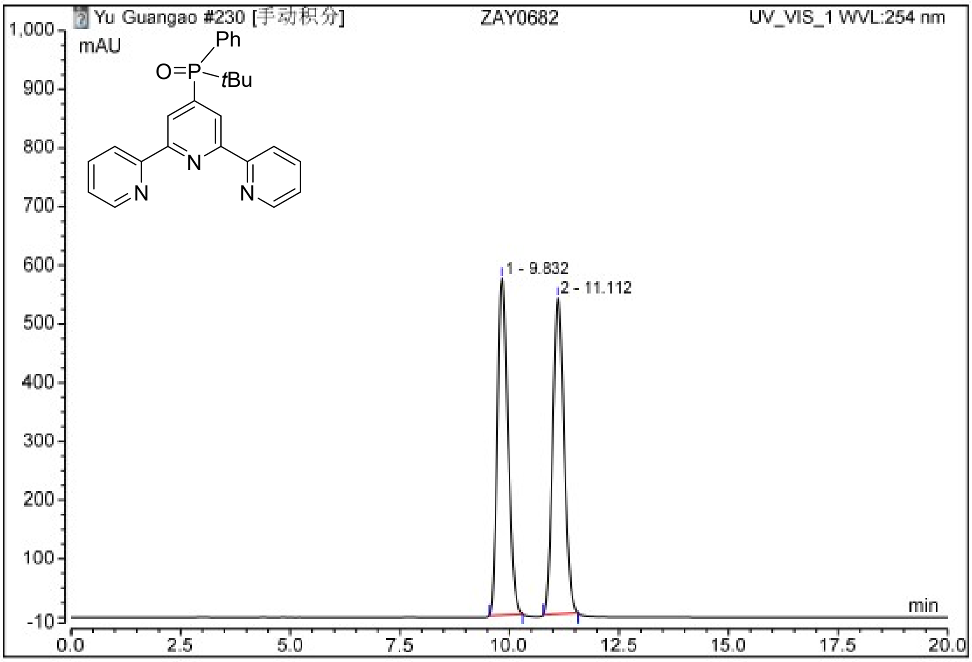

peak.number	peak.name<br>min	peak.area<br>mAU*min	peak.height<br>mAU	peak.rel_area<br>%	peak.rel_height<br>%
1	9.832	160.235	573.421	49.88	51.57
2	11.112	161.015	538.486	50.12	48.43

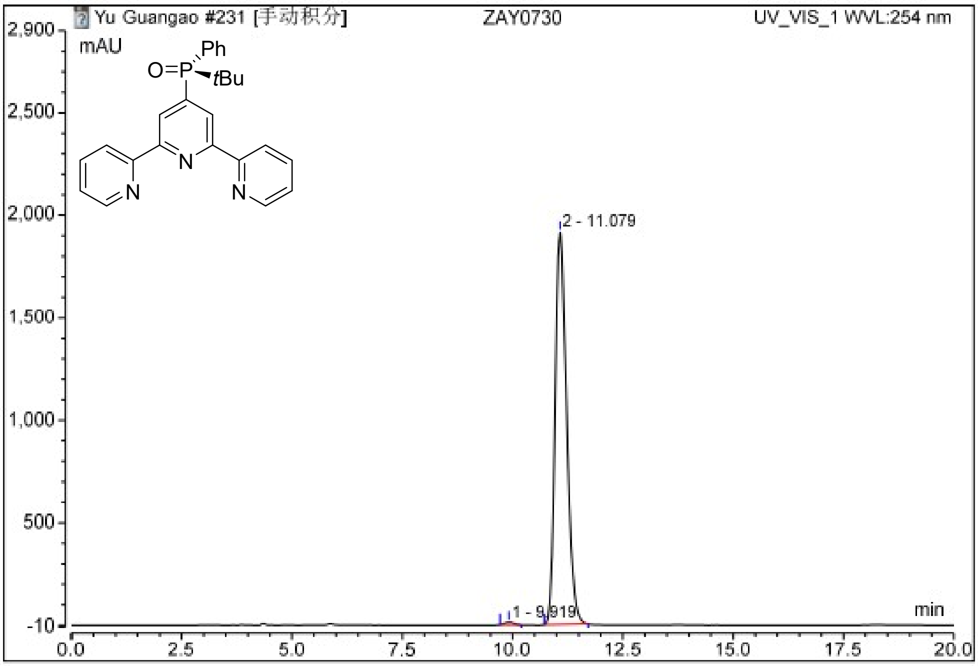

peak.number	peak.name<br>min	peak.area<br>mAU*min	peak.height<br>mAU	peak.rel_area<br>%	peak.rel_height<br>%
1	9.919	2.925	12.393	0.51	0.64
2	11.079	571.001	1914.993	99.49	99.36

Chiral HPLC chromatographic analysis of **39**

Condition: Daicel Chiralcel AD-H, *n*-hexane/*i*-PrOH = 85/15, UV = 254 nm, flow rate: 1.0 mL/min, retention time: t (major) =12.399 min, t (minor) = 13.739 min, ee = 98%.

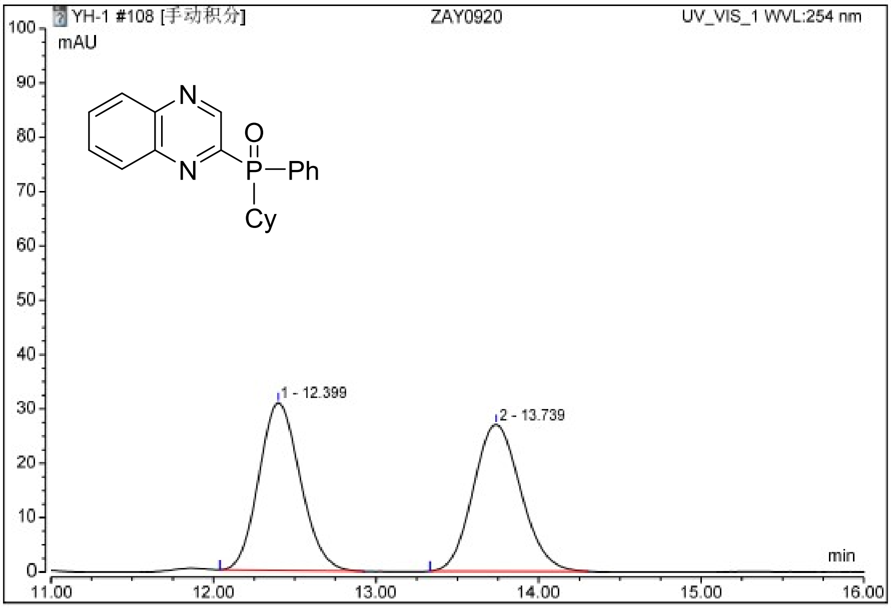

peak.number	peak.name min	peak.area mAU*min	peak.height mAU	peak.rel_area %	peak.rel_height %
1	12.399	9.144	30.843	49.90	53.31
2	13.739	9.179	27.009	50.10	46.69

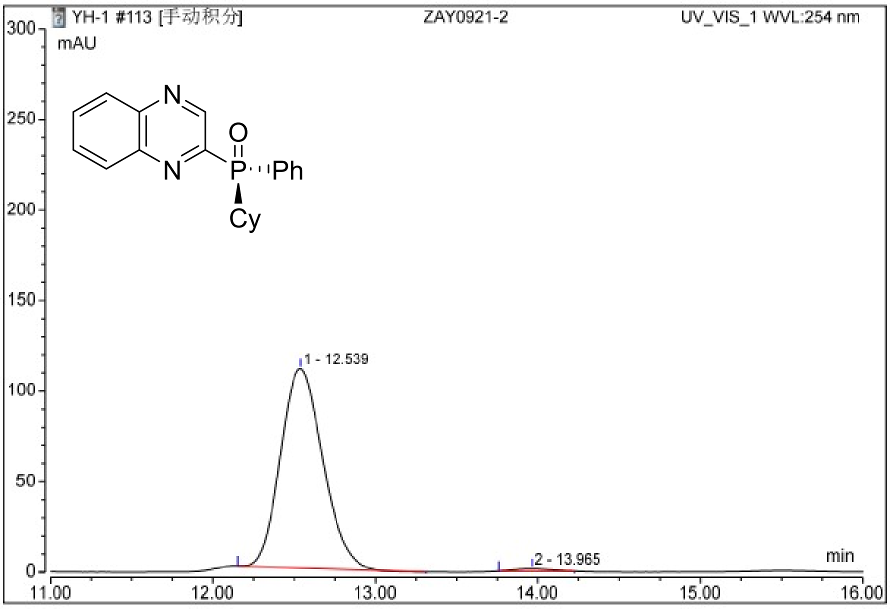

peak.number	peak.name min	peak.area mAU*min	peak.height mAU	peak.rel_area %	peak.rel_height %
1	12.539	33.178	110.056	98.98	98.84
2	13.965	0.343	1.296	1.02	1.16

Chiral HPLC chromatographic analysis of **40**

Condition: Daicel Chiralcel AD-H, *n*-hexane/*i*-PrOH = 85/15, UV = 254 nm, flow rate: 1.0 mL/min, retention time: t (major) =13.992 min, t (minor) = 17.232 min, ee = 97%.

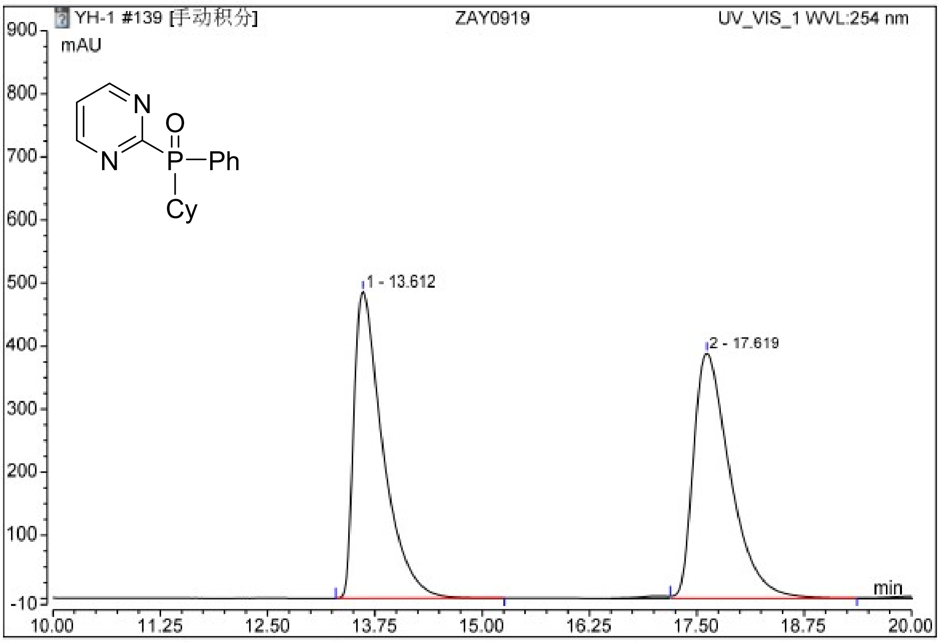

peak.number	peak.name min	peak.area mAU*min	peak.height mAU	peak.rel_area %	peak.rel_height %
1	13.612	190.403	486.557	49.70	55.58
2	17.619	192.714	388.845	50.30	44.42

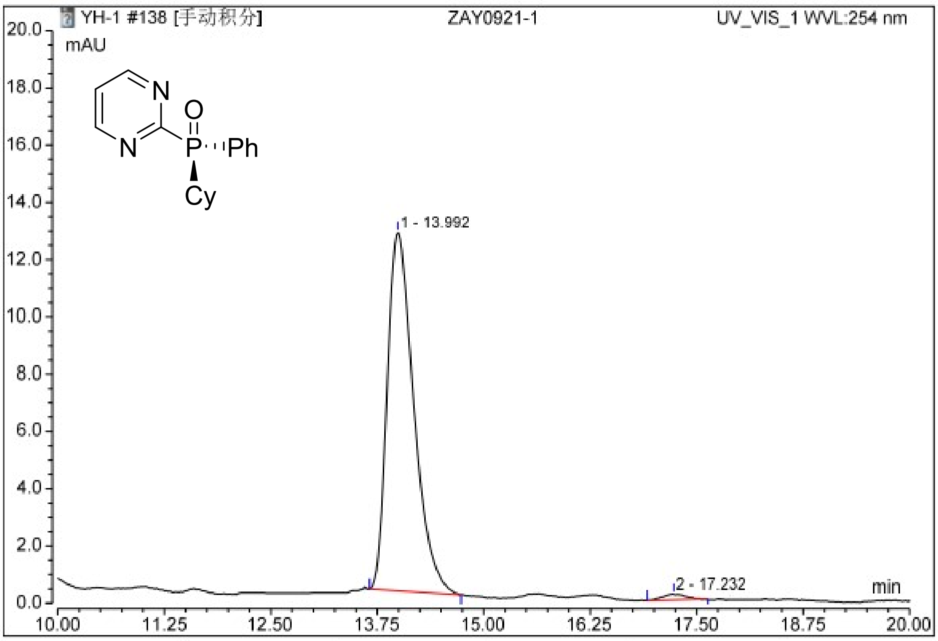

peak.number	peak.name min	peak.area mAU*min	peak.height mAU	peak.rel_area %	peak.rel_height %
1	13.992	4.518	12.527	98.67	98.42
2	17.232	0.061	0.202	1.33	1.58

Chiral HPLC chromatographic analysis of **41**

Condition: Daicel Chiralcel AD-H, *n*-hexane/*i*-PrOH = 85/15, UV = 254 nm, flow rate: 1.0 mL/min, retention time: t (major) =15.545 min, t (minor) = 18.132 min, ee = 99%.

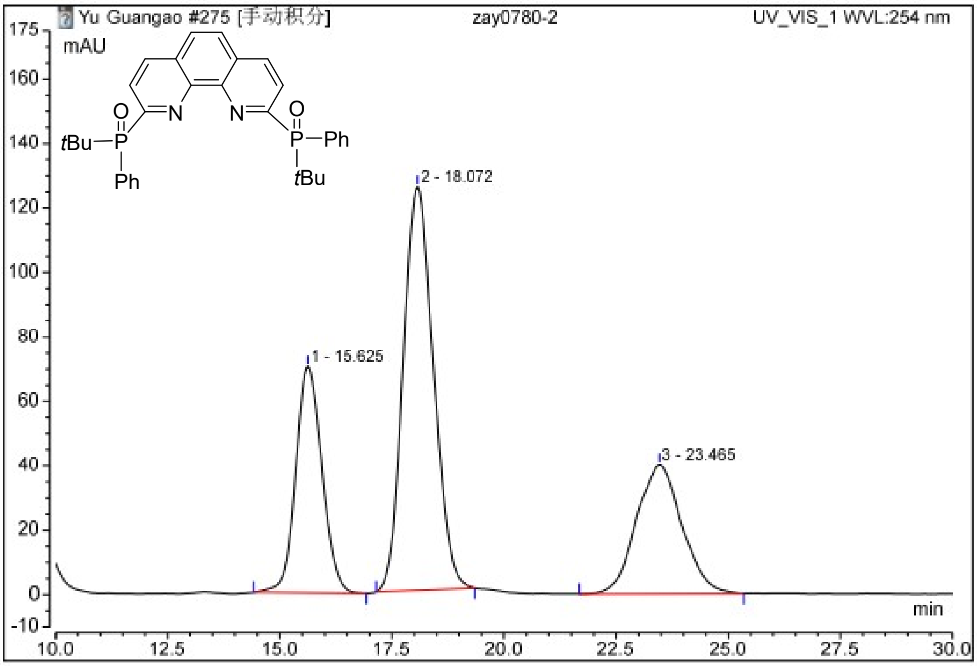

peak.number	peak.name min	peak.area mAU*min	peak.height mAU	peak.rel_area %	peak.rel_height %
1	15.625	47.575	70.283	24.76	29.84
2	18.072	97.478	125.219	50.72	53.16
3	23.465	47.125	40.064	24.52	17.01

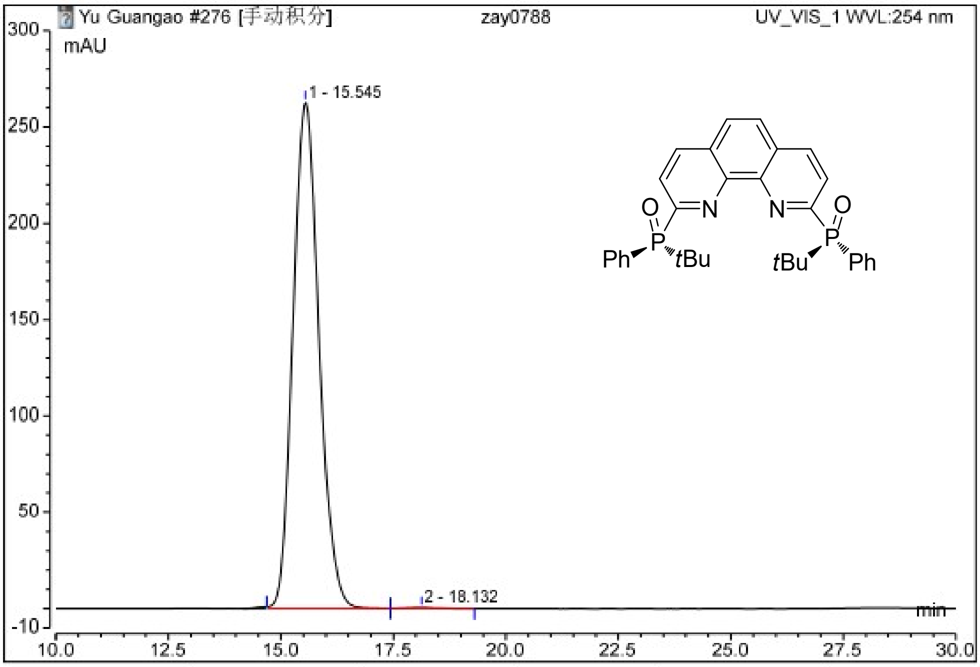

peak.number	peak.name min	peak.area mAU*min	peak.height mAU	peak.rel_area %	peak.rel_height %
1	15.545	176.286	262.911	99.70	99.76
2	18.132	0.534	0.624	0.30	0.24

Chiral HPLC chromatographic analysis of cyclohexyl(phenyl)phosphine oxide:

Condition: Daicel Chiralcel AD-H, *n*-hexane/*i*-PrOH = 85/15, UV = 254 nm, flow rate: 1.0 mL/min, retention time: t (minor) =9.605 min, t (major) = 10.992 min, ee = 98%.

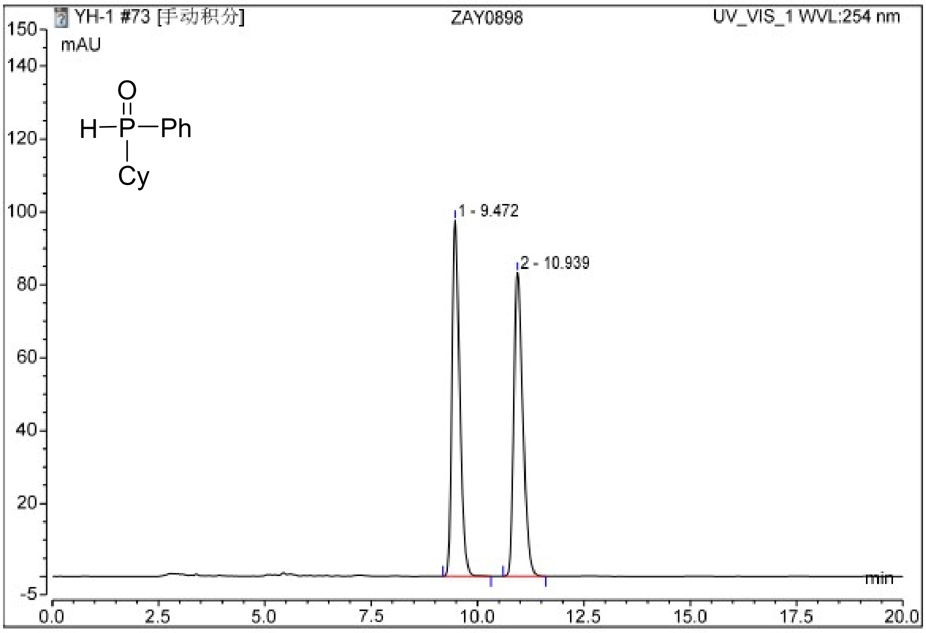

peak.number	peak.name min	peak.area mAU*min	peak.height mAU	peak.rel_area %	peak.rel_height %
1	9.472	20.612	97.532	49.97	53.93
2	10.939	20.637	83.305	50.03	46.07

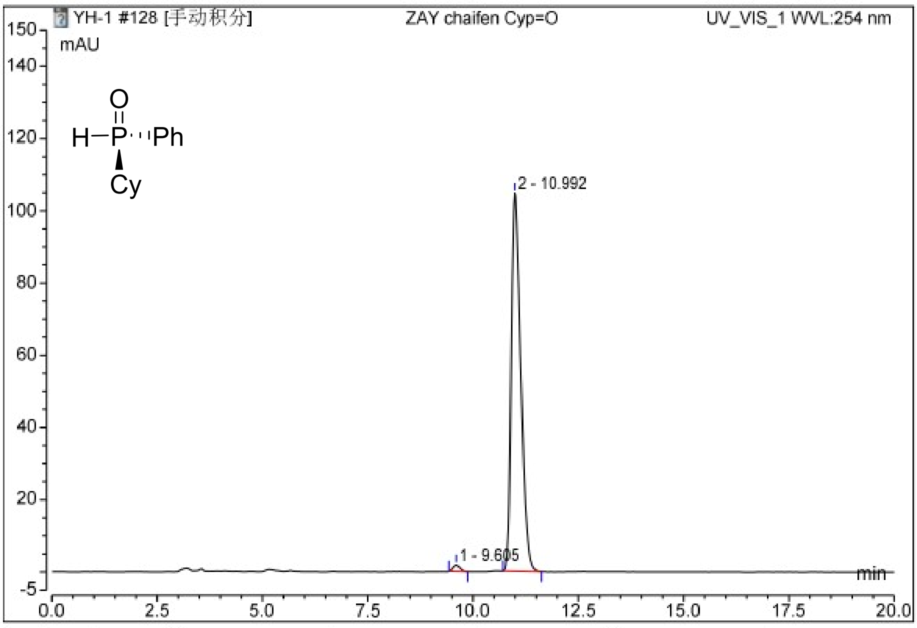

peak.number	peak.name min	peak.area mAU*min	peak.height mAU	peak.rel_area %	peak.rel_height %
1	9.605	0.348	1.816	1.26	1.71
2	10.992	27.342	104.653	98.74	98.29
